# Provenance based data integrity checking and verification in cloud environments

**DOI:** 10.1371/journal.pone.0177576

**Published:** 2017-05-17

**Authors:** Muhammad Imran, Helmut Hlavacs, Inam Ul Haq, Bilal Jan, Fakhri Alam Khan, Awais Ahmad

**Affiliations:** 1 Department of Computer Science, Sarhad University of Science & Information Technology, Peshawar, Pakistan; 2 Entertainment Computing, Faculty of Computer Science, University of Vienna, Austria; 3 Centre of Excellence in Information Technology, IMSciences, Peshawar, Pakistan; Nankai University, CHINA

## Abstract

Cloud computing is a recent tendency in IT that moves computing and data away from desktop and hand-held devices into large scale processing hubs and data centers respectively. It has been proposed as an effective solution for data outsourcing and on demand computing to control the rising cost of IT setups and management in enterprises. However, with Cloud platforms user’s data is moved into remotely located storages such that users lose control over their data. This unique feature of the Cloud is facing many security and privacy challenges which need to be clearly understood and resolved. One of the important concerns that needs to be addressed is to provide the proof of data integrity, i.e., correctness of the user’s data stored in the Cloud storage. The data in Clouds is physically not accessible to the users. Therefore, a mechanism is required where users can check if the integrity of their valuable data is maintained or compromised. For this purpose some methods are proposed like mirroring, checksumming and using third party auditors amongst others. However, these methods use extra storage space by maintaining multiple copies of data or the presence of a third party verifier is required. In this paper, we address the problem of proving data integrity in Cloud computing by proposing a scheme through which users are able to check the integrity of their data stored in Clouds. In addition, users can track the violation of data integrity if occurred. For this purpose, we utilize a relatively new concept in the Cloud computing called “Data Provenance”. Our scheme is capable to reduce the need of any third party services, additional hardware support and the replication of data items on client side for integrity checking.

## Introduction

The rapid and historic advancement in information and communication technologies (ICT) over the past two decades led to the vision that computing will one day become a utility like other traditional utilities, e.g., gas, electricity and water. Like all other existing utilities, this envisioned model of computing consists of commodity services. Such services are available for delivery to the end users at any time (on-demand) without the necessity of hardware/software infrastructure [1, 2]. In this model users access services and data according to their requirements without any concern to the underlying details like how and from where they are delivered. To make this vision a reality, several distributed computing paradigms like Cluster, Utility and Grid Computing have been established. One of the latest models, i.e., Cloud Computing gained significant interest from business and research community. It is based on the concept of distributed computing like its predecessors, however adding many specific characteristics of its own like huge scalability, on-demand model and pay-as-you-go pricing to name a few [3].

Data in Clouds is geographically dispersed which is frequently accessed by number of independent and remote users via Internet such as youtube. In such a shared and distributed environment, data moves from one point to another through communication networks. As the number of users and amount of data increases, the number of data transactions also increases. Significant interactions with this dispersed data increases the chances of data lost, alteration and unauthorized access. Ensuring the security and integrity of user’s data is one of the fundamental concerns of distributed environments such as Clouds [4–8]. Specifically in a research environment, trusting a particular dataset is essentially dependent on the quality of original data along with the services utilized for the transformation of original data into final output. In the current security conscious era, to maintain the quality of the data in such environments has significantly increased the importance of the concept of ‘data integrity’ [4, 9–11]. The outsourced data in Clouds relieves the owner from the management of data but they lose the physical possession of their data. In such an environment, the data integrity and the verification of data becomes an important and challenging task.

According to our research the problem of data integrity proofs in Cloud computing has not been investigated widely and is still in early stages [9, 10]. Several existing approaches like Third Party Auditors (TPA), mirroring and checksumming for data integrity proofs either rely on additional third party services or storing the same data on the client side for integrity checking [9, 12]. Such methods also lack the tracking of integrity violations when occurred. It is extremely important to track the integrity violations for experiments conducted in Clouds for various purposes like reproducibility, verification and audit trials [13].

In order to facilitate Cloud users with an easy and economical way for checking data integrity and violations tracking, we need a comprehensive solution combining several different techniques which rely on the local resources of the Cloud environment. For this purpose we address the problem of data integrity proof by utilizing a powerful local resource of the Cloud called ‘Data Provenance [14]. It is generally defined as “the place of origin or earliest known history of something. Provenance has been investigated in different fields of life like art, medicine, business, science and technology [15–17]. In computational and data science, it is described as the information acquired about processes and original dataset that are used to produce the final result [18, 19]. Various definitions of provenance in different domains of communication and information technology conclude that it can answer many questions about the history and state of a data product such as: (i) From where a data product was acquired? (ii) By whom and when the data product was created? (iii) Who are the authorized stakeholders of the concerned data product? (iv) In what transformations and computations it has been used? (v) What were the inputs for a generated output data item? (vi) Which criterion was applied for generating a data product? The answer to such questions is possible by recording the related information at each stage of the data product life cycle in Clouds. Therefore, any breaches in data integrity can be effectively detected. The contribution of this paper is mainly divided into following parts:

- Collecting, storing and managing provenance information for different layers of Cloud computing.
- Utilizing the provenance information for integrity checks of the original data.
- A sample application and use case scenario proving the usage of provenance for the verification of data.
- Collection and storage overhead for the provenance data of the sample application and the underlying Cloud environment.

The rest of the paper is organized as follows. Section *Related work* provides the literature review of data integrity assurance and proofs in on-premise as well as in Cloud computing. Section *Limitations in existing schemes* discusses the limitations in exiting schemes whereas Section *proposed solution* details our model of using data provenance for integrity verification. Section *Architecture of Integrity Tracker* provides the architecture of Integrity Services created in the proposed solution. Section *Verification* uses a sample application to prove the utility of proposed solution. Section *Performance result* discusses the overhead in terms of provenance collection and storage. Section *Limits* discusses some limitations of our work with future work directions and Section *Conclusion* concludes this paper.

## Related work

The term data integrity has different aspects such as quality, safety, alteration and flow of information between different entities. The most general definition given by Courtney and Ware [20] is the data quality definition which deals with the expected quality of the data. This means that the data has integrity up to some extents where the expected quality meets or exceeds. According to the context and interest of individuals, it is defined by various authors differently [21–23]. The basic meaning of the term ‘integrity’ is quite self-explanatory and it can be said, *integrity* is to assure that something is what it expected to be [24]. According to this simple definition of the term integrity, *data integrity* can be briefly defined as a specific state of the data which is expected by a consumer.

Assuring data integrity for storage systems has been the focus of research in the last two decades. The advent of Cloud computing gives it a new direction to be investigated for dispersed environments. Here we present some state of the art data integrity assurance techniques developed for on premise storage systems as well as for distributed environments such as Cloud.

Sivathanu et al. [25] discussed the three most familiar techniques for data integrity assurance in storage systems. These include Mirroring, RAID Parity and Checksum. In Mirroring, data integrity is checked by comparing already stored multiple copies of same data on different devices. This method is inefficient in terms of space and time because storing multiple copies of same data requires more physical storage space and the comparison of large data items is more time consuming. The RAID (Redundant Array of Inexpensive Disks [26]) Parity is another technique for off-line integrity checks having different levels of storage. In RAID the parity of disks in redundant arrays are computed diagonally. The integrity of the stored data is validated by performing the XOR (Exclusive OR) operation on the computed parity. This method is dependent on specialized hardware and also does not support on-line integrity verification. In the checksum method, checksum values are computed for the stored data using hash functions and stored gradually as the data arrives on the disks. The integrity of data is verified by comparing the stored and the newly computed checksum values on every access. The limitation of this approach is computation overhead because it computes the data integrity on every access of data item. If large numbers of such data items are accessed frequently, the data integrity checking will need more computational power and processing time.

Xie et al. [27] proposed a probabilistic method for data integrity assessment. To examine integrity of the data by this method, a small number of testing tuples are inserted in the outsourced data, when a query is issued for some data object, they assume that there is certain probability that a small number of inserted tuples are returned with the original data. The integrity of data is monitored by analyzing the inserted tuples received in the query response. All data items moving to the service provider are encrypted; hence the service provider cannot distinguish between the original data and inserted tuples. For this reason the clients must maintain a copy of the inserted tuples in order to know the set of tuples returned in the response.

Kumar and Saxena [28] proposed a scheme for data integrity proof in Cloud storage. The proposed method selects some bits randomly in a data block as meta-data. The computed meta-data is then encrypted and appended to the data item. On data integrity verification request of client, verification phase is activated by coining a challenge to Cloud archive and wait for the response. The received response is then compared with the challenge and the result is used for accepting or rejecting the integrity proof of the concerned data item. In this method multiple hash values are maintained on client side which increases the computation overhead on client. Moreover it only considers the static storage of data where as in modern Cloud storage systems the dynamics of the data is very important. This enables enterprises to interact with their data on the Cloud, process it and change it according to the business needs.

Luo and Bai [9] proposed a scheme for data integrity verification on Cloud storage. This scheme is composed of four algorithms KeyGen, SigGen, GenProof and VerifyProof. These algorithms are responsible for key generation, metadata generation (signature), data storage proof generation and verification of the generated proof of data storage respectively. Their proposed scheme works in two phases called SetupPhase and AuditPhase. In Setup Phase public secret parameters of the system are initialized and meta-data of the target data object is generated by KeyGen and SigGen respectively. After this process data file is then stored on the Cloud and its meta-data is published to the third party auditor (TPA). In second phase the TPA issues an audit message to the Cloud server to make sure that the Cloud server preserves the specified file in its original form at the time of the audit. The Cloud server calculates a response message by GenProof for target file. This message is received by the third party auditor where it is verified by Verify-proof algorithm. The security analysis conducted by the authors show that the proposed protocol is secure against the server and capable of preserving the data file privacy against the third party auditor (TPA), but the auditing service in the proposed approach is still in the control of an independent entity which is not a part of the Cloud. The failure of third party auditing system due to any reason can be dangerous for the Cloud data integrity. This also increases overall cost of service provided by the Cloud provider to its users.

Adam Bates et al. [7] provide a mechanism to use provenance as an access control for cloud environments. However, their work assumes that provenance meta-data is provided by end hosts. In case of incorrect provenance, the system might suffer critical problems. In our work, we automatically collect and manage provenance meta-data inside the cloud for the different layers. We also focus on the violation of data integrity and its verification. Similarly, Akhtar et al. [29] provide a mechanism to secure data provenance in cloud. This can be used as an extension to our work for securing the provenance storage.

## Limitations in existing schemes

In summary, the existing schemes of data integrity for both on-premise and off-line storage have limitations such as efficiency problem, requirement of special hardware and dependence on TPA. Moreover such techniques lacks the tracking of integrity violations. Table 1 presents a conceptual matrix where limitations of existing schemes as well their compatibility with Cloud is presented. In a brief manner, our work is focused on the following open issues.

- Finding the cause, origin, history and time/date from which the integrity violations has occurred
- Dependency on TPA for integrity proof is expensive and it is the end user who ultimately suffers in case of any failure. Our proposed solution removes this dependency.
- Client side data storage and computations need sufficient hardware/software resources on the client side which violates the basic theme of Cloud computing.

**Table 1:** Limitations and compatibility issues of existing integrity schemes in Clouds.

Technique	Limitation	Cloud Compatibility
Mirroring Technique	Efficiency problem in terms of storage space and computations	Not tested in Cloud
RAID Parity	Need specialized hardware	Not Tested in Cloud
Checksumming	Computation overhead	Not tested in Cloud
Data Integrity Proof in Cloud Storage [25]	Dependency on client computations	Tested in Cloud
Ensuring Data Integrity in Cloud Storage [9]	Dependency on outside third party	Tested in Cloud

We propose to solve the above mentioned issues for end users with an economically feasible and comprehensive solution for data integrity proof and tracking violations in integrity. For this purpose we investigate the use of data provenance, a local resource in Cloud environments. The proposed solution utilizes the existing resources in such a manner that the underlying architecture of Cloud services is not altered.

## Proposed solution (Utilizing provenance for data integrity)

In this section, we present our Provenance Based Data Integrity Checking and Tracking Scheme for Cloud data. As the name illustrates, provenance information are utilized for data integrity proofs and tracking any violations.

The existing schemes of data integrity in cloud such as Provable data Possession (PdP) [30], Proof or Retreivabilty (PoR) [31], High Availability Integrity Layer (HAIL) [32], and using Third Party Auditors rely on methods like key generation algorithms, cryptographic techniques and replication of data [33–37]. Schemes in the PdP and PoR category work on file or block level and require computation overhead because of key generation algorithms. Schemes in the HAIL category rely on replication of data items which adds to the huge storage overhead. TPA adds privacy issues regarding data because of the involvement of third party. Our proposed scheme is designed to address such issues.

The proposed scheme uses the provenance data i.e. the flow of actions which are performed on the original data uploaded to the cloud. The scheme keeps the history of information such as adding, deleting and updating files in cloud storage. We use this historical record to find any suspicious behavior regarding the data stored in cloud. Therefore, our scheme does not rely on replication of data or executing key generation algorithms.

The proposed scheme is different than the existing schemes in the sense that we do not rely on any third party auditors, replication of data or computation of key-hash values for integrity checks. To perform integrity checks based on provenance data, we have identified some key elements across various layers of cloud (described later in this paper). The proposed scheme is based on the following properties: less storage and computation overhead, no dependency on specialized hardware and client side, no need of third party auditors and, no need to change the inherit architecture of cloud.

**Fig 1:**
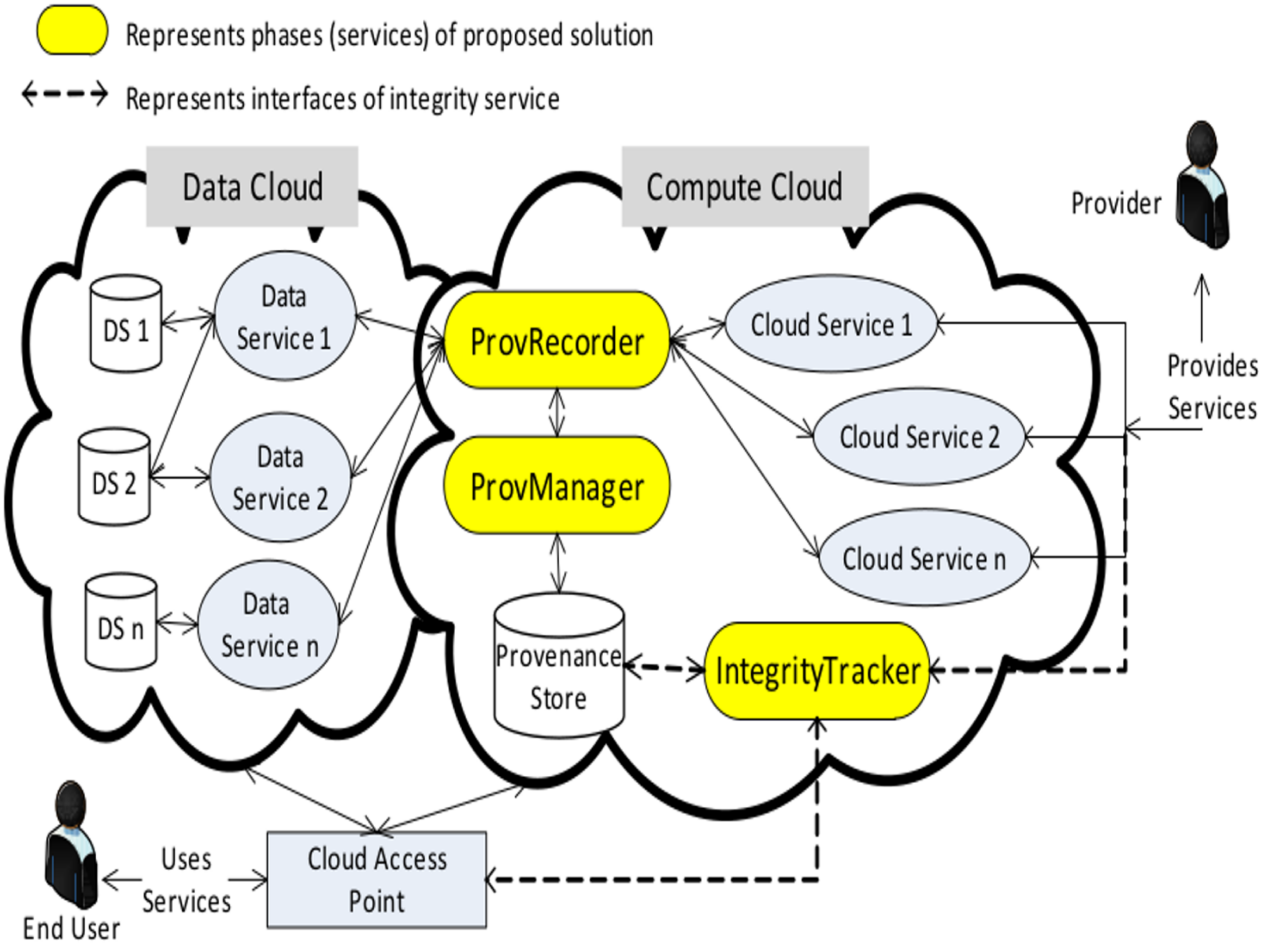
Black box architecture of provenance based data integrity verification.

The scheme is divided into three major phases i.e **ProvRecorder**, **ProvManager** and **IntegrityTracker** as shown in Fig 1. The ProvRecorder phase collects various important information (provenance) related to data items which are created in Cloud. The DataManager phase manages the collected information according to the different operation such as deletion and modification performed on data item. The DataIntegrity phase verifies any violations like unauthorized access to the data items. Components of each phase are developed and implemented as services and published to the Cloud to serve the end user. The subsections below provide the details of each phase and related components.

### ProvRecorder

This first and significant phase of the proposed solution is to record the history information of data items, i.e., provenance for finding any integrity violation. In our previous work [38, 39], we developed a provenance framework which is based on the concept of interception in Service Oriented Architecture (SOA). The framework was deployed and tested for different middlewares like Apache Axis2 and Mule. These middlewares are used in Cloud environments like Eucalyptus and Nimbus for the communication and interaction mechanism between services. Since Cloud is based on SOA, the previous work was utilized to collect important and significant provenance information for data items stored in Clouds.

The collection of provenance is achieved at three different abstraction layers, i.e. client, server (Cloud), and middleware of Cloud. For instance, when a user uploads a data file to the Cloud, various information are collected regarding the file such as:

1. name and size of the file at the *client* layer,
2. user name (owner) and location of the file at the *server (Cloud)* layer, and
3. service name and timestamps (for different operations) at the *middleware* layer.

**Fig 2:**
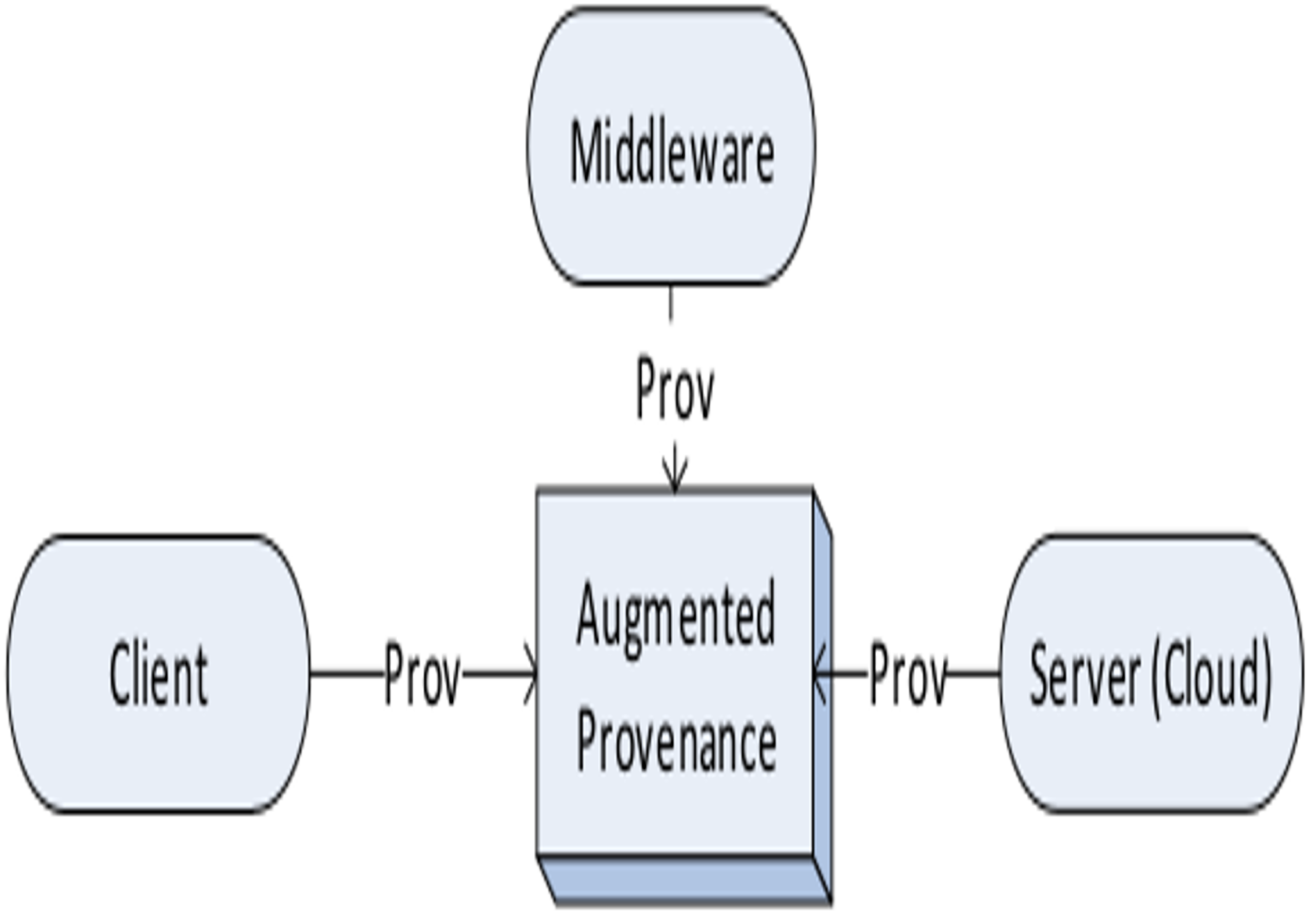
Collection of provenance data from different layers of abstraction in Clouds.

Fig 2 depicts the augmented provenance recorded at different layer of abstraction in a Cloud environment. The augmented provenance is forwarded to the *ProvManager* phase for further processing.

### ProvManager

In phase-II of the solution, the Provenance Manager (ProvManager) is executed on the recorded provenance data. The job of the *ProvManager* is to store and manage various provenance data according to the layers, i.e., client, Cloud and middleware and for different operations such as creation, updation, and/or deletion [40]. For each new arrival of a data item to the Cloud storage, a tag named *Item-ID* and its corresponding value, i.e., an Attribute-Value pair is added to the provenance storage along with timestamps information. Various attributes and their description is provided in Table 2. It is to be noted that Table 2 provides only a subset of the information collected in our solution.

**Table 2:** Various attributed with their description and corresponding layer of Cloud environment.

Layer	Attribute	Description
Server (Cloud)	Item-ID	Unique ID of date item stored in the Cloud storage
	OwnerID	Unique ID of the data item owner
	ItemAccessCounter	A Counter that depicts how many time a particular item was accessed
Middleware	CreationDateTime	Date and Time of arrival for storage
	UpdateDateTime	Date and Time of last change of data item and hash value
Client	InitHashValue	Initial hash value computed at arrival for storage

The attributes values are read from the recorded provenance and written to an XML file for future use, i.e., detection of data integrity violation and tracking. The location of the XML file can be set to the same Cloud storage where original data is stored, or, in a separate and independent location [38, 41]. Both the schemes have their pros and cons. For instance, storing provenance data along with original data provides ease of access, low computation overheard and no network traffic. Storing provenance data outside (in an independent location) the original data provides more security as in case the original hard drive crashes or malfunctions. We chose to store XML files along with the original data because of ease of access and low computation overhead.

The design of an XML file is chosen in a specific format where various related items are grouped together. This grouping is based on different items and their corresponding metadata values such as users with name and group name, files with attributes, and services with their input and output parameters. The grouping of different items in the specified format provides efficient searching and traversing [42]. For instance, users can search for integrity violations of different items based on parameters such as type, size, owner, and/or combination of the parameters. In summary, XML is chosen to represent and store provenance information because of the following reasons:

- Portability: The data can be ported to any platform. In addition, custom algorithms can be designed to port the data into any database
- Lightweight: We keep only significant provenance information
- Efficient Searching: Customized algorithms can be designed to provide efficient search mechanisms

The proposed scheme extends the existing middleware utilized in cloud for the collection of provenance data i.e *ProvRecorder*. A separate module i.e. *ProvManager* is established for storing provenance data into storage unit and its management. This module is directly linked with the collection part of the scheme. The proposed scheme does not change the architecture of the cloud but extend it using the built in features i.e. interceptor and module. Such extension automatically handles (being part of the existing middleware) any number of data items being modified in the cloud. Therefore, the proposed scheme utilizes the existing features of the middleware which handles updates regardless of the numbers of data items and the frequency of modification.

### IntegrityTracker

**Fig 3:**
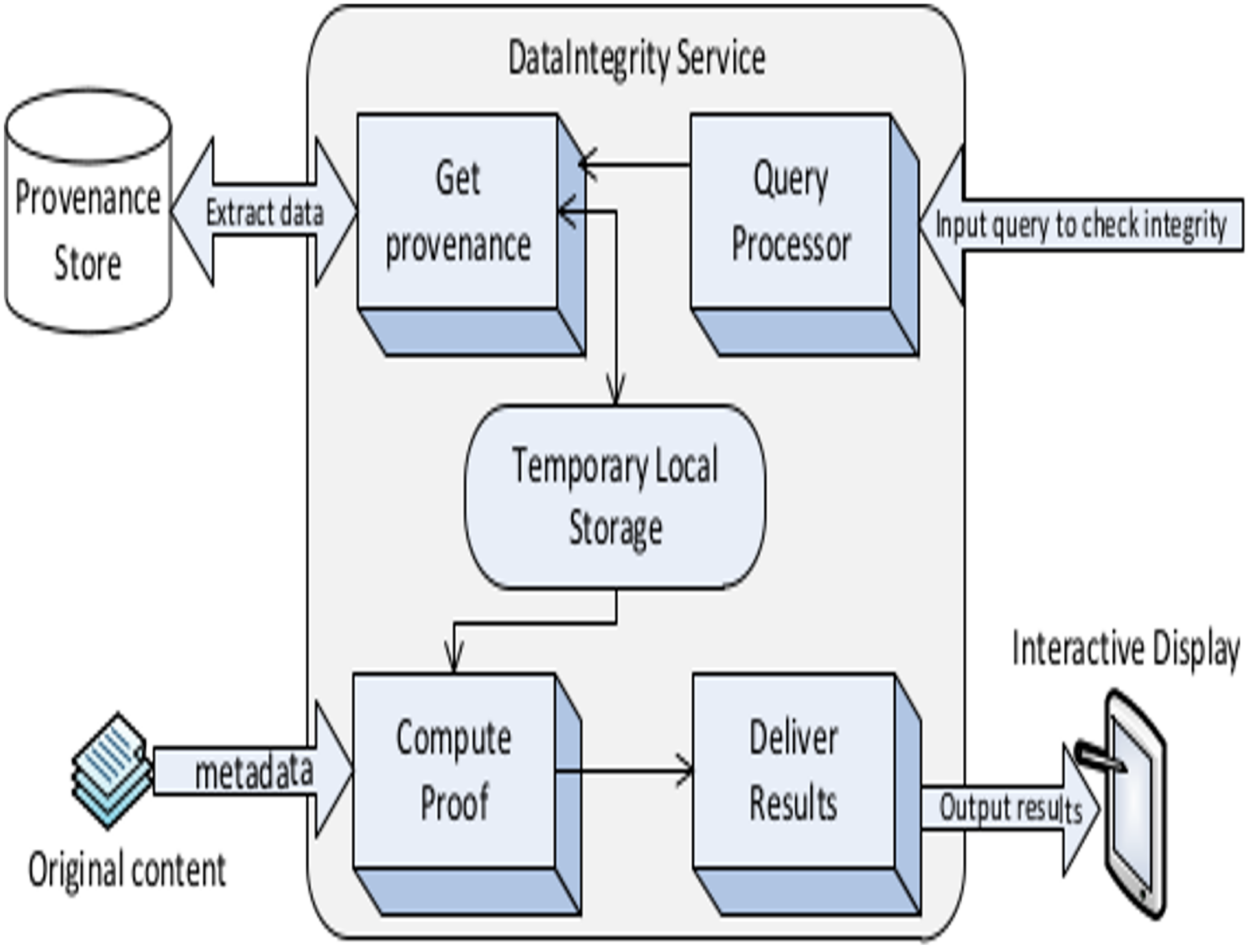
Various components of integrity service (integrity tracker phase).

The *IntegrityTracker* phase of the solution is executed on the stored provenance data for checking any violations. It is achieved via developing and publishing a web service named *Integrity Service* to the existing Cloud environment. The integrity service provides three different interfaces for interaction as shown in Fig 3 by dashed lines. The first interface interacts with the provenance store and retrieves significant data for integrity verification. Various decisions are made based on the retrieved information from the provenance store. The second interface is utilized by the end user for input any query, i.e., data items for which integrity check is required. This interface is also utilized for the output, i.e., generated results. The third interface is for the administrator for any modification in the service such as updated version. The overall process, i.e., different phases of the solution avoids any TPA services and extra hardware support for integrity checking. The integrity service itself is divided into many components which are detailed in the following section.

## Architecture of Integrity Tracker

The integrity service implements a set of algorithms (components) that include *QueryProcessor*, *GetProvenance*, *ComputeProof*, and *DeliverResults* as shown in Fig 3. The subsections below provide the details of each component.

### QueryProcessor

QueryProcessor accepts requests from Cloud users for the verification of data integrity. The request query is generated based on the end user selection. The selection can be simply a document name or a combination of different parameters such as: (i) users and groups, (ii) Access Control Policy (ACP) of the content in Cloud, and (iii) item size, type and/or their location etc. Therefore various parameters are set by the end user in the request query. Such options are made available to the end user so he/she can customize the request query and therefore checks for the integrity of selected content. The creation of a successful query is forwarded to the *GetProvenance* component.

### GetProvenance

**Fig 4:**
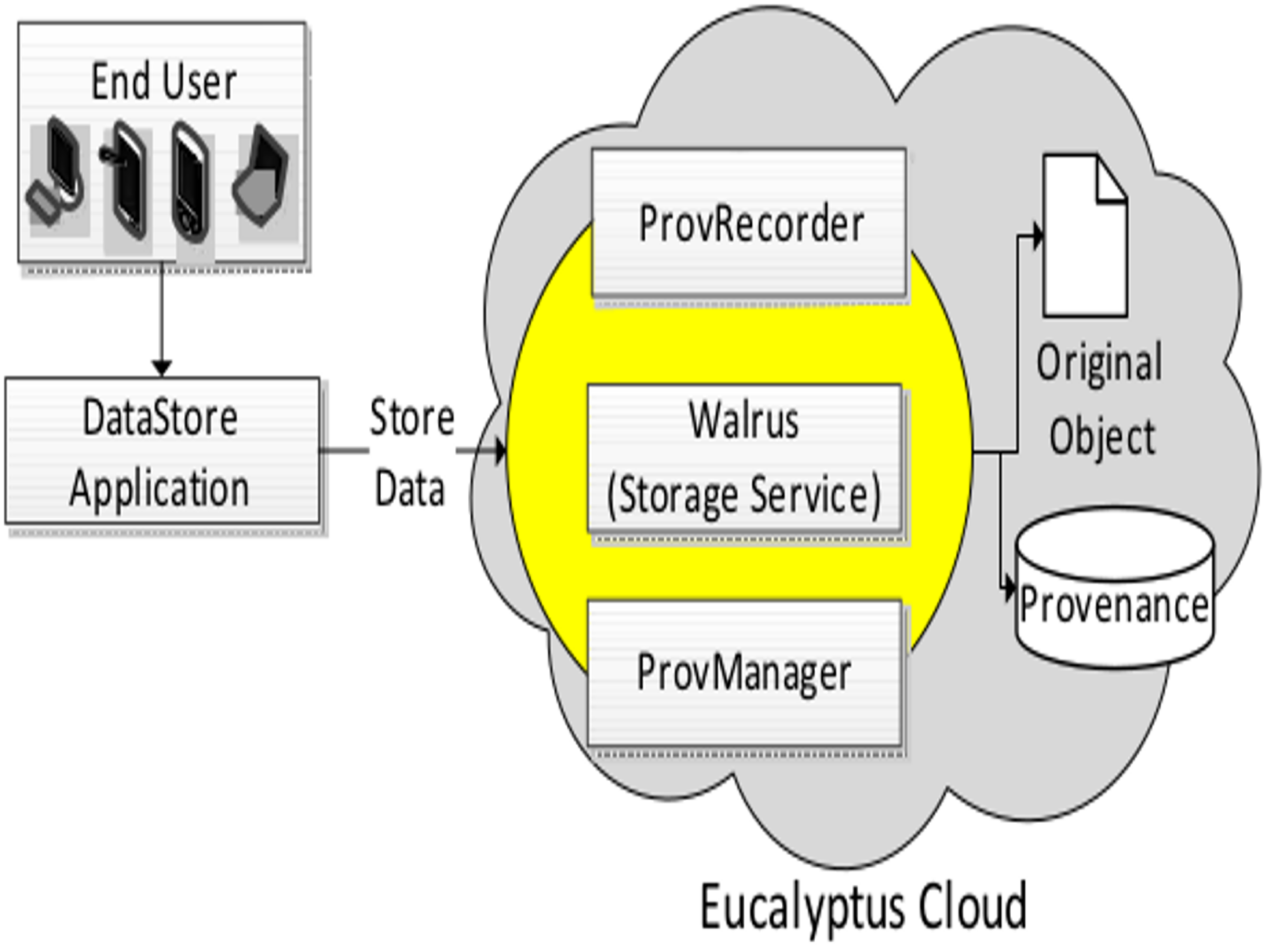
Architecture of *DataStore* application with different layers of abstraction.

The GetProvenance component accepts the query from QueryProcessor. The GetProvenance component has an interface to the provenance store as shown in Fig 4. This component executes the query and extracts the related information based on users selection which includes the data product name, identification number, name of the owner, the original location from where it was generated, last accesses time, and the last known size of the data product amongst others. These extracted results are then stored in a temporarily storage where the next component (*ComputeProof*) can use it for computing the integrity proof.

### ComputeProof

ComputeProof is one of the key components of the data integrity service. When the provenance data is retrieved for a user query, ComputeProof is executed which is responsible for generating an integrity proof. It is achieved by comparing the provenance information extracted in *GetProvenance* stage with the metadata of the original items stored in Cloud storage as shown in Fig 4. The outcome of the ComputeProof indicates whether the integrity of the particular data product is violated or not. In case of an integrity violation it also tracks the source such as by whom and when the item was modified. The results are forwarded to the *DeleiverResults* component.

When different users are adding, deleting or modifying data items, the provenance data is stored and organized using the time stamp of operations performed by different users. Hereby, the integrity of data is defined by arranging the data using the time stamp information (flow of actions). It is to be noted that retrieving the provenance data is established using the i) time stamp information, ii) users information and, iii) data itself. The first method i.e. retrieving provenance based on time stamp information shows a flow of actions performed by various users in the cloud storage. We check the integrity of data using the flow of actions performed by various users and their actions using the timestamps information.

### DeliverResults

DeliverResults is the last component of the integrity service. It is responsible for converting the results of generated proof into a user readable form. The communication with the end user is achieved via graphical user interface using HTML and CSS. The flow of information inside the Data Integrity service is shown in Fig 4.

## Data integrity verification by using *DataStore* application

**Fig 5:**
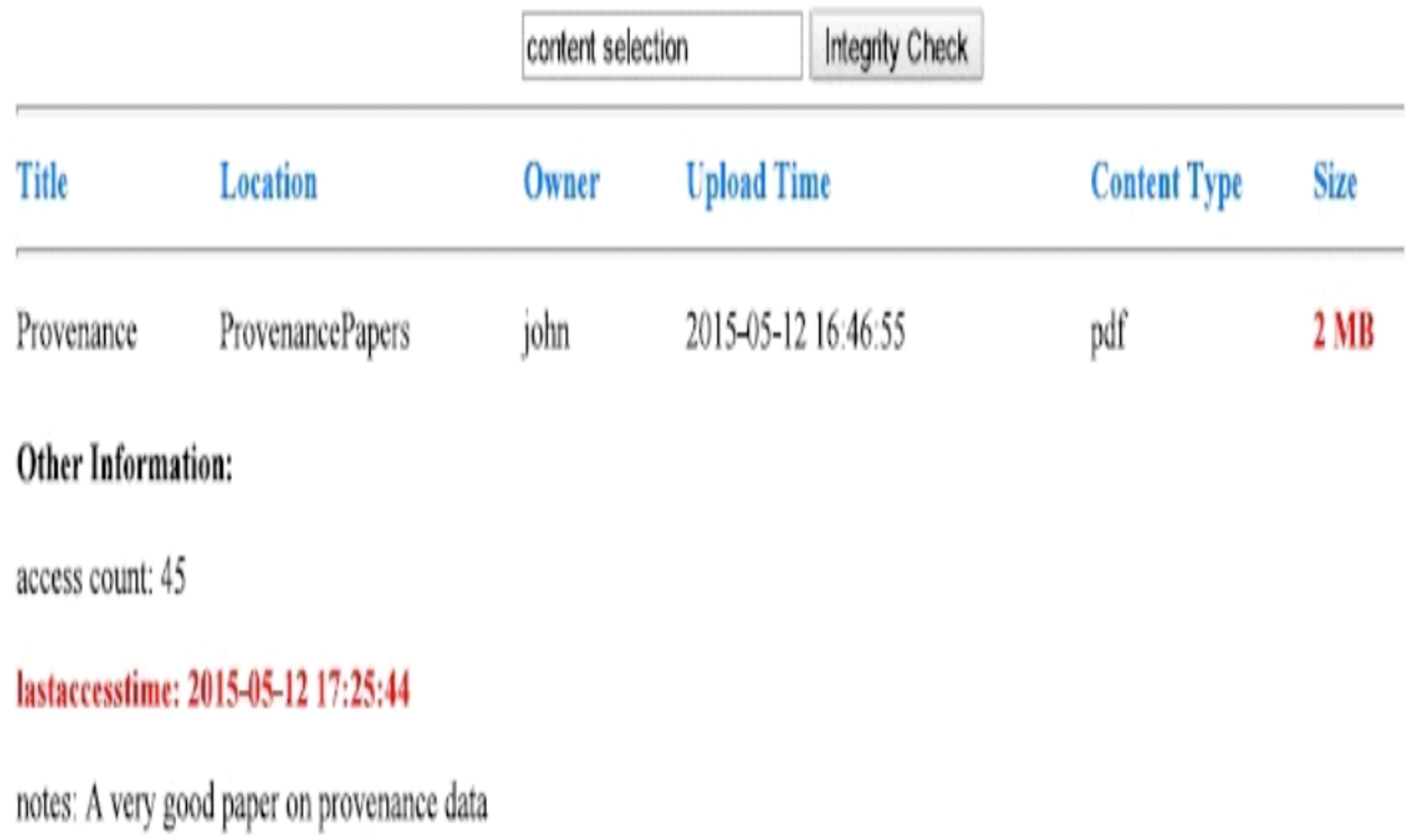
Visualization of integrity leaks to the end user.

Clouds are designed to be abstract and various layers of functionality are hidden from the end user. Here we present an application named *DataStore* that is utilized by end users to store their documents in Cloud storage, e.g., dropbox [43]. The basic architecture of the service is depicted in Fig 4 where different layers of abstractions, i.e., end user, client application and Cloud architecture are depicted. We used the Eucalyptus Cloud as our infrastructure and the Walrus service for data storage. Additional services, i.e., ProvCollector and ProvManager are published to the architecture of the existing Cloud which interact with the Walrus Service. By using *DataStore* application within the environment of Cloud, we present a usecase involving different steps for the verification of data integrity as following:

- **Step 1 (Upload Content):** A User selects various files of different size and type at the client machine and uploads them to the Cloud storage using *DataStore*. The files are uploaded to the Cloud with the additional services in action, i.e., ProvCollector and ProvManager. Therefore, provenance information is collected and stored accordingly into provenance store for each file.
- **Step 2 (Modify Content):** We used a manual approach to modify the information (metadata) of original documents stored in Cloud. We changed different metadata like owner name, security permission, location, and number of pages of a file amongst other. We were able to perform this operation because the files are stored inside our private Cloud. In a real environment, such a situation happens because of threats like hacking, malicious softwares, data loss, hardware failure, and unauthorized access etc.
- **Step 3 (Integrity Proof):** Lastly we focused on the integrity proof. Here we used our *Data Integrity* service to compare the provenance information with the original data. As soon we retrieved the provenance information, the misplaced, missing or changed data was highlighted. Fig 5 depicts the difference between the original data and provenance information, i.e., integrity proof (using bold and red text). It is important to note that if the services which are used to store data in Clouds are hacked (unauthorized access); our provenance store will also contain information of the IP address from where such changes were made (tracking). Such an environment can be used to block suspicious IP addresses or users for the purpose of intrusion detection.

## Performance results

The DataStore application consists of three major services i.e. *ProvRecorder*, *ProvManager* and *IntegrityTracker*. The first two services are extra layers of integration in the Cloud environment and therefore they create extra overhead. The *ProvRecorder* adds computation overhead while collecting provenance from different layers of abstraction of Clouds. The *ProvManager* adds overhead because of the provenance management such as storage of provenance (storage overhead). The *IntegrityTracker* service mines the provenance storage and displays any integrity violation to the end user. The results are calculated using a client/server architecture of three machines with various components of Eucalyptus Cloud installed on individual machines for which the details are provided in Table 3. The subsections present the collection and storage overhead of our proposed scheme.

**Table 3:** System details for evaluation.

Resource	Operating System	Memory (MB)	Eucalyptus Component	Disk Size (GB)	CPU Architecture	CPU Cores	Network (Mb/s)
Machine 1 (Server)	Ubuntu 10.04	2048	Walrus	80	x84_64 Intel(R) Core (TM) 2	2 (2.33 GHz)	100
Machine 2 (Server)	CentOS 6.4	4192	Cluster, Node	250	x84_64 Intel(R) Core (TM)2	4 (2.83 GHz)	100
Machine 3 (Client)	Ubuntu 12.04	2048	Amazon SDK	80	x84_64 Intel(R) Core (TM) 2	2 (2.13 GHz)	100

### ProvCollector overhead

To evaluate the collection overhead, we executed a scenario where various objects of different size and formats (see S1 Dataset) from the client machine are uploaded to a Cloud with and without the support of provenance. We steadily increased the number of objects from 200 to 1000 and calculated the time required to upload each additional 200 objects. The calculated overhead is measured via elapsed time between the objects with/without the provenance, i.e.,

*Elapsed Time* = *Time taken by objects with the provenance minus the time taken by objects without the provenance*

**Fig 6:**
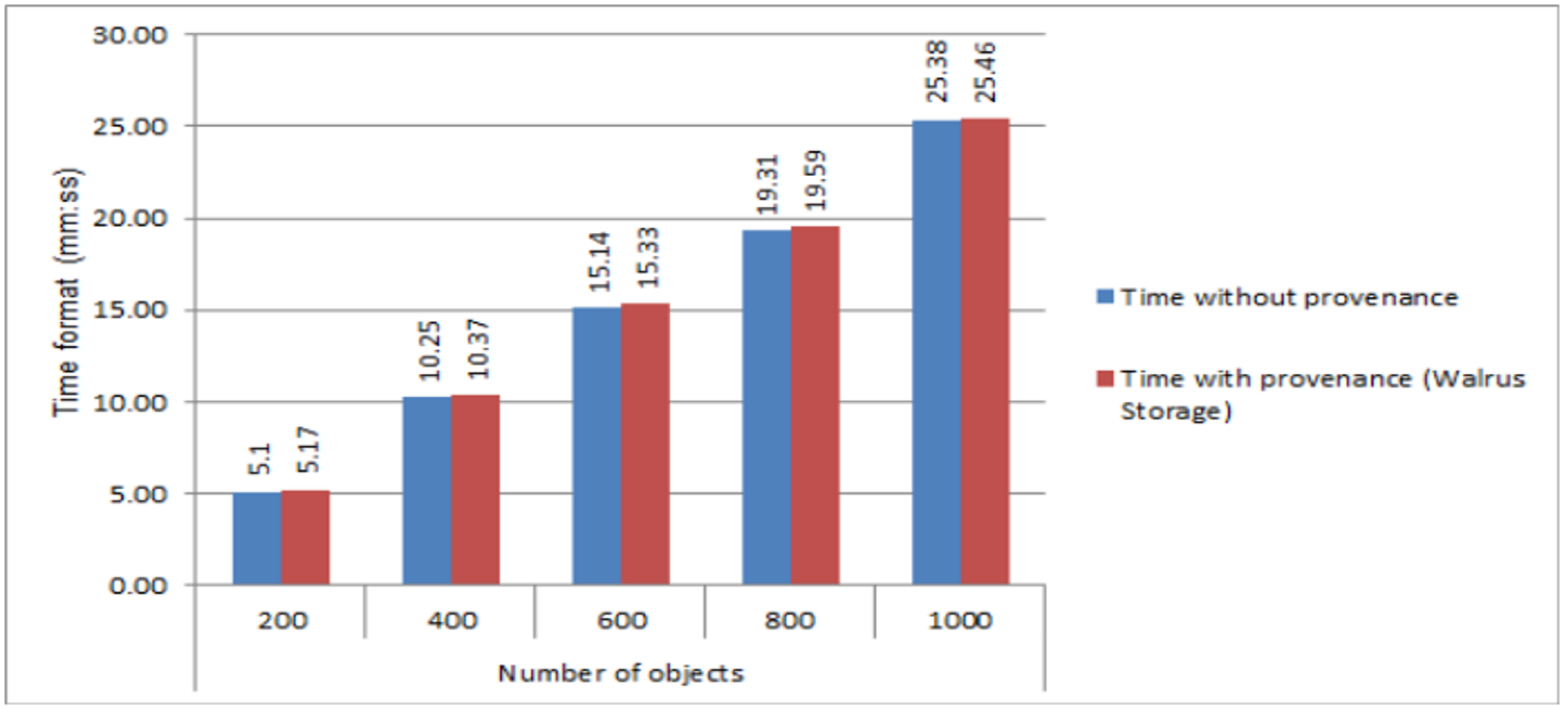
Results of the calculated time (minutes:seconds format) with and without the provenance for Eucalyptus Walrus.

The overhead is presented in Fig 6.

Elapsed time is calculated for different number of objects in Fig 6. For instance, when the numbers of objects are 200, elapsed time is **16 seconds**. Therefore the cost for individual objects is calculated using Eq 1.

$$Cost=\frac{ETn}{n}$$ (1)

where *n* represents the number of objects and *ET* represents the elapsed time.

**Fig 7:**
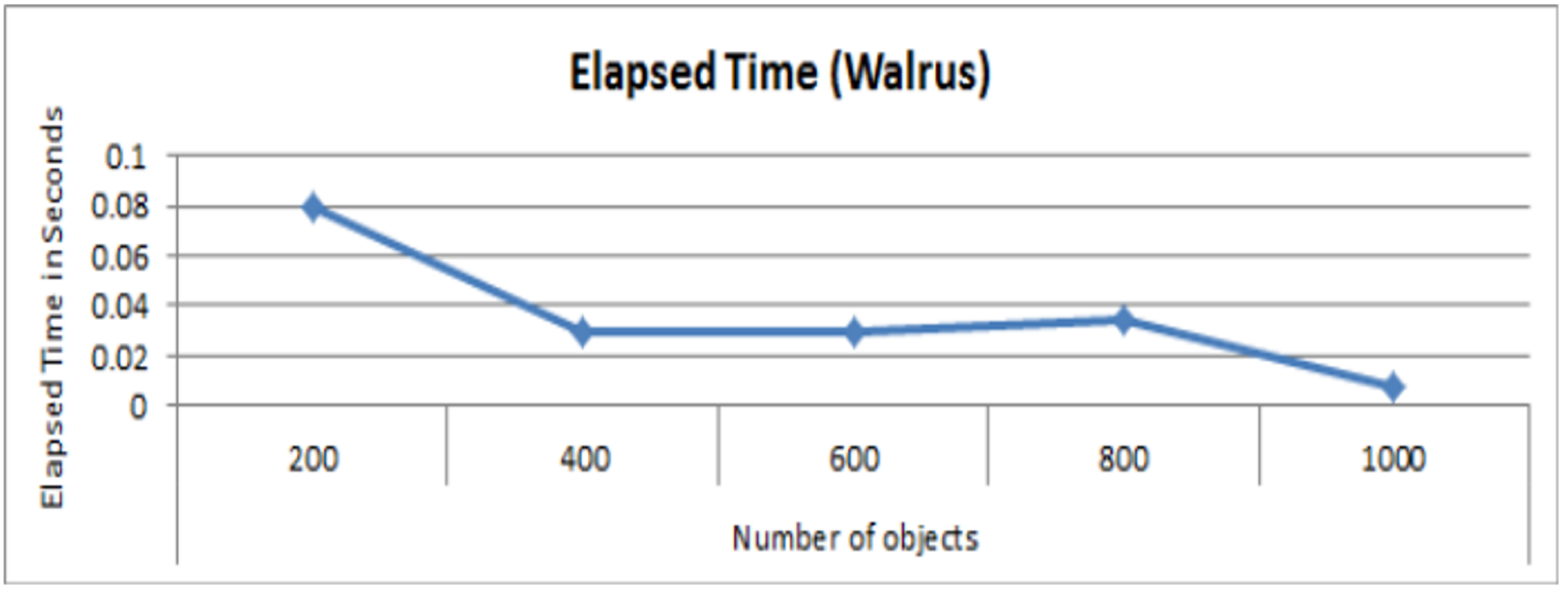
Cost of provenance collection in terms of elapsed time (in seconds) for different number of objects.

Fig 7 presents the elapsed time for different number of objects using Eq 1. The results in Fig 7 clearly show that the involved overhead is negligible when individual objects are uploaded to Cloud. Moreover, it also demonstrates, the cost for individual object remains almost the same regardless the number of objects as depicted by the trend lines.

### ProvStorage overhead

**Fig 8:**
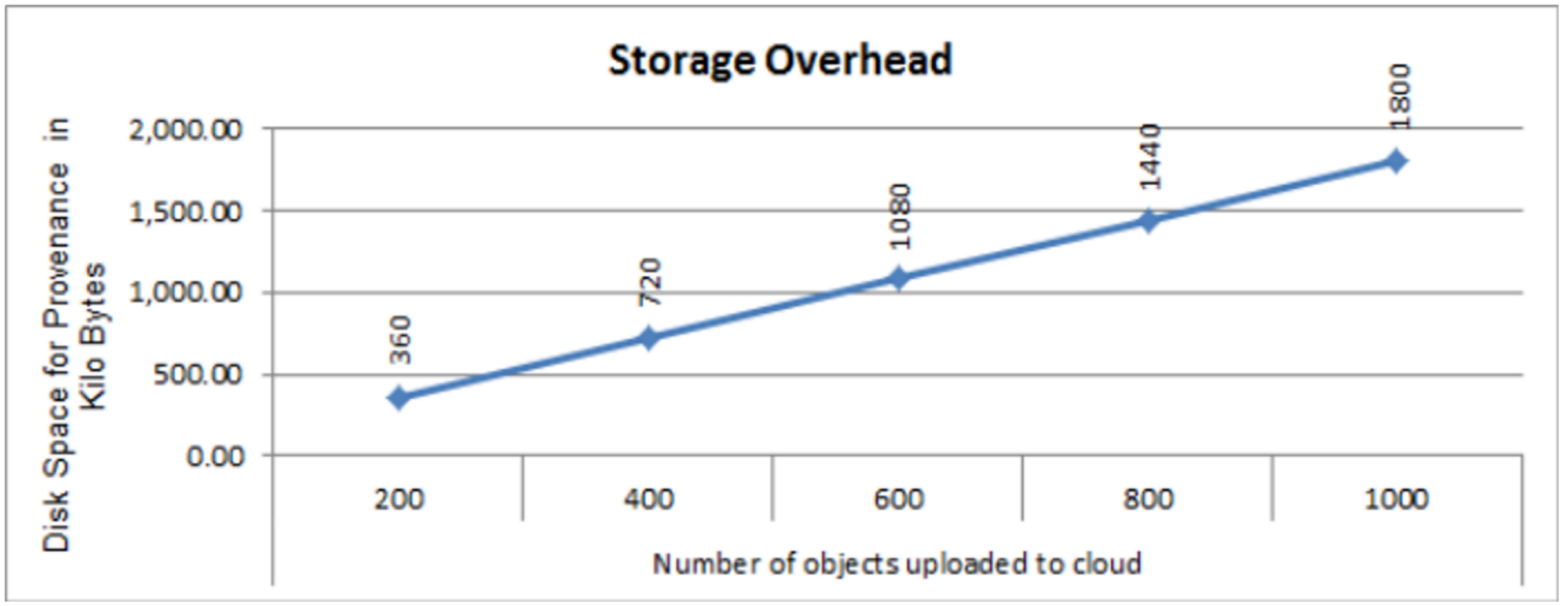
Cost of provenance storage (disk space in Kilo bytes) for different number of objects.

We store the provenance data in XML object where each individual provenance item costs approximately *1.8 KB* of disk space. The individual provenance item contains information from the client, middleware and server. Fig 8 presents the cost of provenance storage using the average size of 1.8 KB of disk space for XML repository. Fig 8 depicts a negligible cost of provenance storage which remains consistent regardless the size of the original objects uploaded to the Cloud. This is achieved using the link based approach and coarse grained provenance for collection and storage.

### Discussion

When considering performance of the proposed scheme we are interested in the computation and storage overhead. We realized that existing schemes utilize data replication or hashing techniques. The replication based scheme requires huge storage overhead because of data redundancy where other schemes require computation overhead because of key generating algorithms on file or block level. Our proposed scheme does not rely on such factors. We calculate coarse grained provenance which keeps the computation overhead minimal where as storing provenance using link based approach require minimal storage overhead [38, 39, 44].

The proposed scheme of integrity checking and verification presents the cost (almost negligible) in terms of provenance collection and storage overheads as shown by various experiments for *DataStore* application. It is observed that our scheme is not affected by the size or format of objects uploaded to Clouds. This is accomplished using coarse grained provenance information and link based mechanishm for storage.

## Comparative analysis of proposed scheme with existing schemes

Cloud utilizes different schemes for ensuring data integrity in single server and multiple server architecture settings [30–32]. Methods like Proof of Retrievability [31, 35] and Proof of Data Possession [30, 34] are utilized mainly in single server setting where as methods like High Availability Integrity Layer (HAIL) [32] and Multiple Replica Provable Data Possession (MR-PDP) [33] are mostly utilized in multiple server settings. Single server settings rely on methods such as key generation algorithms, cryptographic techniques and message authentication codes [34–37]. Multiple servers rely on striped data and redundancy techniques such as creating multiple replicas of original data [32, 33]. Third party auditors are also utilized in cloud model for the checking and verification of data integrity. Such auditors utilize one or more of the mentioned techniques for the verification of data integrity. Table 4 highlights the key integrity schemes used in clouds and their corresponding advantages and limitations.

**Table 4:** Comparison of existing integrity schemes in Cloud.

Data Integrity Scheme	Data Integrity Method	Advantages	Limitations
Provable Data possession [30, 34]	Key generation based on file comparison	Strong data integrity verification, Reduced network traffic	High computational cost on the server end for computing hash value of each file, Utilized mainly in single server settings, Depends on Client.
Proof of Retrievability [31, 35]	Cryptographic techniques such as sentinel based values	Less storage overhead on server side because storing only sentinel values	Computational overhead for pre-processing the sentinel value
HAIL [32]	Data redundancy across multiple servers using principles of RAID	The proof is independent of the size of data items e.g. files of different sizes.	Storage overhead because of replicating data items. Works only for static files. Thin client cannot adopt such a scheme
MR-PDP [33]	Multiple replicas using redundancy techniques	Provide the facility of on-demand replicas	Storage overhead because of replication. Computation required on both the client and server side
Third party auditor	Key generations and MAC based scheme utilized by third party	TPA can apply multiple integrity schemes for checking data integrity, Users can choose different TPA based on their preferences	Retrieval of data blocks from third party for checking data integrity (privacy issues), More cost due to involvement of third party auditors.

The major difference of the proposed scheme is the utilization of provenance data for integrity checks. The major advantage of the proposed scheme is negligible computation and storage overhead and its independence from third party auditors and clients. Table 5 highlights the differences and advantages of the proposed scheme when compared to the existing schemes in terms of dependency, computation cost, storage cost, and level of integrity checks amongst others.

**Table 5:** Comparative analysis of proposed scheme with existing data integrity schemes.

Evaluation Parameter	Proposed scheme	Provable data procession	Proof of Retrievability	HAIL	MR-PDP	Third party auditors
Dependency on third party	No	No	No	No	No	yes
Computation cost	Negligible because of coarse grained provenance [38–40]	High	High	Low	Low	Depends on the scheme
Storage cost	Negligible because of link based provenance [38–40]	Low to Medium	Low to Medium	High	High	Depends on the scheme
Basic Scheme	Utilizing historical data (provenance)	Key generation algorithms	Cryptog- raphic techniques	Data redundancy	Data replicas across multiple servers	Depends on the scheme
Integrity Checks	File level	File level	File level	File and block level	File and block level	Mainly on file level
Dependency on client	No	Yes	Yes	No	No	Yes

## Limitations

Provenance is an important and significant information that are used in wide variety of fields for checking quality and establishing trust [13]. Provenance data is generally categorized based on the granularity level of collected information into coarse-grained and fine-grained schemes in computational and data science [44]. The coarse-grained scheme is focused on storing a limited amount of significant information where fine-grained scheme stores a huge amount of data. Both the approaches have their pros and cons. In this work, we utilized the coarse-grained scheme because we are interested only in the significant information of different items managed through different services (SOA architecture).

The usage of coarse-grained scheme limits our solution of integrity proof for significant attributes collected at the client, middleware and service levels. For instance, if a word file with 200 words is modified maliciously with an additional word, our scheme can verify that the item is changed or corrupted using the details about time stamps and other information. However, we will not be able to find the exact word added to the original document. A fine-grained scheme like PASS [45] can be combined with our solution for creating a hybrid approach to solve such issues which is one of our future work directions.

## Conclusion

In this paper, we discussed the importance of data integrity especially in Cloud computing environments. We analyzed some existing techniques of data integrity assurance and checking in on-premise as well as in Cloud storage. We identified some issues in the existing approaches which makes them inefficient and economically not feasible in Cloud environments. Therefore, we have proposed a new scheme of data integrity proofs in Cloud environment to eliminate such issues and provide a highly flexible solution for the Cloud users. Our proposed scheme is based on the use of data provenance, which is a local resource of the Cloud environment. Provenance is basically the information (metadata) which describe the origin and the processing history of a data product. This metadata is utilized in our solution to track any integrity leaks throughout the data product life cycle in Cloud. In this research work, we investigated what methods should be applied on the recorded data provenance to achieve our expected results. We also presented a test bed scenario which implements the proposed scheme and generates integrity proofs. The successful execution of our scheme without the need of any additional hardware and TPA support clearly proves the utility of our solution in Cloud computing.

## Supporting information

S1 Dataset Files of different size and format. Files in the Dataset are utilized for performance measurement.
(ZIP)
