# Supplementary material for: Provenance based data integrity checking and verification in cloud environments
Source: PLoS One. 2017 May 17;12(5):e0177576. doi: 10.1371/journal.pone.0177576 (PMC5435237; doi:10.1371/journal.pone.0177576)
Supplement: S1 Dataset — Files in the Dataset are utilized for performance measurement. (ZIP) [file pone.0177576.s001.zip › dataset/doc/file3 - Copy (2).docx]

This is a text file ..this is a text file..text file

This is a text file ..this is a text file..text file

This is a text file ..this is a text file..text file

Dfsafdsafdsa fdsafdsa fdsafdsa

This is a text file ..this is a text file..text file

This is a text file ..this is a text file..text file

This is a text file ..this is a text file..text file

This is a text file ..this is a text file..text file

V

vv
