# Supplementary material for: Provenance based data integrity checking and verification in cloud environments
Source: PLoS One. 2017 May 17;12(5):e0177576. doi: 10.1371/journal.pone.0177576 (PMC5435237; doi:10.1371/journal.pone.0177576)
Supplement: S1 Dataset — Files in the Dataset are utilized for performance measurement. (ZIP) [file pone.0177576.s001.zip › dataset/doc/file5 - Copy (2).docx]

**Chapter 1**
**Introduction**
Nam dui ligula, fringilla a, euismod sodales, sollicitudin vel, wisi. Morbi auctor
lorem non justo. Nam lacus libero, pretium at, lobortis vitae, ultricies et, tellus.
Donec aliquet, tortor sed accumsan bibendum, erat ligula aliquet magna, vitae
ornare odio metus a mi. Morbi ac orci et nisl hendrerit mollis. Suspendisse ut
massa. Cras nec ante. Pellentesque a nulla. Cum sociis natoque penatibus et
magnis dis parturient montes, nascetur ridiculus mus. Aliquam tincidunt urna.
Nulla ullamcorper vestibulum turpis. Pellentesque cursus luctus mauris.
Nulla malesuada porttitor diam. Donec felis erat, congue non, volutpat at,
tincidunt tristique, libero. Vivamus viverra fermentum felis. Donec nonummy
pellentesque ante. Phasellus adipiscing semper elit. Proin fermentum massa
ac quam. Sed diam turpis, molestie vitae, placerat a, molestie nec, leo. Maecenas lacinia. Nam ipsum ligula, eleifend at, accumsan nec, suscipit a, ipsum.
Morbi blandit ligula feugiat magna. Nunc eleifend consequat lorem. Sed lacinia
nulla vitae enim. Pellentesque tincidunt purus vel magna. Integer non enim.
Praesent euismod nunc eu purus. Donec bibendum quam in tellus. Nullam cursus pulvinar lectus. Donec et mi. Nam vulputate metus eu enim. Vestibulum
pellentesque felis eu massa.
Quisque ullamcorper placerat ipsum. Cras nibh. Morbi vel justo vitae lacus
tincidunt ultrices. Lorem ipsum dolor sit amet, consectetuer adipiscing elit. In
hac habitasse platea dictumst. Integer tempus convallis augue. Etiam facilisis.
Nunc elementum fermentum wisi. Aenean placerat. Ut imperdiet, enim sed
gravida sollicitudin, felis odio placerat quam, ac pulvinar elit purus eget enim.
Nunc vitae tortor. Proin tempus nibh sit amet nisl. Vivamus quis tortor vitae
risus porta vehicula.
1

**Chapter 1**
**Introduction**
Nam dui ligula, fringilla a, euismod sodales, sollicitudin vel, wisi. Morbi auctor
lorem non justo. Nam lacus libero, pretium at, lobortis vitae, ultricies et, tellus.
Donec aliquet, tortor sed accumsan bibendum, erat ligula aliquet magna, vitae
ornare odio metus a mi. Morbi ac orci et nisl hendrerit mollis. Suspendisse ut
massa. Cras nec ante. Pellentesque a nulla. Cum sociis natoque penatibus et
magnis dis parturient montes, nascetur ridiculus mus. Aliquam tincidunt urna.
Nulla ullamcorper vestibulum turpis. Pellentesque cursus luctus mauris.
Nulla malesuada porttitor diam. Donec felis erat, congue non, volutpat at,
tincidunt tristique, libero. Vivamus viverra fermentum felis. Donec nonummy
pellentesque ante. Phasellus adipiscing semper elit. Proin fermentum massa
ac quam. Sed diam turpis, molestie vitae, placerat a, molestie nec, leo. Maecenas lacinia. Nam ipsum ligula, eleifend at, accumsan nec, suscipit a, ipsum.
Morbi blandit ligula feugiat magna. Nunc eleifend consequat lorem. Sed lacinia
nulla vitae enim. Pellentesque tincidunt purus vel magna. Integer non enim.
Praesent euismod nunc eu purus. Donec bibendum quam in tellus. Nullam cursus pulvinar lectus. Donec et mi. Nam vulputate metus eu enim. Vestibulum
pellentesque felis eu massa.
Quisque ullamcorper placerat ipsum. Cras nibh. Morbi vel justo vitae lacus
tincidunt ultrices. Lorem ipsum dolor sit amet, consectetuer adipiscing elit. In
hac habitasse platea dictumst. Integer tempus convallis augue. Etiam facilisis.
Nunc elementum fermentum wisi. Aenean placerat. Ut imperdiet, enim sed
gravida sollicitudin, felis odio placerat quam, ac pulvinar elit purus eget enim.
Nunc vitae tortor. Proin tempus nibh sit amet nisl. Vivamus quis tortor vitae
risus porta vehicula.
1

**Chapter 1**
**Introduction**
Nam dui ligula, fringilla a, euismod sodales, sollicitudin vel, wisi. Morbi auctor
lorem non justo. Nam lacus libero, pretium at, lobortis vitae, ultricies et, tellus.
Donec aliquet, tortor sed accumsan bibendum, erat ligula aliquet magna, vitae
ornare odio metus a mi. Morbi ac orci et nisl hendrerit mollis. Suspendisse ut
massa. Cras nec ante. Pellentesque a nulla. Cum sociis natoque penatibus et
magnis dis parturient montes, nascetur ridiculus mus. Aliquam tincidunt urna.
Nulla ullamcorper vestibulum turpis. Pellentesque cursus luctus mauris.
Nulla malesuada porttitor diam. Donec felis erat, congue non, volutpat at,
tincidunt tristique, libero. Vivamus viverra fermentum felis. Donec nonummy
pellentesque ante. Phasellus adipiscing semper elit. Proin fermentum massa
ac quam. Sed diam turpis, molestie vitae, placerat a, molestie nec, leo. Maecenas lacinia. Nam ipsum ligula, eleifend at, accumsan nec, suscipit a, ipsum.
Morbi blandit ligula feugiat magna. Nunc eleifend consequat lorem. Sed lacinia
nulla vitae enim. Pellentesque tincidunt purus vel magna. Integer non enim.
Praesent euismod nunc eu purus. Donec bibendum quam in tellus. Nullam cursus pulvinar lectus. Donec et mi. Nam vulputate metus eu enim. Vestibulum
pellentesque felis eu massa.
Quisque ullamcorper placerat ipsum. Cras nibh. Morbi vel justo vitae lacus
tincidunt ultrices. Lorem ipsum dolor sit amet, consectetuer adipiscing elit. In
hac habitasse platea dictumst. Integer tempus convallis augue. Etiam facilisis.
Nunc elementum fermentum wisi. Aenean placerat. Ut imperdiet, enim sed
gravida sollicitudin, felis odio placerat quam, ac pulvinar elit purus eget enim.
Nunc vitae tortor. Proin tempus nibh sit amet nisl. Vivamus quis tortor vitae
risus porta vehicula.
1This is a text file ..this is a text file..text file

This is a text file ..this is a text file..text file

This is a text file ..this is a text file..text file

This is a text file ..this is a text file..text file

This is a text file ..this is a text file..text file

This is a text file ..this is a text file..text file

This is a text file ..this is a text file..text file

V

vv
