# Supplementary material for: Provenance based data integrity checking and verification in cloud environments
Source: PLoS One. 2017 May 17;12(5):e0177576. doi: 10.1371/journal.pone.0177576 (PMC5435237; doi:10.1371/journal.pone.0177576)
Supplement: S1 Dataset — Files in the Dataset are utilized for performance measurement. (ZIP) [file pone.0177576.s001.zip › dataset/html/changes - Copy (2) - Copy.html]

Hadoop Change Log


# Hadoop Change Log

## Release 0.18.0 - Unreleased

- INCOMPATIBLE CHANGES
     (23)
  1. HADOOP-2703. The default options to fsck skips checking files
     that are being written to. The output of fsck is incompatible
     with previous release.  
     (lohit vijayarenu via dhruba)
  2. HADOOP-2865. FsShell.ls() printout format changed to print file names
     in the end of the line.  
     (Edward J. Yoon via shv)
  3. HADOOP-3283. The Datanode has a RPC server. It currently supports
     two RPCs: the first RPC retrives the metadata about a block and the
     second RPC sets the generation stamp of an existing block.
     (Tsz Wo (Nicholas), SZE via dhruba)
  4. HADOOP-2797. Code related to upgrading to 0.14 (Block CRCs) is
     removed. As result, upgrade to 0.18 or later from 0.13 or earlier
     is not supported. If upgrading from 0.13 or earlier is required,
     please upgrade to an intermediate version (0.14-0.17) and then
     to this version.  
     (rangadi)
  5. HADOOP-544. This issue introduces new classes JobID, TaskID and
     TaskAttemptID, which should be used instead of their string counterparts.
     Functions in JobClient, TaskReport, RunningJob, jobcontrol.Job and
     TaskCompletionEvent that use string arguments are deprecated in favor
     of the corresponding ones that use ID objects. Applications can use
     xxxID.toString() and xxxID.forName() methods to convert/restore objects
     to/from strings.  
     (Enis Soztutar via ddas)
  6. HADOOP-2188. RPC client sends a ping rather than throw timeouts.
     RPC server does not throw away old RPCs. If clients and the server are on
     different versions, they are not able to function well. In addition,
     The property ipc.client.timeout is removed from the default hadoop
     configuration. It also removes metrics RpcOpsDiscardedOPsNum.  
     (hairong)
  7. HADOOP-2181. This issue adds logging for input splits in Jobtracker log
     and jobHistory log. Also adds web UI for viewing input splits in job UI
     and history UI.  
     (Amareshwari Sriramadasu via ddas)
  8. HADOOP-3226. Run combiners multiple times over map outputs as they
     are merged in both the map and the reduce tasks.  
     (cdouglas via omalley)
  9. HADOOP-3329. DatanodeDescriptor objects should not be stored in the
     fsimage.  
     (dhruba)
  10. HADOOP-2656. The Block object has a generation stamp inside it.
      Existing blocks get a generation stamp of 0. This is needed to support
      appends.  
      (dhruba)
  11. HADOOP-3390. Removed deprecated ClientProtocol.abandonFileInProgress().
      (Tsz Wo (Nicholas), SZE via rangadi)
  12. HADOOP-3405. Made some map/reduce internal classes non-public:
      MapTaskStatus, ReduceTaskStatus, JobSubmissionProtocol,
      CompletedJobStatusStore.  
      (enis via omaley)
  13. HADOOP-3265. Removed depcrecated API getFileCacheHints().  
      (Lohit Vijayarenu via rangadi)
  14. HADOOP-3310. The namenode instructs the primary datanode to do lease
      recovery. The block gets a new generation stamp.
      (Tsz Wo (Nicholas), SZE via dhruba)
  15. HADOOP-2909. Improve IPC idle connection management. Property
      ipc.client.maxidletime is removed from the default configuration,
      instead it is defined as twice of the ipc.client.connection.maxidletime.
      A connection with outstanding requests won't be treated as idle.  
      (hairong)
  16. HADOOP-3459. Change in the output format of dfs -ls to more closely match
      /bin/ls. New format is: perm repl owner group size date name  
      (Mukund Madhugiri via omally)
  17. HADOOP-3113. An fsync invoked on a HDFS file really really
      persists data! The datanode moves blocks in the tmp directory to
      the real block directory on a datanode-restart.  
      (dhruba)
  18. HADOOP-3452. Change fsck to return non-zero status for a corrupt
      FileSystem.  
      (lohit vijayarenu via cdouglas)
  19. HADOOP-3193. Include the address of the client that found the corrupted
      block in the log. Also include a CorruptedBlocks metric to track the size
      of the corrupted block map.  
      (cdouglas)
  20. HADOOP-3512. Separate out the tools into a tools jar.  
      (omalley)
  21. HADOOP-3598. Ensure that temporary task-output directories are not created
      if they are not necessary e.g. for Maps with no side-effect files.  
      (acmurthy)
  22. HADOOP-3665. Modify WritableComparator so that it only creates instances
      of the keytype if the type does not define a WritableComparator. Calling
      the superclass compare will throw a NullPointerException. Also define
      a RawComparator for NullWritable and permit it to be written as a key
      to SequenceFiles.  
      (cdouglas)
  23. HADOOP-3673. Avoid deadlock caused by DataNode RPC receoverBlock().
      (Tsz Wo (Nicholas), SZE via rangadi)
- NEW FEATURES
     (25)
  1. HADOOP-3074. Provides a UrlStreamHandler for DFS and other FS,
     relying on FileSystem  
     (taton)
  2. HADOOP-2585. Name-node imports namespace data from a recent checkpoint
     accessible via a NFS mount.  
     (shv)
  3. HADOOP-3061. Writable types for doubles and bytes.  
     (Andrzej
     Bialecki via omalley)
  4. HADOOP-2857. Allow libhdfs to set jvm options.  
     (Craig Macdonald
     via omalley)
  5. HADOOP-3317. Add default port for HDFS namenode. The port in
     "hdfs:" URIs now defaults to 8020, so that one may simply use URIs
     of the form "hdfs://example.com/dir/file".
  6. HADOOP-2019. Adds support for .tar, .tgz and .tar.gz files in
     DistributedCache  
     (Amareshwari Sriramadasu via ddas)
  7. HADOOP-3058. Add FSNamesystem status metrics.  
     (Lohit Vjayarenu via rangadi)
  8. HADOOP-1915. Allow users to specify counters via strings instead
     of enumerations.  
     (tomwhite via omalley)
  9. HADOOP-2065. Delay invalidating corrupt replicas of block until its
     is removed from under replicated state. If all replicas are found to
     be corrupt, retain all copies and mark the block as corrupt.  
     (Lohit Vjayarenu via rangadi)
  10. HADOOP-3221. Adds org.apache.hadoop.mapred.lib.NLineInputFormat, which
      splits files into splits each of N lines. N can be specified by
      configuration property "mapred.line.input.format.linespermap", which
      defaults to 1.  
      (Amareshwari Sriramadasu via ddas)
  11. HADOOP-3336. Direct a subset of annotated FSNamesystem calls for audit
      logging.  
      (cdouglas)
  12. HADOOP-3400. A new API FileSystem.deleteOnExit() that facilitates
      handling of temporary files in HDFS.  
      (dhruba)
  13. HADOOP-4. Add fuse-dfs to contrib, permitting one to mount an
      HDFS filesystem on systems that support FUSE, e.g., Linux.  
      (Pete Wyckoff via cutting)
  14. HADOOP-3246. Add FTPFileSystem.  
      (Ankur Goel via cutting)
  15. HADOOP-3250. Extend FileSystem API to allow appending to files.
      (Tsz Wo (Nicholas), SZE via cdouglas)
  16. HADOOP-3177. Implement Syncable interface for FileSystem.
      (Tsz Wo (Nicholas), SZE via dhruba)
  17. HADOOP-1328. Implement user counters in streaming.  
      (tomwhite via
      omalley)
  18. HADOOP-3187. Quotas for namespace management.  
      (Hairong Kuang via ddas)
  19. HADOOP-3307. Support for Archives in Hadoop.  
      (Mahadev Konar via ddas)
  20. HADOOP-3460. Add SequenceFileAsBinaryOutputFormat to permit direct
      writes of serialized data.  
      (Koji Noguchi via cdouglas)
  21. HADOOP-3230. Add ability to get counter values from command
      line.  
      (tomwhite via omalley)
  22. HADOOP-930. Add support for native S3 files.  
      (tomwhite via cutting)
  23. HADOOP-3502. Quota API needs documentation in Forrest.  
      (hairong)
  24. HADOOP-3413. Allow SequenceFile.Reader to use serialization
      framework.  
      (tomwhite via omalley)
  25. HADOOP-3541. Import of the namespace from a checkpoint documented
      in hadoop user guide.  
      (shv)
- IMPROVEMENTS
     (47)
  1. HADOOP-3677. Simplify generation stamp upgrade by making is a
     local upgrade on datandodes. Deleted distributed upgrade.  
     (rangadi)
  2. HADOOP-2928. Remove deprecated FileSystem.getContentLength().  
     (Lohit Vjayarenu via rangadi)
  3. HADOOP-3130. Make the connect timeout smaller for getFile.  
     (Amar Ramesh Kamat via ddas)
  4. HADOOP-3160. Remove deprecated exists() from ClientProtocol and
     FSNamesystem  
     (Lohit Vjayarenu via rangadi)
  5. HADOOP-2910. Throttle IPC Clients during bursts of requests or
     server slowdown. Clients retry connection for up to 15 minutes
     when socket connection times out.  
     (hairong)
  6. HADOOP-3295. Allow TextOutputFormat to use configurable spearators.
     (Zheng Shao via cdouglas).
  7. HADOOP-3308. Improve QuickSort by excluding values eq the pivot from the
     partition.  
     (cdouglas)
  8. HADOOP-2461. Trim property names in configuration.
     (Tsz Wo (Nicholas), SZE via shv)
  9. HADOOP-2799. Deprecate o.a.h.io.Closable in favor of java.io.Closable.
     (Tsz Wo (Nicholas), SZE via cdouglas)
  10. HADOOP-3345. Enhance the hudson-test-patch target to cleanup messages,
      fix minor defects, and add eclipse plugin and python unit tests.  
      (nigel)
  11. HADOOP-3144. Improve robustness of LineRecordReader by defining a maximum
      line length (mapred.linerecordreader.maxlength), thereby avoiding reading
      too far into the following split.  
      (Zheng Shao via cdouglas)
  12. HADOOP-3334. Move lease handling from FSNamesystem into a seperate class.
      (Tsz Wo (Nicholas), SZE via rangadi)
  13. HADOOP-3332. Reduces the amount of logging in Reducer's shuffle phase.  
      (Devaraj Das)
  14. HADOOP-3355. Enhances Configuration class to accept hex numbers for getInt
      and getLong.  
      (Amareshwari Sriramadasu via ddas)
  15. HADOOP-3350. Add an argument to distcp to permit the user to limit the
      number of maps.  
      (cdouglas)
  16. HADOOP-3013. Add corrupt block reporting to fsck.  
      (lohit vijayarenu via cdouglas)
  17. HADOOP-3377. Remove TaskRunner::replaceAll and replace with equivalent
      String::replace.  
      (Brice Arnould via cdouglas)
  18. HADOOP-3398. Minor improvement to a utility function in that participates
      in backoff calculation.  
      (cdouglas)
  19. HADOOP-3381. Clear referenced when directories are deleted so that
      effect of memory leaks are not multiplied.  
      (rangadi)
  20. HADOOP-2867. Adds the task's CWD to its LD\_LIBRARY\_PATH.  
      (Amareshwari Sriramadasu via ddas)
  21. HADOOP-3232. DU class runs the 'du' command in a seperate thread so
      that it does not block user. DataNode misses heartbeats in large
      nodes otherwise.  
      (Johan Oskarsson via rangadi)
  22. HADOOP-3035. During block transfers between datanodes, the receiving
      datanode, now can report corrupt replicas received from src node to
      the namenode.  
      (Lohit Vijayarenu via rangadi)
  23. HADOOP-3434. Retain the cause of the bind failure in Server::bind.  
      (Steve Loughran via cdouglas)
  24. HADOOP-3429. Increases the size of the buffers used for the communication
      for Streaming jobs.  
      (Amareshwari Sriramadasu via ddas)
  25. HADOOP-3486. Change default for initial block report to 0 seconds
      and document it.  
      (Sanjay Radia via omalley)
  26. HADOOP-3448. Improve the text in the assertion making sure the
      layout versions are consistent in the data node.  
      (Steve Loughran
      via omalley)
  27. HADOOP-2095. Improve the Map-Reduce shuffle/merge by cutting down
      buffer-copies; changed intermediate sort/merge to use the new IFile format
      rather than SequenceFiles and compression of map-outputs is now
      implemented by compressing the entire file rather than SequenceFile
      compression. Shuffle also has been changed to use a simple byte-buffer
      manager rather than the InMemoryFileSystem.
      Configuration changes to hadoop-default.xml:
      deprecated mapred.map.output.compression.type  
      (acmurthy)
  28. HADOOP-236. JobTacker now refuses connection from a task tracker with a
      different version number.  
      (Sharad Agarwal via ddas)
  29. HADOOP-3427. Improves the shuffle scheduler. It now waits for notifications
      from shuffle threads when it has scheduled enough, before scheduling more.  
      (ddas)
  30. HADOOP-2393. Moves the handling of dir deletions in the tasktracker to
      a separate thread.  
      (Amareshwari Sriramadasu via ddas)
  31. HADOOP-3501. Deprecate InMemoryFileSystem.  
      (cutting via omalley)
  32. HADOOP-3366. Stall the shuffle while in-memory merge is in progress.  
      (acmurthy)
  33. HADOOP-2916. Refactor src structure, but leave package structure alone.  
      (Raghu Angadi via mukund)
  34. HADOOP-3492. Add forrest documentation for user archives.  
      (Mahadev Konar via hairong)
  35. HADOOP-3467. Improve documentation for FileSystem::deleteOnExit.
      (Tsz Wo (Nicholas), SZE via cdouglas)
  36. HADOOP-3379. Documents stream.non.zero.exit.status.is.failure for Streaming.  
      (Amareshwari Sriramadasu via ddas)
  37. HADOOP-3096. Improves documentation about the Task Execution Environment in
      the Map-Reduce tutorial.  
      (Amareshwari Sriramadasu via ddas)
  38. HADOOP-2984. Add forrest documentation for DistCp  
      (cdouglas)
  39. HADOOP-3406. Add forrest documentation for Profiling.  
      (Amareshwari Sriramadasu via ddas)
  40. HADOOP-2762. Add forrest documentation for controls of memory limits on
      hadoop daemons and Map-Reduce tasks.  
      (Amareshwari Sriramadasu via ddas)
  41. HADOOP-3535. Fix documentation and name of IOUtils.close to
      reflect that it should only be used in cleanup contexts.  
      (omalley)
  42. HADOOP-3593. Updates the mapred tutorial.  
      (ddas)
  43. HADOOP-3547. Documents the way in which native libraries can be distributed
      via the DistributedCache.  
      (Amareshwari Sriramadasu via ddas)
  44. HADOOP-3606. Updates the Streaming doc.  
      (Amareshwari Sriramadasu via ddas)
  45. HADOOP-3532. Add jdiff reports to the build scripts.  
      (omalley)
  46. HADOOP-3100. Develop tests to test the DFS command line interface.  
      (mukund)
  47. HADOOP-3688. Fix up HDFS docs.  
      (Robert Chansler via hairong)
- OPTIMIZATIONS
     (10)
  1. HADOOP-3274. The default constructor of BytesWritable creates empty
     byte array. (Tsz Wo (Nicholas), SZE via shv)
  2. HADOOP-3272. Remove redundant copy of Block object in BlocksMap.  
     (Lohit Vjayarenu via shv)
  3. HADOOP-3164. Reduce DataNode CPU usage by using FileChannel.tranferTo().
     On Linux DataNode takes 5 times less CPU while serving data. Results may
     vary on other platforms.  
     (rangadi)
  4. HADOOP-3248. Optimization of saveFSImage.  
     (Dhruba via shv)
  5. HADOOP-3297. Fetch more task completion events from the job
     tracker and task tracker.  
     (ddas via omalley)
  6. HADOOP-3364. Faster image and log edits loading.  
     (shv)
  7. HADOOP-3369. Fast block processing during name-node startup.  
     (shv)
  8. HADOOP-1702. Reduce buffer copies when data is written to DFS.
     DataNodes take 30% less CPU while writing data.  
     (rangadi)
  9. HADOOP-3095. Speed up split generation in the FileInputSplit,
     especially for non-HDFS file systems. Deprecates
     InputFormat.validateInput.  
     (tomwhite via omalley)
  10. HADOOP-3552. Add forrest documentation for Hadoop commands.  
      (Sharad Agarwal via cdouglas)
- BUG FIXES
     (138)
  1. HADOOP-2905. 'fsck -move' triggers NPE in NameNode.  
     (Lohit Vjayarenu via rangadi)
  2. Increment ClientProtocol.versionID missed by HADOOP-2585.  
     (shv)
  3. HADOOP-3254. Restructure internal namenode methods that process
     heartbeats to use well-defined BlockCommand object(s) instead of
     using the base java Object. (Tsz Wo (Nicholas), SZE via dhruba)
  4. HADOOP-3176. Change lease record when a open-for-write-file
     gets renamed.  
     (dhruba)
  5. HADOOP-3269. Fix a case when namenode fails to restart
     while processing a lease record. ((Tsz Wo (Nicholas), SZE via dhruba)
  6. HADOOP-3282. Port issues in TestCheckpoint resolved.  
     (shv)
  7. HADOOP-3268. file:// URLs issue in TestUrlStreamHandler under Windows.  
     (taton)
  8. HADOOP-3127. Deleting files in trash should really remove them.  
     (Brice Arnould via omalley)
  9. HADOOP-3300. Fix locking of explicit locks in NetworkTopology.  
     (tomwhite via omalley)
  10. HADOOP-3270. Constant DatanodeCommands are stored in static final
      immutable variables for better code clarity.
      (Tsz Wo (Nicholas), SZE via dhruba)
  11. HADOOP-2793. Fix broken links for worst performing shuffle tasks in
      the job history page.  
      (Amareshwari Sriramadasu via ddas)
  12. HADOOP-3313. Avoid unnecessary calls to System.currentTimeMillis
      in RPC::Invoker.  
      (cdouglas)
  13. HADOOP-3318. Recognize "Darwin" as an alias for "Mac OS X" to
      support Soylatte.  
      (Sam Pullara via omalley)
  14. HADOOP-3301. Fix misleading error message when S3 URI hostname
      contains an underscore.  
      (tomwhite via omalley)
  15. HADOOP-3338. Fix Eclipse plugin to compile after HADOOP-544 was
      committed. Updated all references to use the new JobID representation.  
      (taton via nigel)
  16. HADOOP-3337. Loading FSEditLog was broken by HADOOP-3283 since it
      changed Writable serialization of DatanodeInfo. This patch handles it.
      (Tsz Wo (Nicholas), SZE via rangadi)
  17. HADOOP-3101. Prevent JobClient from throwing an exception when printing
      usage.  
      (Edward J. Yoon via cdouglas)
  18. HADOOP-3119. Update javadoc for Text::getBytes to better describe its
      behavior.  
      (Tim Nelson via cdouglas)
  19. HADOOP-2294. Fix documentation in libhdfs to refer to the correct free
      function.  
      (Craig Macdonald via cdouglas)
  20. HADOOP-3335. Prevent the libhdfs build from deleting the wrong
      files on make clean.  
      (cutting via omalley)
  21. HADOOP-2930. Make {start,stop}-balancer.sh work even if hadoop-daemon.sh
      is not in the PATH.  
      (Spiros Papadimitriou via hairong)
  22. HADOOP-3085. Catch Exception in metrics util classes to ensure that
      misconfigured metrics don't prevent others from updating.  
      (cdouglas)
  23. HADOOP-3299. CompositeInputFormat should configure the sub-input
      formats.  
      (cdouglas via omalley)
  24. HADOOP-3309. Lower io.sort.mb and fs.inmemory.size.mb for MiniMRDFSSort
      unit test so it passes on Windows.  
      (lohit vijayarenu via cdouglas)
  25. HADOOP-3348. TestUrlStreamHandler should set URLStreamFactory after
      DataNodes are initialized.  
      (Lohit Vijayarenu via rangadi)
  26. HADOOP-3371. Ignore InstanceAlreadyExistsException from
      MBeanUtil::registerMBean.  
      (lohit vijayarenu via cdouglas)
  27. HADOOP-3349. A file rename was incorrectly changing the name inside a
      lease record. (Tsz Wo (Nicholas), SZE via dhruba)
  28. HADOOP-3365. Removes an unnecessary copy of the key from SegmentDescriptor
      to MergeQueue.  
      (Devaraj Das)
  29. HADOOP-3388. Fix for TestDatanodeBlockScanner to handle blocks with
      generation stamps in them.  
      (dhruba)
  30. HADOOP-3203. Fixes TaskTracker::localizeJob to pass correct file sizes
      for the jarfile and the jobfile.  
      (Amareshwari Sriramadasu via ddas)
  31. HADOOP-3391. Fix a findbugs warning introduced by HADOOP-3248  
      (rangadi)
  32. HADOOP-3393. Fix datanode shutdown to call DataBlockScanner::shutdown and
      close its log, even if the scanner thread is not running.  
      (lohit vijayarenu
      via cdouglas)
  33. HADOOP-3399. A debug message was logged at info level.  
      (rangadi)
  34. HADOOP-3396. TestDatanodeBlockScanner occationally fails.  
      (Lohit Vijayarenu via rangadi)
  35. HADOOP-3339. Some of the failures on 3rd datanode in DFS write pipelie
      are not detected properly. This could lead to hard failure of client's
      write operation.  
      (rangadi)
  36. HADOOP-3409. Namenode should save the root inode into fsimage.  
      (hairong)
  37. HADOOP-3296. Fix task cache to work for more than two levels in the cache
      hierarchy. This also adds a new counter to track cache hits at levels
      greater than two.  
      (Amar Kamat via cdouglas)
  38. HADOOP-3375. Lease paths were sometimes not removed from
      LeaseManager.sortedLeasesByPath. (Tsz Wo (Nicholas), SZE via dhruba)
  39. HADOOP-3424. Values returned by getPartition should be checked to
      make sure they are in the range 0 to #reduces - 1  
      (cdouglas via
      omalley)
  40. HADOOP-3408. Change FSNamesystem to send its metrics as integers to
      accommodate collectors that don't support long values.  
      (lohit vijayarenu
      via cdouglas)
  41. HADOOP-3403. Fixes a problem in the JobTracker to do with handling of lost
      tasktrackers.  
      (Arun Murthy via ddas)
  42. HADOOP-1318. Completed maps are not failed if the number of reducers are
      zero. (Amareshwari Sriramadasu via ddas).
  43. HADOOP-3351. Fixes the history viewer tool to not do huge StringBuffer
      allocations.  
      (Amareshwari Sriramadasu via ddas)
  44. HADOOP-3419. Fixes TestFsck to wait for updates to happen before
      checking results to make the test more reliable.  
      (Lohit Vijaya
      Renu via omalley)
  45. HADOOP-3259. Makes failure to read system properties due to a
      security manager non-fatal.  
      (Edward Yoon via omalley)
  46. HADOOP-3451. Update libhdfs to use FileSystem::getFileBlockLocations
      instead of removed getFileCacheHints.  
      (lohit vijayarenu via cdouglas)
  47. HADOOP-3401. Update FileBench to set the new
      "mapred.work.output.dir" property to work post-3041.  
      (cdouglas via omalley)
  48. HADOOP-2669. DFSClient locks pendingCreates appropriately.  
      (dhruba)
  49. HADOOP-3410. Fix KFS implemenation to return correct file
      modification time.  
      (Sriram Rao via cutting)
  50. HADOOP-3340. Fix DFS metrics for BlocksReplicated, HeartbeatsNum, and
      BlockReportsAverageTime.  
      (lohit vijayarenu via cdouglas)
  51. HADOOP-3435. Remove the assuption in the scripts that bash is at
      /bin/bash and fix the test patch to require bash instead of sh.  
      (Brice Arnould via omalley)
  52. HADOOP-3471. Fix spurious errors from TestIndexedSort and add additional
      logging to let failures be reproducible.  
      (cdouglas)
  53. HADOOP-3443. Avoid copying map output across partitions when renaming a
      single spill.  
      (omalley via cdouglas)
  54. HADOOP-3454. Fix Text::find to search only valid byte ranges.  
      (Chad Whipkey
      via cdouglas)
  55. HADOOP-3417. Removes the static configuration variable,
      commandLineConfig from JobClient. Moves the cli parsing from
      JobShell to GenericOptionsParser. Thus removes the class
      org.apache.hadoop.mapred.JobShell.  
      (Amareshwari Sriramadasu via
      ddas)
  56. HADOOP-2132. Only RUNNING/PREP jobs can be killed.  
      (Jothi Padmanabhan
      via ddas)
  57. HADOOP-3476. Code cleanup in fuse-dfs.  
      (Peter Wyckoff via dhruba)
  58. HADOOP-2427. Ensure that the cwd of completed tasks is cleaned-up
      correctly on task-completion.  
      (Amareshwari Sri Ramadasu via acmurthy)
  59. HADOOP-2565. Remove DFSPath cache of FileStatus.
      (Tsz Wo (Nicholas), SZE via hairong)
  60. HADOOP-3326. Cleanup the local-fs and in-memory merge in the ReduceTask by
      spawing only one thread each for the on-disk and in-memory merge.  
      (Sharad Agarwal via acmurthy)
  61. HADOOP-3493. Fix TestStreamingFailure to use FileUtil.fullyDelete to
      ensure correct cleanup.  
      (Lohit Vijayarenu via acmurthy)
  62. HADOOP-3455. Fix NPE in ipc.Client in case of connection failure and
      improve its synchronization.  
      (hairong)
  63. HADOOP-3240. Fix a testcase to not create files in the current directory.
      Instead the file is created in the test directory  
      (Mahadev Konar via ddas)
  64. HADOOP-3496. Fix failure in TestHarFileSystem.testArchives due to change
      in HADOOP-3095.  
      (tomwhite)
  65. HADOOP-3135. Get the system directory from the JobTracker instead of from
      the conf.  
      (Subramaniam Krishnan via ddas)
  66. HADOOP-3503. Fix a race condition when client and namenode start
      simultaneous recovery of the same block. (dhruba & Tsz Wo
      (Nicholas), SZE)
  67. HADOOP-3440. Fixes DistributedCache to not create symlinks for paths which
      don't have fragments even when createSymLink is true.  
      (Abhijit Bagri via ddas)
  68. HADOOP-3463. Hadoop-daemons script should cd to $HADOOP\_HOME.  
      (omalley)
  69. HADOOP-3489. Fix NPE in SafeModeMonitor.  
      (Lohit Vijayarenu via shv)
  70. HADOOP-3509. Fix NPE in FSNamesystem.close. (Tsz Wo (Nicholas), SZE via
      shv)
  71. HADOOP-3491. Name-node shutdown causes InterruptedException in
      ResolutionMonitor.  
      (Lohit Vijayarenu via shv)
  72. HADOOP-3511. Fixes namenode image to not set the root's quota to an
      invalid value when the quota was not saved in the image.  
      (hairong)
  73. HADOOP-3516. Ensure the JobClient in HadoopArchives is initialized
      with a configuration.  
      (Subramaniam Krishnan via omalley)
  74. HADOOP-3513. Improve NNThroughputBenchmark log messages.  
      (shv)
  75. HADOOP-3519. Fix NPE in DFS FileSystem rename.  
      (hairong via tomwhite)
  76. HADOOP-3528. Metrics FilesCreated and files\_deleted metrics
      do not match.  
      (Lohit via Mahadev)
  77. HADOOP-3418. When a directory is deleted, any leases that point to files
      in the subdirectory are removed. ((Tsz Wo (Nicholas), SZE via dhruba)
  78. HADOOP-3542. Diables the creation of \_logs directory for the archives
      directory.  
      (Mahadev Konar via ddas)
  79. HADOOP-3544. Fixes a documentation issue for hadoop archives.  
      (Mahadev Konar via ddas)
  80. HADOOP-3517. Fixes a problem in the reducer due to which the last InMemory
      merge may be missed.  
      (Arun Murthy via ddas)
  81. HADOOP-3548. Fixes build.xml to copy all \*.jar files to the dist.  
      (Owen O'Malley via ddas)
  82. HADOOP-3363. Fix unformatted storage detection in FSImage.  
      (shv)
  83. HADOOP-3560. Fixes a problem to do with split creation in archives.  
      (Mahadev Konar via ddas)
  84. HADOOP-3545. Fixes a overflow problem in archives.  
      (Mahadev Konar via ddas)
  85. HADOOP-3561. Prevent the trash from deleting its parent directories.  
      (cdouglas)
  86. HADOOP-3575. Fix the clover ant target after package refactoring.  
      (Nigel Daley via cdouglas)
  87. HADOOP-3539. Fix the tool path in the bin/hadoop script under
      cygwin. (Tsz Wo (Nicholas), Sze via omalley)
  88. HADOOP-3520. TestDFSUpgradeFromImage triggers a race condition in the
      Upgrade Manager. Fixed.  
      (dhruba)
  89. HADOOP-3586. Provide deprecated, backwards compatibile semantics for the
      combiner to be run once and only once on each record.  
      (cdouglas)
  90. HADOOP-3533. Add deprecated methods to provide API compatibility
      between 0.18 and 0.17. Remove the deprecated methods in trunk.  
      (omalley)
  91. HADOOP-3580. Fixes a problem to do with specifying a har as an input to
      a job.  
      (Mahadev Konar via ddas)
  92. HADOOP-3333. Don't assign a task to a tasktracker that it failed to
      execute earlier (used to happen in the case of lost tasktrackers where
      the tasktracker would reinitialize and bind to a different port).  
      (Jothi Padmanabhan and Arun Murthy via ddas)
  93. HADOOP-3534. Log IOExceptions that happen in closing the name
      system when the NameNode shuts down. (Tsz Wo (Nicholas) Sze via omalley)
  94. HADOOP-3546. TaskTracker re-initialization gets stuck in cleaning up.  
      (Amareshwari Sriramadasu via ddas)
  95. HADOOP-3576. Fix NullPointerException when renaming a directory
      to its subdirectory. (Tse Wo (Nicholas), SZE via hairong)
  96. HADOOP-3320. Fix NullPointerException in NetworkTopology.getDistance().  
      (hairong)
  97. HADOOP-3569. KFS input stream read() now correctly reads 1 byte
      instead of 4.  
      (Sriram Rao via omalley)
  98. HADOOP-3599. Fix JobConf::setCombineOnceOnly to modify the instance rather
      than a parameter.  
      (Owen O'Malley via cdouglas)
  99. HADOOP-3590. Null pointer exception in JobTracker when the task tracker is
      not yet resolved.  
      (Amar Ramesh Kamat via ddas)
  100. HADOOP-3603. Fix MapOutputCollector to spill when io.sort.spill.percent is
       1.0 and to detect spills when emitted records write no data.  
       (cdouglas)
  101. HADOOP-3615. Set DatanodeProtocol.versionID to the correct value.
       (Tsz Wo (Nicholas), SZE via cdouglas)
  102. HADOOP-3559. Fix the libhdfs test script and config to work with the
       current semantics.  
       (lohit vijayarenu via cdouglas)
  103. HADOOP-3480. Need to update Eclipse template to reflect current trunk.  
       (Brice Arnould via tomwhite)
  104. HADOOP-3588. Fix usability bugs for archives.  
       (mahadev)
  105. HADOOP-3635. Uncaught exception in DataBlockScanner.
       (Tsz Wo (Nicholas), SZE via hairong)
  106. HADOOP-3539. Exception when closing DFSClient while multiple files are
       open.  
       (Benjamin Gufler via hairong)
  107. HADOOP-3572. SetQuotas usage interface has some minor bugs.  
       (hairong)
  108. HADOOP-3649. Fix bug in removing blocks from the corrupted block map.  
       (Lohit Vijayarenu via shv)
  109. HADOOP-3604. Work around a JVM synchronization problem observed while
       retrieving the address of direct buffers from compression code by obtaining
       a lock during this call.  
       (Arun C Murthy via cdouglas)
  110. HADOOP-3683. Fix dfs metrics to count file listings rather than files
       listed.  
       (lohit vijayarenu via cdouglas)
  111. HADOOP-3597. Fix SortValidator to use filesystems other than the default as
       input. Validation job still runs on default fs.  
       (Jothi Padmanabhan via cdouglas)
  112. HADOOP-3693. Fix archives, distcp and native library documentation to
       conform to style guidelines.  
       (Amareshwari Sriramadasu via cdouglas)
  113. HADOOP-3653. Fix test-patch target to properly account for Eclipse
       classpath jars.  
       (Brice Arnould via nigel)
  114. HADOOP-3692. Fix documentation for Cluster setup and Quick start guides.  
       (Amareshwari Sriramadasu via ddas)
  115. HADOOP-3691. Fix streaming and tutorial docs.  
       (Jothi Padmanabhan via ddas)
  116. HADOOP-3630. Fix NullPointerException in CompositeRecordReader from empty
       sources  
       (cdouglas)
  117. HADOOP-3706. Fix a ClassLoader issue in the mapred.join Parser that
       prevents it from loading user-specified InputFormats.  
       (Jingkei Ly via cdouglas)
  118. HADOOP-3718. Fix KFSOutputStream::write(int) to output a byte instead of
       an int, per the OutputStream contract.  
       (Sriram Rao via cdouglas)
  119. HADOOP-3647. Add debug logs to help track down a very occassional,
       hard-to-reproduce, bug in shuffle/merge on the reducer.  
       (acmurthy)
  120. HADOOP-3716. Prevent listStatus in KosmosFileSystem from returning
       null for valid, empty directories.  
       (Sriram Rao via cdouglas)
  121. HADOOP-3752. Fix audit logging to record rename events.  
       (cdouglas)
  122. HADOOP-3737. Fix CompressedWritable to call Deflater::end to release
       compressor memory.  
       (Grant Glouser via cdouglas)
  123. HADOOP-3670. Fixes JobTracker to clear out split bytes when no longer
       required.  
       (Amareshwari Sriramadasu via ddas)
  124. HADOOP-3743. Fix -libjars, -files, -archives options to work even if
       user code does not implement tools.  
       (Amareshwari Sriramadasu via mahadev)
  125. HADOOP-3774. Fix typos in shell output. (Tsz Wo (Nicholas), SZE via
       cdouglas)
  126. HADOOP-3762. Fixed FileSystem cache to work with the default port.  
       (cutting
       via omalley)
  127. HADOOP-3798. Fix tests compilation.  
       (Mukund Madhugiri via omalley)
  128. HADOOP-3794. Return modification time instead of zero for KosmosFileSystem.  
       (Sriram Rao via cdouglas)
  129. HADOOP-3806. Remove debug statement to stdout from QuickSort.  
       (cdouglas)
  130. HADOOP-3776. Fix NPE at NameNode when datanode reports a block after it is
       deleted at NameNode.  
       (rangadi)
  131. HADOOP-3537. Disallow adding a datanode to a network topology when its
       network location is not resolved.  
       (hairong)
  132. HADOOP-3571. Fix bug in block removal used in lease recovery.  
       (shv)
  133. HADOOP-3645. MetricsTimeVaryingRate returns wrong value for
       metric\_avg\_time.  
       (Lohit Vijayarenu via hairong)
  134. HADOOP-3521. Reverted the missing cast to float for sending Counters' values
       to Hadoop metrics which was removed by HADOOP-544.  
       (acmurthy)
  135. HADOOP-3724. Fixes two problems related to storing and recovering lease
       in the fsimage.  
       (dhruba)
  136. HADOOP-3827. Fixed compression of empty map-outputs.  
       (acmurthy)
  137. HADOOP-3855. Fixes an import problem introduced by HADOOP-3827.  
       (Arun Murthy via ddas)
  138. HADOOP-3865. Remove reference to FSNamesystem from metrics preventing
       garbage collection.  
       (Lohit Vijayarenu via cdouglas)

## Release 0.17.2 - Unreleased

- BUG FIXES
     (10)
  1. HADOOP-3678. Avoid spurious exceptions logged at DataNode when clients
     read from DFS.  
     (rangadi)
  2. HADOOP-3760. Fix a bug with HDFS file close() mistakenly introduced
     by HADOOP-3681.  
     (Lohit Vijayarenu via rangadi)
  3. HADOOP-3707. NameNode keeps a count of number of blocks scheduled
     to be written to a datanode and uses it to avoid allocating more
     blocks than a datanode can hold.  
     (rangadi)
  4. HADOOP-3681. DFSClient can get into an infinite loop while closing
     a file if there are some errors.  
     (Lohit Vijayarenu via rangadi)
  5. HADOOP-3002. Hold off block removal while in safe mode.  
     (shv)
  6. HADOOP-3685. Unbalanced replication target.  
     (hairong)
  7. HADOOP-3758. Shutdown datanode on version mismatch instead of retrying
     continuously, preventing excessive logging at the namenode.  
     (lohit vijayarenu via cdouglas)
  8. HADOOP-3633. Correct exception handling in DataXceiveServer, and throttle
     the number of xceiver threads in a data-node.  
     (shv)
  9. HADOOP-3370. Ensure that the TaskTracker.runningJobs data-structure is
     correctly cleaned-up on task completion.  
     (Zheng Shao via acmurthy)
  10. HADOOP-3813. Fix task-output clean-up on HDFS to use the recursive
      FileSystem.delete rather than the FileUtil.fullyDelete.  
      (Amareshwari
      Sri Ramadasu via acmurthy)

## Older Releases

### Release 0.17.1 - 2008-06-23

- INCOMPATIBLE CHANGES
     (1)
  1. HADOOP-3565. Fix the Java serialization, which is not enabled by
     default, to clear the state of the serializer between objects.  
     (tomwhite via omalley)
- IMPROVEMENTS
     (2)
  1. HADOOP-3522. Improve documentation on reduce pointing out that
     input keys and values will be reused.  
     (omalley)
  2. HADOOP-3487. Balancer uses thread pools for managing its threads;
     therefore provides better resource management.  
     (hairong)
- BUG FIXES
     (8)
  1. HADOOP-2159 Namenode stuck in safemode. The counter blockSafe should
     not be decremented for invalid blocks.  
     (hairong)
  2. HADOOP-3472 MapFile.Reader getClosest() function returns incorrect results
     when before is true  
     (Todd Lipcon via Stack)
  3. HADOOP-3442. Limit recursion depth on the stack for QuickSort to prevent
     StackOverflowErrors. To avoid O(n\*n) cases, when partitioning depth
     exceeds
     a multiple of log(n), change to HeapSort.  
     (cdouglas)
  4. HADOOP-3477. Fix build to not package contrib/\*/bin twice in
     distributions.  
     (Adam Heath via cutting)
  5. HADOOP-3475. Fix MapTask to correctly size the accounting allocation of
     io.sort.mb.  
     (cdouglas)
  6. HADOOP-3550. Fix the serialization data structures in MapTask where the
     value lengths are incorrectly calculated.  
     (cdouglas)
  7. HADOOP-3526. Fix contrib/data\_join framework by cloning values retained
     in the reduce.  
     (Spyros Blanas via cdouglas)
  8. HADOOP-1979. Speed up fsck by adding a buffered stream.  
     (Lohit
     Vijaya Renu via omalley)

### Release 0.17.0 - 2008-05-18

- INCOMPATIBLE CHANGES
     (26)
  1. HADOOP-2786. Move hbase out of hadoop core
  2. HADOOP-2345. New HDFS transactions to support appending
     to files. Disk layout version changed from -11 to -12.  
     (dhruba)
  3. HADOOP-2192. Error messages from "dfs mv" command improved.  
     (Mahadev Konar via dhruba)
  4. HADOOP-1902. "dfs du" command without any arguments operates on the
     current working directory.  
     (Mahadev Konar via dhruba)
  5. HADOOP-2873. Fixed bad disk format introduced by HADOOP-2345.
     Disk layout version changed from -12 to -13. See changelist 630992  
     (dhruba)
  6. HADOOP-1985. This addresses rack-awareness for Map tasks and for
     HDFS in a uniform way.  
     (ddas)
  7. HADOOP-1986. Add support for a general serialization mechanism for
     Map Reduce.  
     (tomwhite)
  8. HADOOP-771. FileSystem.delete() takes an explicit parameter that
     specifies whether a recursive delete is intended.  
     (Mahadev Konar via dhruba)
  9. HADOOP-2470. Remove getContentLength(String), open(String, long, long)
     and isDir(String) from ClientProtocol. ClientProtocol version changed
     from 26 to 27. (Tsz Wo (Nicholas), SZE via cdouglas)
  10. HADOOP-2822. Remove deprecated code for classes InputFormatBase and
      PhasedFileSystem.  
      (Amareshwari Sriramadasu via enis)
  11. HADOOP-2116. Changes the layout of the task execution directory.  
      (Amareshwari Sriramadasu via ddas)
  12. HADOOP-2828. The following deprecated methods in Configuration.java
      have been removed
      getObject(String name)
      setObject(String name, Object value)
      get(String name, Object defaultValue)
      set(String name, Object value)
      Iterator entries()  
      (Amareshwari Sriramadasu via ddas)
  13. HADOOP-2824. Removes one deprecated constructor from MiniMRCluster.  
      (Amareshwari Sriramadasu via ddas)
  14. HADOOP-2823. Removes deprecated methods getColumn(), getLine() from
      org.apache.hadoop.record.compiler.generated.SimpleCharStream.  
      (Amareshwari Sriramadasu via ddas)
  15. HADOOP-3060. Removes one unused constructor argument from MiniMRCluster.  
      (Amareshwari Sriramadasu via ddas)
  16. HADOOP-2854. Remove deprecated o.a.h.ipc.Server::getUserInfo().  
      (lohit vijayarenu via cdouglas)
  17. HADOOP-2563. Remove deprecated FileSystem::listPaths.  
      (lohit vijayarenu via cdouglas)
  18. HADOOP-2818. Remove deprecated methods in Counters.  
      (Amareshwari Sriramadasu via tomwhite)
  19. HADOOP-2831. Remove deprecated o.a.h.dfs.INode::getAbsoluteName()  
      (lohit vijayarenu via cdouglas)
  20. HADOOP-2839. Remove deprecated FileSystem::globPaths.  
      (lohit vijayarenu via cdouglas)
  21. HADOOP-2634. Deprecate ClientProtocol::exists.  
      (lohit vijayarenu via cdouglas)
  22. HADOOP-2410. Make EC2 cluster nodes more independent of each other.
      Multiple concurrent EC2 clusters are now supported, and nodes may be
      added to a cluster on the fly with new nodes starting in the same EC2
      availability zone as the cluster. Ganglia monitoring and large
      instance sizes have also been added.  
      (Chris K Wensel via tomwhite)
  23. HADOOP-2826. Deprecated FileSplit.getFile(), LineRecordReader.readLine().  
      (Amareshwari Sriramadasu via ddas)
  24. HADOOP-3239. getFileInfo() returns null for non-existing files instead
      of throwing FileNotFoundException.  
      (Lohit Vijayarenu via shv)
  25. HADOOP-3266. Removed HOD changes from CHANGES.txt, as they are now inside
      src/contrib/hod  
      (Hemanth Yamijala via ddas)
  26. HADOOP-3280. Separate the configuration of the virtual memory size
      (mapred.child.ulimit) from the jvm heap size, so that 64 bit
      streaming applications are supported even when running with 32 bit
      jvms.  
      (acmurthy via omalley)
- NEW FEATURES
     (12)
  1. HADOOP-1398. Add HBase in-memory block cache.  
     (tomwhite)
  2. HADOOP-2178. Job History on DFS.  
     (Amareshwari Sri Ramadasu via ddas)
  3. HADOOP-2063. A new parameter to dfs -get command to fetch a file
     even if it is corrupted. (Tsz Wo (Nicholas), SZE via dhruba)
  4. HADOOP-2219. A new command "df -count" that counts the number of
     files and directories. (Tsz Wo (Nicholas), SZE via dhruba)
  5. HADOOP-2906. Add an OutputFormat capable of using keys, values, and
     config params to map records to different output files.  
     (Runping Qi via cdouglas)
  6. HADOOP-2346. Utilities to support timeout while writing to sockets.
     DFSClient and DataNode sockets have 10min write timeout.  
     (rangadi)
  7. HADOOP-2951. Add a contrib module that provides a utility to
     build or update Lucene indexes using Map/Reduce.  
     (Ning Li via cutting)
  8. HADOOP-1622. Allow multiple jar files for map reduce.  
     (Mahadev Konar via dhruba)
  9. HADOOP-2055. Allows users to set PathFilter on the FileInputFormat.  
     (Alejandro Abdelnur via ddas)
  10. HADOOP-2551. More environment variables like HADOOP\_NAMENODE\_OPTS
      for better control of HADOOP\_OPTS for each component.  
      (rangadi)
  11. HADOOP-3001. Add job counters that measure the number of bytes
      read and written to HDFS, S3, KFS, and local file systems.  
      (omalley)
  12. HADOOP-3048. A new Interface and a default implementation to convert
      and restore serializations of objects to/from strings.  
      (enis)
- IMPROVEMENTS
     (29)
  1. HADOOP-2655. Copy on write for data and metadata files in the
     presence of snapshots. Needed for supporting appends to HDFS
     files.  
     (dhruba)
  2. HADOOP-1967. When a Path specifies the same scheme as the default
     FileSystem but no authority, the default FileSystem's authority is
     used. Also add warnings for old-format FileSystem names, accessor
     methods for fs.default.name, and check for null authority in HDFS.  
     (cutting)
  3. HADOOP-2895. Let the profiling string be configurable.  
     (Martin Traverso via cdouglas)
  4. HADOOP-910. Enables Reduces to do merges for the on-disk map output files
     in parallel with their copying.  
     (Amar Kamat via ddas)
  5. HADOOP-730. Use rename rather than copy for local renames.  
     (cdouglas)
  6. HADOOP-2810. Updated the Hadoop Core logo.  
     (nigel)
  7. HADOOP-2057. Streaming should optionally treat a non-zero exit status
     of a child process as a failed task.  
     (Rick Cox via tomwhite)
  8. HADOOP-2765. Enables specifying ulimits for streaming/pipes tasks  
     (ddas)
  9. HADOOP-2888. Make gridmix scripts more readily configurable and amenable
     to automated execution.  
     (Mukund Madhugiri via cdouglas)
  10. HADOOP-2908. A document that describes the DFS Shell command.  
      (Mahadev Konar via dhruba)
  11. HADOOP-2981. Update README.txt to reflect the upcoming use of
      cryptography.  
      (omalley)
  12. HADOOP-2804. Add support to publish CHANGES.txt as HTML when running
      the Ant 'docs' target.  
      (nigel)
  13. HADOOP-2559. Change DFS block placement to allocate the first replica
      locally, the second off-rack, and the third intra-rack from the
      second.  
      (lohit vijayarenu via cdouglas)
  14. HADOOP-2939. Make the automated patch testing process an executable
      Ant target, test-patch.  
      (nigel)
  15. HADOOP-2239. Add HsftpFileSystem to permit transferring files over ssl.  
      (cdouglas)
  16. HADOOP-2886. Track individual RPC metrics.  
      (girish vaitheeswaran via dhruba)
  17. HADOOP-2373. Improvement in safe-mode reporting.  
      (shv)
  18. HADOOP-3091. Modify FsShell command -put to accept multiple sources.  
      (Lohit Vijaya Renu via cdouglas)
  19. HADOOP-3092. Show counter values from job -status command.  
      (Tom White via ddas)
  20. HADOOP-1228. Ant task to generate Eclipse project files.  
      (tomwhite)
  21. HADOOP-3093. Adds Configuration.getStrings(name, default-value) and
      the corresponding setStrings.  
      (Amareshwari Sriramadasu via ddas)
  22. HADOOP-3106. Adds documentation in forrest for debugging.  
      (Amareshwari Sriramadasu via ddas)
  23. HADOOP-3099. Add an option to distcp to preserve user, group, and
      permission information. (Tsz Wo (Nicholas), SZE via cdouglas)
  24. HADOOP-2841. Unwrap AccessControlException and FileNotFoundException
      from RemoteException for DFSClient.  
      (shv)
  25. HADOOP-3152. Make index interval configuable when using
      MapFileOutputFormat for map-reduce job.  
      (Rong-En Fan via cutting)
  26. HADOOP-3143. Decrease number of slaves from 4 to 3 in TestMiniMRDFSSort,
      as Hudson generates false negatives under the current load.  
      (Nigel Daley via cdouglas)
  27. HADOOP-3174. Illustrative example for MultipleFileInputFormat.  
      (Enis
      Soztutar via acmurthy)
  28. HADOOP-2993. Clarify the usage of JAVA\_HOME in the Quick Start guide.  
      (acmurthy via nigel)
  29. HADOOP-3124. Make DataNode socket write timeout configurable.  
      (rangadi)
- OPTIMIZATIONS
     (12)
  1. HADOOP-2790. Fixed inefficient method hasSpeculativeTask by removing
     repetitive calls to get the current time and late checking to see if
     we want speculation on at all.  
     (omalley)
  2. HADOOP-2758. Reduce buffer copies in DataNode when data is read from
     HDFS, without negatively affecting read throughput.  
     (rangadi)
  3. HADOOP-2399. Input key and value to combiner and reducer is reused.
     (Owen O'Malley via ddas).
  4. HADOOP-2423. Code optimization in FSNamesystem.mkdirs.
     (Tsz Wo (Nicholas), SZE via dhruba)
  5. HADOOP-2606. ReplicationMonitor selects data-nodes to replicate directly
     from needed replication blocks instead of looking up for the blocks for
     each live data-node.  
     (shv)
  6. HADOOP-2148. Eliminate redundant data-node blockMap lookups.  
     (shv)
  7. HADOOP-2027. Return the number of bytes in each block in a file
     via a single rpc to the namenode to speed up job planning.  
     (Lohit Vijaya Renu via omalley)
  8. HADOOP-2902. Replace uses of "fs.default.name" with calls to the
     accessor methods added in HADOOP-1967.  
     (cutting)
  9. HADOOP-2119. Optimize scheduling of jobs with large numbers of
     tasks by replacing static arrays with lists of runnable tasks.  
     (Amar Kamat via omalley)
  10. HADOOP-2919. Reduce the number of memory copies done during the
      map output sorting. Also adds two config variables:
      io.sort.spill.percent - the percentages of io.sort.mb that should
      cause a spill (default 80%)
      io.sort.record.percent - the percent of io.sort.mb that should
      hold key/value indexes (default 5%)  
      (cdouglas via omalley)
  11. HADOOP-3140. Doesn't add a task in the commit queue if the task hadn't
      generated any output.  
      (Amar Kamat via ddas)
  12. HADOOP-3168. Reduce the amount of logging in streaming to an
      exponentially increasing number of records (up to 10,000
      records/log).  
      (Zheng Shao via omalley)
- BUG FIXES
     (102)
  1. HADOOP-2195. '-mkdir' behaviour is now closer to Linux shell in case of
     errors.  
     (Mahadev Konar via rangadi)
  2. HADOOP-2190. bring behaviour '-ls' and '-du' closer to Linux shell
     commands in case of errors.  
     (Mahadev Konar via rangadi)
  3. HADOOP-2193. 'fs -rm' and 'fs -rmr' show error message when the target
     file does not exist.  
     (Mahadev Konar via rangadi)
  4. HADOOP-2738 Text is not subclassable because set(Text) and compareTo(Object)
     access the other instance's private members directly.  
     (jimk)
  5. HADOOP-2779. Remove the references to HBase in the build.xml.  
     (omalley)
  6. HADOOP-2194. dfs cat on a non-existent file throws FileNotFoundException.  
     (Mahadev Konar via dhruba)
  7. HADOOP-2767. Fix for NetworkTopology erroneously skipping the last leaf
     node on a rack.  
     (Hairong Kuang and Mark Butler via dhruba)
  8. HADOOP-1593. FsShell works with paths in non-default FileSystem.  
     (Mahadev Konar via dhruba)
  9. HADOOP-2191. du and dus command on non-existent directory gives
     appropriate error message.  
     (Mahadev Konar via dhruba)
  10. HADOOP-2832. Remove tabs from code of DFSClient for better
      indentation.  
      (dhruba)
  11. HADOOP-2844. distcp closes file handles for sequence files.
      (Tsz Wo (Nicholas), SZE via dhruba)
  12. HADOOP-2727. Fix links in Web UI of the hadoop daemons and some docs  
      (Amareshwari Sri Ramadasu via ddas)
  13. HADOOP-2871. Fixes a problem to do with file: URI in the JobHistory init.  
      (Amareshwari Sri Ramadasu via ddas)
  14. HADOOP-2800. Deprecate SetFile.Writer constructor not the whole class.  
      (Johan Oskarsson via tomwhite)
  15. HADOOP-2891. DFSClient.close() closes all open files.  
      (dhruba)
  16. HADOOP-2845. Fix dfsadmin disk utilization report on Solaris.  
      (Martin Traverso via tomwhite)
  17. HADOOP-2912. MiniDFSCluster restart should wait for namenode to exit
      safemode. This was causing TestFsck to fail.  
      (Mahadev Konar via dhruba)
  18. HADOOP-2820. The following classes in streaming are removed :
      StreamLineRecordReader StreamOutputFormat StreamSequenceRecordReader.  
      (Amareshwari Sri Ramadasu via ddas)
  19. HADOOP-2819. The following methods in JobConf are removed:
      getInputKeyClass() setInputKeyClass getInputValueClass()
      setInputValueClass(Class theClass) setSpeculativeExecution
      getSpeculativeExecution()  
      (Amareshwari Sri Ramadasu via ddas)
  20. HADOOP-2817. Removes deprecated mapred.tasktracker.tasks.maximum and
      ClusterStatus.getMaxTasks().  
      (Amareshwari Sri Ramadasu via ddas)
  21. HADOOP-2821. Removes deprecated ShellUtil and ToolBase classes from
      the util package.  
      (Amareshwari Sri Ramadasu via ddas)
  22. HADOOP-2934. The namenode was encountreing a NPE while loading
      leases from the fsimage. Fixed.  
      (dhruba)
  23. HADOOP-2938. Some fs commands did not glob paths.
      (Tsz Wo (Nicholas), SZE via rangadi)
  24. HADOOP-2943. Compression of intermediate map output causes failures
      in the merge.  
      (cdouglas)
  25. HADOOP-2870. DataNode and NameNode closes all connections while
      shutting down.  
      (Hairong Kuang via dhruba)
  26. HADOOP-2973. Fix TestLocalDFS for Windows platform.
      (Tsz Wo (Nicholas), SZE via dhruba)
  27. HADOOP-2971. select multiple times if it returns early in
      SocketIOWithTimeout.  
      (rangadi)
  28. HADOOP-2955. Fix TestCrcCorruption test failures caused by HADOOP-2758  
      (rangadi)
  29. HADOOP-2657. A flush call on the DFSOutputStream flushes the last
      partial CRC chunk too.  
      (dhruba)
  30. HADOOP-2974. IPC unit tests used "0.0.0.0" to connect to server, which
      is not always supported.  
      (rangadi)
  31. HADOOP-2996. Fixes uses of StringBuffer in StreamUtils class.  
      (Dave Brosius via ddas)
  32. HADOOP-2995. Fixes StreamBaseRecordReader's getProgress to return a
      floating point number.  
      (Dave Brosius via ddas)
  33. HADOOP-2972. Fix for a NPE in FSDataset.invalidate.  
      (Mahadev Konar via dhruba)
  34. HADOOP-2994. Code cleanup for DFSClient: remove redundant
      conversions from string to string.  
      (Dave Brosius via dhruba)
  35. HADOOP-3009. TestFileCreation sometimes fails because restarting
      minidfscluster sometimes creates datanodes with ports that are
      different from their original instance.  
      (dhruba)
  36. HADOOP-2992. Distributed Upgrade framework works correctly with
      more than one upgrade object.  
      (Konstantin Shvachko via dhruba)
  37. HADOOP-2679. Fix a typo in libhdfs.  
      (Jason via dhruba)
  38. HADOOP-2976. When a lease expires, the Namenode ensures that
      blocks of the file are adequately replicated.  
      (dhruba)
  39. HADOOP-2901. Fixes the creation of info servers in the JobClient
      and JobTracker. Removes the creation from JobClient and removes
      additional info server from the JobTracker. Also adds the command
      line utility to view the history files (HADOOP-2896), and fixes
      bugs in JSPs to do with analysis - HADOOP-2742, HADOOP-2792.  
      (Amareshwari Sri Ramadasu via ddas)
  40. HADOOP-2890. If different datanodes report the same block but
      with different sizes to the namenode, the namenode picks the
      replica(s) with the largest size as the only valid replica(s).  
      (dhruba)
  41. HADOOP-2825. Deprecated MapOutputLocation.getFile() is removed.  
      (Amareshwari Sri Ramadasu via ddas)
  42. HADOOP-2806. Fixes a streaming document.  
      (Amareshwari Sriramadasu via ddas)
  43. HADOOP-3008. SocketIOWithTimeout throws InterruptedIOException if the
      thread is interrupted while it is waiting.  
      (rangadi)
  44. HADOOP-3006. Fix wrong packet size reported by DataNode when a block
      is being replicated.  
      (rangadi)
  45. HADOOP-3029. Datanode prints log message "firstbadlink" only if
      it detects a bad connection to another datanode in the pipeline.  
      (dhruba)
  46. HADOOP-3030. Release reserved space for file in InMemoryFileSystem if
      checksum reservation fails.  
      (Devaraj Das via cdouglas)
  47. HADOOP-3036. Fix findbugs warnings in UpgradeUtilities.  
      (Konstantin
      Shvachko via cdouglas)
  48. HADOOP-3025. ChecksumFileSystem supports the delete method with
      the recursive flag.  
      (Mahadev Konar via dhruba)
  49. HADOOP-3012. dfs -mv file to user home directory throws exception if
      the user home directory does not exist.  
      (Mahadev Konar via dhruba)
  50. HADOOP-3066. Should not require superuser privilege to query if hdfs is in
      safe mode  
      (jimk)
  51. HADOOP-3040. If the input line starts with the separator char, the key
      is set as empty.  
      (Amareshwari Sriramadasu via ddas)
  52. HADOOP-3080. Removes flush calls from JobHistory.  
      (Amareshwari Sriramadasu via ddas)
  53. HADOOP-3086. Adds the testcase missed during commit of hadoop-3040.  
      (Amareshwari Sriramadasu via ddas)
  54. HADOOP-3046. Fix the raw comparators for Text and BytesWritables
      to use the provided length rather than recompute it.  
      (omalley)
  55. HADOOP-3094. Fix BytesWritable.toString to avoid extending the sign bit  
      (Owen O'Malley via cdouglas)
  56. HADOOP-3067. DFSInputStream's position read does not close the sockets.  
      (rangadi)
  57. HADOOP-3073. close() on SocketInputStream or SocketOutputStream should
      close the underlying channel.  
      (rangadi)
  58. HADOOP-3087. Fixes a problem to do with refreshing of loadHistory.jsp.  
      (Amareshwari Sriramadasu via ddas)
  59. HADOOP-3065. Better logging message if the rack location of a datanode
      cannot be determined.  
      (Devaraj Das via dhruba)
  60. HADOOP-3064. Commas in a file path should not be treated as delimiters.  
      (Hairong Kuang via shv)
  61. HADOOP-2997. Adds test for non-writable serialier. Also fixes a problem
      introduced by HADOOP-2399.  
      (Tom White via ddas)
  62. HADOOP-3114. Fix TestDFSShell on Windows.  
      (Lohit Vijaya Renu via cdouglas)
  63. HADOOP-3118. Fix Namenode NPE while loading fsimage after a cluster
      upgrade from older disk format.  
      (dhruba)
  64. HADOOP-3161. Fix FIleUtil.HardLink.getLinkCount on Mac OS.  
      (nigel
      via omalley)
  65. HADOOP-2927. Fix TestDU to acurately calculate the expected file size.  
      (shv via nigel)
  66. HADOOP-3123. Fix the native library build scripts to work on Solaris.  
      (tomwhite via omalley)
  67. HADOOP-3089. Streaming should accept stderr from task before
      first key arrives.  
      (Rick Cox via tomwhite)
  68. HADOOP-3146. A DFSOutputStream.flush method is renamed as
      DFSOutputStream.fsync.  
      (dhruba)
  69. HADOOP-3165. -put/-copyFromLocal did not treat input file "-" as stdin.  
      (Lohit Vijayarenu via rangadi)
  70. HADOOP-3041. Deprecate JobConf.setOutputPath and JobConf.getOutputPath.
      Deprecate OutputFormatBase. Add FileOutputFormat. Existing output formats
      extending OutputFormatBase, now extend FileOutputFormat. Add the following
      APIs in FileOutputFormat: setOutputPath, getOutputPath, getWorkOutputPath.  
      (Amareshwari Sriramadasu via nigel)
  71. HADOOP-3083. The fsimage does not store leases. This would have to be
      reworked in the next release to support appends.  
      (dhruba)
  72. HADOOP-3166. Fix an ArrayIndexOutOfBoundsException in the spill thread
      and make exception handling more promiscuous to catch this condition.  
      (cdouglas)
  73. HADOOP-3050. DataNode sends one and only one block report after
      it registers with the namenode.  
      (Hairong Kuang)
  74. HADOOP-3044. NNBench sets the right configuration for the mapper.  
      (Hairong Kuang)
  75. HADOOP-3178. Fix GridMix scripts for small and medium jobs
      to handle input paths differently.  
      (Mukund Madhugiri via nigel)
  76. HADOOP-1911. Fix an infinite loop in DFSClient when all replicas of a
      block are bad  
      (cdouglas)
  77. HADOOP-3157. Fix path handling in DistributedCache and TestMiniMRLocalFS.  
      (Doug Cutting via rangadi)
  78. HADOOP-3018. Fix the eclipse plug-in contrib wrt removed deprecated
      methods  
      (taton)
  79. HADOOP-3183. Fix TestJobShell to use 'ls' instead of java.io.File::exists
      since cygwin symlinks are unsupported.  
      (Mahadev konar via cdouglas)
  80. HADOOP-3175. Fix FsShell.CommandFormat to handle "-" in arguments.  
      (Edward J. Yoon via rangadi)
  81. HADOOP-3220. Safemode message corrected.  
      (shv)
  82. HADOOP-3208. Fix WritableDeserializer to set the Configuration on
      deserialized Writables.  
      (Enis Soztutar via cdouglas)
  83. HADOOP-3224. 'dfs -du /dir' does not return correct size.  
      (Lohit Vjayarenu via rangadi)
  84. HADOOP-3223. Fix typo in help message for -chmod.  
      (rangadi)
  85. HADOOP-1373. checkPath() should ignore case when it compares authoriy.  
      (Edward J. Yoon via rangadi)
  86. HADOOP-3204. Fixes a problem to do with ReduceTask's LocalFSMerger not
      catching Throwable.  
      (Amar Ramesh Kamat via ddas)
  87. HADOOP-3229. Report progress when collecting records from the mapper and
      the combiner.  
      (Doug Cutting via cdouglas)
  88. HADOOP-3225. Unwrapping methods of RemoteException should initialize
      detailedMassage field.  
      (Mahadev Konar, shv, cdouglas)
  89. HADOOP-3247. Fix gridmix scripts to use the correct globbing syntax and
      change maxentToSameCluster to run the correct number of jobs.  
      (Runping Qi via cdouglas)
  90. HADOOP-3242. Fix the RecordReader of SequenceFileAsBinaryInputFormat to
      correctly read from the start of the split and not the beginning of the
      file.  
      (cdouglas via acmurthy)
  91. HADOOP-3256. Encodes the job name used in the filename for history files.  
      (Arun Murthy via ddas)
  92. HADOOP-3162. Ensure that comma-separated input paths are treated correctly
      as multiple input paths.  
      (Amareshwari Sri Ramadasu via acmurthy)
  93. HADOOP-3263. Ensure that the job-history log file always follows the
      pattern of hostname\_timestamp\_jobid\_username\_jobname even if username
      and/or jobname are not specfied. This helps to avoid wrong assumptions
      made about the job-history log filename in jobhistory.jsp.  
      (acmurthy)
  94. HADOOP-3251. Fixes getFilesystemName in JobTracker and LocalJobRunner to
      use FileSystem.getUri instead of FileSystem.getName.  
      (Arun Murthy via ddas)
  95. HADOOP-3237. Fixes TestDFSShell.testErrOutPut on Windows platform.  
      (Mahadev Konar via ddas)
  96. HADOOP-3279. TaskTracker checks for SUCCEEDED task status in addition to
      COMMIT\_PENDING status when it fails maps due to lost map.  
      (Devaraj Das)
  97. HADOOP-3286. Prevent collisions in gridmix output dirs by increasing the
      granularity of the timestamp.  
      (Runping Qi via cdouglas)
  98. HADOOP-3285. Fix input split locality when the splits align to
      fs blocks.  
      (omalley)
  99. HADOOP-3372. Fix heap management in streaming tests.  
      (Arun Murthy via
      cdouglas)
  100. HADOOP-3031. Fix javac warnings in test classes.  
       (cdouglas)
  101. HADOOP-3382. Fix memory leak when files are not cleanly closed  
       (rangadi)
  102. HADOOP-3322. Fix to push MetricsRecord for rpc metrics.  
       (Eric Yang via
       mukund)

### Release 0.16.4 - 2008-05-05

- BUG FIXES
     (3)
  1. HADOOP-3138. DFS mkdirs() should not throw an exception if the directory
     already exists.  
     (rangadi via mukund)
  2. HADOOP-3294. Fix distcp to check the destination length and retry the copy
     if it doesn't match the src length. (Tsz Wo (Nicholas), SZE via mukund)
  3. HADOOP-3186. Fix incorrect permission checkding for mv and renameTo
     in HDFS. (Tsz Wo (Nicholas), SZE via mukund)

### Release 0.16.3 - 2008-04-16

- BUG FIXES
     (7)
  1. HADOOP-3010. Fix ConcurrentModificationException in ipc.Server.Responder.  
     (rangadi)
  2. HADOOP-3154. Catch all Throwables from the SpillThread in MapTask, rather
     than IOExceptions only.  
     (ddas via cdouglas)
  3. HADOOP-3159. Avoid file system cache being overwritten whenever
     configuration is modified. (Tsz Wo (Nicholas), SZE via hairong)
  4. HADOOP-3139. Remove the consistency check for the FileSystem cache in
     closeAll() that causes spurious warnings and a deadlock.
     (Tsz Wo (Nicholas), SZE via cdouglas)
  5. HADOOP-3195. Fix TestFileSystem to be deterministic.
     (Tsz Wo (Nicholas), SZE via cdouglas)
  6. HADOOP-3069. Primary name-node should not truncate image when transferring
     it from the secondary.  
     (shv)
  7. HADOOP-3182. Change permissions of the job-submission directory to 777
     from 733 to ensure sharing of HOD clusters works correctly. (Tsz Wo
     (Nicholas), Sze and Amareshwari Sri Ramadasu via acmurthy)

### Release 0.16.2 - 2008-04-02

- BUG FIXES
     (18)
  1. HADOOP-3011. Prohibit distcp from overwriting directories on the
     destination filesystem with files.  
     (cdouglas)
  2. HADOOP-3033. The BlockReceiver thread in the datanode writes data to
     the block file, changes file position (if needed) and flushes all by
     itself. The PacketResponder thread does not flush block file.  
     (dhruba)
  3. HADOOP-2978. Fixes the JobHistory log format for counters.  
     (Runping Qi via ddas)
  4. HADOOP-2985. Fixes LocalJobRunner to tolerate null job output path.
     Also makes the \_temporary a constant in MRConstants.java.  
     (Amareshwari Sriramadasu via ddas)
  5. HADOOP-3003. FileSystem cache key is updated after a
     FileSystem object is created. (Tsz Wo (Nicholas), SZE via dhruba)
  6. HADOOP-3042. Updates the Javadoc in JobConf.getOutputPath to reflect
     the actual temporary path.  
     (Amareshwari Sriramadasu via ddas)
  7. HADOOP-3007. Tolerate mirror failures while DataNode is replicating
     blocks as it used to before.  
     (rangadi)
  8. HADOOP-2944. Fixes a "Run on Hadoop" wizard NPE when creating a
     Location from the wizard.  
     (taton)
  9. HADOOP-3049. Fixes a problem in MultiThreadedMapRunner to do with
     catching RuntimeExceptions.  
     (Alejandro Abdelnur via ddas)
  10. HADOOP-3039. Fixes a problem to do with exceptions in tasks not
      killing jobs.  
      (Amareshwari Sriramadasu via ddas)
  11. HADOOP-3027. Fixes a problem to do with adding a shutdown hook in
      FileSystem.  
      (Amareshwari Sriramadasu via ddas)
  12. HADOOP-3056. Fix distcp when the target is an empty directory by
      making sure the directory is created first.  
      (cdouglas and acmurthy
      via omalley)
  13. HADOOP-3070. Protect the trash emptier thread from null pointer
      exceptions.  
      (Koji Noguchi via omalley)
  14. HADOOP-3084. Fix HftpFileSystem to work for zero-lenghth files.  
      (cdouglas)
  15. HADOOP-3107. Fix NPE when fsck invokes getListings.  
      (dhruba)
  16. HADOOP-3104. Limit MultithreadedMapRunner to have a fixed length queue
      between the RecordReader and the map threads.  
      (Alejandro Abdelnur via
      omalley)
  17. HADOOP-2833. Do not use "Dr. Who" as the default user in JobClient.
      A valid user name is required. (Tsz Wo (Nicholas), SZE via rangadi)
  18. HADOOP-3128. Throw RemoteException in setPermissions and setOwner of
      DistributedFileSystem.  
      (shv via nigel)

### Release 0.16.1 - 2008-03-13

- INCOMPATIBLE CHANGES
     (1)
  1. HADOOP-2869. Deprecate SequenceFile.setCompressionType in favor of
     SequenceFile.createWriter, SequenceFileOutputFormat.setCompressionType,
     and JobConf.setMapOutputCompressionType. (Arun C Murthy via cdouglas)
     Configuration changes to hadoop-default.xml:
     deprecated io.seqfile.compression.type
- IMPROVEMENTS
     (2)
  1. HADOOP-2371. User guide for file permissions in HDFS.  
     (Robert Chansler via rangadi)
  2. HADOOP-3098. Allow more characters in user and group names while
     using -chown and -chgrp commands.  
     (rangadi)
- BUG FIXES
     (31)
  1. HADOOP-2789. Race condition in IPC Server Responder that could close
     connections early.  
     (Raghu Angadi)
  2. HADOOP-2785. minor. Fix a typo in Datanode block verification  
     (Raghu Angadi)
  3. HADOOP-2788. minor. Fix help message for chgrp shell command (Raghu Angadi).
  4. HADOOP-1188. fstime file is updated when a storage directory containing
     namespace image becomes inaccessible.  
     (shv)
  5. HADOOP-2787. An application can set a configuration variable named
     dfs.umask to set the umask that is used by DFS.
     (Tsz Wo (Nicholas), SZE via dhruba)
  6. HADOOP-2780. The default socket buffer size for DataNodes is 128K.  
     (dhruba)
  7. HADOOP-2716. Superuser privileges for the Balancer.
     (Tsz Wo (Nicholas), SZE via shv)
  8. HADOOP-2754. Filter out .crc files from local file system listing.  
     (Hairong Kuang via shv)
  9. HADOOP-2733. Fix compiler warnings in test code.
     (Tsz Wo (Nicholas), SZE via cdouglas)
  10. HADOOP-2725. Modify distcp to avoid leaving partially copied files at
      the destination after encountering an error. (Tsz Wo (Nicholas), SZE
      via cdouglas)
  11. HADOOP-2391. Cleanup job output directory before declaring a job as
      SUCCESSFUL.  
      (Amareshwari Sri Ramadasu via ddas)
  12. HADOOP-2808. Minor fix to FileUtil::copy to mind the overwrite
      formal.  
      (cdouglas)
  13. HADOOP-2683. Moving UGI out of the RPC Server.
      (Tsz Wo (Nicholas), SZE via shv)
  14. HADOOP-2814. Fix for NPE in datanode in unit test TestDataTransferProtocol.  
      (Raghu Angadi via dhruba)
  15. HADOOP-2811. Dump of counters in job history does not add comma between
      groups.  
      (runping via omalley)
  16. HADOOP-2735. Enables setting TMPDIR for tasks.  
      (Amareshwari Sri Ramadasu via ddas)
  17. HADOOP-2843. Fix protections on map-side join classes to enable derivation.  
      (cdouglas via omalley)
  18. HADOOP-2840. Fix gridmix scripts to correctly invoke the java sort through
      the proper jar.  
      (Mukund Madhugiri via cdouglas)
  19. HADOOP-2769. TestNNThroughputBnechmark should not use a fixed port for
      the namenode http port.  
      (omalley)
  20. HADOOP-2852. Update gridmix benchmark to avoid an artifically long tail.  
      (cdouglas)
  21. HADOOP-2894. Fix a problem to do with tasktrackers failing to connect to
      JobTracker upon reinitialization. (Owen O'Malley via ddas).
  22. HADOOP-2903. Fix exception generated by Metrics while using pushMetric().  
      (girish vaitheeswaran via dhruba)
  23. HADOOP-2904. Fix to RPC metrics to log the correct host name.  
      (girish vaitheeswaran via dhruba)
  24. HADOOP-2918. Improve error logging so that dfs writes failure with
      "No lease on file" can be diagnosed.  
      (dhruba)
  25. HADOOP-2923. Add SequenceFileAsBinaryInputFormat, which was
      missed in the commit for HADOOP-2603.  
      (cdouglas via omalley)
  26. HADOOP-2931. IOException thrown by DFSOutputStream had wrong stack
      trace in some cases.  
      (Michael Bieniosek via rangadi)
  27. HADOOP-2883. Write failures and data corruptions on HDFS files.
      The write timeout is back to what it was on 0.15 release. Also, the
      datnodes flushes the block file buffered output stream before
      sending a positive ack for the packet back to the client.  
      (dhruba)
  28. HADOOP-2756. NPE in DFSClient while closing DFSOutputStreams
      under load.  
      (rangadi)
  29. HADOOP-2958. Fixed FileBench which broke due to HADOOP-2391 which performs
      a check for existence of the output directory and a trivial bug in
      GenericMRLoadGenerator where min/max word lenghts were identical since
      they were looking at the same config variables  
      (Chris Douglas via
      acmurthy)
  30. HADOOP-2915. Fixed FileSystem.CACHE so that a username is included
      in the cache key. (Tsz Wo (Nicholas), SZE via nigel)
  31. HADOOP-2813. TestDU unit test uses its own directory to run its
      sequence of tests.  
      (Mahadev Konar via dhruba)

### Release 0.16.0 - 2008-02-07

- INCOMPATIBLE CHANGES
     (14)
  1. HADOOP-1245. Use the mapred.tasktracker.tasks.maximum value
     configured on each tasktracker when allocating tasks, instead of
     the value configured on the jobtracker. InterTrackerProtocol
     version changed from 5 to 6.  
     (Michael Bieniosek via omalley)
  2. HADOOP-1843. Removed code from Configuration and JobConf deprecated by
     HADOOP-785 and a minor fix to Configuration.toString. Specifically the
     important change is that mapred-default.xml is no longer supported and
     Configuration no longer supports the notion of default/final resources.  
     (acmurthy)
  3. HADOOP-1302. Remove deprecated abacus code from the contrib directory.
     This also fixes a configuration bug in AggregateWordCount, so that the
     job now works.  
     (enis)
  4. HADOOP-2288. Enhance FileSystem API to support access control.
     (Tsz Wo (Nicholas), SZE via dhruba)
  5. HADOOP-2184. RPC Support for user permissions and authentication.  
     (Raghu Angadi via dhruba)
  6. HADOOP-2185. RPC Server uses any available port if the specified
     port is zero. Otherwise it uses the specified port. Also combines
     the configuration attributes for the servers' bind address and
     port from "x.x.x.x" and "y" to "x.x.x.x:y".
     Deprecated configuration variables:
     dfs.info.bindAddress
     dfs.info.port
     dfs.datanode.bindAddress
     dfs.datanode.port
     dfs.datanode.info.bindAdress
     dfs.datanode.info.port
     dfs.secondary.info.bindAddress
     dfs.secondary.info.port
     mapred.job.tracker.info.bindAddress
     mapred.job.tracker.info.port
     mapred.task.tracker.report.bindAddress
     tasktracker.http.bindAddress
     tasktracker.http.port
     New configuration variables (post HADOOP-2404):
     dfs.secondary.http.address
     dfs.datanode.address
     dfs.datanode.http.address
     dfs.http.address
     mapred.job.tracker.http.address
     mapred.task.tracker.report.address
     mapred.task.tracker.http.address  
     (Konstantin Shvachko via dhruba)
  7. HADOOP-2401. Only the current leaseholder can abandon a block for
     a HDFS file. ClientProtocol version changed from 20 to 21.
     (Tsz Wo (Nicholas), SZE via dhruba)
  8. HADOOP-2381. Support permission information in FileStatus. Client
     Protocol version changed from 21 to 22.  
     (Raghu Angadi via dhruba)
  9. HADOOP-2110. Block report processing creates fewer transient objects.
     Datanode Protocol version changed from 10 to 11.  
     (Sanjay Radia via dhruba)
  10. HADOOP-2567. Add FileSystem#getHomeDirectory(), which returns the
      user's home directory in a FileSystem as a fully-qualified path.
      FileSystem#getWorkingDirectory() is also changed to return a
      fully-qualified path, which can break applications that attempt
      to, e.g., pass LocalFileSystem#getWorkingDir().toString() directly
      to java.io methods that accept file names.  
      (cutting)
  11. HADOOP-2514. Change trash feature to maintain a per-user trash
      directory, named ".Trash" in the user's home directory. The
      "fs.trash.root" parameter is no longer used. Full source paths
      are also no longer reproduced within the trash.
  12. HADOOP-2012. Periodic data verification on Datanodes.  
      (Raghu Angadi via dhruba)
  13. HADOOP-1707. The DFSClient does not use a local disk file to cache
      writes to a HDFS file. Changed Data Transfer Version from 7 to 8.  
      (dhruba)
  14. HADOOP-2652. Fix permission issues for HftpFileSystem. This is an
      incompatible change since distcp may not be able to copy files
      from cluster A (compiled with this patch) to cluster B (compiled
      with previous versions). (Tsz Wo (Nicholas), SZE via dhruba)
- NEW FEATURES
     (13)
  1. HADOOP-1857. Ability to run a script when a task fails to capture stack
     traces.  
     (Amareshwari Sri Ramadasu via ddas)
  2. HADOOP-2299. Defination of a login interface. A simple implementation for
     Unix users and groups.  
     (Hairong Kuang via dhruba)
  3. HADOOP-1652. A utility to balance data among datanodes in a HDFS cluster.  
     (Hairong Kuang via dhruba)
  4. HADOOP-2085. A library to support map-side joins of consistently
     partitioned and sorted data sets.  
     (Chris Douglas via omalley)
  5. HADOOP-2336. Shell commands to modify file permissions.  
     (rangadi)
  6. HADOOP-1298. Implement file permissions for HDFS.
     (Tsz Wo (Nicholas) & taton via cutting)
  7. HADOOP-2447. HDFS can be configured to limit the total number of
     objects (inodes and blocks) in the file system.  
     (dhruba)
  8. HADOOP-2487. Added an option to get statuses for all submitted/run jobs.
     This information can be used to develop tools for analysing jobs.  
     (Amareshwari Sri Ramadasu via acmurthy)
  9. HADOOP-1873. Implement user permissions for Map/Reduce framework.  
     (Hairong Kuang via shv)
  10. HADOOP-2532. Add to MapFile a getClosest method that returns the key
      that comes just before if the key is not present.  
      (stack via tomwhite)
  11. HADOOP-1883. Add versioning to Record I/O.  
      (Vivek Ratan via ddas)
  12. HADOOP-2603. Add SeqeunceFileAsBinaryInputFormat, which reads
      sequence files as BytesWritable/BytesWritable regardless of the
      key and value types used to write the file.  
      (cdouglas via omalley)
  13. HADOOP-2367. Add ability to profile a subset of map/reduce tasks and fetch
      the result to the local filesystem of the submitting application. Also
      includes a general IntegerRanges extension to Configuration for setting
      positive, ranged parameters.  
      (Owen O'Malley via cdouglas)
- IMPROVEMENTS
     (54)
  1. HADOOP-2045. Change committer list on website to a table, so that
     folks can list their organization, timezone, etc.  
     (cutting)
  2. HADOOP-2058. Facilitate creating new datanodes dynamically in
     MiniDFSCluster.  
     (Hairong Kuang via dhruba)
  3. HADOOP-1855. fsck verifies block placement policies and reports
     violations.  
     (Konstantin Shvachko via dhruba)
  4. HADOOP-1604. An system administrator can finalize namenode upgrades
     without running the cluster.  
     (Konstantin Shvachko via dhruba)
  5. HADOOP-1839. Link-ify the Pending/Running/Complete/Killed grid in
     jobdetails.jsp to help quickly narrow down and see categorized TIPs'
     details via jobtasks.jsp.  
     (Amar Kamat via acmurthy)
  6. HADOOP-1210. Log counters in job history.  
     (Owen O'Malley via ddas)
  7. HADOOP-1912. Datanode has two new commands COPY and REPLACE. These are
     needed for supporting data rebalance.  
     (Hairong Kuang via dhruba)
  8. HADOOP-2086. This patch adds the ability to add dependencies to a job
     (run via JobControl) after construction.  
     (Adrian Woodhead via ddas)
  9. HADOOP-1185. Support changing the logging level of a server without
     restarting the server. (Tsz Wo (Nicholas), SZE via dhruba)
  10. HADOOP-2134. Remove developer-centric requirements from overview.html and
      keep it end-user focussed, specifically sections related to subversion and
      building Hadoop.  
      (Jim Kellerman via acmurthy)
  11. HADOOP-1989. Support simulated DataNodes. This helps creating large virtual
      clusters for testing purposes.  
      (Sanjay Radia via dhruba)
  12. HADOOP-1274. Support different number of mappers and reducers per
      TaskTracker to allow administrators to better configure and utilize
      heterogenous clusters.
      Configuration changes to hadoop-default.xml:
      add mapred.tasktracker.map.tasks.maximum (default value of 2)
      add mapred.tasktracker.reduce.tasks.maximum (default value of 2)
      remove mapred.tasktracker.tasks.maximum (deprecated for 0.16.0)  
      (Amareshwari Sri Ramadasu via acmurthy)
  13. HADOOP-2104. Adds a description to the ant targets. This makes the
      output of "ant -projecthelp" sensible.  
      (Chris Douglas via ddas)
  14. HADOOP-2127. Added a pipes sort example to benchmark trivial pipes
      application versus trivial java application.  
      (omalley via acmurthy)
  15. HADOOP-2113. A new shell command "dfs -text" to view the contents of
      a gziped or SequenceFile.  
      (Chris Douglas via dhruba)
  16. HADOOP-2207. Add a "package" target for contrib modules that
      permits each to determine what files are copied into release
      builds.  
      (stack via cutting)
  17. HADOOP-1984. Makes the backoff for failed fetches exponential.
      Earlier, it was a random backoff from an interval.  
      (Amar Kamat via ddas)
  18. HADOOP-1327. Include website documentation for streaming.  
      (Rob Weltman
      via omalley)
  19. HADOOP-2000. Rewrite NNBench to measure namenode performance accurately.
      It now uses the map-reduce framework for load generation.  
      (Mukund Madhugiri via dhruba)
  20. HADOOP-2248. Speeds up the framework w.r.t Counters. Also has API
      updates to the Counters part.  
      (Owen O'Malley via ddas)
  21. HADOOP-2326. The initial block report at Datanode startup time has
      a random backoff period.  
      (Sanjay Radia via dhruba)
  22. HADOOP-2432. HDFS includes the name of the file while throwing
      "File does not exist" exception.  
      (Jim Kellerman via dhruba)
  23. HADOOP-2457. Added a 'forrest.home' property to the 'docs' target in
      build.xml.  
      (acmurthy)
  24. HADOOP-2149. A new benchmark for three name-node operation: file create,
      open, and block report, to evaluate the name-node performance
      for optimizations or new features.  
      (Konstantin Shvachko via shv)
  25. HADOOP-2466. Change FileInputFormat.computeSplitSize to a protected
      non-static method to allow sub-classes to provide alternate
      implementations.  
      (Alejandro Abdelnur via acmurthy)
  26. HADOOP-2425. Change TextOutputFormat to handle Text specifically for better
      performance. Make NullWritable implement Comparable. Make TextOutputFormat
      treat NullWritable like null.  
      (omalley)
  27. HADOOP-1719. Improves the utilization of shuffle copier threads.  
      (Amar Kamat via ddas)
  28. HADOOP-2390. Added documentation for user-controls for intermediate
      map-outputs & final job-outputs and native-hadoop libraries.  
      (acmurthy)
  29. HADOOP-1660. Add the cwd of the map/reduce task to the java.library.path
      of the child-jvm to support loading of native libraries distributed via
      the DistributedCache.  
      (acmurthy)
  30. HADOOP-2285. Speeds up TextInputFormat. Also includes updates to the
      Text API.  
      (Owen O'Malley via cdouglas)
  31. HADOOP-2233. Adds a generic load generator for modeling MR jobs.  
      (cdouglas)
  32. HADOOP-2369. Adds a set of scripts for simulating a mix of user map/reduce
      workloads.  
      (Runping Qi via cdouglas)
  33. HADOOP-2547. Removes use of a 'magic number' in build.xml.  
      (Hrishikesh via nigel)
  34. HADOOP-2268. Fix org.apache.hadoop.mapred.jobcontrol classes to use the
      List/Map interfaces rather than concrete ArrayList/HashMap classes
      internally.  
      (Adrian Woodhead via acmurthy)
  35. HADOOP-2406. Add a benchmark for measuring read/write performance through
      the InputFormat interface, particularly with compression.  
      (cdouglas)
  36. HADOOP-2131. Allow finer-grained control over speculative-execution. Now
      users can set it for maps and reduces independently.
      Configuration changes to hadoop-default.xml:
      deprecated mapred.speculative.execution
      add mapred.map.tasks.speculative.execution
      add mapred.reduce.tasks.speculative.execution  
      (Amareshwari Sri Ramadasu via acmurthy)
  37. HADOOP-1965. Interleave sort/spill in teh map-task along with calls to the
      Mapper.map method. This is done by splitting the 'io.sort.mb' buffer into
      two and using one half for collecting map-outputs and the other half for
      sort/spill.  
      (Amar Kamat via acmurthy)
  38. HADOOP-2464. Unit tests for chmod, chown, and chgrp using DFS.  
      (Raghu Angadi)
  39. HADOOP-1876. Persist statuses of completed jobs in HDFS so that the
      JobClient can query and get information about decommissioned jobs and also
      across JobTracker restarts.
      Configuration changes to hadoop-default.xml:
      add mapred.job.tracker.persist.jobstatus.active (default value of false)
      add mapred.job.tracker.persist.jobstatus.hours (default value of 0)
      add mapred.job.tracker.persist.jobstatus.dir (default value of
      /jobtracker/jobsInfo)  
      (Alejandro Abdelnur via acmurthy)
  40. HADOOP-2077. Added version and build information to STARTUP\_MSG for all
      hadoop daemons to aid error-reporting, debugging etc.  
      (acmurthy)
  41. HADOOP-2398. Additional instrumentation for NameNode and RPC server.
      Add support for accessing instrumentation statistics via JMX.  
      (Sanjay radia via dhruba)
  42. HADOOP-2449. A return of the non-MR version of NNBench.  
      (Sanjay Radia via shv)
  43. HADOOP-1989. Remove 'datanodecluster' command from bin/hadoop.  
      (Sanjay Radia via shv)
  44. HADOOP-1742. Improve JavaDoc documentation for ClientProtocol, DFSClient,
      and FSNamesystem.  
      (Konstantin Shvachko)
  45. HADOOP-2298. Add Ant target for a binary-only distribution.  
      (Hrishikesh via nigel)
  46. HADOOP-2509. Add Ant target for Rat report (Apache license header
      reports).  
      (Hrishikesh via nigel)
  47. HADOOP-2469. WritableUtils.clone should take a Configuration
      instead of a JobConf.  
      (stack via omalley)
  48. HADOOP-2659. Introduce superuser permissions for admin operations.
      (Tsz Wo (Nicholas), SZE via shv)
  49. HADOOP-2596. Added a SequenceFile.createWriter api which allows the user
      to specify the blocksize, replication factor and the buffersize to be
      used for the underlying HDFS file.  
      (Alejandro Abdelnur via acmurthy)
  50. HADOOP-2431. Test HDFS File Permissions.  
      (Hairong Kuang via shv)
  51. HADOOP-2232. Add an option to disable Nagle's algorithm in the IPC stack.  
      (Clint Morgan via cdouglas)
  52. HADOOP-2342. Created a micro-benchmark for measuring
      local-file versus hdfs reads.  
      (Owen O'Malley via nigel)
  53. HADOOP-2529. First version of HDFS User Guide.  
      (Raghu Angadi)
  54. HADOOP-2690. Add jar-test target to build.xml, separating compilation
      and packaging of the test classes.  
      (Enis Soztutar via cdouglas)
- OPTIMIZATIONS
     (4)
  1. HADOOP-1898. Release the lock protecting the last time of the last stack
     dump while the dump is happening.  
     (Amareshwari Sri Ramadasu via omalley)
  2. HADOOP-1900. Makes the heartbeat and task event queries interval
     dependent on the cluster size.  
     (Amareshwari Sri Ramadasu via ddas)
  3. HADOOP-2208. Counter update frequency (from TaskTracker to JobTracker) is
     capped at 1 minute.  
     (Amareshwari Sri Ramadasu via ddas)
  4. HADOOP-2284. Reduce the number of progress updates during the sorting in
     the map task.  
     (Amar Kamat via ddas)
- BUG FIXES
     (90)
  1. HADOOP-2583. Fixes a bug in the Eclipse plug-in UI to edit locations.
     Plug-in version is now synchronized with Hadoop version.
  2. HADOOP-2100. Remove faulty check for existence of $HADOOP\_PID\_DIR and let
     'mkdir -p' check & create it.  
     (Michael Bieniosek via acmurthy)
  3. HADOOP-1642. Ensure jobids generated by LocalJobRunner are unique to
     avoid collissions and hence job-failures.  
     (Doug Cutting via acmurthy)
  4. HADOOP-2096. Close open file-descriptors held by streams while localizing
     job.xml in the JobTracker and while displaying it on the webui in
     jobconf.jsp.  
     (Amar Kamat via acmurthy)
  5. HADOOP-2098. Log start & completion of empty jobs to JobHistory, which
     also ensures that we close the file-descriptor of the job's history log
     opened during job-submission.  
     (Amar Kamat via acmurthy)
  6. HADOOP-2112. Adding back changes to build.xml lost while reverting
     HADOOP-1622 i.e. http://svn.apache.org/viewvc?view=rev&revision=588771.  
     (acmurthy)
  7. HADOOP-2089. Fixes the command line argument handling to handle multiple
     -cacheArchive in Hadoop streaming.  
     (Lohit Vijayarenu via ddas)
  8. HADOOP-2071. Fix StreamXmlRecordReader to use a BufferedInputStream
     wrapped over the DFSInputStream since mark/reset aren't supported by
     DFSInputStream anymore.  
     (Lohit Vijayarenu via acmurthy)
  9. HADOOP-1348. Allow XML comments inside configuration files.  
     (Rajagopal Natarajan and Enis Soztutar via enis)
  10. HADOOP-1952. Improve handling of invalid, user-specified classes while
      configuring streaming jobs such as combiner, input/output formats etc.
      Now invalid options are caught, logged and jobs are failed early.  
      (Lohit
      Vijayarenu via acmurthy)
  11. HADOOP-2151. FileSystem.globPaths validates the list of Paths that
      it returns.  
      (Lohit Vijayarenu via dhruba)
  12. HADOOP-2121. Cleanup DFSOutputStream when the stream encountered errors
      when Datanodes became full.  
      (Raghu Angadi via dhruba)
  13. HADOOP-1130. The FileSystem.closeAll() method closes all existing
      DFSClients.  
      (Chris Douglas via dhruba)
  14. HADOOP-2204. DFSTestUtil.waitReplication was not waiting for all replicas
      to get created, thus causing unit test failure.  
      (Raghu Angadi via dhruba)
  15. HADOOP-2078. An zero size file may have no blocks associated with it.  
      (Konstantin Shvachko via dhruba)
  16. HADOOP-2212. ChecksumFileSystem.getSumBufferSize might throw
      java.lang.ArithmeticException. The fix is to initialize bytesPerChecksum
      to 0.  
      (Michael Bieniosek via ddas)
  17. HADOOP-2216. Fix jobtasks.jsp to ensure that it first collects the
      taskids which satisfy the filtering criteria and then use that list to
      print out only the required task-reports, previously it was oblivious to
      the filtering and hence used the wrong index into the array of task-reports.  
      (Amar Kamat via acmurthy)
  18. HADOOP-2272. Fix findbugs target to reflect changes made to the location
      of the streaming jar file by HADOOP-2207.  
      (Adrian Woodhead via nigel)
  19. HADOOP-2244. Fixes the MapWritable.readFields to clear the instance
      field variable every time readFields is called. (Michael Stack via ddas).
  20. HADOOP-2245. Fixes LocalJobRunner to include a jobId in the mapId. Also,
      adds a testcase for JobControl. (Adrian Woodhead via ddas).
  21. HADOOP-2275. Fix erroneous detection of corrupted file when namenode
      fails to allocate any datanodes for newly allocated block.  
      (Dhruba Borthakur via dhruba)
  22. HADOOP-2256. Fix a buf in the namenode that could cause it to encounter
      an infinite loop while deleting excess replicas that were created by
      block rebalancing.  
      (Hairong Kuang via dhruba)
  23. HADOOP-2209. SecondaryNamenode process exits if it encounters exceptions
      that it cannot handle.  
      (Dhruba Borthakur via dhruba)
  24. HADOOP-2314. Prevent TestBlockReplacement from occasionally getting
      into an infinite loop.  
      (Hairong Kuang via dhruba)
  25. HADOOP-2300. This fixes a bug where mapred.tasktracker.tasks.maximum
      would be ignored even if it was set in hadoop-site.xml.  
      (Amareshwari Sri Ramadasu via ddas)
  26. HADOOP-2349. Improve code layout in file system transaction logging code.
      (Tsz Wo (Nicholas), SZE via dhruba)
  27. HADOOP-2368. Fix unit tests on Windows.
      (Tsz Wo (Nicholas), SZE via dhruba)
  28. HADOOP-2363. This fix allows running multiple instances of the unit test
      in parallel. The bug was introduced in HADOOP-2185 that changed
      port-rolling behaviour.  
      (Konstantin Shvachko via dhruba)
  29. HADOOP-2271. Fix chmod task to be non-parallel.  
      (Adrian Woodhead via
      omalley)
  30. HADOOP-2313. Fail the build if building libhdfs fails.  
      (nigel via omalley)
  31. HADOOP-2359. Remove warning for interruptted exception when closing down
      minidfs.  
      (dhruba via omalley)
  32. HADOOP-1841. Prevent slow clients from consuming threads in the NameNode.  
      (dhruba)
  33. HADOOP-2323. JobTracker.close() should not print stack traces for
      normal exit.  
      (jimk via cutting)
  34. HADOOP-2376. Prevents sort example from overriding the number of maps.  
      (Owen O'Malley via ddas)
  35. HADOOP-2434. FSDatasetInterface read interface causes HDFS reads to occur
      in 1 byte chunks, causing performance degradation.  
      (Raghu Angadi via dhruba)
  36. HADOOP-2459. Fix package target so that src/docs/build files are not
      included in the release.  
      (nigel)
  37. HADOOP-2215. Fix documentation in cluster\_setup.html &
      mapred\_tutorial.html reflect that mapred.tasktracker.tasks.maximum has
      been superceeded by mapred.tasktracker.{map|reduce}.tasks.maximum.  
      (Amareshwari Sri Ramadasu via acmurthy)
  38. HADOOP-2459. Fix package target so that src/docs/build files are not
      included in the release.  
      (nigel)
  39. HADOOP-2352. Remove AC\_CHECK\_LIB for libz and liblzo to ensure that
      libhadoop.so doesn't have a dependency on them.  
      (acmurthy)
  40. HADOOP-2453. Fix the configuration for wordcount-simple example in Hadoop
      Pipes which currently produces an XML parsing error.  
      (Amareshwari Sri
      Ramadasu via acmurthy)
  41. HADOOP-2476. Unit test failure while reading permission bits of local
      file system (on Windows) fixed.  
      (Raghu Angadi via dhruba)
  42. HADOOP-2247. Fine-tune the strategies for killing mappers and reducers
      due to failures while fetching map-outputs. Now the map-completion times
      and number of currently running reduces are taken into account by the
      JobTracker before killing the mappers, while the progress made by the
      reducer and the number of fetch-failures vis-a-vis total number of
      fetch-attempts are taken into account before teh reducer kills itself.  
      (Amar Kamat via acmurthy)
  43. HADOOP-2452. Fix eclipse plug-in build.xml to refers to the right
      location where hadoop-\*-core.jar is generated.  
      (taton)
  44. HADOOP-2492. Additional debugging in the rpc server to better
      diagnose ConcurrentModificationException.  
      (dhruba)
  45. HADOOP-2344. Enhance the utility for executing shell commands to read the
      stdout/stderr streams while waiting for the command to finish (to free up
      the buffers). Also, this patch throws away stderr of the DF utility.
      @deprecated
      org.apache.hadoop.fs.ShellCommand for org.apache.hadoop.util.Shell
      org.apache.hadoop.util.ShellUtil for
      org.apache.hadoop.util.Shell.ShellCommandExecutor  
      (Amar Kamat via acmurthy)
  46. HADOOP-2511. Fix a javadoc warning in org.apache.hadoop.util.Shell
      introduced by HADOOP-2344.  
      (acmurthy)
  47. HADOOP-2442. Fix TestLocalFileSystemPermission.testLocalFSsetOwner
      to work on more platforms.  
      (Raghu Angadi via nigel)
  48. HADOOP-2488. Fix a regression in random read performance.  
      (Michael Stack via rangadi)
  49. HADOOP-2523. Fix TestDFSShell.testFilePermissions on Windows.  
      (Raghu Angadi via nigel)
  50. HADOOP-2535. Removed support for deprecated mapred.child.heap.size and
      fixed some indentation issues in TaskRunner. (acmurthy)
      Configuration changes to hadoop-default.xml:
      remove mapred.child.heap.size
  51. HADOOP-2512. Fix error stream handling in Shell. Use exit code to
      detect shell command errors in RawLocalFileSystem.  
      (Raghu Angadi)
  52. HADOOP-2446. Fixes TestHDFSServerPorts and TestMRServerPorts so they
      do not rely on statically configured ports and cleanup better.  
      (nigel)
  53. HADOOP-2537. Make build process compatible with Ant 1.7.0.  
      (Hrishikesh via nigel)
  54. HADOOP-1281. Ensure running tasks of completed map TIPs (e.g. speculative
      tasks) are killed as soon as the TIP completed.  
      (acmurthy)
  55. HADOOP-2571. Suppress a suprious warning in test code.  
      (cdouglas)
  56. HADOOP-2481. NNBench report its progress periodically.  
      (Hairong Kuang via dhruba)
  57. HADOOP-2601. Start name-node on a free port for TestNNThroughputBenchmark.  
      (Konstantin Shvachko)
  58. HADOOP-2494. Set +x on contrib/\*/bin/\* in packaged tar bundle.  
      (stack via tomwhite)
  59. HADOOP-2605. Remove bogus leading slash in task-tracker report bindAddress.  
      (Konstantin Shvachko)
  60. HADOOP-2620. Trivial. 'bin/hadoop fs -help' did not list chmod, chown, and
      chgrp.  
      (Raghu Angadi)
  61. HADOOP-2614. The DFS WebUI accesses are configured to be from the user
      specified by dfs.web.ugi. (Tsz Wo (Nicholas), SZE via dhruba)
  62. HADOOP-2543. Implement a "no-permission-checking" mode for smooth
      upgrade from a pre-0.16 install of HDFS.  
      (Hairong Kuang via dhruba)
  63. HADOOP-290. A DataNode log message now prints the target of a replication
      request correctly.  
      (dhruba)
  64. HADOOP-2538. Redirect to a warning, if plaintext parameter is true but
      the filter parameter is not given in TaskLogServlet.  
      (Michael Bieniosek via enis)
  65. HADOOP-2582. Prevent 'bin/hadoop fs -copyToLocal' from creating
      zero-length files when the src does not exist.  
      (Lohit Vijayarenu via cdouglas)
  66. HADOOP-2189. Incrementing user counters should count as progress.  
      (ddas)
  67. HADOOP-2649. The NameNode periodically computes replication work for
      the datanodes. The periodicity of this computation is now configurable.  
      (dhruba)
  68. HADOOP-2549. Correct disk size computation so that data-nodes could switch
      to other local drives if current is full.  
      (Hairong Kuang via shv)
  69. HADOOP-2633. Fsck should call name-node methods directly rather than
      through rpc. (Tsz Wo (Nicholas), SZE via shv)
  70. HADOOP-2687. Modify a few log message generated by dfs client to be
      logged only at INFO level.  
      (stack via dhruba)
  71. HADOOP-2402. Fix BlockCompressorStream to ensure it buffers data before
      sending it down to the compressor so that each write call doesn't
      compress.  
      (Chris Douglas via acmurthy)
  72. HADOOP-2645. The Metrics initialization code does not throw
      exceptions when servers are restarted by MiniDFSCluster.  
      (Sanjay Radia via dhruba)
  73. HADOOP-2691. Fix a race condition that was causing the DFSClient
      to erroneously remove a good datanode from a pipeline that actually
      had another datanode that was bad.  
      (dhruba)
  74. HADOOP-1195. All code in FSNamesystem checks the return value
      of getDataNode for null before using it.  
      (dhruba)
  75. HADOOP-2640. Fix a bug in MultiFileSplitInputFormat that was always
      returning 1 split in some circumstances.  
      (Enis Soztutar via nigel)
  76. HADOOP-2626. Fix paths with special characters to work correctly
      with the local filesystem.  
      (Thomas Friol via cutting)
  77. HADOOP-2646. Fix SortValidator to work with fully-qualified
      working directories.  
      (Arun C Murthy via nigel)
  78. HADOOP-2092. Added a ping mechanism to the pipes' task to periodically
      check if the parent Java task is running, and exit if the parent isn't
      alive and responding.  
      (Amareshwari Sri Ramadasu via acmurthy)
  79. HADOOP-2714. TestDecommission failed on windows because the replication
      request was timing out.  
      (dhruba)
  80. HADOOP-2576. Namenode performance degradation over time triggered by
      large heartbeat interval.  
      (Raghu Angadi)
  81. HADOOP-2713. TestDatanodeDeath failed on windows because the replication
      request was timing out.  
      (dhruba)
  82. HADOOP-2639. Fixes a problem to do with incorrect maintenance of values
      for runningMapTasks/runningReduceTasks.  
      (Amar Kamat and Arun Murthy
      via ddas)
  83. HADOOP-2723. Fixed the check for checking whether to do user task
      profiling.  
      (Amareshwari Sri Ramadasu via omalley)
  84. HADOOP-2734. Link forrest docs to new http://hadoop.apache.org  
      (Doug Cutting via nigel)
  85. HADOOP-2641. Added Apache license headers to 95 files.  
      (nigel)
  86. HADOOP-2732. Fix bug in path globbing.  
      (Hairong Kuang via nigel)
  87. HADOOP-2404. Fix backwards compatability with hadoop-0.15 configuration
      files that was broken by HADOOP-2185.  
      (omalley)
  88. HADOOP-2755. Fix fsck performance degradation because of permissions
      issue. (Tsz Wo (Nicholas), SZE via dhruba)
  89. HADOOP-2768. Fix performance regression caused by HADOOP-1707.  
      (dhruba borthakur via nigel)
  90. HADOOP-3108. Fix NPE in setPermission and setOwner.  
      (shv)

### Release 0.15.3 - 2008-01-18

- BUG FIXES
     (4)
  1. HADOOP-2562. globPaths supports {ab,cd}.  
     (Hairong Kuang via dhruba)
  2. HADOOP-2540. fsck reports missing blocks incorrectly.  
     (dhruba)
  3. HADOOP-2570. "work" directory created unconditionally, and symlinks
     created from the task cwds.
  4. HADOOP-2574. Fixed mapred\_tutorial.xml to correct minor errors with the
     WordCount examples.  
     (acmurthy)

### Release 0.15.2 - 2008-01-02

- BUG FIXES
     (11)
  1. HADOOP-2246. Moved the changelog for HADOOP-1851 from the NEW FEATURES
     section to the INCOMPATIBLE CHANGES section.  
     (acmurthy)
  2. HADOOP-2238. Fix TaskGraphServlet so that it sets the content type of
     the response appropriately.  
     (Paul Saab via enis)
  3. HADOOP-2129. Fix so that distcp works correctly when source is
     HDFS but not the default filesystem. HDFS paths returned by the
     listStatus() method are now fully-qualified.  
     (cutting)
  4. HADOOP-2378. Fixes a problem where the last task completion event would
     get created after the job completes.  
     (Alejandro Abdelnur via ddas)
  5. HADOOP-2228. Checks whether a job with a certain jobId is already running
     and then tries to create the JobInProgress object.  
     (Johan Oskarsson via ddas)
  6. HADOOP-2422. dfs -cat multiple files fail with 'Unable to write to
     output stream'.  
     (Raghu Angadi via dhruba)
  7. HADOOP-2460. When the namenode encounters ioerrors on writing a
     transaction log, it stops writing new transactions to that one.  
     (Raghu Angadi via dhruba)
  8. HADOOP-2227. Use the LocalDirAllocator uniformly for handling all of the
     temporary storage required for a given task. It also implies that
     mapred.local.dir.minspacestart is handled by checking if there is enough
     free-space on any one of the available disks.  
     (Amareshwari Sri Ramadasu
     via acmurthy)
  9. HADOOP-2437. Fix the LocalDirAllocator to choose the seed for the
     round-robin disk selections randomly. This helps in spreading data across
     multiple partitions much better.  
     (acmurhty)
  10. HADOOP-2486. When the list of files from the InMemoryFileSystem is obtained
      for merging, this patch will ensure that only those files whose checksums
      have also got created (renamed) are returned.  
      (ddas)
  11. HADOOP-2456. Hardcode English locale to prevent NumberFormatException
      from occurring when starting the NameNode with certain locales.  
      (Matthias Friedrich via nigel)
- IMPROVEMENTS
     (4)
  1. HADOOP-2160. Remove project-level, non-user documentation from
     releases, since it's now maintained in a separate tree.  
     (cutting)
  2. HADOOP-1327. Add user documentation for streaming.  
     (cutting)
  3. HADOOP-2382. Add hadoop-default.html to subversion.  
     (cutting)
  4. HADOOP-2158. hdfsListDirectory calls FileSystem.listStatus instead
     of FileSystem.listPaths. This reduces the number of RPC calls on the
     namenode, thereby improving scalability.  
     (Christian Kunz via dhruba)

### Release 0.15.1 - 2007-11-27

- INCOMPATIBLE CHANGES
     (1)
  1. HADOOP-713. Reduce CPU usage on namenode while listing directories.
     FileSystem.listPaths does not return the size of the entire subtree.
     Introduced a new API ClientProtocol.getContentLength that returns the
     size of the subtree.  
     (Dhruba Borthakur via dhruba)
- IMPROVEMENTS
     (1)
  1. HADOOP-1917. Addition of guides/tutorial for better overall
     documentation for Hadoop. Specifically:
     \* quickstart.html is targetted towards first-time users and helps them
     setup a single-node cluster and play with Hadoop.
     \* cluster\_setup.html helps admins to configure and setup non-trivial
     hadoop clusters.
     \* mapred\_tutorial.html is a comprehensive Map-Reduce tutorial.  
     (acmurthy)
- BUG FIXES
     (3)
  1. HADOOP-2174. Removed the unnecessary Reporter.setStatus call from
     FSCopyFilesMapper.close which led to a NPE since the reporter isn't valid
     in the close method.  
     (Chris Douglas via acmurthy)
  2. HADOOP-2172. Restore performance of random access to local files
     by caching positions of local input streams, avoiding a system
     call.  
     (cutting)
  3. HADOOP-2205. Regenerate the Hadoop website since some of the changes made
     by HADOOP-1917 weren't correctly copied over to the trunk/docs directory.
     Also fixed a couple of minor typos and broken links.  
     (acmurthy)

### Release 0.15.0 - 2007-11-2

- INCOMPATIBLE CHANGES
     (10)
  1. HADOOP-1708. Make files appear in namespace as soon as they are
     created.  
     (Dhruba Borthakur via dhruba)
  2. HADOOP-999. A HDFS Client immediately informs the NameNode of a new
     file creation. ClientProtocol version changed from 14 to 15.
     (Tsz Wo (Nicholas), SZE via dhruba)
  3. HADOOP-932. File locking interfaces and implementations (that were
     earlier deprecated) are removed. Client Protocol version changed
     from 15 to 16.  
     (Raghu Angadi via dhruba)
  4. HADOOP-1621. FileStatus is now a concrete class and FileSystem.listPaths
     is deprecated and replaced with listStatus.  
     (Chris Douglas via omalley)
  5. HADOOP-1656. The blockSize of a file is stored persistently in the file
     inode.  
     (Dhruba Borthakur via dhruba)
  6. HADOOP-1838. The blocksize of files created with an earlier release is
     set to the default block size.  
     (Dhruba Borthakur via dhruba)
  7. HADOOP-785. Add support for 'final' Configuration parameters,
     removing support for 'mapred-default.xml', and changing
     'hadoop-site.xml' to not override other files. Now folks should
     generally use 'hadoop-site.xml' for all configurations. Values
     with a 'final' tag may not be overridden by subsequently loaded
     configuration files, e.g., by jobs.  
     (Arun C. Murthy via cutting)
  8. HADOOP-1846. DatanodeReport in ClientProtocol can report live
     datanodes, dead datanodes or all datanodes. Client Protocol version
     changed from 17 to 18.  
     (Hairong Kuang via dhruba)
  9. HADOOP-1851. Permit specification of map output compression type
     and codec, independent of the final output's compression
     parameters.  
     (Arun C Murthy via cutting)
  10. HADOOP-1819. Jobtracker cleanups, including binding ports before
      clearing state directories, so that inadvertently starting a
      second jobtracker doesn't trash one that's already running. Removed
      method JobTracker.getTracker() because the static variable, which
      stored the value caused initialization problems.  
      (omalley via cutting)
- NEW FEATURES
     (14)
  1. HADOOP-89. A client can access file data even before the creator
     has closed the file. Introduce a new command "tail" from dfs shell.  
     (Dhruba Borthakur via dhruba)
  2. HADOOP-1636. Allow configuration of the number of jobs kept in
     memory by the JobTracker.  
     (Michael Bieniosek via omalley)
  3. HADOOP-1667. Reorganize CHANGES.txt into sections to make it
     easier to read. Also remove numbering, to make merging easier.  
     (cutting)
  4. HADOOP-1610. Add metrics for failed tasks.  
     (Devaraj Das via tomwhite)
  5. HADOOP-1767. Add "bin/hadoop job -list" sub-command.  
     (taton via cutting)
  6. HADOOP-1351. Add "bin/hadoop job [-fail-task|-kill-task]" sub-commands
     to terminate a particular task-attempt.  
     (Enis Soztutar via acmurthy)
  7. HADOOP-1880. SleepJob : An example job that sleeps at each map and
     reduce task.  
     (enis)
  8. HADOOP-1809. Add a link in web site to #hadoop IRC channel.  
     (enis)
  9. HADOOP-1894. Add percentage graphs and mapred task completion graphs
     to Web User Interface. Users not using Firefox may install a plugin to
     their browsers to see svg graphics.  
     (enis)
  10. HADOOP-1914. Introduce a new NamenodeProtocol to allow secondary
      namenodes and rebalancing processes to communicate with a primary
      namenode.  
      (Hairong Kuang via dhruba)
  11. HADOOP-1963. Add a FileSystem implementation for the Kosmos
      Filesystem (KFS).  
      (Sriram Rao via cutting)
  12. HADOOP-1822. Allow the specialization and configuration of socket
      factories. Provide a StandardSocketFactory, and a SocksSocketFactory to
      allow the use of SOCKS proxies. (taton).
  13. HADOOP-1968. FileSystem supports wildcard input syntax "{ }".  
      (Hairong Kuang via dhruba)
  14. HADOOP-2566. Add globStatus method to the FileSystem interface
      and deprecate globPath and listPath.  
      (Hairong Kuang via hairong)
- OPTIMIZATIONS
     (8)
  1. HADOOP-1910. Reduce the number of RPCs that DistributedFileSystem.create()
     makes to the namenode.  
     (Raghu Angadi via dhruba)
  2. HADOOP-1565. Reduce memory usage of NameNode by replacing
     TreeMap in HDFS Namespace with ArrayList.  
     (Dhruba Borthakur via dhruba)
  3. HADOOP-1743. Change DFS INode from a nested class to standalone
     class, with specialized subclasses for directories and files, to
     save memory on the namenode.  
     (Konstantin Shvachko via cutting)
  4. HADOOP-1759. Change file name in INode from String to byte[],
     saving memory on the namenode.  
     (Konstantin Shvachko via cutting)
  5. HADOOP-1766. Save memory in namenode by having BlockInfo extend
     Block, and replace many uses of Block with BlockInfo.  
     (Konstantin Shvachko via cutting)
  6. HADOOP-1687. Save memory in namenode by optimizing BlockMap
     representation.  
     (Konstantin Shvachko via cutting)
  7. HADOOP-1774. Remove use of INode.parent in Block CRC upgrade.  
     (Raghu Angadi via dhruba)
  8. HADOOP-1788. Increase the buffer size on the Pipes command socket.  
     (Amareshwari Sri Ramadasu and Christian Kunz via omalley)
- BUG FIXES
     (64)
  1. HADOOP-1946. The Datanode code does not need to invoke du on
     every heartbeat.  
     (Hairong Kuang via dhruba)
  2. HADOOP-1935. Fix a NullPointerException in internalReleaseCreate.  
     (Dhruba Borthakur)
  3. HADOOP-1933. The nodes listed in include and exclude files
     are always listed in the datanode report.  
     (Raghu Angadi via dhruba)
  4. HADOOP-1953. The job tracker should wait beteween calls to try and delete
     the system directory  
     (Owen O'Malley via devaraj)
  5. HADOOP-1932. TestFileCreation fails with message saying filestatus.dat
     is of incorrect size.  
     (Dhruba Borthakur via dhruba)
  6. HADOOP-1573. Support for 0 reducers in PIPES.  
     (Owen O'Malley via devaraj)
  7. HADOOP-1500. Fix typographical errors in the DFS WebUI.  
     (Nigel Daley via dhruba)
  8. HADOOP-1076. Periodic checkpoint can continue even if an earlier
     checkpoint encountered an error.  
     (Dhruba Borthakur via dhruba)
  9. HADOOP-1887. The Namenode encounters an ArrayIndexOutOfBoundsException
     while listing a directory that had a file that was
     being actively written to.  
     (Dhruba Borthakur via dhruba)
  10. HADOOP-1904. The Namenode encounters an exception because the
      list of blocks per datanode-descriptor was corrupted.  
      (Konstantin Shvachko via dhruba)
  11. HADOOP-1762. The Namenode fsimage does not contain a list of
      Datanodes.  
      (Raghu Angadi via dhruba)
  12. HADOOP-1890. Removed debugging prints introduced by HADOOP-1774.  
      (Raghu Angadi via dhruba)
  13. HADOOP-1763. Too many lost task trackers on large clusters due to
      insufficient number of RPC handler threads on the JobTracker.  
      (Devaraj Das)
  14. HADOOP-1463. HDFS report correct usage statistics for disk space
      used by HDFS.  
      (Hairong Kuang via dhruba)
  15. HADOOP-1692. In DFS ant task, don't cache the Configuration.  
      (Chris Douglas via cutting)
  16. HADOOP-1726. Remove lib/jetty-ext/ant.jar.  
      (omalley)
  17. HADOOP-1772. Fix hadoop-daemon.sh script to get correct hostname
      under Cygwin. (Tsz Wo (Nicholas), SZE via cutting)
  18. HADOOP-1749. Change TestDFSUpgrade to sort files, fixing sporadic
      test failures.  
      (Enis Soztutar via cutting)
  19. HADOOP-1748. Fix tasktracker to be able to launch tasks when log
      directory is relative.  
      (omalley via cutting)
  20. HADOOP-1775. Fix a NullPointerException and an
      IllegalArgumentException in MapWritable.  
      (Jim Kellerman via cutting)
  21. HADOOP-1795. Fix so that jobs can generate output file names with
      special characters.  
      (Fr??d??ric Bertin via cutting)
  22. HADOOP-1810. Fix incorrect value type in MRBench (SmallJobs)  
      (Devaraj Das via tomwhite)
  23. HADOOP-1806. Fix ant task to compile again, also fix default
      builds to compile ant tasks.  
      (Chris Douglas via cutting)
  24. HADOOP-1758. Fix escape processing in librecordio to not be
      quadratic.  
      (Vivek Ratan via cutting)
  25. HADOOP-1817. Fix MultiFileSplit to read and write the split
      length, so that it is not always zero in map tasks.  
      (Thomas Friol via cutting)
  26. HADOOP-1853. Fix contrib/streaming to accept multiple -cacheFile
      options.  
      (Prachi Gupta via cutting)
  27. HADOOP-1818. Fix MultiFileInputFormat so that it does not return
      empty splits when numPaths < numSplits.  
      (Thomas Friol via enis)
  28. HADOOP-1840. Fix race condition which leads to task's diagnostic
      messages getting lost.  
      (acmurthy)
  29. HADOOP-1885. Fix race condition in MiniDFSCluster shutdown.  
      (Chris Douglas via nigel)
  30. HADOOP-1889. Fix path in EC2 scripts for building your own AMI.  
      (tomwhite)
  31. HADOOP-1892. Fix a NullPointerException in the JobTracker when
      trying to fetch a task's diagnostic messages from the JobClient.  
      (Amar Kamat via acmurthy)
  32. HADOOP-1897. Completely remove about.html page from the web site.  
      (enis)
  33. HADOOP-1907. Fix null pointer exception when getting task diagnostics
      in JobClient.  
      (Christian Kunz via omalley)
  34. HADOOP-1882. Remove spurious asterisks from decimal number displays.  
      (Raghu Angadi via cutting)
  35. HADOOP-1783. Make S3 FileSystem return Paths fully-qualified with
      scheme and host.  
      (tomwhite)
  36. HADOOP-1925. Make pipes' autoconf script look for libsocket and libnsl, so
      that it can compile under Solaris.  
      (omalley)
  37. HADOOP-1940. TestDFSUpgradeFromImage must shut down its MiniDFSCluster.  
      (Chris Douglas via nigel)
  38. HADOOP-1930. Fix the blame for failed fetchs on the right host.  
      (Arun C.
      Murthy via omalley)
  39. HADOOP-1934. Fix the platform name on Mac to use underscores rather than
      spaces.  
      (omalley)
  40. HADOOP-1959. Use "/" instead of File.separator in the StatusHttpServer.  
      (jimk via omalley)
  41. HADOOP-1626. Improve dfsadmin help messages.  
      (Lohit Vijayarenu via dhruba)
  42. HADOOP-1695. The SecondaryNamenode waits for the Primary NameNode to
      start up.  
      (Dhruba Borthakur)
  43. HADOOP-1983. Have Pipes flush the command socket when progress is sent
      to prevent timeouts during long computations.  
      (omalley)
  44. HADOOP-1875. Non-existant directories or read-only directories are
      filtered from dfs.client.buffer.dir.  
      (Hairong Kuang via dhruba)
  45. HADOOP-1992. Fix the performance degradation in the sort validator.  
      (acmurthy via omalley)
  46. HADOOP-1874. Move task-outputs' promotion/discard to a separate thread
      distinct from the main heartbeat-processing thread. The main upside being
      that we do not lock-up the JobTracker during HDFS operations, which
      otherwise may lead to lost tasktrackers if the NameNode is unresponsive.  
      (Devaraj Das via acmurthy)
  47. HADOOP-2026. Namenode prints out one log line for "Number of transactions"
      at most once every minute.  
      (Dhruba Borthakur)
  48. HADOOP-2022. Ensure that status information for successful tasks is correctly
      recorded at the JobTracker, so that, for example, one may view correct
      information via taskdetails.jsp. This bug was introduced by HADOOP-1874.  
      (Amar Kamat via acmurthy)
  49. HADOOP-2031. Correctly maintain the taskid which takes the TIP to
      completion, failing which the case of lost tasktrackers isn't handled
      properly i.e. the map TIP is incorrectly left marked as 'complete' and it
      is never rescheduled elsewhere, leading to hung reduces.  
      (Devaraj Das via acmurthy)
  50. HADOOP-2018. The source datanode of a data transfer waits for
      a response from the target datanode before closing the data stream.  
      (Hairong Kuang via dhruba)
  51. HADOOP-2023. Disable TestLocalDirAllocator on Windows.  
      (Hairong Kuang via nigel)
  52. HADOOP-2016. Ignore status-updates from FAILED/KILLED tasks at the
      TaskTracker. This fixes a race-condition which caused the tasks to wrongly
      remain in the RUNNING state even after being killed by the JobTracker and
      thus handicap the cleanup of the task's output sub-directory.  
      (acmurthy)
  53. HADOOP-1771. Fix a NullPointerException in streaming caused by an
      IOException in MROutputThread.  
      (lohit vijayarenu via nigel)
  54. HADOOP-2028. Fix distcp so that the log dir does not need to be
      specified and the destination does not need to exist.  
      (Chris Douglas via nigel)
  55. HADOOP-2044. The namenode protects all lease manipulations using a
      sortedLease lock.  
      (Dhruba Borthakur)
  56. HADOOP-2051. The TaskCommit thread should not die for exceptions other
      than the InterruptedException. This behavior is there for the other long
      running threads in the JobTracker.  
      (Arun C Murthy via ddas)
  57. HADOOP-1973. The FileSystem object would be accessed on the JobTracker
      through a RPC in the InterTrackerProtocol. The check for the object being
      null was missing and hence NPE would be thrown sometimes. This issue fixes
      that problem.  
      (Amareshwari Sri Ramadasu via ddas)
  58. HADOOP-2033. The SequenceFile.Writer.sync method was a no-op, which caused
      very uneven splits for applications like distcp that count on them.  
      (omalley)
  59. HADOOP-2070. Added a flush method to pipes' DownwardProtocol and call
      that before waiting for the application to finish to ensure all buffered
      data is flushed.  
      (Owen O'Malley via acmurthy)
  60. HADOOP-2080. Fixed calculation of the checksum file size when the values
      are large.  
      (omalley)
  61. HADOOP-2048. Change error handling in distcp so that each map copies
      as much as possible before reporting the error. Also report progress on
      every copy.  
      (Chris Douglas via omalley)
  62. HADOOP-2073. Change size of VERSION file after writing contents to it.  
      (Konstantin Shvachko via dhruba)
  63. HADOOP-2102. Fix the deprecated ToolBase to pass its Configuration object
      to the superceding ToolRunner to ensure it picks up the appropriate
      configuration resources.  
      (Dennis Kubes and Enis Soztutar via acmurthy)
  64. HADOOP-2103. Fix minor javadoc bugs introduce by HADOOP-2046.  
      (Nigel
      Daley via acmurthy)
- IMPROVEMENTS
     (37)
  1. HADOOP-1908. Restructure data node code so that block sending and
     receiving are seperated from data transfer header handling.  
     (Hairong Kuang via dhruba)
  2. HADOOP-1921. Save the configuration of completed/failed jobs and make them
     available via the web-ui.  
     (Amar Kamat via devaraj)
  3. HADOOP-1266. Remove dependency of package org.apache.hadoop.net on
     org.apache.hadoop.dfs.  
     (Hairong Kuang via dhruba)
  4. HADOOP-1779. Replace INodeDirectory.getINode() by a getExistingPathINodes()
     to allow the retrieval of all existing INodes along a given path in a
     single lookup. This facilitates removal of the 'parent' field in the
     inode.  
     (Christophe Taton via dhruba)
  5. HADOOP-1756. Add toString() to some Writable-s.  
     (ab)
  6. HADOOP-1727. New classes: MapWritable and SortedMapWritable.  
     (Jim Kellerman via ab)
  7. HADOOP-1651. Improve progress reporting.  
     (Devaraj Das via tomwhite)
  8. HADOOP-1595. dfsshell can wait for a file to achieve its intended
     replication target. (Tsz Wo (Nicholas), SZE via dhruba)
  9. HADOOP-1693. Remove un-needed log fields in DFS replication classes,
     since the log may be accessed statically.  
     (Konstantin Shvachko via cutting)
  10. HADOOP-1231. Add generics to Mapper and Reducer interfaces.  
      (tomwhite via cutting)
  11. HADOOP-1436. Improved command-line APIs, so that all tools need
      not subclass ToolBase, and generic parameter parser is public.  
      (Enis Soztutar via cutting)
  12. HADOOP-1703. DFS-internal code cleanups, removing several uses of
      the obsolete UTF8.  
      (Christophe Taton via cutting)
  13. HADOOP-1731. Add Hadoop's version to contrib jar file names.  
      (cutting)
  14. HADOOP-1689. Make shell scripts more portable. All shell scripts
      now explicitly depend on bash, but do not require that bash be
      installed in a particular location, as long as it is on $PATH.  
      (cutting)
  15. HADOOP-1744. Remove many uses of the deprecated UTF8 class from
      the HDFS namenode.  
      (Christophe Taton via cutting)
  16. HADOOP-1654. Add IOUtils class, containing generic io-related
      utility methods.  
      (Enis Soztutar via cutting)
  17. HADOOP-1158. Change JobTracker to record map-output transmission
      errors and use them to trigger speculative re-execution of tasks.  
      (Arun C Murthy via cutting)
  18. HADOOP-1601. Change GenericWritable to use ReflectionUtils for
      instance creation, avoiding classloader issues, and to implement
      Configurable.  
      (Enis Soztutar via cutting)
  19. HADOOP-1750. Log standard output and standard error when forking
      task processes.  
      (omalley via cutting)
  20. HADOOP-1803. Generalize build.xml to make files in all
      src/contrib/\*/bin directories executable.  
      (stack via cutting)
  21. HADOOP-1739. Let OS always choose the tasktracker's umbilical
      port. Also switch default address for umbilical connections to
      loopback.  
      (cutting)
  22. HADOOP-1812. Let OS choose ports for IPC and RPC unit tests.  
      (cutting)
  23. HADOOP-1825. Create $HADOOP\_PID\_DIR when it does not exist.  
      (Michael Bieniosek via cutting)
  24. HADOOP-1425. Replace uses of ToolBase with the Tool interface.  
      (Enis Soztutar via cutting)
  25. HADOOP-1569. Reimplement DistCP to use the standard FileSystem/URI
      code in Hadoop so that you can copy from and to all of the supported file
      systems.  
      (Chris Douglas via omalley)
  26. HADOOP-1018. Improve documentation w.r.t handling of lost hearbeats between
      TaskTrackers and JobTracker.  
      (acmurthy)
  27. HADOOP-1718. Add ant targets for measuring code coverage with clover.  
      (simonwillnauer via nigel)
  28. HADOOP-1592. Log error messages to the client console when tasks
      fail.  
      (Amar Kamat via cutting)
  29. HADOOP-1879. Remove some unneeded casts.  
      (Nilay Vaish via cutting)
  30. HADOOP-1878. Add space between priority links on job details
      page.  
      (Thomas Friol via cutting)
  31. HADOOP-120. In ArrayWritable, prevent creation with null value
      class, and improve documentation.  
      (Cameron Pope via cutting)
  32. HADOOP-1926. Add a random text writer example/benchmark so that we can
      benchmark compression codecs on random data.  
      (acmurthy via omalley)
  33. HADOOP-1906. Warn the user if they have an obsolete madred-default.xml
      file in their configuration directory.  
      (acmurthy via omalley)
  34. HADOOP-1971. Warn when job does not specify a jar.  
      (enis via cutting)
  35. HADOOP-1942. Increase the concurrency of transaction logging to
      edits log. Reduce the number of syncs by double-buffering the changes
      to the transaction log.  
      (Dhruba Borthakur)
  36. HADOOP-2046. Improve mapred javadoc.  
      (Arun C. Murthy via cutting)
  37. HADOOP-2105. Improve overview.html to clarify supported platforms,
      software pre-requisites for hadoop, how to install them on various
      platforms and a better general description of hadoop and it's utility.  
      (Jim Kellerman via acmurthy)

### Release 0.14.4 - 2007-11-26

- BUG FIXES
     (3)
  1. HADOOP-2140. Add missing Apache Licensing text at the front of several
     C and C++ files.
  2. HADOOP-2169. Fix the DT\_SONAME field of libhdfs.so to set it to the
     correct value of 'libhdfs.so', currently it is set to the absolute path of
     libhdfs.so.  
     (acmurthy)
  3. HADOOP-2001. Make the job priority updates and job kills synchronized on
     the JobTracker. Deadlock was seen in the JobTracker because of the lack of
     this synchronization.  
     (Arun C Murthy via ddas)

### Release 0.14.3 - 2007-10-19

- BUG FIXES
     (3)
  1. HADOOP-2053. Fixed a dangling reference to a memory buffer in the map
     output sorter.  
     (acmurthy via omalley)
  2. HADOOP-2036. Fix a NullPointerException in JvmMetrics class.  
     (nigel)
  3. HADOOP-2043. Release 0.14.2 was compiled with Java 1.6 rather than
     Java 1.5.  
     (cutting)

### Release 0.14.2 - 2007-10-09

- BUG FIXES
     (9)
  1. HADOOP-1948. Removed spurious error message during block crc upgrade.  
     (Raghu Angadi via dhruba)
  2. HADOOP-1862. reduces are getting stuck trying to find map outputs.  
     (Arun C. Murthy via ddas)
  3. HADOOP-1977. Fixed handling of ToolBase cli options in JobClient.  
     (enis via omalley)
  4. HADOOP-1972. Fix LzoCompressor to ensure the user has actually asked
     to finish compression.  
     (arun via omalley)
  5. HADOOP-1970. Fix deadlock in progress reporting in the task.  
     (Vivek
     Ratan via omalley)
  6. HADOOP-1978. Name-node removes edits.new after a successful startup.  
     (Konstantin Shvachko via dhruba)
  7. HADOOP-1955. The Namenode tries to not pick the same source Datanode for
     a replication request if the earlier replication request for the same
     block and that source Datanode had failed.  
     (Raghu Angadi via dhruba)
  8. HADOOP-1961. The -get option to dfs-shell works when a single filename
     is specified.  
     (Raghu Angadi via dhruba)
  9. HADOOP-1997. TestCheckpoint closes the edits file after writing to it,
     otherwise the rename of this file on Windows fails.  
     (Konstantin Shvachko via dhruba)

### Release 0.14.1 - 2007-09-04

- BUG FIXES
     (3)
  1. HADOOP-1740. Fix null pointer exception in sorting map outputs.  
     (Devaraj
     Das via omalley)
  2. HADOOP-1790. Fix tasktracker to work correctly on multi-homed
     boxes.  
     (Torsten Curdt via cutting)
  3. HADOOP-1798. Fix jobtracker to correctly account for failed
     tasks.  
     (omalley via cutting)

### Release 0.14.0 - 2007-08-17

- INCOMPATIBLE CHANGES
     (160)
  1. HADOOP-1134.
     CONFIG/API - dfs.block.size must now be a multiple of
     io.byte.per.checksum, otherwise new files can not be written.
     LAYOUT - DFS layout version changed from -6 to -7, which will require an
     upgrade from previous versions.
     PROTOCOL - Datanode RPC protocol version changed from 7 to 8.
  2. HADOOP-1283
     API - deprecated file locking API.
  3. HADOOP-894
     PROTOCOL - changed ClientProtocol to fetch parts of block locations.
  4. HADOOP-1336
     CONFIG - Enable speculative execution by default.
  5. HADOOP-1197
     API - deprecated method for Configuration.getObject, because
     Configurations should only contain strings.
  6. HADOOP-1343
     API - deprecate Configuration.set(String,Object) so that only strings are
     put in Configrations.
  7. HADOOP-1207
     CLI - Fix FsShell 'rm' command to continue when a non-existent file is
     encountered.
  8. HADOOP-1473
     CLI/API - Job, TIP, and Task id formats have changed and are now unique
     across job tracker restarts.
  9. HADOOP-1400
     API - JobClient constructor now takes a JobConf object instead of a
     Configuration object.

     NEW FEATURES and BUG FIXES
  10. HADOOP-1197. In Configuration, deprecate getObject() and add
      getRaw(), which skips variable expansion.  
      (omalley via cutting)
  11. HADOOP-1343. In Configuration, deprecate set(String,Object) and
      implement Iterable.  
      (omalley via cutting)
  12. HADOOP-1344. Add RunningJob#getJobName().  
      (Michael Bieniosek via cutting)
  13. HADOOP-1342. In aggregators, permit one to limit the number of
      unique values per key.  
      (Runping Qi via cutting)
  14. HADOOP-1340. Set the replication factor of the MD5 file in the filecache
      to be the same as the replication factor of the original file.  
      (Dhruba Borthakur via tomwhite.)
  15. HADOOP-1355. Fix null pointer dereference in
      TaskLogAppender.append(LoggingEvent).  
      (Arun C Murthy via tomwhite.)
  16. HADOOP-1357. Fix CopyFiles to correctly avoid removing "/".  
      (Arun C Murthy via cutting)
  17. HADOOP-234. Add pipes facility, which permits writing MapReduce
      programs in C++.
  18. HADOOP-1359. Fix a potential NullPointerException in HDFS.  
      (Hairong Kuang via cutting)
  19. HADOOP-1364. Fix inconsistent synchronization in SequenceFile.  
      (omalley via cutting)
  20. HADOOP-1379. Add findbugs target to build.xml.  
      (Nigel Daley via cutting)
  21. HADOOP-1364. Fix various inconsistent synchronization issues.  
      (Devaraj Das via cutting)
  22. HADOOP-1393. Remove a potential unexpected negative number from
      uses of random number generator.  
      (omalley via cutting)
  23. HADOOP-1387. A number of "performance" code-cleanups suggested
      by findbugs.  
      (Arun C Murthy via cutting)
  24. HADOOP-1401. Add contrib/hbase javadoc to tree.  
      (stack via cutting)
  25. HADOOP-894. Change HDFS so that the client only retrieves a limited
      number of block locations per request from the namenode.  
      (Konstantin Shvachko via cutting)
  26. HADOOP-1406. Plug a leak in MapReduce's use of metrics.  
      (David Bowen via cutting)
  27. HADOOP-1394. Implement "performance" code-cleanups in HDFS
      suggested by findbugs.  
      (Raghu Angadi via cutting)
  28. HADOOP-1413. Add example program that uses Knuth's dancing links
      algorithm to solve pentomino problems.  
      (omalley via cutting)
  29. HADOOP-1226. Change HDFS so that paths it returns are always
      fully qualified.  
      (Dhruba Borthakur via cutting)
  30. HADOOP-800. Improvements to HDFS web-based file browser.  
      (Enis Soztutar via cutting)
  31. HADOOP-1408. Fix a compiler warning by adding a class to replace
      a generic.  
      (omalley via cutting)
  32. HADOOP-1376. Modify RandomWriter example so that it can generate
      data for the Terasort benchmark.  
      (Devaraj Das via cutting)
  33. HADOOP-1429. Stop logging exceptions during normal IPC server
      shutdown.  
      (stack via cutting)
  34. HADOOP-1461. Fix the synchronization of the task tracker to
      avoid lockups in job cleanup.  
      (Arun C Murthy via omalley)
  35. HADOOP-1446. Update the TaskTracker metrics while the task is
      running.  
      (Devaraj via omalley)
  36. HADOOP-1414. Fix a number of issues identified by FindBugs as
      "Bad Practice".  
      (Dhruba Borthakur via cutting)
  37. HADOOP-1392. Fix "correctness" bugs identified by FindBugs in
      fs and dfs packages.  
      (Raghu Angadi via cutting)
  38. HADOOP-1412. Fix "dodgy" bugs identified by FindBugs in fs and
      io packages.  
      (Hairong Kuang via cutting)
  39. HADOOP-1261. Remove redundant events from HDFS namenode's edit
      log when a datanode restarts.  
      (Raghu Angadi via cutting)
  40. HADOOP-1336. Re-enable speculative execution by
      default.  
      (omalley via cutting)
  41. HADOOP-1311. Fix a bug in BytesWritable#set() where start offset
      was ignored.  
      (Dhruba Borthakur via cutting)
  42. HADOOP-1450. Move checksumming closer to user code, so that
      checksums are created before data is stored in large buffers and
      verified after data is read from large buffers, to better catch
      memory errors.  
      (cutting)
  43. HADOOP-1447. Add support in contrib/data\_join for text inputs.  
      (Senthil Subramanian via cutting)
  44. HADOOP-1456. Fix TestDecommission assertion failure by setting
      the namenode to ignore the load on datanodes while allocating
      replicas.  
      (Dhruba Borthakur via tomwhite)
  45. HADOOP-1396. Fix FileNotFoundException on DFS block.  
      (Dhruba Borthakur via tomwhite)
  46. HADOOP-1467. Remove redundant counters from WordCount example.  
      (Owen O'Malley via tomwhite)
  47. HADOOP-1139. Log HDFS block transitions at INFO level, to better
      enable diagnosis of problems.  
      (Dhruba Borthakur via cutting)
  48. HADOOP-1269. Finer grained locking in HDFS namenode.  
      (Dhruba Borthakur via cutting)
  49. HADOOP-1438. Improve HDFS documentation, correcting typos and
      making images appear in PDF. Also update copyright date for all
      docs.  
      (Luke Nezda via cutting)
  50. HADOOP-1457. Add counters for monitoring task assignments.  
      (Arun C Murthy via tomwhite)
  51. HADOOP-1472. Fix so that timed-out tasks are counted as failures
      rather than as killed.  
      (Arun C Murthy via cutting)
  52. HADOOP-1234. Fix a race condition in file cache that caused
      tasktracker to not be able to find cached files.  
      (Arun C Murthy via cutting)
  53. HADOOP-1482. Fix secondary namenode to roll info port.  
      (Dhruba Borthakur via cutting)
  54. HADOOP-1300. Improve removal of excess block replicas to be
      rack-aware. Attempts are now made to keep replicas on more
      racks.  
      (Hairong Kuang via cutting)
  55. HADOOP-1417. Disable a few FindBugs checks that generate a lot
      of spurious warnings.  
      (Nigel Daley via cutting)
  56. HADOOP-1320. Rewrite RandomWriter example to bypass reduce.  
      (Arun C Murthy via cutting)
  57. HADOOP-1449. Add some examples to contrib/data\_join.  
      (Senthil Subramanian via cutting)
  58. HADOOP-1459. Fix so that, in HDFS, getFileCacheHints() returns
      hostnames instead of IP addresses.  
      (Dhruba Borthakur via cutting)
  59. HADOOP-1493. Permit specification of "java.library.path" system
      property in "mapred.child.java.opts" configuration property.  
      (Enis Soztutar via cutting)
  60. HADOOP-1372. Use LocalDirAllocator for HDFS temporary block
      files, so that disk space, writability, etc. is considered.  
      (Dhruba Borthakur via cutting)
  61. HADOOP-1193. Pool allocation of compression codecs. This
      eliminates a memory leak that could cause OutOfMemoryException,
      and also substantially improves performance.  
      (Arun C Murthy via cutting)
  62. HADOOP-1492. Fix a NullPointerException handling version
      mismatch during datanode registration.  
      (Konstantin Shvachko via cutting)
  63. HADOOP-1442. Fix handling of zero-length input splits.  
      (Senthil Subramanian via cutting)
  64. HADOOP-1444. Fix HDFS block id generation to check pending
      blocks for duplicates.  
      (Dhruba Borthakur via cutting)
  65. HADOOP-1207. Fix FsShell's 'rm' command to not stop when one of
      the named files does not exist.  
      (Tsz Wo Sze via cutting)
  66. HADOOP-1475. Clear tasktracker's file cache before it
      re-initializes, to avoid confusion.  
      (omalley via cutting)
  67. HADOOP-1505. Remove spurious stacktrace in ZlibFactory
      introduced in HADOOP-1093.  
      (Michael Stack via tomwhite)
  68. HADOOP-1484. Permit one to kill jobs from the web ui. Note that
      this is disabled by default. One must set
      "webinterface.private.actions" to enable this.  
      (Enis Soztutar via cutting)
  69. HADOOP-1003. Remove flushing of namenode edit log from primary
      namenode lock, increasing namenode throughput.  
      (Dhruba Borthakur via cutting)
  70. HADOOP-1023. Add links to searchable mail archives.  
      (tomwhite via cutting)
  71. HADOOP-1504. Fix terminate-hadoop-cluster script in contrib/ec2
      to only terminate Hadoop instances, and not other instances
      started by the same user.  
      (tomwhite via cutting)
  72. HADOOP-1462. Improve task progress reporting. Progress reports
      are no longer blocking since i/o is performed in a separate
      thread. Reporting during sorting and more is also more
      consistent.  
      (Vivek Ratan via cutting)
  73. [ intentionally blank ]
  74. HADOOP-1453. Remove some unneeded calls to FileSystem#exists()
      when opening files, reducing the namenode load somewhat.  
      (Raghu Angadi via cutting)
  75. HADOOP-1489. Fix text input truncation bug due to mark/reset.
      Add a unittest.  
      (Bwolen Yang via cutting)
  76. HADOOP-1455. Permit specification of arbitrary job options on
      pipes command line.  
      (Devaraj Das via cutting)
  77. HADOOP-1501. Better randomize sending of block reports to
      namenode, so reduce load spikes.  
      (Dhruba Borthakur via cutting)
  78. HADOOP-1147. Remove @author tags from Java source files.
  79. HADOOP-1283. Convert most uses of UTF8 in the namenode to be
      String.  
      (Konstantin Shvachko via cutting)
  80. HADOOP-1511. Speedup hbase unit tests.  
      (stack via cutting)
  81. HADOOP-1517. Remove some synchronization in namenode to permit
      finer grained locking previously added.  
      (Konstantin Shvachko via cutting)
  82. HADOOP-1512. Fix failing TestTextInputFormat on Windows.  
      (Senthil Subramanian via nigel)
  83. HADOOP-1518. Add a session id to job metrics, for use by HOD.  
      (David Bowen via cutting)
  84. HADOOP-1292. Change 'bin/hadoop fs -get' to first copy files to
      a temporary name, then rename them to their final name, so that
      failures don't leave partial files.  
      (Tsz Wo Sze via cutting)
  85. HADOOP-1377. Add support for modification time to FileSystem and
      implement in HDFS and local implementations. Also, alter access
      to file properties to be through a new FileStatus interface.  
      (Dhruba Borthakur via cutting)
  86. HADOOP-1515. Add MultiFileInputFormat, which can pack multiple,
      typically small, input files into each split.  
      (Enis Soztutar via cutting)
  87. HADOOP-1514. Make reducers report progress while waiting for map
      outputs, so they're not killed.  
      (Vivek Ratan via cutting)
  88. HADOOP-1508. Add an Ant task for FsShell operations. Also add
      new FsShell commands "touchz", "test" and "stat".  
      (Chris Douglas via cutting)
  89. HADOOP-1028. Add log messages for server startup and shutdown.  
      (Tsz Wo Sze via cutting)
  90. HADOOP-1485. Add metrics for monitoring shuffle.  
      (Devaraj Das via cutting)
  91. HADOOP-1536. Remove file locks from libhdfs tests.  
      (Dhruba Borthakur via nigel)
  92. HADOOP-1520. Add appropriate synchronization to FSEditsLog.  
      (Dhruba Borthakur via nigel)
  93. HADOOP-1513. Fix a race condition in directory creation.  
      (Devaraj via omalley)
  94. HADOOP-1546. Remove spurious column from HDFS web UI.  
      (Dhruba Borthakur via cutting)
  95. HADOOP-1556. Make LocalJobRunner delete working files at end of
      job run.  
      (Devaraj Das via tomwhite)
  96. HADOOP-1571. Add contrib lib directories to root build.xml
      javadoc classpath.  
      (Michael Stack via tomwhite)
  97. HADOOP-1554. Log killed tasks to the job history and display them on the
      web/ui.  
      (Devaraj Das via omalley)
  98. HADOOP-1533. Add persistent error logging for distcp. The logs are stored
      into a specified hdfs directory.  
      (Senthil Subramanian via omalley)
  99. HADOOP-1286. Add support to HDFS for distributed upgrades, which
      permits coordinated upgrade of datanode data.  
      (Konstantin Shvachko via cutting)
  100. HADOOP-1580. Improve contrib/streaming so that subprocess exit
       status is displayed for errors.  
       (John Heidemann via cutting)
  101. HADOOP-1448. In HDFS, randomize lists of non-local block
       locations returned to client, so that load is better balanced.  
       (Hairong Kuang via cutting)
  102. HADOOP-1578. Fix datanode to send its storage id to namenode
       during registration.  
       (Konstantin Shvachko via cutting)
  103. HADOOP-1584. Fix a bug in GenericWritable which limited it to
       128 types instead of 256.  
       (Espen Amble Kolstad via cutting)
  104. HADOOP-1473. Make job ids unique across jobtracker restarts.  
       (omalley via cutting)
  105. HADOOP-1582. Fix hdfslib to return 0 instead of -1 at
       end-of-file, per C conventions.  
       (Christian Kunz via cutting)
  106. HADOOP-911. Fix a multithreading bug in libhdfs.  
       (Christian Kunz)
  107. HADOOP-1486. Fix so that fatal exceptions in namenode cause it
       to exit.  
       (Dhruba Borthakur via cutting)
  108. HADOOP-1470. Factor checksum generation and validation out of
       ChecksumFileSystem so that it can be reused by FileSystem's with
       built-in checksumming.  
       (Hairong Kuang via cutting)
  109. HADOOP-1590. Use relative urls in jobtracker jsp pages, so that
       webapp can be used in non-root contexts.  
       (Thomas Friol via cutting)
  110. HADOOP-1596. Fix the parsing of taskids by streaming and improve the
       error reporting.  
       (omalley)
  111. HADOOP-1535. Fix the user-controlled grouping to the reduce function.  
       (Vivek Ratan via omalley)
  112. HADOOP-1585. Modify GenericWritable to declare the classes as subtypes
       of Writable  
       (Espen Amble Kolstad via omalley)
  113. HADOOP-1576. Fix errors in count of completed tasks when
       speculative execution is enabled.  
       (Arun C Murthy via cutting)
  114. HADOOP-1598. Fix license headers: adding missing; updating old.  
       (Enis Soztutar via cutting)
  115. HADOOP-1547. Provide examples for aggregate library.  
       (Runping Qi via tomwhite)
  116. HADOOP-1570. Permit jobs to enable and disable the use of
       hadoop's native library.  
       (Arun C Murthy via cutting)
  117. HADOOP-1433. Add job priority.  
       (Johan Oskarsson via tomwhite)
  118. HADOOP-1597. Add status reports and post-upgrade options to HDFS
       distributed upgrade.  
       (Konstantin Shvachko via cutting)
  119. HADOOP-1524. Permit user task logs to appear as they're
       created.  
       (Michael Bieniosek via cutting)
  120. HADOOP-1599. Fix distcp bug on Windows.  
       (Senthil Subramanian via cutting)
  121. HADOOP-1562. Add JVM metrics, including GC and logging stats.  
       (David Bowen via cutting)
  122. HADOOP-1613. Fix "DFS Health" page to display correct time of
       last contact.  
       (Dhruba Borthakur via cutting)
  123. HADOOP-1134. Add optimized checksum support to HDFS. Checksums
       are now stored with each block, rather than as parallel files.
       This reduces the namenode's memory requirements and increases
       data integrity.  
       (Raghu Angadi via cutting)
  124. HADOOP-1400. Make JobClient retry requests, so that clients can
       survive jobtracker problems.  
       (omalley via cutting)
  125. HADOOP-1564. Add unit tests for HDFS block-level checksums.  
       (Dhruba Borthakur via cutting)
  126. HADOOP-1620. Reduce the number of abstract FileSystem methods,
       simplifying implementations.  
       (cutting)
  127. HADOOP-1625. Fix a "could not move files" exception in datanode.  
       (Raghu Angadi via cutting)
  128. HADOOP-1624. Fix an infinite loop in datanode.  
       (Raghu Angadi via cutting)
  129. HADOOP-1084. Switch mapred file cache to use file modification
       time instead of checksum to detect file changes, as checksums are
       no longer easily accessed.  
       (Arun C Murthy via cutting)
  130. HADOOP-1623. Fix an infinite loop when copying directories.  
       (Dhruba Borthakur via cutting)
  131. HADOOP-1603. Fix a bug in namenode initialization where
       default replication is sometimes reset to one on restart.  
       (Raghu Angadi via cutting)
  132. HADOOP-1635. Remove hardcoded keypair name and fix launch-hadoop-cluster
       to support later versions of ec2-api-tools.  
       (Stu Hood via tomwhite)
  133. HADOOP-1638. Fix contrib EC2 scripts to support NAT addressing.  
       (Stu Hood via tomwhite)
  134. HADOOP-1632. Fix an IllegalArgumentException in fsck.  
       (Hairong Kuang via cutting)
  135. HADOOP-1619. Fix FSInputChecker to not attempt to read past EOF.  
       (Hairong Kuang via cutting)
  136. HADOOP-1640. Fix TestDecommission on Windows.  
       (Dhruba Borthakur via cutting)
  137. HADOOP-1587. Fix TestSymLink to get required system properties.  
       (Devaraj Das via omalley)
  138. HADOOP-1628. Add block CRC protocol unit tests.  
       (Raghu Angadi via omalley)
  139. HADOOP-1653. FSDirectory code-cleanups. FSDirectory.INode
       becomes a static class.  
       (Christophe Taton via dhruba)
  140. HADOOP-1066. Restructure documentation to make more user
       friendly.  
       (Connie Kleinjans and Jeff Hammerbacher via cutting)
  141. HADOOP-1551. libhdfs supports setting replication factor and
       retrieving modification time of files.  
       (Sameer Paranjpye via dhruba)
  142. HADOOP-1647. FileSystem.getFileStatus returns valid values for "/".  
       (Dhruba Borthakur via dhruba)
  143. HADOOP-1657. Fix NNBench to ensure that the block size is a
       multiple of bytes.per.checksum.  
       (Raghu Angadi via dhruba)
  144. HADOOP-1553. Replace user task output and log capture code to use shell
       redirection instead of copier threads in the TaskTracker. Capping the
       size of the output is now done via tail in memory and thus should not be
       large. The output of the tasklog servlet is not forced into UTF8 and is
       not buffered entirely in memory. (omalley)
       Configuration changes to hadoop-default.xml:
       remove mapred.userlog.num.splits
       remove mapred.userlog.purge.splits
       change default mapred.userlog.limit.kb to 0 (no limit)
       change default mapred.userlog.retain.hours to 24
       Configuration changes to log4j.properties:
       remove log4j.appender.TLA.noKeepSplits
       remove log4j.appender.TLA.purgeLogSplits
       remove log4j.appender.TLA.logsRetainHours
       URL changes:
       http://<tasktracker>/tasklog.jsp -> http://<tasktracker>tasklog with
       parameters limited to start and end, which may be positive (from
       start) or negative (from end).
       Environment:
       require bash (v2 or later) and tail
  145. HADOOP-1659. Fix a job id/job name mixup.  
       (Arun C. Murthy via omalley)
  146. HADOOP-1665. With HDFS Trash enabled and the same file was created
       and deleted more than once, the suceeding deletions creates Trash item
       names suffixed with a integer.  
       (Dhruba Borthakur via dhruba)
  147. HADOOP-1666. FsShell object can be used for multiple fs commands.  
       (Dhruba Borthakur via dhruba)
  148. HADOOP-1654. Remove performance regression introduced by Block CRC.  
       (Raghu Angadi via dhruba)
  149. HADOOP-1680. Improvements to Block CRC upgrade messages.  
       (Raghu Angadi via dhruba)
  150. HADOOP-71. Allow Text and SequenceFile Map/Reduce inputs from non-default
       filesystems.  
       (omalley)
  151. HADOOP-1568. Expose HDFS as xml/http filesystem to provide cross-version
       compatability.  
       (Chris Douglas via omalley)
  152. HADOOP-1668. Added an INCOMPATIBILITY section to CHANGES.txt.  
       (nigel)
  153. HADOOP-1629. Added a upgrade test for HADOOP-1134.  
       (Raghu Angadi via nigel)
  154. HADOOP-1698. Fix performance problems on map output sorting for jobs
       with large numbers of reduces.  
       (Devaraj Das via omalley)
  155. HADOOP-1716. Fix a Pipes wordcount example to remove the 'file:'
       schema from its output path.  
       (omalley via cutting)
  156. HADOOP-1714. Fix TestDFSUpgradeFromImage to work on Windows.  
       (Raghu Angadi via nigel)
  157. HADOOP-1663. Return a non-zero exit code if streaming fails.  
       (Lohit Renu
       via omalley)
  158. HADOOP-1712. Fix an unhandled exception on datanode during block
       CRC upgrade.  
       (Raghu Angadi via cutting)
  159. HADOOP-1717. Fix TestDFSUpgradeFromImage to work on Solaris.  
       (nigel via cutting)
  160. HADOOP-1437. Add Eclipse plugin in contrib.  
       (Eugene Hung and Christophe Taton via cutting)

### Release 0.13.0 - 2007-06-08

1. HADOOP-1047. Fix TestReplication to succeed more reliably.  
   (Hairong Kuang via cutting)
2. HADOOP-1063. Fix a race condition in MiniDFSCluster test code.  
   (Hairong Kuang via cutting)
3. HADOOP-1101. In web ui, split shuffle statistics from reduce
   statistics, and add some task averages.  
   (Devaraj Das via cutting)
4. HADOOP-1071. Improve handling of protocol version mismatch in
   JobTracker.  
   (Tahir Hashmi via cutting)
5. HADOOP-1116. Increase heap size used for contrib unit tests.  
   (Philippe Gassmann via cutting)
6. HADOOP-1120. Add contrib/data\_join, tools to simplify joining
   data from multiple sources using MapReduce.  
   (Runping Qi via cutting)
7. HADOOP-1064. Reduce log level of some DFSClient messages.  
   (Dhruba Borthakur via cutting)
8. HADOOP-1137. Fix StatusHttpServer to work correctly when
   resources are in a jar file.  
   (Benjamin Reed via cutting)
9. HADOOP-1094. Optimize generated Writable implementations for
   records to not allocate a new BinaryOutputArchive or
   BinaryInputArchive per call.  
   (Milind Bhandarkar via cutting)
10. HADOOP-1068. Improve error message for clusters with 0 datanodes.  
    (Dhruba Borthakur via tomwhite)
11. HADOOP-1122. Fix divide-by-zero exception in FSNamesystem
    chooseTarget method.  
    (Dhruba Borthakur via tomwhite)
12. HADOOP-1131. Add a closeAll() static method to FileSystem.  
    (Philippe Gassmann via tomwhite)
13. HADOOP-1085. Improve port selection in HDFS and MapReduce test
    code. Ports are now selected by the OS during testing rather than
    by probing for free ports, improving test reliability.  
    (Arun C Murthy via cutting)
14. HADOOP-1153. Fix HDFS daemons to correctly stop their threads.  
    (Konstantin Shvachko via cutting)
15. HADOOP-1146. Add a counter for reduce input keys and rename the
    "reduce input records" counter to be "reduce input groups".  
    (David Bowen via cutting)
16. HADOOP-1165. In records, replace idential generated toString
    methods with a method on the base class.  
    (Milind Bhandarkar via cutting)
17. HADOOP-1164. Fix TestReplicationPolicy to specify port zero, so
    that a free port is automatically selected.  
    (omalley via cutting)
18. HADOOP-1166. Add a NullOutputFormat and use it in the
    RandomWriter example.  
    (omalley via cutting)
19. HADOOP-1169. Fix a cut/paste error in CopyFiles utility so that
    S3-based source files are correctly copied.  
    (Michael Stack via cutting)
20. HADOOP-1167. Remove extra synchronization in InMemoryFileSystem.  
    (omalley via cutting)
21. HADOOP-1110. Fix an off-by-one error counting map inputs.  
    (David Bowen via cutting)
22. HADOOP-1178. Fix a NullPointerException during namenode startup.  
    (Dhruba Borthakur via cutting)
23. HADOOP-1011. Fix a ConcurrentModificationException when viewing
    job history.  
    (Tahir Hashmi via cutting)
24. HADOOP-672. Improve help for fs shell commands.  
    (Dhruba Borthakur via cutting)
25. HADOOP-1170. Improve datanode performance by removing device
    checks from common operations.  
    (Igor Bolotin via cutting)
26. HADOOP-1090. Fix SortValidator's detection of whether the input
    file belongs to the sort-input or sort-output directory.  
    (Arun C Murthy via tomwhite)
27. HADOOP-1081. Fix bin/hadoop on Darwin.  
    (Michael Bieniosek via cutting)
28. HADOOP-1045. Add contrib/hbase, a BigTable-like online database.  
    (Jim Kellerman via cutting)
29. HADOOP-1156. Fix a NullPointerException in MiniDFSCluster.  
    (Hairong Kuang via cutting)
30. HADOOP-702. Add tools to help automate HDFS upgrades.  
    (Konstantin Shvachko via cutting)
31. HADOOP-1163. Fix ganglia metrics to aggregate metrics from different
    hosts properly.  
    (Michael Bieniosek via tomwhite)
32. HADOOP-1194. Make compression style record level for map output
    compression.  
    (Arun C Murthy via tomwhite)
33. HADOOP-1187. Improve DFS Scalability: avoid scanning entire list of
    datanodes in getAdditionalBlocks.  
    (Dhruba Borthakur via tomwhite)
34. HADOOP-1133. Add tool to analyze and debug namenode on a production
    cluster.  
    (Dhruba Borthakur via tomwhite)
35. HADOOP-1151. Remove spurious printing to stderr in streaming
    PipeMapRed.  
    (Koji Noguchi via tomwhite)
36. HADOOP-988. Change namenode to use a single map of blocks to metadata.  
    (Raghu Angadi via tomwhite)
37. HADOOP-1203. Change UpgradeUtilities used by DFS tests to use
    MiniDFSCluster to start and stop NameNode/DataNodes.  
    (Nigel Daley via tomwhite)
38. HADOOP-1217. Add test.timeout property to build.xml, so that
    long-running unit tests may be automatically terminated.  
    (Nigel Daley via cutting)
39. HADOOP-1149. Improve DFS Scalability: make
    processOverReplicatedBlock() a no-op if blocks are not
    over-replicated.  
    (Raghu Angadi via tomwhite)
40. HADOOP-1149. Improve DFS Scalability: optimize getDistance(),
    contains(), and isOnSameRack() in NetworkTopology.  
    (Hairong Kuang via tomwhite)
41. HADOOP-1218. Make synchronization on TaskTracker's RunningJob
    object consistent.  
    (Devaraj Das via tomwhite)
42. HADOOP-1219. Ignore progress report once a task has reported as
    'done'.  
    (Devaraj Das via tomwhite)
43. HADOOP-1114. Permit user to specify additional CLASSPATH elements
    with a HADOOP\_CLASSPATH environment variable.  
    (cutting)
44. HADOOP-1198. Remove ipc.client.timeout parameter override from
    unit test configuration. Using the default is more robust and
    has almost the same run time.  
    (Arun C Murthy via tomwhite)
45. HADOOP-1211. Remove deprecated constructor and unused static
    members in DataNode class.  
    (Konstantin Shvachko via tomwhite)
46. HADOOP-1136. Fix ArrayIndexOutOfBoundsException in
    FSNamesystem$UnderReplicatedBlocks add() method.  
    (Hairong Kuang via tomwhite)
47. HADOOP-978. Add the client name and the address of the node that
    previously started to create the file to the description of
    AlreadyBeingCreatedException.  
    (Konstantin Shvachko via tomwhite)
48. HADOOP-1001. Check the type of keys and values generated by the
    mapper against the types specified in JobConf.  
    (Tahir Hashmi via tomwhite)
49. HADOOP-971. Improve DFS Scalability: Improve name node performance
    by adding a hostname to datanodes map.  
    (Hairong Kuang via tomwhite)
50. HADOOP-1189. Fix 'No space left on device' exceptions on datanodes.  
    (Raghu Angadi via tomwhite)
51. HADOOP-819. Change LineRecordWriter to not insert a tab between
    key and value when either is null, and to print nothing when both
    are null.  
    (Runping Qi via cutting)
52. HADOOP-1204. Rename InputFormatBase to be FileInputFormat, and
    deprecate InputFormatBase. Also make LineRecordReader easier to
    extend.  
    (Runping Qi via cutting)
53. HADOOP-1213. Improve logging of errors by IPC server, to
    consistently include the service name and the call.  
    (cutting)
54. HADOOP-1238. Fix metrics reporting by TaskTracker to correctly
    track maps\_running and reduces\_running.  
    (Michael Bieniosek via cutting)
55. HADOOP-1093. Fix a race condition in HDFS where blocks were
    sometimes erased before they were reported written.  
    (Dhruba Borthakur via cutting)
56. HADOOP-1239. Add a package name to some testjar test classes.  
    (Jim Kellerman via cutting)
57. HADOOP-1241. Fix NullPointerException in processReport when
    namenode is restarted.  
    (Dhruba Borthakur via tomwhite)
58. HADOOP-1244. Fix stop-dfs.sh to no longer incorrectly specify
    slaves file for stopping datanode.  
    (Michael Bieniosek via tomwhite)
59. HADOOP-1253. Fix ConcurrentModificationException and
    NullPointerException in JobControl.  
    (Johan Oskarson via tomwhite)
60. HADOOP-1256. Fix NameNode so that multiple DataNodeDescriptors
    can no longer be created on startup.  
    (Hairong Kuang via cutting)
61. HADOOP-1214. Replace streaming classes with new counterparts
    from Hadoop core.  
    (Runping Qi via tomwhite)
62. HADOOP-1250. Move a chmod utility from streaming to FileUtil.  
    (omalley via cutting)
63. HADOOP-1258. Fix TestCheckpoint test case to wait for
    MiniDFSCluster to be active.  
    (Nigel Daley via tomwhite)
64. HADOOP-1148. Re-indent all Java source code to consistently use
    two spaces per indent level.  
    (cutting)
65. HADOOP-1251. Add a method to Reporter to get the map InputSplit.  
    (omalley via cutting)
66. HADOOP-1224. Fix "Browse the filesystem" link to no longer point
    to dead datanodes.  
    (Enis Soztutar via tomwhite)
67. HADOOP-1154. Fail a streaming task if the threads reading from or
    writing to the streaming process fail.  
    (Koji Noguchi via tomwhite)
68. HADOOP-968. Move shuffle and sort to run in reduce's child JVM,
    rather than in TaskTracker.  
    (Devaraj Das via cutting)
69. HADOOP-1111. Add support for client notification of job
    completion. If the job configuration has a job.end.notification.url
    property it will make a HTTP GET request to the specified URL.
    The number of retries and the interval between retries is also
    configurable.  
    (Alejandro Abdelnur via tomwhite)
70. HADOOP-1275. Fix misspelled job notification property in
    hadoop-default.xml.  
    (Alejandro Abdelnur via tomwhite)
71. HADOOP-1152. Fix race condition in MapOutputCopier.copyOutput file
    rename causing possible reduce task hang.  
    (Tahir Hashmi via tomwhite)
72. HADOOP-1050. Distinguish between failed and killed tasks so as to
    not count a lost tasktracker against the job.  
    (Arun C Murthy via tomwhite)
73. HADOOP-1271. Fix StreamBaseRecordReader to be able to log record
    data that's not UTF-8.  
    (Arun C Murthy via tomwhite)
74. HADOOP-1190. Fix unchecked warnings in main Hadoop code.  
    (tomwhite)
75. HADOOP-1127. Fix AlreadyBeingCreatedException in namenode for
    jobs run with speculative execution.  
    (Arun C Murthy via tomwhite)
76. HADOOP-1282. Omnibus HBase patch. Improved tests & configuration.  
    (Jim Kellerman via cutting)
77. HADOOP-1262. Make dfs client try to read from a different replica
    of the checksum file when a checksum error is detected.  
    (Hairong Kuang via tomwhite)
78. HADOOP-1279. Fix JobTracker to maintain list of recently
    completed jobs by order of completion, not submission.  
    (Arun C Murthy via cutting)
79. HADOOP-1284. In contrib/streaming, permit flexible specification
    of field delimiter and fields for partitioning and sorting.  
    (Runping Qi via cutting)
80. HADOOP-1176. Fix a bug where reduce would hang when a map had
    more than 2GB of output for it.  
    (Arun C Murthy via cutting)
81. HADOOP-1293. Fix contrib/streaming to print more than the first
    twenty lines of standard error.  
    (Koji Noguchi via cutting)
82. HADOOP-1297. Fix datanode so that requests to remove blocks that
    do not exist no longer causes block reports to be re-sent every
    second.  
    (Dhruba Borthakur via cutting)
83. HADOOP-1216. Change MapReduce so that, when numReduceTasks is
    zero, map outputs are written directly as final output, skipping
    shuffle, sort and reduce. Use this to implement reduce=NONE
    option in contrib/streaming.  
    (Runping Qi via cutting)
84. HADOOP-1294. Fix unchecked warnings in main Hadoop code under
    Java 6.  
    (tomwhite)
85. HADOOP-1299. Fix so that RPC will restart after RPC.stopClient()
    has been called.  
    (Michael Stack via cutting)
86. HADOOP-1278. Improve blacklisting of TaskTrackers by JobTracker,
    to reduce false positives.  
    (Arun C Murthy via cutting)
87. HADOOP-1290. Move contrib/abacus into mapred/lib/aggregate.  
    (Runping Qi via cutting)
88. HADOOP-1272. Extract inner classes from FSNamesystem into separate
    classes.  
    (Dhruba Borthakur via tomwhite)
89. HADOOP-1247. Add support to contrib/streaming for aggregate
    package, formerly called Abacus.  
    (Runping Qi via cutting)
90. HADOOP-1061. Fix bug in listing files in the S3 filesystem.
    NOTE: this change is not backwards compatible! You should use the
    MigrationTool supplied to migrate existing S3 filesystem data to
    the new format. Please backup your data first before upgrading
    (using 'hadoop distcp' for example).  
    (tomwhite)
91. HADOOP-1304. Make configurable the maximum number of task
    attempts before a job fails.  
    (Devaraj Das via cutting)
92. HADOOP-1308. Use generics to restrict types when classes are
    passed as parameters to JobConf methods.  
    (Michael Bieniosek via cutting)
93. HADOOP-1312. Fix a ConcurrentModificationException in NameNode
    that killed the heartbeat monitoring thread.  
    (Dhruba Borthakur via cutting)
94. HADOOP-1315. Clean up contrib/streaming, switching it to use core
    classes more and removing unused code.  
    (Runping Qi via cutting)
95. HADOOP-485. Allow a different comparator for grouping keys in
    calls to reduce.  
    (Tahir Hashmi via cutting)
96. HADOOP-1322. Fix TaskTracker blacklisting to work correctly in
    one- and two-node clusters.  
    (Arun C Murthy via cutting)
97. HADOOP-1144. Permit one to specify a maximum percentage of tasks
    that can fail before a job is aborted. The default is zero.  
    (Arun C Murthy via cutting)
98. HADOOP-1184. Fix HDFS decomissioning to complete when the only
    copy of a block is on a decommissioned node.  
    (Dhruba Borthakur via cutting)
99. HADOOP-1263. Change DFSClient to retry certain namenode calls
    with a random, exponentially increasing backoff time, to avoid
    overloading the namenode on, e.g., job start.  
    (Hairong Kuang via cutting)
100. HADOOP-1325. First complete, functioning version of HBase.  
     (Jim Kellerman via cutting)
101. HADOOP-1276. Make tasktracker expiry interval configurable.  
     (Arun C Murthy via cutting)
102. HADOOP-1326. Change JobClient#RunJob() to return the job.  
     (omalley via cutting)
103. HADOOP-1270. Randomize the fetch of map outputs, speeding the
     shuffle.  
     (Arun C Murthy via cutting)
104. HADOOP-1200. Restore disk checking lost in HADOOP-1170.  
     (Hairong Kuang via cutting)
105. HADOOP-1252. Changed MapReduce's allocation of local files to
     use round-robin among available devices, rather than a hashcode.
     More care is also taken to not allocate files on full or offline
     drives.  
     (Devaraj Das via cutting)
106. HADOOP-1324. Change so that an FSError kills only the task that
     generates it rather than the entire task tracker.  
     (Arun C Murthy via cutting)
107. HADOOP-1310. Fix unchecked warnings in aggregate code.  
     (tomwhite)
108. HADOOP-1255. Fix a bug where the namenode falls into an infinite
     loop trying to remove a dead node.  
     (Hairong Kuang via cutting)
109. HADOOP-1160. Fix DistributedFileSystem.close() to close the
     underlying FileSystem, correctly aborting files being written.  
     (Hairong Kuang via cutting)
110. HADOOP-1341. Fix intermittent failures in HBase unit tests
     caused by deadlock.  
     (Jim Kellerman via cutting)
111. HADOOP-1350. Fix shuffle performance problem caused by forcing
     chunked encoding of map outputs.  
     (Devaraj Das via cutting)
112. HADOOP-1345. Fix HDFS to correctly retry another replica when a
     checksum error is encountered.  
     (Hairong Kuang via cutting)
113. HADOOP-1205. Improve synchronization around HDFS block map.  
     (Hairong Kuang via cutting)
114. HADOOP-1353. Fix a potential NullPointerException in namenode.  
     (Dhruba Borthakur via cutting)
115. HADOOP-1354. Fix a potential NullPointerException in FsShell.  
     (Hairong Kuang via cutting)
116. HADOOP-1358. Fix a potential bug when DFSClient calls skipBytes.  
     (Hairong Kuang via cutting)
117. HADOOP-1356. Fix a bug in ValueHistogram.  
     (Runping Qi via cutting)
118. HADOOP-1363. Fix locking bug in JobClient#waitForCompletion().  
     (omalley via cutting)
119. HADOOP-1368. Fix inconsistent synchronization in JobInProgress.  
     (omalley via cutting)
120. HADOOP-1369. Fix inconsistent synchronization in TaskTracker.  
     (omalley via cutting)
121. HADOOP-1361. Fix various calls to skipBytes() to check return
     value.  
     (Hairong Kuang via cutting)
122. HADOOP-1388. Fix a potential NullPointerException in web ui.  
     (Devaraj Das via cutting)
123. HADOOP-1385. Fix MD5Hash#hashCode() to generally hash to more
     than 256 values.  
     (omalley via cutting)
124. HADOOP-1386. Fix Path to not permit the empty string as a
     path, as this has lead to accidental file deletion. Instead
     force applications to use "." to name the default directory.  
     (Hairong Kuang via cutting)
125. HADOOP-1407. Fix integer division bug in JobInProgress which
     meant failed tasks didn't cause the job to fail.  
     (Arun C Murthy via tomwhite)
126. HADOOP-1427. Fix a typo that caused GzipCodec to incorrectly use
     a very small input buffer.  
     (Espen Amble Kolstad via cutting)
127. HADOOP-1435. Fix globbing code to no longer use the empty string
     to indicate the default directory, per HADOOP-1386.  
     (Hairong Kuang via cutting)
128. HADOOP-1411. Make task retry framework handle
     AlreadyBeingCreatedException when wrapped as a RemoteException.  
     (Hairong Kuang via tomwhite)
129. HADOOP-1242. Improve handling of DFS upgrades.  
     (Konstantin Shvachko via cutting)
130. HADOOP-1332. Fix so that TaskTracker exits reliably during unit
     tests on Windows.  
     (omalley via cutting)
131. HADOOP-1431. Fix so that sort progress reporting during map runs
     only while sorting, so that stuck maps are correctly terminated.  
     (Devaraj Das and Arun C Murthy via cutting)
132. HADOOP-1452. Change TaskTracker.MapOutputServlet.doGet.totalRead
     to a long, permitting map outputs to exceed 2^31 bytes.  
     (omalley via cutting)
133. HADOOP-1443. Fix a bug opening zero-length files in HDFS.  
     (Konstantin Shvachko via cutting)

### Release 0.12.3 - 2007-04-06

1. HADOOP-1162. Fix bug in record CSV and XML serialization of
   binary values.  
   (Milind Bhandarkar via cutting)
2. HADOOP-1123. Fix NullPointerException in LocalFileSystem when
   trying to recover from a checksum error.  
   (Hairong Kuang & Nigel Daley via tomwhite)
3. HADOOP-1177. Fix bug where IOException in MapOutputLocation.getFile
   was not being logged.  
   (Devaraj Das via tomwhite)
4. HADOOP-1175. Fix bugs in JSP for displaying a task's log messages.  
   (Arun C Murthy via cutting)
5. HADOOP-1191. Fix map tasks to wait until sort progress thread has
   stopped before reporting the task done.  
   (Devaraj Das via cutting)
6. HADOOP-1192. Fix an integer overflow bug in FSShell's 'dus'
   command and a performance problem in HDFS's implementation of it.  
   (Hairong Kuang via cutting)
7. HADOOP-1105. Fix reducers to make "progress" while iterating
   through values.  
   (Devaraj Das & Owen O'Malley via tomwhite)
8. HADOOP-1179. Make Task Tracker close index file as soon as the read
   is done when serving get-map-output requests.  
   (Devaraj Das via tomwhite)

### Release 0.12.2 - 2007-23-17

1. HADOOP-1135. Fix bug in block report processing which may cause
   the namenode to delete blocks.  
   (Dhruba Borthakur via tomwhite)
2. HADOOP-1145. Make XML serializer and deserializer classes public
   in record package.  
   (Milind Bhandarkar via cutting)
3. HADOOP-1140. Fix a deadlock in metrics.  
   (David Bowen via cutting)
4. HADOOP-1150. Fix streaming -reducer and -mapper to give them
   defaults.  
   (Owen O'Malley via tomwhite)

### Release 0.12.1 - 2007-03-17

1. HADOOP-1035. Fix a StackOverflowError in FSDataSet.  
   (Raghu Angadi via cutting)
2. HADOOP-1053. Fix VInt representation of negative values. Also
   remove references in generated record code to methods outside of
   the record package and improve some record documentation.  
   (Milind Bhandarkar via cutting)
3. HADOOP-1067. Compile fails if Checkstyle jar is present in lib
   directory. Also remove dependency on a particular Checkstyle
   version number.  
   (tomwhite)
4. HADOOP-1060. Fix an IndexOutOfBoundsException in the JobTracker
   that could cause jobs to hang.  
   (Arun C Murthy via cutting)
5. HADOOP-1077. Fix a race condition fetching map outputs that could
   hang reduces.  
   (Devaraj Das via cutting)
6. HADOOP-1083. Fix so that when a cluster restarts with a missing
   datanode, its blocks are replicated.  
   (Hairong Kuang via cutting)
7. HADOOP-1082. Fix a NullPointerException in ChecksumFileSystem.  
   (Hairong Kuang via cutting)
8. HADOOP-1088. Fix record serialization of negative values.  
   (Milind Bhandarkar via cutting)
9. HADOOP-1080. Fix bug in bin/hadoop on Windows when native
   libraries are present.  
   (ab via cutting)
10. HADOOP-1091. Fix a NullPointerException in MetricsRecord.  
    (David Bowen via tomwhite)
11. HADOOP-1092. Fix a NullPointerException in HeartbeatMonitor
    thread.  
    (Hairong Kuang via tomwhite)
12. HADOOP-1112. Fix a race condition in Hadoop metrics.  
    (David Bowen via tomwhite)
13. HADOOP-1108. Checksummed file system should retry reading if a
    different replica is found when handling ChecksumException.  
    (Hairong Kuang via tomwhite)
14. HADOOP-1070. Fix a problem with number of racks and datanodes
    temporarily doubling.  
    (Konstantin Shvachko via tomwhite)
15. HADOOP-1099. Fix NullPointerException in JobInProgress.  
    (Gautam Kowshik via tomwhite)
16. HADOOP-1115. Fix bug where FsShell copyToLocal doesn't
    copy directories.  
    (Hairong Kuang via tomwhite)
17. HADOOP-1109. Fix NullPointerException in StreamInputFormat.  
    (Koji Noguchi via tomwhite)
18. HADOOP-1117. Fix DFS scalability: when the namenode is
    restarted it consumes 80% CPU.  
    (Dhruba Borthakur via
    tomwhite)
19. HADOOP-1089. Make the C++ version of write and read v-int
    agree with the Java versions.  
    (Milind Bhandarkar via
    tomwhite)
20. HADOOP-1096. Rename InputArchive and OutputArchive and
    make them public.  
    (Milind Bhandarkar via tomwhite)
21. HADOOP-1128. Fix missing progress information in map tasks.  
    (Espen Amble Kolstad, Andrzej Bialecki, and Owen O'Malley
    via tomwhite)
22. HADOOP-1129. Fix DFSClient to not hide IOExceptions in
    flush method.  
    (Hairong Kuang via tomwhite)
23. HADOOP-1126. Optimize CPU usage for under replicated blocks
    when cluster restarts.  
    (Hairong Kuang via tomwhite)

### Release 0.12.0 - 2007-03-02

1. HADOOP-975. Separate stdout and stderr from tasks.  
   (Arun C Murthy via cutting)
2. HADOOP-982. Add some setters and a toString() method to
   BytesWritable.  
   (omalley via cutting)
3. HADOOP-858. Move contrib/smallJobsBenchmark to src/test, removing
   obsolete bits.  
   (Nigel Daley via cutting)
4. HADOOP-992. Fix MiniMR unit tests to use MiniDFS when specified,
   rather than the local FS.  
   (omalley via cutting)
5. HADOOP-954. Change use of metrics to use callback mechanism.
   Also rename utility class Metrics to MetricsUtil.  
   (David Bowen & Nigel Daley via cutting)
6. HADOOP-893. Improve HDFS client's handling of dead datanodes.
   The set is no longer reset with each block, but rather is now
   maintained for the life of an open file.  
   (Raghu Angadi via cutting)
7. HADOOP-882. Upgrade to jets3t version 0.5, used by the S3
   FileSystem. This version supports retries.  
   (Michael Stack via cutting)
8. HADOOP-977. Send task's stdout and stderr to JobClient's stdout
   and stderr respectively, with each line tagged by the task's name.  
   (Arun C Murthy via cutting)
9. HADOOP-761. Change unit tests to not use /tmp.  
   (Nigel Daley via cutting)
10. HADOOP-1007. Make names of metrics used in Hadoop unique.  
    (Nigel Daley via cutting)
11. HADOOP-491. Change mapred.task.timeout to be per-job, and make a
    value of zero mean no timeout. Also change contrib/streaming to
    disable task timeouts.  
    (Arun C Murthy via cutting)
12. HADOOP-1010. Add Reporter.NULL, a Reporter implementation that
    does nothing.  
    (Runping Qi via cutting)
13. HADOOP-923. In HDFS NameNode, move replication computation to a
    separate thread, to improve heartbeat processing time.  
    (Dhruba Borthakur via cutting)
14. HADOOP-476. Rewrite contrib/streaming command-line processing,
    improving parameter validation.  
    (Sanjay Dahiya via cutting)
15. HADOOP-973. Improve error messages in Namenode. This should help
    to track down a problem that was appearing as a
    NullPointerException.  
    (Dhruba Borthakur via cutting)
16. HADOOP-649. Fix so that jobs with no tasks are not lost.  
    (Thomas Friol via cutting)
17. HADOOP-803. Reduce memory use by HDFS namenode, phase I.  
    (Raghu Angadi via cutting)
18. HADOOP-1021. Fix MRCaching-based unit tests on Windows.  
    (Nigel Daley via cutting)
19. HADOOP-889. Remove duplicate code from HDFS unit tests.  
    (Milind Bhandarkar via cutting)
20. HADOOP-943. Improve HDFS's fsck command to display the filename
    for under-replicated blocks.  
    (Dhruba Borthakur via cutting)
21. HADOOP-333. Add validator for sort benchmark output.  
    (Arun C Murthy via cutting)
22. HADOOP-947. Improve performance of datanode decomissioning.  
    (Dhruba Borthakur via cutting)
23. HADOOP-442. Permit one to specify hosts allowed to connect to
    namenode and jobtracker with include and exclude files.  
    (Wendy
    Chien via cutting)
24. HADOOP-1017. Cache constructors, for improved performance.  
    (Ron Bodkin via cutting)
25. HADOOP-867. Move split creation out of JobTracker to client.
    Splits are now saved in a separate file, read by task processes
    directly, so that user code is no longer required in the
    JobTracker.  
    (omalley via cutting)
26. HADOOP-1006. Remove obsolete '-local' option from test code.  
    (Gautam Kowshik via cutting)
27. HADOOP-952. Create a public (shared) Hadoop EC2 AMI.
    The EC2 scripts now support launch of public AMIs.  
    (tomwhite)
28. HADOOP-1025. Remove some obsolete code in ipc.Server.  
    (cutting)
29. HADOOP-997. Implement S3 retry mechanism for failed block
    transfers. This includes a generic retry mechanism for use
    elsewhere in Hadoop.  
    (tomwhite)
30. HADOOP-990. Improve HDFS support for full datanode volumes.  
    (Raghu Angadi via cutting)
31. HADOOP-564. Replace uses of "dfs://" URIs with the more standard
    "hdfs://".  
    (Wendy Chien via cutting)
32. HADOOP-1030. In unit tests, unify setting of ipc.client.timeout.
    Also increase the value used from one to two seconds, in hopes of
    making tests complete more reliably.  
    (cutting)
33. HADOOP-654. Stop assigning tasks to a tasktracker if it has
    failed more than a specified number in the job.  
    (Arun C Murthy via cutting)
34. HADOOP-985. Change HDFS to identify nodes by IP address rather
    than by DNS hostname.  
    (Raghu Angadi via cutting)
35. HADOOP-248. Optimize location of map outputs to not use random
    probes.  
    (Devaraj Das via cutting)
36. HADOOP-1029. Fix streaming's input format to correctly seek to
    the start of splits.  
    (Arun C Murthy via cutting)
37. HADOOP-492. Add per-job and per-task counters. These are
    incremented via the Reporter interface and available through the
    web ui and the JobClient API. The mapreduce framework maintains a
    few basic counters, and applications may add their own. Counters
    are also passed to the metrics system.  
    (David Bowen via cutting)
38. HADOOP-1034. Fix datanode to better log exceptions.  
    (Philippe Gassmann via cutting)
39. HADOOP-878. In contrib/streaming, fix reducer=NONE to work with
    multiple maps.  
    (Arun C Murthy via cutting)
40. HADOOP-1039. In HDFS's TestCheckpoint, avoid restarting
    MiniDFSCluster so often, speeding this test.  
    (Dhruba Borthakur via cutting)
41. HADOOP-1040. Update RandomWriter example to use counters and
    user-defined input and output formats.  
    (omalley via cutting)
42. HADOOP-1027. Fix problems with in-memory merging during shuffle
    and re-enable this optimization.  
    (Devaraj Das via cutting)
43. HADOOP-1036. Fix exception handling in TaskTracker to keep tasks
    from being lost.  
    (Arun C Murthy via cutting)
44. HADOOP-1042. Improve the handling of failed map output fetches.  
    (Devaraj Das via cutting)
45. HADOOP-928. Make checksums optional per FileSystem.  
    (Hairong Kuang via cutting)
46. HADOOP-1044. Fix HDFS's TestDecommission to not spuriously fail.  
    (Wendy Chien via cutting)
47. HADOOP-972. Optimize HDFS's rack-aware block placement algorithm.  
    (Hairong Kuang via cutting)
48. HADOOP-1043. Optimize shuffle, increasing parallelism.  
    (Devaraj Das via cutting)
49. HADOOP-940. Improve HDFS's replication scheduling.  
    (Dhruba Borthakur via cutting)
50. HADOOP-1020. Fix a bug in Path resolution, and a with unit tests
    on Windows.  
    (cutting)
51. HADOOP-941. Enhance record facility.  
    (Milind Bhandarkar via cutting)
52. HADOOP-1000. Fix so that log messages in task subprocesses are
    not written to a task's standard error.  
    (Arun C Murthy via cutting)
53. HADOOP-1037. Fix bin/slaves.sh, which currently only works with
    /bin/bash, to specify /bin/bash rather than /bin/sh.  
    (cutting)
54. HADOOP-1046. Clean up tmp from partially received stale block files.  
    (ab)
55. HADOOP-1041. Optimize mapred counter implementation. Also group
    counters by their declaring Enum.  
    (David Bowen via cutting)
56. HADOOP-1032. Permit one to specify jars that will be cached
    across multiple jobs.  
    (Gautam Kowshik via cutting)
57. HADOOP-1051. Add optional checkstyle task to build.xml. To use
    this developers must download the (LGPL'd) checkstyle jar
    themselves.  
    (tomwhite via cutting)
58. HADOOP-1049. Fix a race condition in IPC client.  
    (Devaraj Das via cutting)
59. HADOOP-1056. Check HDFS include/exclude node lists with both IP
    address and hostname.  
    (Wendy Chien via cutting)
60. HADOOP-994. In HDFS, limit the number of blocks invalidated at
    once. Large lists were causing datenodes to timeout.  
    (Dhruba Borthakur via cutting)
61. HADOOP-432. Add a trash feature, disabled by default. When
    enabled, the FSShell 'rm' command will move things to a trash
    directory in the filesystem. In HDFS, a thread periodically
    checkpoints the trash and removes old checkpoints.  
    (cutting)

### Release 0.11.2 - 2007-02-16

1. HADOOP-1009. Fix an infinite loop in the HDFS namenode.  
   (Dhruba Borthakur via cutting)
2. HADOOP-1014. Disable in-memory merging during shuffle, as this is
   causing data corruption.  
   (Devaraj Das via cutting)

### Release 0.11.1 - 2007-02-09

1. HADOOP-976. Make SequenceFile.Metadata public.  
   (Runping Qi via cutting)
2. HADOOP-917. Fix a NullPointerException in SequenceFile's merger
   with large map outputs.  
   (omalley via cutting)
3. HADOOP-984. Fix a bug in shuffle error handling introduced by
   HADOOP-331. If a map output is unavailable, the job tracker is
   once more informed.  
   (Arun C Murthy via cutting)
4. HADOOP-987. Fix a problem in HDFS where blocks were not removed
   from neededReplications after a replication target was selected.  
   (Hairong Kuang via cutting)

### Release 0.11.0 - 2007-02-02

1. HADOOP-781. Remove methods deprecated in 0.10 that are no longer
   widely used.  
   (cutting)
2. HADOOP-842. Change HDFS protocol so that the open() method is
   passed the client hostname, to permit the namenode to order block
   locations on the basis of network topology.  
   (Hairong Kuang via cutting)
3. HADOOP-852. Add an ant task to compile record definitions, and
   use it to compile record unit tests.  
   (Milind Bhandarkar via cutting)
4. HADOOP-757. Fix "Bad File Descriptor" exception in HDFS client
   when an output file is closed twice.  
   (Raghu Angadi via cutting)
5. [ intentionally blank ]
6. HADOOP-890. Replace dashes in metric names with underscores,
   for better compatibility with some monitoring systems.  
   (Nigel Daley via cutting)
7. HADOOP-801. Add to jobtracker a log of task completion events.  
   (Sanjay Dahiya via cutting)
8. HADOOP-855. In HDFS, try to repair files with checksum errors.
   An exception is still thrown, but corrupt blocks are now removed
   when they have replicas.  
   (Wendy Chien via cutting)
9. HADOOP-886. Reduce number of timer threads created by metrics API
   by pooling contexts.  
   (Nigel Daley via cutting)
10. HADOOP-897. Add a "javac.args" property to build.xml that permits
    one to pass arbitrary options to javac.  
    (Milind Bhandarkar via cutting)
11. HADOOP-899. Update libhdfs for changes in HADOOP-871.  
    (Sameer Paranjpye via cutting)
12. HADOOP-905. Remove some dead code from JobClient.  
    (cutting)
13. HADOOP-902. Fix a NullPointerException in HDFS client when
    closing output streams.  
    (Raghu Angadi via cutting)
14. HADOOP-735. Switch generated record code to use BytesWritable to
    represent fields of type 'buffer'.  
    (Milind Bhandarkar via cutting)
15. HADOOP-830. Improve mapreduce merge performance by buffering and
    merging multiple map outputs as they arrive at reduce nodes before
    they're written to disk.  
    (Devaraj Das via cutting)
16. HADOOP-908. Add a new contrib package, Abacus, that simplifies
    counting and aggregation, built on MapReduce.  
    (Runping Qi via cutting)
17. HADOOP-901. Add support for recursive renaming to the S3 filesystem.  
    (Tom White via cutting)
18. HADOOP-912. Fix a bug in TaskTracker.isIdle() that was
    sporadically causing unit test failures.  
    (Arun C Murthy via cutting)
19. HADOOP-909. Fix the 'du' command to correctly compute the size of
    FileSystem directory trees.  
    (Hairong Kuang via cutting)
20. HADOOP-731. When a checksum error is encountered on a file stored
    in HDFS, try another replica of the data, if any.  
    (Wendy Chien via cutting)
21. HADOOP-732. Add support to SequenceFile for arbitrary metadata,
    as a set of attribute value pairs.  
    (Runping Qi via cutting)
22. HADOOP-929. Fix PhasedFileSystem to pass configuration to
    underlying FileSystem.  
    (Sanjay Dahiya via cutting)
23. HADOOP-935. Fix contrib/abacus to not delete pre-existing output
    files, but rather to fail in this case.  
    (Runping Qi via cutting)
24. HADOOP-936. More metric renamings, as in HADOOP-890.  
    (Nigel Daley via cutting)
25. HADOOP-856. Fix HDFS's fsck command to not report that
    non-existent filesystems are healthy.  
    (Milind Bhandarkar via cutting)
26. HADOOP-602. Remove the dependency on Lucene's PriorityQueue
    utility, by copying it into Hadoop. This facilitates using Hadoop
    with different versions of Lucene without worrying about CLASSPATH
    order.  
    (Milind Bhandarkar via cutting)
27. [ intentionally blank ]
28. HADOOP-227. Add support for backup namenodes, which periodically
    get snapshots of the namenode state.  
    (Dhruba Borthakur via cutting)
29. HADOOP-884. Add scripts in contrib/ec2 to facilitate running
    Hadoop on an Amazon's EC2 cluster.  
    (Tom White via cutting)
30. HADOOP-937. Change the namenode to request re-registration of
    datanodes in more circumstances.  
    (Hairong Kuang via cutting)
31. HADOOP-922. Optimize small forward seeks in HDFS. If data is has
    likely already in flight, skip ahead rather than re-opening the
    block.  
    (Dhruba Borthakur via cutting)
32. HADOOP-961. Add a 'job -events' sub-command that prints job
    events, including task completions and failures.  
    (omalley via cutting)
33. HADOOP-959. Fix namenode snapshot code added in HADOOP-227 to
    work on Windows.  
    (Dhruba Borthakur via cutting)
34. HADOOP-934. Fix TaskTracker to catch metrics exceptions that were
    causing heartbeats to fail.  
    (Arun Murthy via cutting)
35. HADOOP-881. Fix JobTracker web interface to display the correct
    number of task failures.  
    (Sanjay Dahiya via cutting)
36. HADOOP-788. Change contrib/streaming to subclass TextInputFormat,
    permitting it to take advantage of native compression facilities.  
    (Sanjay Dahiya via cutting)
37. HADOOP-962. In contrib/ec2: make scripts executable in tar file;
    add a README; make the environment file use a template.  
    (Tom White via cutting)
38. HADOOP-549. Fix a NullPointerException in TaskReport's
    serialization.  
    (omalley via cutting)
39. HADOOP-963. Fix remote exceptions to have the stack trace of the
    caller thread, not the IPC listener thread.  
    (omalley via cutting)
40. HADOOP-967. Change RPC clients to start sending a version header.  
    (omalley via cutting)
41. HADOOP-964. Fix a bug introduced by HADOOP-830 where jobs failed
    whose comparators and/or i/o types were in the job's jar.  
    (Dennis Kubes via cutting)
42. HADOOP-969. Fix a deadlock in JobTracker.  
    (omalley via cutting)
43. HADOOP-862. Add support for the S3 FileSystem to the CopyFiles
    tool.  
    (Michael Stack via cutting)
44. HADOOP-965. Fix IsolationRunner so that job's jar can be found.  
    (Dennis Kubes via cutting)
45. HADOOP-309. Fix two NullPointerExceptions in StatusHttpServer.  
    (navychen via cutting)
46. HADOOP-692. Add rack awareness to HDFS's placement of blocks.  
    (Hairong Kuang via cutting)

### Release 0.10.1 - 2007-01-10

1. HADOOP-857. Fix S3 FileSystem implementation to permit its use
   for MapReduce input and output.  
   (Tom White via cutting)
2. HADOOP-863. Reduce logging verbosity introduced by HADOOP-813.  
   (Devaraj Das via cutting)
3. HADOOP-815. Fix memory leaks in JobTracker.  
   (Arun C Murthy via cutting)
4. HADOOP-600. Fix a race condition in JobTracker.  
   (Arun C Murthy via cutting)
5. HADOOP-864. Fix 'bin/hadoop -jar' to operate correctly when
   hadoop.tmp.dir does not yet exist.  
   (omalley via cutting)
6. HADOOP-866. Fix 'dfs -get' command to remove existing crc files,
   if any.  
   (Milind Bhandarkar via cutting)
7. HADOOP-871. Fix a bug in bin/hadoop setting JAVA\_LIBRARY\_PATH.  
   (Arun C Murthy via cutting)
8. HADOOP-868. Decrease the number of open files during map,
   respecting io.sort.fa ctor.  
   (Devaraj Das via cutting)
9. HADOOP-865. Fix S3 FileSystem so that partially created files can
   be deleted.  
   (Tom White via cutting)
10. HADOOP-873. Pass java.library.path correctly to child processes.  
    (omalley via cutting)
11. HADOOP-851. Add support for the LZO codec. This is much faster
    than the default, zlib-based compression, but it is only available
    when the native library is built.  
    (Arun C Murthy via cutting)
12. HADOOP-880. Fix S3 FileSystem to remove directories.  
    (Tom White via cutting)
13. HADOOP-879. Fix InputFormatBase to handle output generated by
    MapFileOutputFormat.  
    (cutting)
14. HADOOP-659. In HDFS, prioritize replication of blocks based on
    current replication level. Blocks which are severely
    under-replicated should be further replicated before blocks which
    are less under-replicated.  
    (Hairong Kuang via cutting)
15. HADOOP-726. Deprecate FileSystem locking methods. They are not
    currently usable. Locking should eventually provided as an
    independent service.  
    (Raghu Angadi via cutting)
16. HADOOP-758. Fix exception handling during reduce so that root
    exceptions are not masked by exceptions in cleanups.  
    (Raghu Angadi via cutting)

### Release 0.10.0 - 2007-01-05

1. HADOOP-763. Change DFS namenode benchmark to not use MapReduce.  
   (Nigel Daley via cutting)
2. HADOOP-777. Use fully-qualified hostnames for tasktrackers and
   datanodes.  
   (Mahadev Konar via cutting)
3. HADOOP-621. Change 'dfs -cat' to exit sooner when output has been
   closed.  
   (Dhruba Borthakur via cutting)
4. HADOOP-752. Rationalize some synchronization in DFS namenode.  
   (Dhruba Borthakur via cutting)
5. HADOOP-629. Fix RPC services to better check the protocol name and
   version.  
   (omalley via cutting)
6. HADOOP-774. Limit the number of invalid blocks returned with
   heartbeats by the namenode to datanodes. Transmitting and
   processing very large invalid block lists can tie up both the
   namenode and datanode for too long.  
   (Dhruba Borthakur via cutting)
7. HADOOP-738. Change 'dfs -get' command to not create CRC files by
   default, adding a -crc option to force their creation.  
   (Milind Bhandarkar via cutting)
8. HADOOP-676. Improved exceptions and error messages for common job
   input specification errors.  
   (Sanjay Dahiya via cutting)
9. [Included in 0.9.2 release]
10. HADOOP-756. Add new dfsadmin option to wait for filesystem to be
    operational.  
    (Dhruba Borthakur via cutting)
11. HADOOP-770. Fix jobtracker web interface to display, on restart,
    jobs that were running when it was last stopped.  
    (Sanjay Dahiya via cutting)
12. HADOOP-331. Write all map outputs to a single file with an index,
    rather than to a separate file per reduce task. This should both
    speed the shuffle and make things more scalable.  
    (Devaraj Das via cutting)
13. HADOOP-818. Fix contrib unit tests to not depend on core unit
    tests.  
    (omalley via cutting)
14. HADOOP-786. Log common exception at debug level.  
    (Sanjay Dahiya via cutting)
15. HADOOP-796. Provide more convenient access to failed task
    information in the web interface.  
    (Sanjay Dahiya via cutting)
16. HADOOP-764. Reduce memory allocations in namenode some.  
    (Dhruba Borthakur via cutting)
17. HADOOP-802. Update description of mapred.speculative.execution to
    mention reduces.  
    (Nigel Daley via cutting)
18. HADOOP-806. Include link to datanodes on front page of namenode
    web interface.  
    (Raghu Angadi via cutting)
19. HADOOP-618. Make JobSubmissionProtocol public.  
    (Arun C Murthy via cutting)
20. HADOOP-782. Fully remove killed tasks.  
    (Arun C Murthy via cutting)
21. HADOOP-792. Fix 'dfs -mv' to return correct status.  
    (Dhruba Borthakur via cutting)
22. HADOOP-673. Give each task its own working directory again.  
    (Mahadev Konar via cutting)
23. HADOOP-571. Extend the syntax of Path to be a URI; to be
    optionally qualified with a scheme and authority. The scheme
    determines the FileSystem implementation, while the authority
    determines the FileSystem instance. New FileSystem
    implementations may be provided by defining an fs.<scheme>.impl
    property, naming the FileSystem implementation class. This
    permits easy integration of new FileSystem implementations.  
    (cutting)
24. HADOOP-720. Add an HDFS white paper to website.  
    (Dhruba Borthakur via cutting)
25. HADOOP-794. Fix a divide-by-zero exception when a job specifies
    zero map tasks.  
    (omalley via cutting)
26. HADOOP-454. Add a 'dfs -dus' command that provides summary disk
    usage.  
    (Hairong Kuang via cutting)
27. HADOOP-574. Add an Amazon S3 implementation of FileSystem. To
    use this, one need only specify paths of the form
    s3://id:secret@bucket/. Alternately, the AWS access key id and
    secret can be specified in your config, with the properties
    fs.s3.awsAccessKeyId and fs.s3.awsSecretAccessKey.  
    (Tom White via cutting)
28. HADOOP-824. Rename DFSShell to be FsShell, since it applies
    generically to all FileSystem implementations.  
    (cutting)
29. HADOOP-813. Fix map output sorting to report progress, so that
    sorts which take longer than the task timeout do not fail.  
    (Devaraj Das via cutting)
30. HADOOP-825. Fix HDFS daemons when configured with new URI syntax.  
    (omalley via cutting)
31. HADOOP-596. Fix a bug in phase reporting during reduce.  
    (Sanjay Dahiya via cutting)
32. HADOOP-811. Add a utility, MultithreadedMapRunner.  
    (Alejandro Abdelnur via cutting)
33. HADOOP-829. Within HDFS, clearly separate three different
    representations for datanodes: one for RPCs, one for
    namenode-internal use, and one for namespace persistence.  
    (Dhruba Borthakur via cutting)
34. HADOOP-823. Fix problem starting datanode when not all configured
    data directories exist.  
    (Bryan Pendleton via cutting)
35. HADOOP-451. Add a Split interface. CAUTION: This incompatibly
    changes the InputFormat and RecordReader interfaces. Not only is
    FileSplit replaced with Split, but a FileSystem parameter is no
    longer passed in several methods, input validation has changed,
    etc.  
    (omalley via cutting)
36. HADOOP-814. Optimize locking in namenode.  
    (Dhruba Borthakur via cutting)
37. HADOOP-738. Change 'fs -put' and 'fs -get' commands to accept
    standard input and output, respectively. Standard i/o is
    specified by a file named '-'.  
    (Wendy Chien via cutting)
38. HADOOP-835. Fix a NullPointerException reading record-compressed
    SequenceFiles.  
    (Hairong Kuang via cutting)
39. HADOOP-836. Fix a MapReduce bug on Windows, where the wrong
    FileSystem was used. Also add a static FileSystem.getLocal()
    method and better Path checking in HDFS, to help avoid such issues
    in the future.  
    (omalley via cutting)
40. HADOOP-837. Improve RunJar utility to unpack jar file
    hadoop.tmp.dir, rather than the system temporary directory.  
    (Hairong Kuang via cutting)
41. HADOOP-841. Fix native library to build 32-bit version even when
    on a 64-bit host, if a 32-bit JVM is used.  
    (Arun C Murthy via cutting)
42. HADOOP-838. Fix tasktracker to pass java.library.path to
    sub-processes, so that libhadoop.a is found.  
    (Arun C Murthy via cutting)
43. HADOOP-844. Send metrics messages on a fixed-delay schedule
    instead of a fixed-rate schedule.  
    (David Bowen via cutting)
44. HADOOP-849. Fix OutOfMemory exceptions in TaskTracker due to a
    file handle leak in SequenceFile.  
    (Devaraj Das via cutting)
45. HADOOP-745. Fix a synchronization bug in the HDFS namenode.  
    (Dhruba Borthakur via cutting)
46. HADOOP-850. Add Writable implementations for variable-length
    integers.  
    (ab via cutting)
47. HADOOP-525. Add raw comparators to record types. This greatly
    improves record sort performance.  
    (Milind Bhandarkar via cutting)
48. HADOOP-628. Fix a problem with 'fs -cat' command, where some
    characters were replaced with question marks.  
    (Wendy Chien via cutting)
49. HADOOP-804. Reduce verbosity of MapReduce logging.  
    (Sanjay Dahiya via cutting)
50. HADOOP-853. Rename 'site' to 'docs', in preparation for inclusion
    in releases.  
    (cutting)
51. HADOOP-371. Include contrib jars and site documentation in
    distributions. Also add contrib and example documentation to
    distributed javadoc, in separate sections.  
    (Nigel Daley via cutting)
52. HADOOP-846. Report progress during entire map, as sorting of
    intermediate outputs may happen at any time, potentially causing
    task timeouts.  
    (Devaraj Das via cutting)
53. HADOOP-840. In task tracker, queue task cleanups and perform them
    in a separate thread.  
    (omalley & Mahadev Konar via cutting)
54. HADOOP-681. Add to HDFS the ability to decommission nodes. This
    causes their blocks to be re-replicated on other nodes, so that
    they may be removed from a cluster.  
    (Dhruba Borthakur via cutting)
55. HADOOP-470. In HDFS web ui, list the datanodes containing each
    copy of a block.  
    (Hairong Kuang via cutting)
56. HADOOP-700. Change bin/hadoop to only include core jar file on
    classpath, not example, test, etc. Also rename core jar to
    hadoop-${version}-core.jar so that it can be more easily
    identified.  
    (Nigel Daley via cutting)
57. HADOOP-619. Extend InputFormatBase to accept individual files and
    glob patterns as MapReduce inputs, not just directories. Also
    change contrib/streaming to use this.  
    (Sanjay Dahia via cutting)

### Release 0.9.2 - 2006-12-15

1. HADOOP-639. Restructure InterTrackerProtocol to make task
   accounting more reliable.  
   (Arun C Murthy via cutting)
2. HADOOP-827. Turn off speculative execution by default, since it's
   currently broken.  
   (omalley via cutting)
3. HADOOP-791. Fix a deadlock in the task tracker.  
   (Mahadev Konar via cutting)

### Release 0.9.1 - 2006-12-06

1. HADOOP-780. Use ReflectionUtils to instantiate key and value
   objects.  
   (ab)
2. HADOOP-779. Fix contrib/streaming to work correctly with gzipped
   input files.  
   (Hairong Kuang via cutting)

### Release 0.9.0 - 2006-12-01

1. HADOOP-655. Remove most deprecated code. A few deprecated things
   remain, notably UTF8 and some methods that are still required.
   Also cleaned up constructors for SequenceFile, MapFile, SetFile,
   and ArrayFile a bit.  
   (cutting)
2. HADOOP-565. Upgrade to Jetty version 6.  
   (Sanjay Dahiya via cutting)
3. HADOOP-682. Fix DFS format command to work correctly when
   configured with a non-existent directory.  
   (Sanjay Dahiya via cutting)
4. HADOOP-645. Fix a bug in contrib/streaming when -reducer is NONE.  
   (Dhruba Borthakur via cutting)
5. HADOOP-687. Fix a classpath bug in bin/hadoop that blocked the
   servers from starting.  
   (Sameer Paranjpye via omalley)
6. HADOOP-683. Remove a script dependency on bash, so it works with
   dash, the new default for /bin/sh on Ubuntu.  
   (James Todd via cutting)
7. HADOOP-382. Extend unit tests to run multiple datanodes.  
   (Milind Bhandarkar via cutting)
8. HADOOP-604. Fix some synchronization issues and a
   NullPointerException in DFS datanode.  
   (Raghu Angadi via cutting)
9. HADOOP-459. Fix memory leaks and a host of other issues with
   libhdfs.  
   (Sameer Paranjpye via cutting)
10. HADOOP-694. Fix a NullPointerException in jobtracker.  
    (Mahadev Konar via cutting)
11. HADOOP-637. Fix a memory leak in the IPC server. Direct buffers
    are not collected like normal buffers, and provided little
    advantage.  
    (Raghu Angadi via cutting)
12. HADOOP-696. Fix TestTextInputFormat unit test to not rely on the
    order of directory listings.  
    (Sameer Paranjpye via cutting)
13. HADOOP-611. Add support for iterator-based merging to
    SequenceFile.  
    (Devaraj Das via cutting)
14. HADOOP-688. Move DFS administrative commands to a separate
    command named 'dfsadmin'.  
    (Dhruba Borthakur via cutting)
15. HADOOP-708. Fix test-libhdfs to return the correct status, so
    that failures will break the build.  
    (Nigel Daley via cutting)
16. HADOOP-646. Fix namenode to handle edits files larger than 2GB.  
    (Milind Bhandarkar via cutting)
17. HADOOP-705. Fix a bug in the JobTracker when failed jobs were
    not completely cleaned up.  
    (Mahadev Konar via cutting)
18. HADOOP-613. Perform final merge while reducing. This removes one
    sort pass over the data and should consequently significantly
    decrease overall processing time.  
    (Devaraj Das via cutting)
19. HADOOP-661. Make each job's configuration visible through the web
    ui.  
    (Arun C Murthy via cutting)
20. HADOOP-489. In MapReduce, separate user logs from system logs.
    Each task's log output is now available through the web ui.  
    (Arun
    C Murthy via cutting)
21. HADOOP-712. Fix record io's xml serialization to correctly handle
    control-characters.  
    (Milind Bhandarkar via cutting)
22. HADOOP-668. Improvements to the web-based DFS browser.  
    (Hairong Kuang via cutting)
23. HADOOP-715. Fix build.xml so that test logs are written in build
    directory, rather than in CWD.  
    (Arun C Murthy via cutting)
24. HADOOP-538. Add support for building an optional native library,
    libhadoop.so, that improves the performance of zlib-based
    compression. To build this, specify -Dcompile.native to Ant.  
    (Arun C Murthy via cutting)
25. HADOOP-610. Fix an problem when the DFS block size is configured
    to be smaller than the buffer size, typically only when debugging.  
    (Milind Bhandarkar via cutting)
26. HADOOP-695. Fix a NullPointerException in contrib/streaming.  
    (Hairong Kuang via cutting)
27. HADOOP-652. In DFS, when a file is deleted, the block count is
    now decremented.  
    (Vladimir Krokhmalyov via cutting)
28. HADOOP-725. In DFS, optimize block placement algorithm,
    previously a performance bottleneck.  
    (Milind Bhandarkar via cutting)
29. HADOOP-723. In MapReduce, fix a race condition during the
    shuffle, which resulted in FileNotFoundExceptions.  
    (omalley via cutting)
30. HADOOP-447. In DFS, fix getBlockSize(Path) to work with relative
    paths.  
    (Raghu Angadi via cutting)
31. HADOOP-733. Make exit codes in DFShell consistent and add a unit
    test.  
    (Dhruba Borthakur via cutting)
32. HADOOP-709. Fix contrib/streaming to work with commands that
    contain control characters.  
    (Dhruba Borthakur via cutting)
33. HADOOP-677. In IPC, permit a version header to be transmitted
    when connections are established. This will permit us to change
    the format of IPC requests back-compatibly in subsequent releases.  
    (omalley via cutting)
34. HADOOP-699. Fix DFS web interface so that filesystem browsing
    works correctly, using the right port number. Also add support
    for sorting datanode list by various columns.  
    (Raghu Angadi via cutting)
35. HADOOP-76. Implement speculative reduce. Now when a job is
    configured for speculative execution, both maps and reduces will
    execute speculatively. Reduce outputs are written to temporary
    location and moved to the final location when reduce is complete.  
    (Sanjay Dahiya via cutting)
36. HADOOP-736. Roll back to Jetty 5.1.4, due to performance problems
    with Jetty 6.0.1.
37. HADOOP-739. Fix TestIPC to use different port number, making it
    more reliable.  
    (Nigel Daley via cutting)
38. HADOOP-749. Fix a NullPointerException in jobfailures.jsp.  
    (omalley via cutting)
39. HADOOP-747. Fix record serialization to work correctly when
    records are embedded in Maps.  
    (Milind Bhandarkar via cutting)
40. HADOOP-698. Fix HDFS client not to retry the same datanode on
    read failures.  
    (Milind Bhandarkar via cutting)
41. HADOOP-689. Add GenericWritable, to facilitate polymorphism in
    MapReduce, SequenceFile, etc.  
    (Feng Jiang via cutting)
42. HADOOP-430. Stop datanode's HTTP server when registration with
    namenode fails.  
    (Wendy Chien via cutting)
43. HADOOP-750. Fix a potential race condition during mapreduce
    shuffle.  
    (omalley via cutting)
44. HADOOP-728. Fix contrib/streaming-related issues, including
    '-reducer NONE'.  
    (Sanjay Dahiya via cutting)

### Release 0.8.0 - 2006-11-03

1. HADOOP-477. Extend contrib/streaming to scan the PATH environment
   variables when resolving executable program names.  
   (Dhruba Borthakur via cutting)
2. HADOOP-583. In DFSClient, reduce the log level of re-connect
   attempts from 'info' to 'debug', so they are not normally shown.  
   (Konstantin Shvachko via cutting)
3. HADOOP-498. Re-implement DFS integrity checker to run server-side,
   for much improved performance.  
   (Milind Bhandarkar via cutting)
4. HADOOP-586. Use the jar name for otherwise un-named jobs.  
   (Sanjay Dahiya via cutting)
5. HADOOP-514. Make DFS heartbeat interval configurable.  
   (Milind Bhandarkar via cutting)
6. HADOOP-588. Fix logging and accounting of failed tasks.  
   (Sanjay Dahiya via cutting)
7. HADOOP-462. Improve command line parsing in DFSShell, so that
   incorrect numbers of arguments result in informative errors rather
   than ArrayOutOfBoundsException.  
   (Dhruba Borthakur via cutting)
8. HADOOP-561. Fix DFS so that one replica of each block is written
   locally, if possible. This was the intent, but there as a bug.  
   (Dhruba Borthakur via cutting)
9. HADOOP-610. Fix TaskTracker to survive more exceptions, keeping
   tasks from becoming lost.  
   (omalley via cutting)
10. HADOOP-625. Add a servlet to all http daemons that displays a
    stack dump, useful for debugging.  
    (omalley via cutting)
11. HADOOP-554. Fix DFSShell to return -1 for errors.  
    (Dhruba Borthakur via cutting)
12. HADOOP-626. Correct the documentation in the NNBench example
    code, and also remove a mistaken call there.  
    (Nigel Daley via cutting)
13. HADOOP-634. Add missing license to many files.  
    (Nigel Daley via cutting)
14. HADOOP-627. Fix some synchronization problems in MiniMRCluster
    that sometimes caused unit tests to fail.  
    (Nigel Daley via cutting)
15. HADOOP-563. Improve the NameNode's lease policy so that leases
    are held for one hour without renewal (instead of one minute).
    However another attempt to create the same file will still succeed
    if the lease has not been renewed within a minute. This prevents
    communication or scheduling problems from causing a write to fail
    for up to an hour, barring some other process trying to create the
    same file.  
    (Dhruba Borthakur via cutting)
16. HADOOP-635. In DFSShell, permit specification of multiple files
    as the source for file copy and move commands.  
    (Dhruba Borthakur via cutting)
17. HADOOP-641. Change NameNode to request a fresh block report from
    a re-discovered DataNode, so that no-longer-needed replications
    are stopped promptly.  
    (Konstantin Shvachko via cutting)
18. HADOOP-642. Change IPC client to specify an explicit connect
    timeout.  
    (Konstantin Shvachko via cutting)
19. HADOOP-638. Fix an unsynchronized access to TaskTracker's
    internal state.  
    (Nigel Daley via cutting)
20. HADOOP-624. Fix servlet path to stop a Jetty warning on startup.  
    (omalley via cutting)
21. HADOOP-578. Failed tasks are no longer placed at the end of the
    task queue. This was originally done to work around other
    problems that have now been fixed. Re-executing failed tasks
    sooner causes buggy jobs to fail faster.  
    (Sanjay Dahiya via cutting)
22. HADOOP-658. Update source file headers per Apache policy.  
    (cutting)
23. HADOOP-636. Add MapFile & ArrayFile constructors which accept a
    Progressable, and pass it down to SequenceFile. This permits
    reduce tasks which use MapFile to still report progress while
    writing blocks to the filesystem.  
    (cutting)
24. HADOOP-576. Enable contrib/streaming to use the file cache. Also
    extend the cache to permit symbolic links to cached items, rather
    than local file copies.  
    (Mahadev Konar via cutting)
25. HADOOP-482. Fix unit tests to work when a cluster is running on
    the same machine, removing port conflicts.  
    (Wendy Chien via cutting)
26. HADOOP-90. Permit dfs.name.dir to list multiple directories,
    where namenode data is to be replicated.  
    (Milind Bhandarkar via cutting)
27. HADOOP-651. Fix DFSCk to correctly pass parameters to the servlet
    on the namenode.  
    (Milind Bhandarkar via cutting)
28. HADOOP-553. Change main() routines of DataNode and NameNode to
    log exceptions rather than letting the JVM print them to standard
    error. Also, change the hadoop-daemon.sh script to rotate
    standard i/o log files.  
    (Raghu Angadi via cutting)
29. HADOOP-399. Fix javadoc warnings.  
    (Nigel Daley via cutting)
30. HADOOP-599. Fix web ui and command line to correctly report DFS
    filesystem size statistics. Also improve web layout.  
    (Raghu Angadi via cutting)
31. HADOOP-660. Permit specification of junit test output format.  
    (Nigel Daley via cutting)
32. HADOOP-663. Fix a few unit test issues.  
    (Mahadev Konar via cutting)
33. HADOOP-664. Cause entire build to fail if libhdfs tests fail.  
    (Nigel Daley via cutting)
34. HADOOP-633. Keep jobtracker from dying when job initialization
    throws exceptions. Also improve exception handling in a few other
    places and add more informative thread names.  
    (omalley via cutting)
35. HADOOP-669. Fix a problem introduced by HADOOP-90 that can cause
    DFS to lose files.  
    (Milind Bhandarkar via cutting)
36. HADOOP-373. Consistently check the value returned by
    FileSystem.mkdirs().  
    (Wendy Chien via cutting)
37. HADOOP-670. Code cleanups in some DFS internals: use generic
    types, replace Vector with ArrayList, etc.  
    (Konstantin Shvachko via cutting)
38. HADOOP-647. Permit map outputs to use a different compression
    type than the job output.  
    (omalley via cutting)
39. HADOOP-671. Fix file cache to check for pre-existence before
    creating .  
    (Mahadev Konar via cutting)
40. HADOOP-665. Extend many DFSShell commands to accept multiple
    arguments. Now commands like "ls", "rm", etc. will operate on
    multiple files.  
    (Dhruba Borthakur via cutting)

### Release 0.7.2 - 2006-10-18

1. HADOOP-607. Fix a bug where classes included in job jars were not
   found by tasks.  
   (Mahadev Konar via cutting)
2. HADOOP-609. Add a unit test that checks that classes in job jars
   can be found by tasks. Also modify unit tests to specify multiple
   local directories.  
   (Mahadev Konar via cutting)

### Release 0.7.1 - 2006-10-11

1. HADOOP-593. Fix a NullPointerException in the JobTracker.  
   (omalley via cutting)
2. HADOOP-592. Fix a NullPointerException in the IPC Server. Also
   consistently log when stale calls are discarded.  
   (omalley via cutting)
3. HADOOP-594. Increase the DFS safe-mode threshold from .95 to
   .999, so that nearly all blocks must be reported before filesystem
   modifications are permitted.  
   (Konstantin Shvachko via cutting)
4. HADOOP-598. Fix tasks to retry when reporting completion, so that
   a single RPC timeout won't fail a task.  
   (omalley via cutting)
5. HADOOP-597. Fix TaskTracker to not discard map outputs for errors
   in transmitting them to reduce nodes.  
   (omalley via cutting)

### Release 0.7.0 - 2006-10-06

1. HADOOP-243. Fix rounding in the display of task and job progress
   so that things are not shown to be 100% complete until they are in
   fact finished.  
   (omalley via cutting)
2. HADOOP-438. Limit the length of absolute paths in DFS, since the
   file format used to store pathnames has some limitations.  
   (Wendy Chien via cutting)
3. HADOOP-530. Improve error messages in SequenceFile when keys or
   values are of the wrong type.  
   (Hairong Kuang via cutting)
4. HADOOP-288. Add a file caching system and use it in MapReduce to
   cache job jar files on slave nodes.  
   (Mahadev Konar via cutting)
5. HADOOP-533. Fix unit test to not modify conf directory.  
   (Hairong Kuang via cutting)
6. HADOOP-527. Permit specification of the local address that various
   Hadoop daemons should bind to.  
   (Philippe Gassmann via cutting)
7. HADOOP-542. Updates to contrib/streaming: reformatted source code,
   on-the-fly merge sort, a fix for HADOOP-540, etc.  
   (Michel Tourn via cutting)
8. HADOOP-545. Remove an unused config file parameter.  
   (Philippe Gassmann via cutting)
9. HADOOP-548. Add an Ant property "test.output" to build.xml that
   causes test output to be logged to the console.  
   (omalley via cutting)
10. HADOOP-261. Record an error message when map output is lost.  
    (omalley via cutting)
11. HADOOP-293. Report the full list of task error messages in the
    web ui, not just the most recent.  
    (omalley via cutting)
12. HADOOP-551. Restore JobClient's console printouts to only include
    a maximum of one update per one percent of progress.  
    (omalley via cutting)
13. HADOOP-306. Add a "safe" mode to DFS. The name node enters this
    when less than a specified percentage of file data is complete.
    Currently safe mode is only used on startup, but eventually it
    will also be entered when datanodes disconnect and file data
    becomes incomplete. While in safe mode no filesystem
    modifications are permitted and block replication is inhibited.  
    (Konstantin Shvachko via cutting)
14. HADOOP-431. Change 'dfs -rm' to not operate recursively and add a
    new command, 'dfs -rmr' which operates recursively.  
    (Sameer Paranjpye via cutting)
15. HADOOP-263. Include timestamps for job transitions. The web
    interface now displays the start and end times of tasks and the
    start times of sorting and reducing for reduce tasks. Also,
    extend ObjectWritable to handle enums, so that they can be passed
    as RPC parameters.  
    (Sanjay Dahiya via cutting)
16. HADOOP-556. Contrib/streaming: send keep-alive reports to task
    tracker every 10 seconds rather than every 100 records, to avoid
    task timeouts.  
    (Michel Tourn via cutting)
17. HADOOP-547. Fix reduce tasks to ping tasktracker while copying
    data, rather than only between copies, avoiding task timeouts.  
    (Sanjay Dahiya via cutting)
18. HADOOP-537. Fix src/c++/libhdfs build process to create files in
    build/, no longer modifying the source tree.  
    (Arun C Murthy via cutting)
19. HADOOP-487. Throw a more informative exception for unknown RPC
    hosts.  
    (Sameer Paranjpye via cutting)
20. HADOOP-559. Add file name globbing (pattern matching) support to
    the FileSystem API, and use it in DFSShell ('bin/hadoop dfs')
    commands.  
    (Hairong Kuang via cutting)
21. HADOOP-508. Fix a bug in FSDataInputStream. Incorrect data was
    returned after seeking to a random location.  
    (Milind Bhandarkar via cutting)
22. HADOOP-560. Add a "killed" task state. This can be used to
    distinguish kills from other failures. Task state has also been
    converted to use an enum type instead of an int, uncovering a bug
    elsewhere. The web interface is also updated to display killed
    tasks.  
    (omalley via cutting)
23. HADOOP-423. Normalize Paths containing directories named "." and
    "..", using the standard, unix interpretation. Also add checks in
    DFS, prohibiting the use of "." or ".." as directory or file
    names.  
    (Wendy Chien via cutting)
24. HADOOP-513. Replace map output handling with a servlet, rather
    than a JSP page. This fixes an issue where
    IllegalStateException's were logged, sets content-length
    correctly, and better handles some errors.  
    (omalley via cutting)
25. HADOOP-552. Improved error checking when copying map output files
    to reduce nodes.  
    (omalley via cutting)
26. HADOOP-566. Fix scripts to work correctly when accessed through
    relative symbolic links.  
    (Lee Faris via cutting)
27. HADOOP-519. Add positioned read methods to FSInputStream. These
    permit one to read from a stream without moving its position, and
    can hence be performed by multiple threads at once on a single
    stream. Implement an optimized version for DFS and local FS.  
    (Milind Bhandarkar via cutting)
28. HADOOP-522. Permit block compression with MapFile and SetFile.
    Since these formats are always sorted, block compression can
    provide a big advantage.  
    (cutting)
29. HADOOP-567. Record version and revision information in builds. A
    package manifest is added to the generated jar file containing
    version information, and a VersionInfo utility is added that
    includes further information, including the build date and user,
    and the subversion revision and repository. A 'bin/hadoop
    version' comand is added to show this information, and it is also
    added to various web interfaces.  
    (omalley via cutting)
30. HADOOP-568. Fix so that errors while initializing tasks on a
    tasktracker correctly report the task as failed to the jobtracker,
    so that it will be rescheduled.  
    (omalley via cutting)
31. HADOOP-550. Disable automatic UTF-8 validation in Text. This
    permits, e.g., TextInputFormat to again operate on non-UTF-8 data.  
    (Hairong and Mahadev via cutting)
32. HADOOP-343. Fix mapred copying so that a failed tasktracker
    doesn't cause other copies to slow.  
    (Sameer Paranjpye via cutting)
33. HADOOP-239. Add a persistent job history mechanism, so that basic
    job statistics are not lost after 24 hours and/or when the
    jobtracker is restarted.  
    (Sanjay Dahiya via cutting)
34. HADOOP-506. Ignore heartbeats from stale task trackers.  
    (Sanjay Dahiya via cutting)
35. HADOOP-255. Discard stale, queued IPC calls. Do not process
    calls whose clients will likely time out before they receive a
    response. When the queue is full, new calls are now received and
    queued, and the oldest calls are discarded, so that, when servers
    get bogged down, they no longer develop a backlog on the socket.
    This should improve some DFS namenode failure modes.  
    (omalley via cutting)
36. HADOOP-581. Fix datanode to not reset itself on communications
    errors with the namenode. If a request to the namenode fails, the
    datanode should retry, not restart. This reduces the load on the
    namenode, since restarts cause a resend of the block report.  
    (omalley via cutting)

### Release 0.6.2 - 2006-09-18

1. HADOOP-532. Fix a bug reading value-compressed sequence files,
   where an exception was thrown reporting that the full value had not
   been read.  
   (omalley via cutting)
2. HADOOP-534. Change the default value class in JobConf to be Text
   instead of the now-deprecated UTF8. This fixes the Grep example
   program, which was updated to use Text, but relies on this
   default.  
   (Hairong Kuang via cutting)

### Release 0.6.1 - 2006-09-13

1. HADOOP-520. Fix a bug in libhdfs, where write failures were not
   correctly returning error codes.  
   (Arun C Murthy via cutting)
2. HADOOP-523. Fix a NullPointerException when TextInputFormat is
   explicitly specified. Also add a test case for this.  
   (omalley via cutting)
3. HADOOP-521. Fix another NullPointerException finding the
   ClassLoader when using libhdfs.  
   (omalley via cutting)
4. HADOOP-526. Fix a NullPointerException when attempting to start
   two datanodes in the same directory.  
   (Milind Bhandarkar via cutting)
5. HADOOP-529. Fix a NullPointerException when opening
   value-compressed sequence files generated by pre-0.6.0 Hadoop.  
   (omalley via cutting)

### Release 0.6.0 - 2006-09-08

1. HADOOP-427. Replace some uses of DatanodeDescriptor in the DFS
   web UI code with DatanodeInfo, the preferred public class.  
   (Devaraj Das via cutting)
2. HADOOP-426. Fix streaming contrib module to work correctly on
   Solaris. This was causing nightly builds to fail.  
   (Michel Tourn via cutting)
3. HADOOP-400. Improvements to task assignment. Tasks are no longer
   re-run on nodes where they have failed (unless no other node is
   available). Also, tasks are better load-balanced among nodes.  
   (omalley via cutting)
4. HADOOP-324. Fix datanode to not exit when a disk is full, but
   rather simply to fail writes.  
   (Wendy Chien via cutting)
5. HADOOP-434. Change smallJobsBenchmark to use standard Hadoop
   scripts.  
   (Sanjay Dahiya via cutting)
6. HADOOP-453. Fix a bug in Text.setCapacity().  
   (siren via cutting)
7. HADOOP-450. Change so that input types are determined by the
   RecordReader rather than specified directly in the JobConf. This
   facilitates jobs with a variety of input types.

   WARNING: This contains incompatible API changes! The RecordReader
   interface has two new methods that all user-defined InputFormats
   must now define. Also, the values returned by TextInputFormat are
   no longer of class UTF8, but now of class Text.
8. HADOOP-436. Fix an error-handling bug in the web ui.  
   (Devaraj Das via cutting)
9. HADOOP-455. Fix a bug in Text, where DEL was not permitted.  
   (Hairong Kuang via cutting)
10. HADOOP-456. Change the DFS namenode to keep a persistent record
    of the set of known datanodes. This will be used to implement a
    "safe mode" where filesystem changes are prohibited when a
    critical percentage of the datanodes are unavailable.  
    (Konstantin Shvachko via cutting)
11. HADOOP-322. Add a job control utility. This permits one to
    specify job interdependencies. Each job is submitted only after
    the jobs it depends on have successfully completed.  
    (Runping Qi via cutting)
12. HADOOP-176. Fix a bug in IntWritable.Comparator.  
    (Dick King via cutting)
13. HADOOP-421. Replace uses of String in recordio package with Text
    class, for improved handling of UTF-8 data.  
    (Milind Bhandarkar via cutting)
14. HADOOP-464. Improved error message when job jar not found.  
    (Michel Tourn via cutting)
15. HADOOP-469. Fix /bin/bash specifics that have crept into our
    /bin/sh scripts since HADOOP-352.  
    (Jean-Baptiste Quenot via cutting)
16. HADOOP-468. Add HADOOP\_NICENESS environment variable to set
    scheduling priority for daemons.  
    (Vetle Roeim via cutting)
17. HADOOP-473. Fix TextInputFormat to correctly handle more EOL
    formats. Things now work correctly with CR, LF or CRLF.  
    (Dennis Kubes & James White via cutting)
18. HADOOP-461. Make Java 1.5 an explicit requirement.  
    (cutting)
19. HADOOP-54. Add block compression to SequenceFile. One may now
    specify that blocks of keys and values are compressed together,
    improving compression for small keys and values.
    SequenceFile.Writer's constructor is now deprecated and replaced
    with a factory method.  
    (Arun C Murthy via cutting)
20. HADOOP-281. Prohibit DFS files that are also directories.  
    (Wendy Chien via cutting)
21. HADOOP-486. Add the job username to JobStatus instances returned
    by JobClient.  
    (Mahadev Konar via cutting)
22. HADOOP-437. contrib/streaming: Add support for gzipped inputs.  
    (Michel Tourn via cutting)
23. HADOOP-463. Add variable expansion to config files.
    Configuration property values may now contain variable
    expressions. A variable is referenced with the syntax
    '${variable}'. Variables values are found first in the
    configuration, and then in Java system properties. The default
    configuration is modified so that temporary directories are now
    under ${hadoop.tmp.dir}, which is, by default,
    /tmp/hadoop-${user.name}.  
    (Michel Tourn via cutting)
24. HADOOP-419. Fix a NullPointerException finding the ClassLoader
    when using libhdfs.  
    (omalley via cutting)
25. HADOOP-460. Fix contrib/smallJobsBenchmark to use Text instead of
    UTF8.  
    (Sanjay Dahiya via cutting)
26. HADOOP-196. Fix Configuration(Configuration) constructor to work
    correctly.  
    (Sami Siren via cutting)
27. HADOOP-501. Fix Configuration.toString() to handle URL resources.  
    (Thomas Friol via cutting)
28. HADOOP-499. Reduce the use of Strings in contrib/streaming,
    replacing them with Text for better performance.  
    (Hairong Kuang via cutting)
29. HADOOP-64. Manage multiple volumes with a single DataNode.
    Previously DataNode would create a separate daemon per configured
    volume, each with its own connection to the NameNode. Now all
    volumes are handled by a single DataNode daemon, reducing the load
    on the NameNode.  
    (Milind Bhandarkar via cutting)
30. HADOOP-424. Fix MapReduce so that jobs which generate zero splits
    do not fail.  
    (Fr??d??ric Bertin via cutting)
31. HADOOP-408. Adjust some timeouts and remove some others so that
    unit tests run faster.  
    (cutting)
32. HADOOP-507. Fix an IllegalAccessException in DFS.  
    (omalley via cutting)
33. HADOOP-320. Fix so that checksum files are correctly copied when
    the destination of a file copy is a directory.  
    (Hairong Kuang via cutting)
34. HADOOP-286. In DFSClient, avoid pinging the NameNode with
    renewLease() calls when no files are being written.  
    (Konstantin Shvachko via cutting)
35. HADOOP-312. Close idle IPC connections. All IPC connections were
    cached forever. Now, after a connection has been idle for more
    than a configurable amount of time (one second by default), the
    connection is closed, conserving resources on both client and
    server.  
    (Devaraj Das via cutting)
36. HADOOP-497. Permit the specification of the network interface and
    nameserver to be used when determining the local hostname
    advertised by datanodes and tasktrackers.  
    (Lorenzo Thione via cutting)
37. HADOOP-441. Add a compression codec API and extend SequenceFile
    to use it. This will permit the use of alternate compression
    codecs in SequenceFile.  
    (Arun C Murthy via cutting)
38. HADOOP-483. Improvements to libhdfs build and documentation.  
    (Arun C Murthy via cutting)
39. HADOOP-458. Fix a memory corruption bug in libhdfs.  
    (Arun C Murthy via cutting)
40. HADOOP-517. Fix a contrib/streaming bug in end-of-line detection.  
    (Hairong Kuang via cutting)
41. HADOOP-474. Add CompressionCodecFactory, and use it in
    TextInputFormat and TextOutputFormat. Compressed input files are
    automatically decompressed when they have the correct extension.
    Output files will, when output compression is specified, be
    generated with an approprate extension. Also add a gzip codec and
    fix problems with UTF8 text inputs.  
    (omalley via cutting)

### Release 0.5.0 - 2006-08-04

1. HADOOP-352. Fix shell scripts to use /bin/sh instead of
   /bin/bash, for better portability.  
   (Jean-Baptiste Quenot via cutting)
2. HADOOP-313. Permit task state to be saved so that single tasks
   may be manually re-executed when debugging.  
   (omalley via cutting)
3. HADOOP-339. Add method to JobClient API listing jobs that are
   not yet complete, i.e., that are queued or running.  
   (Mahadev Konar via cutting)
4. HADOOP-355. Updates to the streaming contrib module, including
   API fixes, making reduce optional, and adding an input type for
   StreamSequenceRecordReader.  
   (Michel Tourn via cutting)
5. HADOOP-358. Fix a NPE bug in Path.equals().  
   (Fr??d??ric Bertin via cutting)
6. HADOOP-327. Fix ToolBase to not call System.exit() when
   exceptions are thrown.  
   (Hairong Kuang via cutting)
7. HADOOP-359. Permit map output to be compressed.  
   (omalley via cutting)
8. HADOOP-341. Permit input URI to CopyFiles to use the HTTP
   protocol. This lets one, e.g., more easily copy log files into
   DFS.  
   (Arun C Murthy via cutting)
9. HADOOP-361. Remove unix dependencies from streaming contrib
   module tests, making them pure java.  
   (Michel Tourn via cutting)
10. HADOOP-354. Make public methods to stop DFS daemons.  
    (Barry Kaplan via cutting)
11. HADOOP-252. Add versioning to RPC protocols.  
    (Milind Bhandarkar via cutting)
12. HADOOP-356. Add contrib to "compile" and "test" build targets, so
    that this code is better maintained.  
    (Michel Tourn via cutting)
13. HADOOP-307. Add smallJobsBenchmark contrib module. This runs
    lots of small jobs, in order to determine per-task overheads.  
    (Sanjay Dahiya via cutting)
14. HADOOP-342. Add a tool for log analysis: Logalyzer.  
    (Arun C Murthy via cutting)
15. HADOOP-347. Add web-based browsing of DFS content. The namenode
    redirects browsing requests to datanodes. Content requests are
    redirected to datanodes where the data is local when possible.  
    (Devaraj Das via cutting)
16. HADOOP-351. Make Hadoop IPC kernel independent of Jetty.  
    (Devaraj Das via cutting)
17. HADOOP-237. Add metric reporting to DFS and MapReduce. With only
    minor configuration changes, one can now monitor many Hadoop
    system statistics using Ganglia or other monitoring systems.  
    (Milind Bhandarkar via cutting)
18. HADOOP-376. Fix datanode's HTTP server to scan for a free port.  
    (omalley via cutting)
19. HADOOP-260. Add --config option to shell scripts, specifying an
    alternate configuration directory.  
    (Milind Bhandarkar via cutting)
20. HADOOP-381. Permit developers to save the temporary files for
    tasks whose names match a regular expression, to facilliate
    debugging.  
    (omalley via cutting)
21. HADOOP-344. Fix some Windows-related problems with DF.  
    (Konstantin Shvachko via cutting)
22. HADOOP-380. Fix reduce tasks to poll less frequently for map
    outputs.  
    (Mahadev Konar via cutting)
23. HADOOP-321. Refactor DatanodeInfo, in preparation for
    HADOOP-306.  
    (Konstantin Shvachko & omalley via cutting)
24. HADOOP-385. Fix some bugs in record io code generation.  
    (Milind Bhandarkar via cutting)
25. HADOOP-302. Add new Text class to replace UTF8, removing
    limitations of that class. Also refactor utility methods for
    writing zero-compressed integers (VInts and VLongs).  
    (Hairong Kuang via cutting)
26. HADOOP-335. Refactor DFS namespace/transaction logging in
    namenode.  
    (Konstantin Shvachko via cutting)
27. HADOOP-375. Fix handling of the datanode HTTP daemon's port so
    that multiple datanode's can be run on a single host.  
    (Devaraj Das via cutting)
28. HADOOP-386. When removing excess DFS block replicas, remove those
    on nodes with the least free space first.  
    (Johan Oskarson via cutting)
29. HADOOP-389. Fix intermittent failures of mapreduce unit tests.
    Also fix some build dependencies.  
    (Mahadev & Konstantin via cutting)
30. HADOOP-362. Fix a problem where jobs hang when status messages
    are recieved out-of-order.  
    (omalley via cutting)
31. HADOOP-394. Change order of DFS shutdown in unit tests to
    minimize errors logged.  
    (Konstantin Shvachko via cutting)
32. HADOOP-396. Make DatanodeID implement Writable.  
    (Konstantin Shvachko via cutting)
33. HADOOP-377. Permit one to add URL resources to a Configuration.  
    (Jean-Baptiste Quenot via cutting)
34. HADOOP-345. Permit iteration over Configuration key/value pairs.  
    (Michel Tourn via cutting)
35. HADOOP-409. Streaming contrib module: make configuration
    properties available to commands as environment variables.  
    (Michel Tourn via cutting)
36. HADOOP-369. Add -getmerge option to dfs command that appends all
    files in a directory into a single local file.  
    (Johan Oskarson via cutting)
37. HADOOP-410. Replace some TreeMaps with HashMaps in DFS, for
    a 17% performance improvement.  
    (Milind Bhandarkar via cutting)
38. HADOOP-411. Add unit tests for command line parser.  
    (Hairong Kuang via cutting)
39. HADOOP-412. Add MapReduce input formats that support filtering
    of SequenceFile data, including sampling and regex matching.
    Also, move JobConf.newInstance() to a new utility class.  
    (Hairong Kuang via cutting)
40. HADOOP-226. Fix fsck command to properly consider replication
    counts, now that these can vary per file.  
    (Bryan Pendleton via cutting)
41. HADOOP-425. Add a Python MapReduce example, using Jython.  
    (omalley via cutting)

### Release 0.4.0 - 2006-06-28

1. HADOOP-298. Improved progress reports for CopyFiles utility, the
   distributed file copier.  
   (omalley via cutting)
2. HADOOP-299. Fix the task tracker, permitting multiple jobs to
   more easily execute at the same time.  
   (omalley via cutting)
3. HADOOP-250. Add an HTTP user interface to the namenode, running
   on port 50070.  
   (Devaraj Das via cutting)
4. HADOOP-123. Add MapReduce unit tests that run a jobtracker and
   tasktracker, greatly increasing code coverage.  
   (Milind Bhandarkar via cutting)
5. HADOOP-271. Add links from jobtracker's web ui to tasktracker's
   web ui. Also attempt to log a thread dump of child processes
   before they're killed.  
   (omalley via cutting)
6. HADOOP-210. Change RPC server to use a selector instead of a
   thread per connection. This should make it easier to scale to
   larger clusters. Note that this incompatibly changes the RPC
   protocol: clients and servers must both be upgraded to the new
   version to ensure correct operation.  
   (Devaraj Das via cutting)
7. HADOOP-311. Change DFS client to retry failed reads, so that a
   single read failure will not alone cause failure of a task.  
   (omalley via cutting)
8. HADOOP-314. Remove the "append" phase when reducing. Map output
   files are now directly passed to the sorter, without first
   appending them into a single file. Now, the first third of reduce
   progress is "copy" (transferring map output to reduce nodes), the
   middle third is "sort" (sorting map output) and the last third is
   "reduce" (generating output). Long-term, the "sort" phase will
   also be removed.  
   (omalley via cutting)
9. HADOOP-316. Fix a potential deadlock in the jobtracker.  
   (omalley via cutting)
10. HADOOP-319. Fix FileSystem.close() to remove the FileSystem
    instance from the cache.  
    (Hairong Kuang via cutting)
11. HADOOP-135. Fix potential deadlock in JobTracker by acquiring
    locks in a consistent order.  
    (omalley via cutting)
12. HADOOP-278. Check for existence of input directories before
    starting MapReduce jobs, making it easier to debug this common
    error.  
    (omalley via cutting)
13. HADOOP-304. Improve error message for
    UnregisterdDatanodeException to include expected node name.  
    (Konstantin Shvachko via cutting)
14. HADOOP-305. Fix TaskTracker to ask for new tasks as soon as a
    task is finished, rather than waiting for the next heartbeat.
    This improves performance when tasks are short.  
    (Mahadev Konar via cutting)
15. HADOOP-59. Add support for generic command line options. One may
    now specify the filesystem (-fs), the MapReduce jobtracker (-jt),
    a config file (-conf) or any configuration property (-D). The
    "dfs", "fsck", "job", and "distcp" commands currently support
    this, with more to be added.  
    (Hairong Kuang via cutting)
16. HADOOP-296. Permit specification of the amount of reserved space
    on a DFS datanode. One may specify both the percentage free and
    the number of bytes.  
    (Johan Oskarson via cutting)
17. HADOOP-325. Fix a problem initializing RPC parameter classes, and
    remove the workaround used to initialize classes.  
    (omalley via cutting)
18. HADOOP-328. Add an option to the "distcp" command to ignore read
    errors while copying.  
    (omalley via cutting)
19. HADOOP-27. Don't allocate tasks to trackers whose local free
    space is too low.  
    (Johan Oskarson via cutting)
20. HADOOP-318. Keep slow DFS output from causing task timeouts.
    This incompatibly changes some public interfaces, adding a
    parameter to OutputFormat.getRecordWriter() and the new method
    Reporter.progress(), but it makes lots of tasks succeed that were
    previously failing.  
    (Milind Bhandarkar via cutting)

### Release 0.3.2 - 2006-06-09

1. HADOOP-275. Update the streaming contrib module to use log4j for
   its logging.  
   (Michel Tourn via cutting)
2. HADOOP-279. Provide defaults for log4j logging parameters, so
   that things still work reasonably when Hadoop-specific system
   properties are not provided.  
   (omalley via cutting)
3. HADOOP-280. Fix a typo in AllTestDriver which caused the wrong
   test to be run when "DistributedFSCheck" was specified.  
   (Konstantin Shvachko via cutting)
4. HADOOP-240. DFS's mkdirs() implementation no longer logs a warning
   when the directory already exists.  
   (Hairong Kuang via cutting)
5. HADOOP-285. Fix DFS datanodes to be able to re-join the cluster
   after the connection to the namenode is lost.  
   (omalley via cutting)
6. HADOOP-277. Fix a race condition when creating directories.  
   (Sameer Paranjpye via cutting)
7. HADOOP-289. Improved exception handling in DFS datanode.  
   (Konstantin Shvachko via cutting)
8. HADOOP-292. Fix client-side logging to go to standard error
   rather than standard output, so that it can be distinguished from
   application output.  
   (omalley via cutting)
9. HADOOP-294. Fixed bug where conditions for retrying after errors
   in the DFS client were reversed.  
   (omalley via cutting)

### Release 0.3.1 - 2006-06-05

1. HADOOP-272. Fix a bug in bin/hadoop setting log
   parameters.  
   (omalley & cutting)
2. HADOOP-274. Change applications to log to standard output rather
   than to a rolling log file like daemons.  
   (omalley via cutting)
3. HADOOP-262. Fix reduce tasks to report progress while they're
   waiting for map outputs, so that they do not time out.  
   (Mahadev Konar via cutting)
4. HADOOP-245 and HADOOP-246. Improvements to record io package.  
   (Mahadev Konar via cutting)
5. HADOOP-276. Add logging config files to jar file so that they're
   always found.  
   (omalley via cutting)

### Release 0.3.0 - 2006-06-02

1. HADOOP-208. Enhance MapReduce web interface, adding new pages
   for failed tasks, and tasktrackers.  
   (omalley via cutting)
2. HADOOP-204. Tweaks to metrics package.  
   (David Bowen via cutting)
3. HADOOP-209. Add a MapReduce-based file copier. This will
   copy files within or between file systems in parallel.  
   (Milind Bhandarkar via cutting)
4. HADOOP-146. Fix DFS to check when randomly generating a new block
   id that no existing blocks already have that id.  
   (Milind Bhandarkar via cutting)
5. HADOOP-180. Make a daemon thread that does the actual task clean ups, so
   that the main offerService thread in the taskTracker doesn't get stuck
   and miss his heartbeat window. This was killing many task trackers as
   big jobs finished (300+ tasks / node).  
   (omalley via cutting)
6. HADOOP-200. Avoid transmitting entire list of map task names to
   reduce tasks. Instead just transmit the number of map tasks and
   henceforth refer to them by number when collecting map output.  
   (omalley via cutting)
7. HADOOP-219. Fix a NullPointerException when handling a checksum
   exception under SequenceFile.Sorter.sort().  
   (cutting & stack)
8. HADOOP-212. Permit alteration of the file block size in DFS. The
   default block size for new files may now be specified in the
   configuration with the dfs.block.size property. The block size
   may also be specified when files are opened.  
   (omalley via cutting)
9. HADOOP-218. Avoid accessing configuration while looping through
   tasks in JobTracker.  
   (Mahadev Konar via cutting)
10. HADOOP-161. Add hashCode() method to DFS's Block.  
    (Milind Bhandarkar via cutting)
11. HADOOP-115. Map output types may now be specified. These are also
    used as reduce input types, thus permitting reduce input types to
    differ from reduce output types.  
    (Runping Qi via cutting)
12. HADOOP-216. Add task progress to task status page.  
    (Bryan Pendelton via cutting)
13. HADOOP-233. Add web server to task tracker that shows running
    tasks and logs. Also add log access to job tracker web interface.  
    (omalley via cutting)
14. HADOOP-205. Incorporate pending tasks into tasktracker load
    calculations.  
    (Mahadev Konar via cutting)
15. HADOOP-247. Fix sort progress to better handle exceptions.  
    (Mahadev Konar via cutting)
16. HADOOP-195. Improve performance of the transfer of map outputs to
    reduce nodes by performing multiple transfers in parallel, each on
    a separate socket.  
    (Sameer Paranjpye via cutting)
17. HADOOP-251. Fix task processes to be tolerant of failed progress
    reports to their parent process.  
    (omalley via cutting)
18. HADOOP-325. Improve the FileNotFound exceptions thrown by
    LocalFileSystem to include the name of the file.  
    (Benjamin Reed via cutting)
19. HADOOP-254. Use HTTP to transfer map output data to reduce
    nodes. This, together with HADOOP-195, greatly improves the
    performance of these transfers.  
    (omalley via cutting)
20. HADOOP-163. Cause datanodes that\ are unable to either read or
    write data to exit, so that the namenode will no longer target
    them for new blocks and will replicate their data on other nodes.  
    (Hairong Kuang via cutting)
21. HADOOP-222. Add a -setrep option to the dfs commands that alters
    file replication levels.  
    (Johan Oskarson via cutting)
22. HADOOP-75. In DFS, only check for a complete file when the file
    is closed, rather than as each block is written.  
    (Milind Bhandarkar via cutting)
23. HADOOP-124. Change DFS so that datanodes are identified by a
    persistent ID rather than by host and port. This solves a number
    of filesystem integrity problems, when, e.g., datanodes are
    restarted.  
    (Konstantin Shvachko via cutting)
24. HADOOP-256. Add a C API for DFS.  
    (Arun C Murthy via cutting)
25. HADOOP-211. Switch to use the Jakarta Commons logging internally,
    configured to use log4j by default.  
    (Arun C Murthy and cutting)
26. HADOOP-265. Tasktracker now fails to start if it does not have a
    writable local directory for temporary files. In this case, it
    logs a message to the JobTracker and exits.  
    (Hairong Kuang via cutting)
27. HADOOP-270. Fix potential deadlock in datanode shutdown.  
    (Hairong Kuang via cutting)

### Release 0.2.1 - 2006-05-12

1. HADOOP-199. Fix reduce progress (broken by HADOOP-182).  
   (omalley via cutting)
2. HADOOP-201. Fix 'bin/hadoop dfs -report'.  
   (cutting)
3. HADOOP-207. Fix JDK 1.4 incompatibility introduced by HADOOP-96.
   System.getenv() does not work in JDK 1.4.  
   (Hairong Kuang via cutting)

### Release 0.2.0 - 2006-05-05

1. Fix HADOOP-126. 'bin/hadoop dfs -cp' now correctly copies .crc
   files.  
   (Konstantin Shvachko via cutting)
2. Fix HADOOP-51. Change DFS to support per-file replication counts.  
   (Konstantin Shvachko via cutting)
3. Fix HADOOP-131. Add scripts to start/stop dfs and mapred daemons.
   Use these in start/stop-all scripts.  
   (Chris Mattmann via cutting)
4. Stop using ssh options by default that are not yet in widely used
   versions of ssh. Folks can still enable their use by uncommenting
   a line in conf/hadoop-env.sh.  
   (cutting)
5. Fix HADOOP-92. Show information about all attempts to run each
   task in the web ui.  
   (Mahadev konar via cutting)
6. Fix HADOOP-128. Improved DFS error handling.  
   (Owen O'Malley via cutting)
7. Fix HADOOP-129. Replace uses of java.io.File with new class named
   Path. This fixes bugs where java.io.File methods were called
   directly when FileSystem methods were desired, and reduces the
   likelihood of such bugs in the future. It also makes the handling
   of pathnames more consistent between local and dfs FileSystems and
   between Windows and Unix. java.io.File-based methods are still
   available for back-compatibility, but are deprecated and will be
   removed once 0.2 is released.  
   (cutting)
8. Change dfs.data.dir and mapred.local.dir to be comma-separated
   lists of directories, no longer be space-separated. This fixes
   several bugs on Windows.  
   (cutting)
9. Fix HADOOP-144. Use mapred task id for dfs client id, to
   facilitate debugging.  
   (omalley via cutting)
10. Fix HADOOP-143. Do not line-wrap stack-traces in web ui.  
    (omalley via cutting)
11. Fix HADOOP-118. In DFS, improve clean up of abandoned file
    creations.  
    (omalley via cutting)
12. Fix HADOOP-138. Stop multiple tasks in a single heartbeat, rather
    than one per heartbeat.  
    (Stefan via cutting)
13. Fix HADOOP-139. Remove a potential deadlock in
    LocalFileSystem.lock().  
    (Igor Bolotin via cutting)
14. Fix HADOOP-134. Don't hang jobs when the tasktracker is
    misconfigured to use an un-writable local directory.  
    (omalley via cutting)
15. Fix HADOOP-115. Correct an error message.  
    (Stack via cutting)
16. Fix HADOOP-133. Retry pings from child to parent, in case of
    (local) communcation problems. Also log exit status, so that one
    can distinguish patricide from other deaths.  
    (omalley via cutting)
17. Fix HADOOP-142. Avoid re-running a task on a host where it has
    previously failed.  
    (omalley via cutting)
18. Fix HADOOP-148. Maintain a task failure count for each
    tasktracker and display it in the web ui.  
    (omalley via cutting)
19. Fix HADOOP-151. Close a potential socket leak, where new IPC
    connection pools were created per configuration instance that RPCs
    use. Now a global RPC connection pool is used again, as
    originally intended.  
    (cutting)
20. Fix HADOOP-69. Don't throw a NullPointerException when getting
    hints for non-existing file split.  
    (Bryan Pendelton via cutting)
21. Fix HADOOP-157. When a task that writes dfs files (e.g., a reduce
    task) failed and was retried, it would fail again and again,
    eventually failing the job. The problem was that dfs did not yet
    know that the failed task had abandoned the files, and would not
    yet let another task create files with the same names. Dfs now
    retries when creating a file long enough for locks on abandoned
    files to expire.  
    (omalley via cutting)
22. Fix HADOOP-150. Improved task names that include job
    names.  
    (omalley via cutting)
23. Fix HADOOP-162. Fix ConcurrentModificationException when
    releasing file locks.  
    (omalley via cutting)
24. Fix HADOOP-132. Initial check-in of new Metrics API, including
    implementations for writing metric data to a file and for sending
    it to Ganglia.  
    (David Bowen via cutting)
25. Fix HADOOP-160. Remove some uneeded synchronization around
    time-consuming operations in the TaskTracker.  
    (omalley via cutting)
26. Fix HADOOP-166. RPCs failed when passed subclasses of a declared
    parameter type. This is fixed by changing ObjectWritable to store
    both the declared type and the instance type for Writables. Note
    that this incompatibly changes the format of ObjectWritable and
    will render unreadable any ObjectWritables stored in files.
    Nutch only uses ObjectWritable in intermediate files, so this
    should not be a problem for Nutch.  
    (Stefan & cutting)
27. Fix HADOOP-168. MapReduce RPC protocol methods should all declare
    IOException, so that timeouts are handled appropriately.  
    (omalley via cutting)
28. Fix HADOOP-169. Don't fail a reduce task if a call to the
    jobtracker to locate map outputs fails.  
    (omalley via cutting)
29. Fix HADOOP-170. Permit FileSystem clients to examine and modify
    the replication count of individual files. Also fix a few
    replication-related bugs.  
    (Konstantin Shvachko via cutting)
30. Permit specification of a higher replication levels for job
    submission files (job.xml and job.jar). This helps with large
    clusters, since these files are read by every node.  
    (cutting)
31. HADOOP-173. Optimize allocation of tasks with local data.  
    (cutting)
32. HADOOP-167. Reduce number of Configurations and JobConf's
    created.  
    (omalley via cutting)
33. NUTCH-256. Change FileSystem#createNewFile() to create a .crc
    file. The lack of a .crc file was causing warnings.  
    (cutting)
34. HADOOP-174. Change JobClient to not abort job until it has failed
    to contact the job tracker for five attempts, not just one as
    before.  
    (omalley via cutting)
35. HADOOP-177. Change MapReduce web interface to page through tasks.
    Previously, when jobs had more than a few thousand tasks they
    could crash web browsers.  
    (Mahadev Konar via cutting)
36. HADOOP-178. In DFS, piggyback blockwork requests from datanodes
    on heartbeat responses from namenode. This reduces the volume of
    RPC traffic. Also move startup delay in blockwork from datanode
    to namenode. This fixes a problem where restarting the namenode
    triggered a lot of uneeded replication.  
    (Hairong Kuang via cutting)
37. HADOOP-183. If the DFS namenode is restarted with different
    minimum and/or maximum replication counts, existing files'
    replication counts are now automatically adjusted to be within the
    newly configured bounds.  
    (Hairong Kuang via cutting)
38. HADOOP-186. Better error handling in TaskTracker's top-level
    loop. Also improve calculation of time to send next heartbeat.  
    (omalley via cutting)
39. HADOOP-187. Add two MapReduce examples/benchmarks. One creates
    files containing random data. The second sorts the output of the
    first.  
    (omalley via cutting)
40. HADOOP-185. Fix so that, when a task tracker times out making the
    RPC asking for a new task to run, the job tracker does not think
    that it is actually running the task returned.  
    (omalley via cutting)
41. HADOOP-190. If a child process hangs after it has reported
    completion, its output should not be lost.  
    (Stack via cutting)
42. HADOOP-184. Re-structure some test code to better support testing
    on a cluster.  
    (Mahadev Konar via cutting)
43. HADOOP-191 Add streaming package, Hadoop's first contrib module.
    This permits folks to easily submit MapReduce jobs whose map and
    reduce functions are implemented by shell commands. Use
    'bin/hadoop jar build/hadoop-streaming.jar' to get details.  
    (Michel Tourn via cutting)
44. HADOOP-189. Fix MapReduce in standalone configuration to
    correctly handle job jar files that contain a lib directory with
    nested jar files.  
    (cutting)
45. HADOOP-65. Initial version of record I/O framework that enables
    the specification of record types and generates marshalling code
    in both Java and C++. Generated Java code implements
    WritableComparable, but is not yet otherwise used by
    Hadoop.  
    (Milind Bhandarkar via cutting)
46. HADOOP-193. Add a MapReduce-based FileSystem benchmark.  
    (Konstantin Shvachko via cutting)
47. HADOOP-194. Add a MapReduce-based FileSystem checker. This reads
    every block in every file in the filesystem.  
    (Konstantin Shvachko
    via cutting)
48. HADOOP-182. Fix so that lost task trackers to not change the
    status of reduce tasks or completed jobs. Also fixes the progress
    meter so that failed tasks are subtracted.  
    (omalley via cutting)
49. HADOOP-96. Logging improvements. Log files are now separate from
    standard output and standard error files. Logs are now rolled.
    Logging of all DFS state changes can be enabled, to facilitate
    debugging.  
    (Hairong Kuang via cutting)

### Release 0.1.1 - 2006-04-08

1. Added CHANGES.txt, logging all significant changes to Hadoop.  
   (cutting)
2. Fix MapReduceBase.close() to throw IOException, as declared in the
   Closeable interface. This permits subclasses which override this
   method to throw that exception.  
   (cutting)
3. Fix HADOOP-117. Pathnames were mistakenly transposed in
   JobConf.getLocalFile() causing many mapred temporary files to not
   be removed.  
   (Raghavendra Prabhu via cutting)
4. Fix HADOOP-116. Clean up job submission files when jobs complete.  
   (cutting)
5. Fix HADOOP-125. Fix handling of absolute paths on Windows  
   (cutting)

### Release 0.1.0 - 2006-04-01

1. The first release of Hadoop.
