# Supplementary material for: Provenance based data integrity checking and verification in cloud environments
Source: PLoS One. 2017 May 17;12(5):e0177576. doi: 10.1371/journal.pone.0177576 (PMC5435237; doi:10.1371/journal.pone.0177576)
Supplement: S1 Dataset — Files in the Dataset are utilized for performance measurement. (ZIP) [file pone.0177576.s001.zip › dataset/html/cluster_setup - Copy (2) - Copy.html]

Hadoop Cluster Setup


Apache > Hadoop > Core

- Project
- Wiki
- Hadoop 0.18 Documentation

Documentation

Overview

Quickstart

Cluster Setup

HDFS Architecture

HDFS User Guide

HDFS Permissions Guide

HDFS Quotas Administrator Guide

Commands Manual

FS Shell Guide

DistCp Guide

Map-Reduce Tutorial

Native Hadoop Libraries

Streaming

Hadoop Archives

Hadoop On Demand

API Docs

Wiki

FAQ

Mailing Lists

Release Notes

All Changes


PDF

# Hadoop Cluster Setup

- Purpose
- Pre-requisites
- Installation
- Configuration
  - Configuration Files
  - Site Configuration
    - Configuring the Environment of the Hadoop Daemons
    - Configuring the Hadoop Daemons
    - Slaves
    - Logging
- Hadoop Rack Awareness
- Hadoop Startup
- Hadoop Shutdown

## Purpose

This document describes how to install, configure and manage non-trivial
Hadoop clusters ranging from a few nodes to extremely large clusters with
thousands of nodes.

If you are looking to install Hadoop on a single machine to play
with it, you can find relevant details here.

## Pre-requisites

1. Make sure all requisite software
   is installed on all nodes in your cluster.
2. Get the Hadoop software.

## Installation

Installing a Hadoop cluster typically involves unpacking the software
on all the machines in the cluster.

Typically one machine in the cluster is designated as the
NameNode and another machine the as JobTracker,
exclusively. These are the *masters*. The rest of the machines in
the cluster act as both DataNode *and*
TaskTracker. These are the *slaves*.

The root of the distribution is referred to as
HADOOP\_HOME. All machines in the cluster usually have the same
HADOOP\_HOME path.

## Configuration

The following sections describe how to configure a Hadoop cluster.

### Configuration Files

Hadoop configuration is driven by two important configuration files
found in the conf/ directory of the distribution:

1. hadoop-default.xml - Read-only
   default configuration.
2. *hadoop-site.xml* - Site-specific configuration.

To learn more about how the Hadoop framework is controlled by these
configuration files, look
here.

Additionally, you can control the Hadoop scripts found in the
bin/ directory of the distribution, by setting site-specific
values via the conf/hadoop-env.sh.

### Site Configuration

To configure the the Hadoop cluster you will need to configure the
*environment* in which the Hadoop daemons execute as well as
the *configuration parameters* for the Hadoop daemons.

The Hadoop daemons are NameNode/DataNode
and JobTracker/TaskTracker.

#### Configuring the Environment of the Hadoop Daemons

Administrators should use the conf/hadoop-env.sh script
to do site-specific customization of the Hadoop daemons' process
environment.

At the very least you should specify the
JAVA\_HOME so that it is correctly defined on each
remote node.

Administrators can configure individual daemons using the
configuration options HADOOP\_\*\_OPTS. Various options
available are shown below in the table.

| Daemon | Configure Options |
| --- | --- |
| NameNode | HADOOP\_NAMENODE\_OPTS |
| DataNode | HADOOP\_DATANODE\_OPTS |
| SecondaryNamenode | HADOOP\_SECONDARYNAMENODE\_OPTS |
| JobTracker | HADOOP\_JOBTRACKER\_OPTS |
| TaskTracker | HADOOP\_TASKTRACKER\_OPTS |

For example, To configure Namenode to use parallelGC, the
following statement should be added in hadoop-env.sh :
  

export HADOOP\_NAMENODE\_OPTS="-XX:+UseParallelGC ${HADOOP\_NAMENODE\_OPTS}"

Other useful configuration parameters that you can customize
include:

- HADOOP\_LOG\_DIR - The directory where the daemons'
  log files are stored. They are automatically created if they don't
  exist.
- HADOOP\_HEAPSIZE - The maximum amount of heapsize
  to use, in MB e.g. 1000MB. This is used to
  configure the heap size for the hadoop daemon. By default,
  the value is 1000MB.

#### Configuring the Hadoop Daemons

This section deals with important parameters to be specified in the
conf/hadoop-site.xml for the Hadoop cluster.

| Parameter | Value | Notes |
| --- | --- | --- |
| fs.default.name | URI of NameNode. | *hdfs://hostname/* |
| mapred.job.tracker | Host or IP and port of JobTracker. | *host:port* pair. |
| dfs.name.dir | Path on the local filesystem where the NameNode stores the namespace and transactions logs persistently. | If this is a comma-delimited list of directories then the name table is replicated in all of the directories, for redundancy. |
| dfs.data.dir | Comma separated list of paths on the local filesystem of a DataNode where it should store its blocks. | If this is a comma-delimited list of directories, then data will be stored in all named directories, typically on different devices. |
| mapred.system.dir | Path on the HDFS where where the Map/Reduce framework stores system files e.g. /hadoop/mapred/system/. | This is in the default filesystem (HDFS) and must be accessible from both the server and client machines. |
| mapred.local.dir | Comma-separated list of paths on the local filesystem where temporary Map/Reduce data is written. | Multiple paths help spread disk i/o. |
| mapred.tasktracker.{map\|reduce}.tasks.maximum | The maximum number of Map/Reduce tasks, which are run simultaneously on a given TaskTracker, individually. | Defaults to 2 (2 maps and 2 reduces), but vary it depending on your hardware. |
| dfs.hosts/dfs.hosts.exclude | List of permitted/excluded DataNodes. | If necessary, use these files to control the list of allowable datanodes. |
| mapred.hosts/mapred.hosts.exclude | List of permitted/excluded TaskTrackers. | If necessary, use these files to control the list of allowable TaskTrackers. |

Typically all the above parameters are marked as
final to ensure that they cannot be overriden by user-applications.

##### Real-World Cluster Configurations

This section lists some non-default configuration parameters which
have been used to run the *sort* benchmark on very large
clusters.

- Some non-default configuration values used to run sort900,
  that is 9TB of data sorted on a cluster with 900 nodes:

  | Parameter | Value | Notes |
  | --- | --- | --- |
  | dfs.block.size | 134217728 | HDFS blocksize of 128MB for large file-systems. |
  | dfs.namenode.handler.count | 40 | More NameNode server threads to handle RPCs from large number of DataNodes. |
  | mapred.reduce.parallel.copies | 20 | Higher number of parallel copies run by reduces to fetch outputs from very large number of maps. |
  | mapred.child.java.opts | -Xmx512M | Larger heap-size for child jvms of maps/reduces. |
  | fs.inmemory.size.mb | 200 | Larger amount of memory allocated for the in-memory file-system used to merge map-outputs at the reduces. |
  | io.sort.factor | 100 | More streams merged at once while sorting files. |
  | io.sort.mb | 200 | Higher memory-limit while sorting data. |
  | io.file.buffer.size | 131072 | Size of read/write buffer used in SequenceFiles. |
- Updates to some configuration values to run sort1400 and
  sort2000, that is 14TB of data sorted on 1400 nodes and 20TB of
  data sorted on 2000 nodes:

  | Parameter | Value | Notes |
  | --- | --- | --- |
  | mapred.job.tracker.handler.count | 60 | More JobTracker server threads to handle RPCs from large number of TaskTrackers. |
  | mapred.reduce.parallel.copies | 50 |  |
  | tasktracker.http.threads | 50 | More worker threads for the TaskTracker's http server. The http server is used by reduces to fetch intermediate map-outputs. |
  | mapred.child.java.opts | -Xmx1024M | Larger heap-size for child jvms of maps/reduces. |

#### Slaves

Typically you choose one machine in the cluster to act as the
NameNode and one machine as to act as the
JobTracker, exclusively. The rest of the machines act as
both a DataNode and TaskTracker and are
referred to as *slaves*.

List all slave hostnames or IP addresses in your
conf/slaves file, one per line.

#### Logging

Hadoop uses the Apache
log4j via the Apache
Commons Logging framework for logging. Edit the
conf/log4j.properties file to customize the Hadoop
daemons' logging configuration (log-formats and so on).

##### History Logging

The job history files are stored in central location
 hadoop.job.history.location  which can be on DFS also,
whose default value is ${HADOOP\_LOG\_DIR}/history.
The history web UI is accessible from job tracker web UI.

The history files are also logged to user specified directory
hadoop.job.history.user.location
which defaults to job output directory. The files are stored in
"\_logs/history/" in the specified directory. Hence, by default
they will be in "mapred.output.dir/\_logs/history/". User can stop
logging by giving the value none for
hadoop.job.history.user.location

User can view the history logs summary in specified directory
using the following command   
$ bin/hadoop job -history output-dir
  
This command will print job details, failed and killed tip
details.   
More details about the job such as successful tasks and
task attempts made for each task can be viewed using the
following command   
$ bin/hadoop job -history all output-dir

Once all the necessary configuration is complete, distribute the files
to the HADOOP\_CONF\_DIR directory on all the machines,
typically ${HADOOP\_HOME}/conf.

## Hadoop Rack Awareness

The HDFS and the Map/Reduce components are rack-aware.

The NameNode and the JobTracker obtains the
rack id of the slaves in the cluster by invoking an API
resolve in an administrator configured
module. The API resolves the slave's DNS name (also IP address) to a
rack id. What module to use can be configured using the configuration
item topology.node.switch.mapping.impl. The default
implementation of the same runs a script/command configured using
topology.script.file.name. If topology.script.file.name is
not set, the rack id /default-rack is returned for any
passed IP address. The additional configuration in the Map/Reduce
part is mapred.cache.task.levels which determines the number
of levels (in the network topology) of caches. So, for example, if it is
the default value of 2, two levels of caches will be constructed -
one for hosts (host -> task mapping) and another for racks
(rack -> task mapping).

## Hadoop Startup

To start a Hadoop cluster you will need to start both the HDFS and
Map/Reduce cluster.

Format a new distributed filesystem:  
$ bin/hadoop namenode -format

Start the HDFS with the following command, run on the designated
NameNode:  
$ bin/start-dfs.sh

The bin/start-dfs.sh script also consults the
${HADOOP\_CONF\_DIR}/slaves file on the NameNode
and starts the DataNode daemon on all the listed slaves.

Start Map-Reduce with the following command, run on the designated
JobTracker:  
$ bin/start-mapred.sh

The bin/start-mapred.sh script also consults the
${HADOOP\_CONF\_DIR}/slaves file on the JobTracker
and starts the TaskTracker daemon on all the listed slaves.

## Hadoop Shutdown

Stop HDFS with the following command, run on the designated
NameNode:  
$ bin/stop-dfs.sh

The bin/stop-dfs.sh script also consults the
${HADOOP\_CONF\_DIR}/slaves file on the NameNode
and stops the DataNode daemon on all the listed slaves.

Stop Map/Reduce with the following command, run on the designated
the designated JobTracker:  
$ bin/stop-mapred.sh

The bin/stop-mapred.sh script also consults the
${HADOOP\_CONF\_DIR}/slaves file on the JobTracker
and stops the TaskTracker daemon on all the listed slaves.

Copyright ©
2007 The Apache Software Foundation.
