# Supplementary material for: Provenance based data integrity checking and verification in cloud environments
Source: PLoS One. 2017 May 17;12(5):e0177576. doi: 10.1371/journal.pone.0177576 (PMC5435237; doi:10.1371/journal.pone.0177576)
Supplement: S1 Dataset — Files in the Dataset are utilized for performance measurement. (ZIP) [file pone.0177576.s001.zip › dataset/html/commands_manual - Copy (2) - Copy.html]

Commands Manual


Apache > Hadoop > Core

- Project
- Wiki
- Hadoop 0.18 Documentation

Documentation

Overview

Quickstart

Cluster Setup

HDFS Architecture

HDFS User Guide

HDFS Permissions Guide

HDFS Quotas Administrator Guide

Commands Manual

FS Shell Guide

DistCp Guide

Map-Reduce Tutorial

Native Hadoop Libraries

Streaming

Hadoop Archives

Hadoop On Demand

API Docs

Wiki

FAQ

Mailing Lists

Release Notes

All Changes


PDF

# Commands Manual

- Overview
  - Generic Options
- User Commands 
  - archive
  - distcp
  - fs
  - fsck
  - jar
  - job
  - pipes
  - version
  - CLASSNAME
- Administration Commands 
  - balancer
  - daemonlog
  - datanode
  - dfsadmin
  - jobtracker
  - namenode
  - secondarynamenode
  - tasktracker

## Overview

All the hadoop commands are invoked by the bin/hadoop script. Running hadoop
script without any arguments prints the description for all commands.

Usage: hadoop [--config confdir] [COMMAND] [GENERIC\_OPTIONS] [COMMAND\_OPTIONS]

Hadoop has an option parsing framework that employs parsing generic options as well as running classes.

| COMMAND\_OPTION | Description |
| --- | --- |
| --config confdir | Overwrites the default Configuration directory. Default is ${HADOOP\_HOME}/conf. |
| GENERIC\_OPTIONS | The common set of options supported by multiple commands. |
| COMMAND   COMMAND\_OPTIONS | Various commands with their options are described in the following sections. The commands have been grouped into User Commands and Administration Commands. |

### Generic Options

Following are supported by dfsadmin,
fs, fsck and
job.
Applications should implement
Tool to support
GenericOptions.

| GENERIC\_OPTION | Description |
| --- | --- |
| -conf <configuration file> | Specify an application configuration file. |
| -D <property=value> | Use value for given property. |
| -fs <local\|namenode:port> | Specify a namenode. |
| -jt <local\|jobtracker:port> | Specify a job tracker. Applies only to job. |
| -files <comma separated list of files> | Specify comma separated files to be copied to the map reduce cluster. Applies only to job. |
| -libjars <comma seperated list of jars> | Specify comma separated jar files to include in the classpath. Applies only to job. |
| -archives <comma separated list of archives> | Specify comma separated archives to be unarchived on the compute machines. Applies only to job. |

## User Commands

Commands useful for users of a hadoop cluster.

### archive

Creates a hadoop archive. More information can be found at Hadoop Archives.

Usage: hadoop archive -archiveName NAME <src>\* <dest>

| COMMAND\_OPTION | Description |
| --- | --- |
| -archiveName NAME | Name of the archive to be created. |
| src | Filesystem pathnames which work as usual with regular expressions. |
| dest | Destination directory which would contain the archive. |

### distcp

Copy file or directories recursively. More information can be found at DistCp Guide.

Usage: hadoop distcp <srcurl> <desturl>

| COMMAND\_OPTION | Description |
| --- | --- |
| srcurl | Source Url |
| desturl | Destination Url |

### fs

Usage: hadoop fs [GENERIC\_OPTIONS]
[COMMAND\_OPTIONS]

Runs a generic filesystem user client.

The various COMMAND\_OPTIONS can be found at HDFS Shell Guide.

### fsck

Runs a HDFS filesystem checking utility. See Fsck for more info.

Usage: hadoop fsck [GENERIC\_OPTIONS]
<path> [-move | -delete | -openforwrite] [-files [-blocks
[-locations | -racks]]]

| COMMAND\_OPTION | Description |
| --- | --- |
| <path> | Start checking from this path. |
| -move | Move corrupted files to /lost+found |
| -delete | Delete corrupted files. |
| -openforwrite | Print out files opened for write. |
| -files | Print out files being checked. |
| -blocks | Print out block report. |
| -locations | Print out locations for every block. |
| -racks | Print out network topology for data-node locations. |

### jar

Runs a jar file. Users can bundle their Map Reduce code in a jar file and execute it using this command.

Usage: hadoop jar <jar> [mainClass] args...

The streaming jobs are run via this command. Examples can be referred from
Streaming examples

Word count example is also run using jar command. It can be referred from
Wordcount example

### job

Command to interact with Map Reduce Jobs.

Usage: hadoop job [GENERIC\_OPTIONS]
[-submit <job-file>] | [-status <job-id>] |
[-counter <job-id> <group-name> <counter-name>] | [-kill <job-id>] |
[-events <job-id> <from-event-#> <#-of-events>] | [-history [all] <jobOutputDir>] |
[-list [all]] | [-kill-task <task-id>] | [-fail-task <task-id>]

| COMMAND\_OPTION | Description |
| --- | --- |
| -submit <job-file> | Submits the job. |
| -status <job-id> | Prints the map and reduce completion percentage and all job counters. |
| -counter <job-id> <group-name> <counter-name> | Prints the counter value. |
| -kill <job-id> | Kills the job. |
| -events <job-id> <from-event-#> <#-of-events> | Prints the events' details received by jobtracker for the given range. |
| -history [all] <jobOutputDir> | -history <jobOutputDir> prints job details, failed and killed tip details. More details about the job such as successful tasks and task attempts made for each task can be viewed by specifying the [all] option. |
| -list [all] | -list all displays all jobs. -list displays only jobs which are yet to complete. |
| -kill-task <task-id> | Kills the task. Killed tasks are NOT counted against failed attempts. |
| -fail-task <task-id> | Fails the task. Failed tasks are counted against failed attempts. |

### pipes

Runs a pipes job.

Usage: hadoop pipes [-conf <path>] [-jobconf <key=value>, <key=value>, ...]
[-input <path>] [-output <path>] [-jar <jar file>] [-inputformat <class>]
[-map <class>] [-partitioner <class>] [-reduce <class>] [-writer <class>]
[-program <executable>] [-reduces <num>]

| COMMAND\_OPTION | Description |
| --- | --- |
| -conf <path> | Configuration for job |
| -jobconf <key=value>, <key=value>, ... | Add/override configuration for job |
| -input <path> | Input directory |
| -output <path> | Output directory |
| -jar <jar file> | Jar filename |
| -inputformat <class> | InputFormat class |
| -map <class> | Java Map class |
| -partitioner <class> | Java Partitioner |
| -reduce <class> | Java Reduce class |
| -writer <class> | Java RecordWriter |
| -program <executable> | Executable URI |
| -reduces <num> | Number of reduces |

### version

Prints the version.

Usage: hadoop version

### CLASSNAME

hadoop script can be used to invoke any class.

Usage: hadoop CLASSNAME

Runs the class named CLASSNAME.

## Administration Commands

Commands useful for administrators of a hadoop cluster.

### balancer

Runs a cluster balancing utility. An administrator can simply press Ctrl-C to stop the
rebalancing process. See Rebalancer for more details.

Usage: hadoop balancer [-threshold <threshold>]

| COMMAND\_OPTION | Description |
| --- | --- |
| -threshold <threshold> | Percentage of disk capacity. This overwrites the default threshold. |

### daemonlog

Get/Set the log level for each daemon.

Usage: hadoop daemonlog -getlevel <host:port> <name>
  
Usage: hadoop daemonlog -setlevel <host:port> <name> <level>

| COMMAND\_OPTION | Description |
| --- | --- |
| -getlevel <host:port> <name> | Prints the log level of the daemon running at <host:port>. This command internally connects to http://<host:port>/logLevel?log=<name> |
| -setlevel <host:port> <name> <level> | Sets the log level of the daemon running at <host:port>. This command internally connects to http://<host:port>/logLevel?log=<name> |

### datanode

Runs a HDFS datanode.

Usage: hadoop datanode [-rollback]

| COMMAND\_OPTION | Description |
| --- | --- |
| -rollback | Rollsback the datanode to the previous version. This should be used after stopping the datanode and distributing the old hadoop version. |

### dfsadmin

Runs a HDFS dfsadmin client.

Usage: hadoop dfsadmin [GENERIC\_OPTIONS] [-report] [-safemode enter | leave | get | wait] [-refreshNodes]
[-finalizeUpgrade] [-upgradeProgress status | details | force] [-metasave filename]
[-setQuota <quota> <dirname>...<dirname>] [-clrQuota <dirname>...<dirname>]
[-help [cmd]]

| COMMAND\_OPTION | Description |
| --- | --- |
| -report | Reports basic filesystem information and statistics. |
| -safemode enter \| leave \| get \| wait | Safe mode maintenance command. Safe mode is a Namenode state in which it   1. does not accept changes to the name space (read-only)   2. does not replicate or delete blocks.   Safe mode is entered automatically at Namenode startup, and leaves safe mode automatically when the configured minimum percentage of blocks satisfies the minimum replication condition. Safe mode can also be entered manually, but then it can only be turned off manually as well. |
| -refreshNodes | Re-read the hosts and exclude files to update the set of Datanodes that are allowed to connect to the Namenode and those that should be decommissioned or recommissioned. |
| -finalizeUpgrade | Finalize upgrade of HDFS. Datanodes delete their previous version working directories, followed by Namenode doing the same. This completes the upgrade process. |
| -upgradeProgress status \| details \| force | Request current distributed upgrade status, a detailed status or force the upgrade to proceed. |
| -metasave filename | Save Namenode's primary data structures to <filename> in the directory specified by hadoop.log.dir property. <filename> will contain one line for each of the following   1. Datanodes heart beating with Namenode  2. Blocks waiting to be replicated  3. Blocks currrently being replicated  4. Blocks waiting to be deleted |
| -setQuota <quota> <dirname>...<dirname> | Set the quota <quota> for each directory <dirname>. The directory quota is a long integer that puts a hard limit on the number of names in the directory tree.  Best effort for the directory, with faults reported if  1. N is not a positive integer, or  2. user is not an administrator, or  3. the directory does not exist or is a file, or  4. the directory would immediately exceed the new quota. |
| -clrQuota <dirname>...<dirname> | Clear the quota for each directory <dirname>.  Best effort for the directory. with fault reported if  1. the directory does not exist or is a file, or  2. user is not an administrator.  It does not fault if the directory has no quota. |
| -help [cmd] | Displays help for the given command or all commands if none is specified. |

### jobtracker

Runs the MapReduce job Tracker node.

Usage: hadoop jobtracker

### namenode

Runs the namenode. More info about the upgrade, rollback and finalize is at
Upgrade Rollback

Usage: hadoop namenode [-format] | [-upgrade] | [-rollback] | [-finalize] | [-importCheckpoint]

| COMMAND\_OPTION | Description |
| --- | --- |
| -format | Formats the namenode. It starts the namenode, formats it and then shut it down. |
| -upgrade | Namenode should be started with upgrade option after the distribution of new hadoop version. |
| -rollback | Rollsback the namenode to the previous version. This should be used after stopping the cluster and distributing the old hadoop version. |
| -finalize | Finalize will remove the previous state of the files system. Recent upgrade will become permanent. Rollback option will not be available anymore. After finalization it shuts the namenode down. |
| -importCheckpoint | Loads image from a checkpoint directory and save it into the current one. Checkpoint dir is read from property fs.checkpoint.dir |

### secondarynamenode

Runs the HDFS secondary namenode. See Secondary Namenode
for more info.

Usage: hadoop secondarynamenode [-checkpoint [force]] | [-geteditsize]

| COMMAND\_OPTION | Description |
| --- | --- |
| -checkpoint [force] | Checkpoints the Secondary namenode if EditLog size >= fs.checkpoint.size. If -force is used, checkpoint irrespective of EditLog size. |
| -geteditsize | Prints the EditLog size. |

### tasktracker

Runs a MapReduce task Tracker node.

Usage: hadoop tasktracker

Copyright ©
2007 The Apache Software Foundation.
