# Supplementary material for: Provenance based data integrity checking and verification in cloud environments
Source: PLoS One. 2017 May 17;12(5):e0177576. doi: 10.1371/journal.pone.0177576 (PMC5435237; doi:10.1371/journal.pone.0177576)
Supplement: S1 Dataset — Files in the Dataset are utilized for performance measurement. (ZIP) [file pone.0177576.s001.zip › dataset/html/distcp - Copy (2) - Copy.html]

DistCp


Apache > Hadoop > Core

- Project
- Wiki
- Hadoop 0.18 Documentation

Documentation

Overview

Quickstart

Cluster Setup

HDFS Architecture

HDFS User Guide

HDFS Permissions Guide

HDFS Quotas Administrator Guide

Commands Manual

FS Shell Guide

DistCp Guide

Map-Reduce Tutorial

Native Hadoop Libraries

Streaming

Hadoop Archives

Hadoop On Demand

API Docs

Wiki

FAQ

Mailing Lists

Release Notes

All Changes


PDF

# DistCp

- Overview
- Usage
  - Basic
  - Options
    - Option Index
    - Update and Overwrite
- Appendix
  - Map sizing
  - Copying between versions of HDFS
  - Map/Reduce and other side-effects

## Overview

DistCp (distributed copy) is a tool used for large inter/intra-cluster
copying. It uses Map/Reduce to effect its distribution, error
handling and recovery, and reporting. It expands a list of files and
directories into input to map tasks, each of which will copy a partition
of the files specified in the source list. Its Map/Reduce pedigree has
endowed it with some quirks in both its semantics and execution. The
purpose of this document is to offer guidance for common tasks and to
elucidate its model.

## Usage

### Basic

The most common invocation of DistCp is an inter-cluster copy:

bash$ hadoop distcp hdfs://nn1:8020/foo/bar \
  
         
         
hdfs://nn2:8020/bar/foo

This will expand the namespace under /foo/bar on nn1
into a temporary file, partition its contents among a set of map
tasks, and start a copy on each TaskTracker from nn1 to nn2. Note
that DistCp expects absolute paths.

One can also specify multiple source directories on the command
line:

bash$ hadoop distcp hdfs://nn1:8020/foo/a \
  
         
         
hdfs://nn1:8020/foo/b \
  
         
         
hdfs://nn2:8020/bar/foo

Or, equivalently, from a file using the -f option:  
bash$ hadoop distcp -f hdfs://nn1:8020/srclist \
  
          
          
 hdfs://nn2:8020/bar/foo

Where srclist contains  
    hdfs://nn1:8020/foo/a
  
    hdfs://nn1:8020/foo/b

When copying from multiple sources, DistCp will abort the copy with
an error message if two sources collide, but collisions at the
destination are resolved per the options
specified. By default, files already existing at the destination are
skipped (i.e. not replaced by the source file). A count of skipped
files is reported at the end of each job, but it may be inaccurate if a
copier failed for some subset of its files, but succeeded on a later
attempt (see Appendix).

It is important that each TaskTracker can reach and communicate with
both the source and destination file systems. For HDFS, both the source
and destination must be running the same version of the protocol or use
a backwards-compatible protocol (see Copying Between
Versions).

After a copy, it is recommended that one generates and cross-checks
a listing of the source and destination to verify that the copy was
truly successful. Since DistCp employs both Map/Reduce and the
FileSystem API, issues in or between any of the three could adversely
and silently affect the copy. Some have had success running with
-update enabled to perform a second pass, but users should
be acquainted with its semantics before attempting this.

It's also worth noting that if another client is still writing to a
source file, the copy will likely fail. Attempting to overwrite a file
being written at the destination should also fail on HDFS. If a source
file is (re)moved before it is copied, the copy will fail with a
FileNotFoundException.

### Options

#### Option Index

| Flag | Description | Notes |
| --- | --- | --- |
| -p[rbugp] | Preserve    r: replication number    b: block size    u: user    g: group    p: permission | Modification times are not preserved. Also, when -update is specified, status updates will **not** be synchronized unless the file sizes also differ (i.e. unless the file is re-created). |
| -i | Ignore failures | As explained in the Appendix, this option will keep more accurate statistics about the copy than the default case. It also preserves logs from failed copies, which can be valuable for debugging. Finally, a failing map will not cause the job to fail before all splits are attempted. |
| -log <logdir> | Write logs to <logdir> | DistCp keeps logs of each file it attempts to copy as map output. If a map fails, the log output will not be retained if it is re-executed. |
| -m <num\_maps> | Maximum number of simultaneous copies | Specify the number of maps to copy data. Note that more maps may not necessarily improve throughput. |
| -overwrite | Overwrite destination | If a map fails and -i is not specified, all the files in the split, not only those that failed, will be recopied. As discussed in the following, it also changes the semantics for generating destination paths, so users should use this carefully. |
| -update | Overwrite if src size different from dst size | As noted in the preceding, this is not a "sync" operation. The only criterion examined is the source and destination file sizes; if they differ, the source file replaces the destination file. As discussed in the following, it also changes the semantics for generating destination paths, so users should use this carefully. |
| -f <urilist\_uri> | Use list at <urilist\_uri> as src list | This is equivalent to listing each source on the command line. The urilist\_uri list should be a fully qualified URI. |

#### Update and Overwrite

It's worth giving some examples of -update and
-overwrite. Consider a copy from /foo/a and
/foo/b to /bar/foo, where the sources contain
the following:

    hdfs://nn1:8020/foo/a
  
    hdfs://nn1:8020/foo/a/aa
  
    hdfs://nn1:8020/foo/a/ab
  
    hdfs://nn1:8020/foo/b
  
    hdfs://nn1:8020/foo/b/ba
  
    hdfs://nn1:8020/foo/b/ab

If either -update or -overwrite is set,
then both sources will map an entry to /bar/foo/ab at the
destination. For both options, the contents of each source directory
are compared with the **contents** of the destination
directory. Rather than permit this conflict, DistCp will abort.

In the default case, both /bar/foo/a and
/bar/foo/b will be created and neither will collide.

Now consider a legal copy using -update:  
distcp -update hdfs://nn1:8020/foo/a \
  
       
      
hdfs://nn1:8020/foo/b \
  
       
      
hdfs://nn2:8020/bar

With sources/sizes:

    hdfs://nn1:8020/foo/a
  
    hdfs://nn1:8020/foo/a/aa 32
  
    hdfs://nn1:8020/foo/a/ab 32
  
    hdfs://nn1:8020/foo/b
  
    hdfs://nn1:8020/foo/b/ba 64
  
    hdfs://nn1:8020/foo/b/bb 32

And destination/sizes:

    hdfs://nn2:8020/bar
  
    hdfs://nn2:8020/bar/aa 32
  
    hdfs://nn2:8020/bar/ba 32
  
    hdfs://nn2:8020/bar/bb 64

Will effect:

    hdfs://nn2:8020/bar
  
    hdfs://nn2:8020/bar/aa 32
  
    hdfs://nn2:8020/bar/ab 32
  
    hdfs://nn2:8020/bar/ba 64
  
    hdfs://nn2:8020/bar/bb 32

Only aa is not overwritten on nn2. If
-overwrite were specified, all elements would be
overwritten.


## Appendix

### Map sizing

DistCp makes a faint attempt to size each map comparably so that
each copies roughly the same number of bytes. Note that files are the
finest level of granularity, so increasing the number of simultaneous
copiers (i.e. maps) may not always increase the number of
simultaneous copies nor the overall throughput.

If -m is not specified, DistCp will attempt to
schedule work for min (total\_bytes / bytes.per.map, 20 \*
num\_task\_trackers) where bytes.per.map defaults
to 256MB.

Tuning the number of maps to the size of the source and
destination clusters, the size of the copy, and the available
bandwidth is recommended for long-running and regularly run jobs.

### Copying between versions of HDFS

For copying between two different versions of Hadoop, one will
usually use HftpFileSystem. This is a read-only FileSystem, so DistCp
must be run on the destination cluster (more specifically, on
TaskTrackers that can write to the destination cluster). Each source is
specified as hftp://<dfs.http.address>/<path>
(the default dfs.http.address is
<namenode>:50070).

### Map/Reduce and other side-effects

As has been mentioned in the preceding, should a map fail to copy
one of its inputs, there will be several side-effects.

- Unless -i is specified, the logs generated by that
  task attempt will be replaced by the previous attempt.
- Unless -overwrite is specified, files successfully
  copied by a previous map on a re-execution will be marked as
  "skipped".
- If a map fails mapred.map.max.attempts times, the
  remaining map tasks will be killed (unless -i is
  set).
- If mapred.speculative.execution is set set
  final and true, the result of the copy is
  undefined.

Copyright ©
2007 The Apache Software Foundation.
