# Supplementary material for: Provenance based data integrity checking and verification in cloud environments
Source: PLoS One. 2017 May 17;12(5):e0177576. doi: 10.1371/journal.pone.0177576 (PMC5435237; doi:10.1371/journal.pone.0177576)
Supplement: S1 Dataset — Files in the Dataset are utilized for performance measurement. (ZIP) [file pone.0177576.s001.zip › dataset/html/hadoop-default - Copy (2) - Copy.html]

|  |  |  |
| --- | --- | --- |
| name | value | description |
| hadoop.tmp.dir | /tmp/hadoop-${user.name} | A base for other temporary directories. |
| hadoop.native.lib | true | Should native hadoop libraries, if present, be used. |
| hadoop.logfile.size | 10000000 | The max size of each log file |
| hadoop.logfile.count | 10 | The max number of log files |
| hadoop.job.history.location |  | If job tracker is static the history files are stored in this single well known place. If No value is set here, by default, it is in the local file system at ${hadoop.log.dir}/history. |
| hadoop.job.history.user.location |  | User can specify a location to store the history files of a particular job. If nothing is specified, the logs are stored in output directory. The files are stored in "\_logs/history/" in the directory. User can stop logging by giving the value "none". |
| dfs.namenode.logging.level | info | The logging level for dfs namenode. Other values are "dir"(trac e namespace mutations), "block"(trace block under/over replications and block creations/deletions), or "all". |
| io.sort.factor | 10 | The number of streams to merge at once while sorting files. This determines the number of open file handles. |
| io.sort.mb | 100 | The total amount of buffer memory to use while sorting files, in megabytes. By default, gives each merge stream 1MB, which should minimize seeks. |
| io.sort.record.percent | 0.05 | The percentage of io.sort.mb dedicated to tracking record boundaries. Let this value be r, io.sort.mb be x. The maximum number of records collected before the collection thread must block is equal to (r \* x) / 4 |
| io.sort.spill.percent | 0.80 | The soft limit in either the buffer or record collection buffers. Once reached, a thread will begin to spill the contents to disk in the background. Note that this does not imply any chunking of data to the spill. A value less than 0.5 is not recommended. |
| io.file.buffer.size | 4096 | The size of buffer for use in sequence files. The size of this buffer should probably be a multiple of hardware page size (4096 on Intel x86), and it determines how much data is buffered during read and write operations. |
| io.bytes.per.checksum | 512 | The number of bytes per checksum. Must not be larger than io.file.buffer.size. |
| io.skip.checksum.errors | false | If true, when a checksum error is encountered while reading a sequence file, entries are skipped, instead of throwing an exception. |
| io.map.index.skip | 0 | Number of index entries to skip between each entry. Zero by default. Setting this to values larger than zero can facilitate opening large map files using less memory. |
| io.compression.codecs | org.apache.hadoop.io.compress.DefaultCodec,org.apache.hadoop.io.compress.GzipCodec | A list of the compression codec classes that can be used for compression/decompression. |
| io.serializations | org.apache.hadoop.io.serializer.WritableSerialization | A list of serialization classes that can be used for obtaining serializers and deserializers. |
| fs.default.name | file:/// | The name of the default file system. A URI whose scheme and authority determine the FileSystem implementation. The uri's scheme determines the config property (fs.SCHEME.impl) naming the FileSystem implementation class. The uri's authority is used to determine the host, port, etc. for a filesystem. |
| fs.trash.interval | 0 | Number of minutes between trash checkpoints. If zero, the trash feature is disabled. |
| fs.file.impl | org.apache.hadoop.fs.LocalFileSystem | The FileSystem for file: uris. |
| fs.hdfs.impl | org.apache.hadoop.dfs.DistributedFileSystem | The FileSystem for hdfs: uris. |
| fs.s3.impl | org.apache.hadoop.fs.s3.S3FileSystem | The FileSystem for s3: uris. |
| fs.s3n.impl | org.apache.hadoop.fs.s3native.NativeS3FileSystem | The FileSystem for s3n: (Native S3) uris. |
| fs.kfs.impl | org.apache.hadoop.fs.kfs.KosmosFileSystem | The FileSystem for kfs: uris. |
| fs.hftp.impl | org.apache.hadoop.dfs.HftpFileSystem |  |
| fs.hsftp.impl | org.apache.hadoop.dfs.HsftpFileSystem |  |
| fs.ftp.impl | org.apache.hadoop.fs.ftp.FTPFileSystem | The FileSystem for ftp: uris. |
| fs.ramfs.impl | org.apache.hadoop.fs.InMemoryFileSystem | The FileSystem for ramfs: uris. |
| fs.har.impl | org.apache.hadoop.fs.HarFileSystem | The filesystem for Hadoop archives. |
| fs.inmemory.size.mb | 75 | The size of the in-memory filsystem instance in MB |
| fs.checkpoint.dir | ${hadoop.tmp.dir}/dfs/namesecondary | Determines where on the local filesystem the DFS secondary name node should store the temporary images and edits to merge. If this is a comma-delimited list of directories then the image is replicated in all of the directories for redundancy. |
| fs.checkpoint.period | 3600 | The number of seconds between two periodic checkpoints. |
| fs.checkpoint.size | 67108864 | The size of the current edit log (in bytes) that triggers a periodic checkpoint even if the fs.checkpoint.period hasn't expired. |
| dfs.secondary.http.address | 0.0.0.0:50090 | The secondary namenode http server address and port. If the port is 0 then the server will start on a free port. |
| dfs.datanode.address | 0.0.0.0:50010 | The address where the datanode server will listen to. If the port is 0 then the server will start on a free port. |
| dfs.datanode.http.address | 0.0.0.0:50075 | The datanode http server address and port. If the port is 0 then the server will start on a free port. |
| dfs.datanode.ipc.address | 0.0.0.0:50020 | The datanode ipc server address and port. If the port is 0 then the server will start on a free port. |
| dfs.datanode.handler.count | 3 | The number of server threads for the datanode. |
| dfs.http.address | 0.0.0.0:50070 | The address and the base port where the dfs namenode web ui will listen on. If the port is 0 then the server will start on a free port. |
| dfs.datanode.https.address | 0.0.0.0:50475 |  |
| dfs.https.address | 0.0.0.0:50470 |  |
| https.keystore.info.rsrc | sslinfo.xml | The name of the resource from which ssl keystore information will be extracted |
| dfs.datanode.dns.interface | default | The name of the Network Interface from which a data node should report its IP address. |
| dfs.datanode.dns.nameserver | default | The host name or IP address of the name server (DNS) which a DataNode should use to determine the host name used by the NameNode for communication and display purposes. |
| dfs.replication.considerLoad | true | Decide if chooseTarget considers the target's load or not |
| dfs.default.chunk.view.size | 32768 | The number of bytes to view for a file on the browser. |
| dfs.datanode.du.reserved | 0 | Reserved space in bytes per volume. Always leave this much space free for non dfs use. |
| dfs.datanode.du.pct | 0.98f | When calculating remaining space, only use this percentage of the real available space |
| dfs.name.dir | ${hadoop.tmp.dir}/dfs/name | Determines where on the local filesystem the DFS name node should store the name table. If this is a comma-delimited list of directories then the name table is replicated in all of the directories, for redundancy. |
| dfs.web.ugi | webuser,webgroup | The user account used by the web interface. Syntax: USERNAME,GROUP1,GROUP2, ... |
| dfs.permissions | true | If "true", enable permission checking in HDFS. If "false", permission checking is turned off, but all other behavior is unchanged. Switching from one parameter value to the other does not change the mode, owner or group of files or directories. |
| dfs.permissions.supergroup | supergroup | The name of the group of super-users. |
| dfs.client.buffer.dir | ${hadoop.tmp.dir}/dfs/tmp | Determines where on the local filesystem an DFS client should store its blocks before it sends them to the datanode. |
| dfs.data.dir | ${hadoop.tmp.dir}/dfs/data | Determines where on the local filesystem an DFS data node should store its blocks. If this is a comma-delimited list of directories, then data will be stored in all named directories, typically on different devices. Directories that do not exist are ignored. |
| dfs.replication | 3 | Default block replication. The actual number of replications can be specified when the file is created. The default is used if replication is not specified in create time. |
| dfs.replication.max | 512 | Maximal block replication. |
| dfs.replication.min | 1 | Minimal block replication. |
| dfs.block.size | 67108864 | The default block size for new files. |
| dfs.df.interval | 60000 | Disk usage statistics refresh interval in msec. |
| dfs.client.block.write.retries | 3 | The number of retries for writing blocks to the data nodes, before we signal failure to the application. |
| dfs.blockreport.intervalMsec | 3600000 | Determines block reporting interval in milliseconds. |
| dfs.blockreport.initialDelay | 0 | Delay for first block report in seconds. |
| dfs.heartbeat.interval | 3 | Determines datanode heartbeat interval in seconds. |
| dfs.namenode.handler.count | 10 | The number of server threads for the namenode. |
| dfs.safemode.threshold.pct | 0.999f | Specifies the percentage of blocks that should satisfy the minimal replication requirement defined by dfs.replication.min. Values less than or equal to 0 mean not to start in safe mode. Values greater than 1 will make safe mode permanent. |
| dfs.safemode.extension | 30000 | Determines extension of safe mode in milliseconds after the threshold level is reached. |
| dfs.balance.bandwidthPerSec | 1048576 | Specifies the maximum amount of bandwidth that each datanode can utilize for the balancing purpose in term of the number of bytes per second. |
| dfs.hosts |  | Names a file that contains a list of hosts that are permitted to connect to the namenode. The full pathname of the file must be specified. If the value is empty, all hosts are permitted. |
| dfs.hosts.exclude |  | Names a file that contains a list of hosts that are not permitted to connect to the namenode. The full pathname of the file must be specified. If the value is empty, no hosts are excluded. |
| dfs.max.objects | 0 | The maximum number of files, directories and blocks dfs supports. A value of zero indicates no limit to the number of objects that dfs supports. |
| dfs.namenode.decommission.interval | 300 | Namenode periodicity in seconds to check if decommission is complete. |
| dfs.replication.interval | 3 | The periodicity in seconds with which the namenode computes repliaction work for datanodes. |
| fs.s3.block.size | 67108864 | Block size to use when writing files to S3. |
| fs.s3.buffer.dir | ${hadoop.tmp.dir}/s3 | Determines where on the local filesystem the S3 filesystem should store files before sending them to S3 (or after retrieving them from S3). |
| fs.s3.maxRetries | 4 | The maximum number of retries for reading or writing files to S3, before we signal failure to the application. |
| fs.s3.sleepTimeSeconds | 10 | The number of seconds to sleep between each S3 retry. |
| mapred.job.tracker | local | The host and port that the MapReduce job tracker runs at. If "local", then jobs are run in-process as a single map and reduce task. |
| mapred.job.tracker.http.address | 0.0.0.0:50030 | The job tracker http server address and port the server will listen on. If the port is 0 then the server will start on a free port. |
| mapred.job.tracker.handler.count | 10 | The number of server threads for the JobTracker. This should be roughly 4% of the number of tasktracker nodes. |
| mapred.task.tracker.report.address | 127.0.0.1:0 | The interface and port that task tracker server listens on. Since it is only connected to by the tasks, it uses the local interface. EXPERT ONLY. Should only be changed if your host does not have the loopback interface. |
| mapred.local.dir | ${hadoop.tmp.dir}/mapred/local | The local directory where MapReduce stores intermediate data files. May be a comma-separated list of directories on different devices in order to spread disk i/o. Directories that do not exist are ignored. |
| local.cache.size | 10737418240 | The limit on the size of cache you want to keep, set by default to 10GB. This will act as a soft limit on the cache directory for out of band data. |
| mapred.system.dir | ${hadoop.tmp.dir}/mapred/system | The shared directory where MapReduce stores control files. |
| mapred.temp.dir | ${hadoop.tmp.dir}/mapred/temp | A shared directory for temporary files. |
| mapred.local.dir.minspacestart | 0 | If the space in mapred.local.dir drops under this, do not ask for more tasks. Value in bytes. |
| mapred.local.dir.minspacekill | 0 | If the space in mapred.local.dir drops under this, do not ask more tasks until all the current ones have finished and cleaned up. Also, to save the rest of the tasks we have running, kill one of them, to clean up some space. Start with the reduce tasks, then go with the ones that have finished the least. Value in bytes. |
| mapred.tasktracker.expiry.interval | 600000 | Expert: The time-interval, in miliseconds, after which a tasktracker is declared 'lost' if it doesn't send heartbeats. |
| mapred.map.tasks | 2 | The default number of map tasks per job. Typically set to a prime several times greater than number of available hosts. Ignored when mapred.job.tracker is "local". |
| mapred.reduce.tasks | 1 | The default number of reduce tasks per job. Typically set to a prime close to the number of available hosts. Ignored when mapred.job.tracker is "local". |
| mapred.map.max.attempts | 4 | Expert: The maximum number of attempts per map task. In other words, framework will try to execute a map task these many number of times before giving up on it. |
| mapred.reduce.max.attempts | 4 | Expert: The maximum number of attempts per reduce task. In other words, framework will try to execute a reduce task these many number of times before giving up on it. |
| mapred.reduce.parallel.copies | 5 | The default number of parallel transfers run by reduce during the copy(shuffle) phase. |
| mapred.reduce.copy.backoff | 300 | The maximum amount of time (in seconds) a reducer spends on fetching one map output before declaring it as failed. |
| mapred.task.timeout | 600000 | The number of milliseconds before a task will be terminated if it neither reads an input, writes an output, nor updates its status string. |
| mapred.tasktracker.map.tasks.maximum | 2 | The maximum number of map tasks that will be run simultaneously by a task tracker. |
| mapred.tasktracker.reduce.tasks.maximum | 2 | The maximum number of reduce tasks that will be run simultaneously by a task tracker. |
| mapred.jobtracker.completeuserjobs.maximum | 100 | The maximum number of complete jobs per user to keep around before delegating them to the job history. |
| mapred.child.java.opts | -Xmx200m | Java opts for the task tracker child processes. The following symbol, if present, will be interpolated: @taskid@ is replaced by current TaskID. Any other occurrences of '@' will go unchanged. For example, to enable verbose gc logging to a file named for the taskid in /tmp and to set the heap maximum to be a gigabyte, pass a 'value' of: -Xmx1024m -verbose:gc -Xloggc:/tmp/@taskid@.gc The configuration variable mapred.child.ulimit can be used to control the maximum virtual memory of the child processes. |
| mapred.child.ulimit |  | The maximum virtual memory, in KB, of a process launched by the Map-Reduce framework. This can be used to control both the Mapper/Reducer tasks and applications using Hadoop Pipes, Hadoop Streaming etc. By default it is left unspecified to let cluster admins control it via limits.conf and other such relevant mechanisms. Note: mapred.child.ulimit must be greater than or equal to the -Xmx passed to JavaVM, else the VM might not start. |
| mapred.child.tmp | ./tmp | To set the value of tmp directory for map and reduce tasks. If the value is an absolute path, it is directly assigned. Otherwise, it is prepended with task's working directory. The java tasks are executed with option -Djava.io.tmpdir='the absolute path of the tmp dir'. Pipes and streaming are set with environment variable, TMPDIR='the absolute path of the tmp dir' |
| mapred.inmem.merge.threshold | 1000 | The threshold, in terms of the number of files for the in-memory merge process. When we accumulate threshold number of files we initiate the in-memory merge and spill to disk. A value of 0 or less than 0 indicates we want to DON'T have any threshold and instead depend only on the ramfs's memory consumption to trigger the merge. |
| mapred.map.tasks.speculative.execution | true | If true, then multiple instances of some map tasks may be executed in parallel. |
| mapred.reduce.tasks.speculative.execution | true | If true, then multiple instances of some reduce tasks may be executed in parallel. |
| mapred.min.split.size | 0 | The minimum size chunk that map input should be split into. Note that some file formats may have minimum split sizes that take priority over this setting. |
| mapred.submit.replication | 10 | The replication level for submitted job files. This should be around the square root of the number of nodes. |
| mapred.tasktracker.dns.interface | default | The name of the Network Interface from which a task tracker should report its IP address. |
| mapred.tasktracker.dns.nameserver | default | The host name or IP address of the name server (DNS) which a TaskTracker should use to determine the host name used by the JobTracker for communication and display purposes. |
| tasktracker.http.threads | 40 | The number of worker threads that for the http server. This is used for map output fetching |
| mapred.task.tracker.http.address | 0.0.0.0:50060 | The task tracker http server address and port. If the port is 0 then the server will start on a free port. |
| keep.failed.task.files | false | Should the files for failed tasks be kept. This should only be used on jobs that are failing, because the storage is never reclaimed. It also prevents the map outputs from being erased from the reduce directory as they are consumed. |
| mapred.output.compress | false | Should the job outputs be compressed? |
| mapred.output.compression.type | RECORD | If the job outputs are to compressed as SequenceFiles, how should they be compressed? Should be one of NONE, RECORD or BLOCK. |
| mapred.output.compression.codec | org.apache.hadoop.io.compress.DefaultCodec | If the job outputs are compressed, how should they be compressed? |
| mapred.compress.map.output | false | Should the outputs of the maps be compressed before being sent across the network. Uses SequenceFile compression. |
| mapred.map.output.compression.codec | org.apache.hadoop.io.compress.DefaultCodec | If the map outputs are compressed, how should they be compressed? |
| io.seqfile.compress.blocksize | 1000000 | The minimum block size for compression in block compressed SequenceFiles. |
| io.seqfile.lazydecompress | true | Should values of block-compressed SequenceFiles be decompressed only when necessary. |
| io.seqfile.sorter.recordlimit | 1000000 | The limit on number of records to be kept in memory in a spill in SequenceFiles.Sorter |
| map.sort.class | org.apache.hadoop.util.QuickSort | The default sort class for sorting keys. |
| mapred.userlog.limit.kb | 0 | The maximum size of user-logs of each task in KB. 0 disables the cap. |
| mapred.userlog.retain.hours | 24 | The maximum time, in hours, for which the user-logs are to be retained. |
| mapred.hosts |  | Names a file that contains the list of nodes that may connect to the jobtracker. If the value is empty, all hosts are permitted. |
| mapred.hosts.exclude |  | Names a file that contains the list of hosts that should be excluded by the jobtracker. If the value is empty, no hosts are excluded. |
| mapred.max.tracker.failures | 4 | The number of task-failures on a tasktracker of a given job after which new tasks of that job aren't assigned to it. |
| jobclient.output.filter | FAILED | The filter for controlling the output of the task's userlogs sent to the console of the JobClient. The permissible options are: NONE, KILLED, FAILED, SUCCEEDED and ALL. |
| mapred.job.tracker.persist.jobstatus.active | false | Indicates if persistency of job status information is active or not. |
| mapred.job.tracker.persist.jobstatus.hours | 0 | The number of hours job status information is persisted in DFS. The job status information will be available after it drops of the memory queue and between jobtracker restarts. With a zero value the job status information is not persisted at all in DFS. |
| mapred.job.tracker.persist.jobstatus.dir | /jobtracker/jobsInfo | The directory where the job status information is persisted in a file system to be available after it drops of the memory queue and between jobtracker restarts. |
| mapred.task.profile | false | To set whether the system should collect profiler information for some of the tasks in this job? The information is stored in the the user log directory. The value is "true" if task profiling is enabled. |
| mapred.task.profile.maps | 0-2 | To set the ranges of map tasks to profile. mapred.task.profile has to be set to true for the value to be accounted. |
| mapred.task.profile.reduces | 0-2 | To set the ranges of reduce tasks to profile. mapred.task.profile has to be set to true for the value to be accounted. |
| mapred.line.input.format.linespermap | 1 | Number of lines per split in NLineInputFormat. |
| ipc.client.idlethreshold | 4000 | Defines the threshold number of connections after which connections will be inspected for idleness. |
| ipc.client.kill.max | 10 | Defines the maximum number of clients to disconnect in one go. |
| ipc.client.connection.maxidletime | 10000 | The maximum time in msec after which a client will bring down the connection to the server. |
| ipc.client.connect.max.retries | 10 | Indicates the number of retries a client will make to establish a server connection. |
| ipc.server.listen.queue.size | 128 | Indicates the length of the listen queue for servers accepting client connections. |
| ipc.server.tcpnodelay | false | Turn on/off Nagle's algorithm for the TCP socket connection on the server. Setting to true disables the algorithm and may decrease latency with a cost of more/smaller packets. |
| ipc.client.tcpnodelay | false | Turn on/off Nagle's algorithm for the TCP socket connection on the client. Setting to true disables the algorithm and may decrease latency with a cost of more/smaller packets. |
| job.end.retry.attempts | 0 | Indicates how many times hadoop should attempt to contact the notification URL |
| job.end.retry.interval | 30000 | Indicates time in milliseconds between notification URL retry calls |
| webinterface.private.actions | false | If set to true, the web interfaces of JT and NN may contain actions, such as kill job, delete file, etc., that should not be exposed to public. Enable this option if the interfaces are only reachable by those who have the right authorization. |
| hadoop.rpc.socket.factory.class.default | org.apache.hadoop.net.StandardSocketFactory | Default SocketFactory to use. This parameter is expected to be formatted as "package.FactoryClassName". |
| hadoop.rpc.socket.factory.class.ClientProtocol |  | SocketFactory to use to connect to a DFS. If null or empty, use hadoop.rpc.socket.class.default. This socket factory is also used by DFSClient to create sockets to DataNodes. |
| hadoop.rpc.socket.factory.class.JobSubmissionProtocol |  | SocketFactory to use to connect to a Map/Reduce master (JobTracker). If null or empty, then use hadoop.rpc.socket.class.default. |
| hadoop.socks.server |  | Address (host:port) of the SOCKS server to be used by the SocksSocketFactory. |
| topology.node.switch.mapping.impl | org.apache.hadoop.net.ScriptBasedMapping | The default implementation of the DNSToSwitchMapping. It invokes a script specified in topology.script.file.name to resolve node names. If the value for topology.script.file.name is not set, the default value of DEFAULT\_RACK is returned for all node names. |
| topology.script.file.name |  | The script name that should be invoked to resolve DNS names to NetworkTopology names. Example: the script would take host.foo.bar as an argument, and return /rack1 as the output. |
| topology.script.number.args | 20 | The max number of args that the script configured with topology.script.file.name should be run with. Each arg is an IP address. |
| mapred.task.cache.levels | 2 | This is the max level of the task cache. For example, if the level is 2, the tasks cached are at the host level and at the rack level. |
