# Supplementary material for: Provenance based data integrity checking and verification in cloud environments
Source: PLoS One. 2017 May 17;12(5):e0177576. doi: 10.1371/journal.pone.0177576 (PMC5435237; doi:10.1371/journal.pone.0177576)
Supplement: S1 Dataset — Files in the Dataset are utilized for performance measurement. (ZIP) [file pone.0177576.s001.zip › dataset/html/HadoopVersionAnnotation - Copy (2).html]

HadoopVersionAnnotation (Hadoop 0.18.0 API)


|  |  |  |
| --- | --- | --- |
| **Overview** \| **Package** \| **Class** \| **Use** \| **Tree** \| **Deprecated** \| **Index** \| **Help** | |  |
| PREV CLASS   NEXT CLASS | **FRAMES**    **NO FRAMES**     **All Classes** |  |
| SUMMARY: REQUIRED \| OPTIONAL | DETAIL: ELEMENT |  |


---


## org.apache.hadoop Annotation Type HadoopVersionAnnotation

---

``` @Retention(value=RUNTIME) @Target(value=PACKAGE) public @interface HadoopVersionAnnotation ```

A package attribute that captures the version of Hadoop that was compiled.

---

| **Required Element Summary** | |
| --- | --- |
| `String` | `date`             Get the date when Hadoop was compiled. |
| `String` | `revision`             Get the subversion revision. |
| `String` | `url`             Get the url for the subversion repository. |
| `String` | `user`             Get the username that compiled Hadoop. |
| `String` | `version`             Get the Hadoop version |

**Element Detail**

### version

```
public abstract String version
```

:   Get the Hadoop version

    :   **Returns:**: the version string "0.6.3-dev"

---


### user

```
public abstract String user
```

:   Get the username that compiled Hadoop.

---


### date

```
public abstract String date
```

:   Get the date when Hadoop was compiled.

    :   **Returns:**: the date in unix 'date' format

---


### url

```
public abstract String url
```

:   Get the url for the subversion repository.

---


### revision

```
public abstract String revision
```

:   Get the subversion revision.

    :   **Returns:**: the revision number as a string (eg. "451451")


---


|  |  |  |
| --- | --- | --- |
| **Overview** \| **Package** \| **Class** \| **Use** \| **Tree** \| **Deprecated** \| **Index** \| **Help** | |  |
| PREV CLASS   NEXT CLASS | **FRAMES**    **NO FRAMES**     **All Classes** |  |
| SUMMARY: REQUIRED \| OPTIONAL | DETAIL: ELEMENT |  |


---

Copyright © 2008 The Apache Software Foundation
