# Supplementary material for: Provenance based data integrity checking and verification in cloud environments
Source: PLoS One. 2017 May 17;12(5):e0177576. doi: 10.1371/journal.pone.0177576 (PMC5435237; doi:10.1371/journal.pone.0177576)
Supplement: S1 Dataset — Files in the Dataset are utilized for performance measurement. (ZIP) [file pone.0177576.s001.zip › dataset/html/hadoop_archives - Copy (2) - Copy.html]

Hadoop Archives


Apache > Hadoop > Core

- Project
- Wiki
- Hadoop 0.18 Documentation

Documentation

Overview

Quickstart

Cluster Setup

HDFS Architecture

HDFS User Guide

HDFS Permissions Guide

HDFS Quotas Administrator Guide

Commands Manual

FS Shell Guide

DistCp Guide

Map-Reduce Tutorial

Native Hadoop Libraries

Streaming

Hadoop Archives

Hadoop On Demand

API Docs

Wiki

FAQ

Mailing Lists

Release Notes

All Changes


PDF

# Hadoop Archives

- What are Hadoop archives?
- How to create an archive?
- How to look up files in archives?

## What are Hadoop archives?

Hadoop archives are special format archives. A Hadoop archive
maps to a file system directory. A Hadoop archive always has a \*.har
extension. A Hadoop archive directory contains metadata (in the form
of \_index and \_masterindex) and data (part-\*) files. The \_index file contains
the name of the files that are part of the archive and the location
within the part files.

## How to create an archive?

Usage: hadoop archive -archiveName name <src>\* <dest>

-archiveName is the name of the archive you would like to create.
An example would be foo.har. The name should have a \*.har extension.
The inputs are file system pathnames which work as usual with regular
expressions. The destination directory would contain the archive.
Note that this is a Map/Reduce job that creates the archives. You would
need a map reduce cluster to run this. The following is an example:

hadoop archive -archiveName foo.har /user/hadoop/dir1 /user/hadoop/dir2 /user/zoo/

In the above example /user/hadoop/dir1 and /user/hadoop/dir2 will be
archived in the following file system directory -- /user/zoo/foo.har.
The sources are not changed or removed when an archive is created.

## How to look up files in archives?

The archive exposes itself as a file system layer. So all the fs shell
commands in the archives work but with a different URI. Also, note that
archives are immutable. So, rename's, deletes and creates return
an error. URI for Hadoop Archives is

har://scheme-hostname:port/archivepath/fileinarchive

If no scheme is provided it assumes the underlying filesystem.
In that case the URI would look like

har:///archivepath/fileinarchive

Here is an example of archive. The input to the archives is /dir. The directory dir contains
files filea, fileb. To archive /dir to /user/hadoop/foo.har, the command is

hadoop archive -archiveName foo.har /dir /user/hadoop

To get file listing for files in the created archive

hadoop dfs -lsr har:///user/hadoop/foo.har

To cat filea in archive -

hadoop dfs -cat har:///user/hadoop/foo.har/dir/filea

Copyright ©
2007 The Apache Software Foundation.
