# Supplementary material for: Provenance based data integrity checking and verification in cloud environments
Source: PLoS One. 2017 May 17;12(5):e0177576. doi: 10.1371/journal.pone.0177576 (PMC5435237; doi:10.1371/journal.pone.0177576)
Supplement: S1 Dataset — Files in the Dataset are utilized for performance measurement. (ZIP) [file pone.0177576.s001.zip › dataset/html/hdfs_design - Copy (2) - Copy.html]

The Hadoop Distributed File System: Architecture and Design


Apache > Hadoop > Core

- Project
- Wiki
- Hadoop 0.18 Documentation

Documentation

Overview

Quickstart

Cluster Setup

HDFS Architecture

HDFS User Guide

HDFS Permissions Guide

HDFS Quotas Administrator Guide

Commands Manual

FS Shell Guide

DistCp Guide

Map-Reduce Tutorial

Native Hadoop Libraries

Streaming

Hadoop Archives

Hadoop On Demand

API Docs

Wiki

FAQ

Mailing Lists

Release Notes

All Changes


PDF

# The Hadoop Distributed File System: Architecture and Design

- Introduction
- Assumptions and Goals 
  - Hardware Failure
  - Streaming Data Access
  - Large Data Sets
  - Simple Coherency Model
  - “Moving Computation is Cheaper than Moving Data”
  - Portability Across Heterogeneous Hardware and Software Platforms
- NameNode and DataNodes
- The File System Namespace
- Data Replication 
  - Replica Placement: The First Baby Steps
  - Replica Selection
  - Safemode
- The Persistence of File System Metadata
- The Communication Protocols
- Robustness 
  - Data Disk Failure, Heartbeats and Re-Replication
  - Cluster Rebalancing
  - Data Integrity
  - Metadata Disk Failure
  - Snapshots
- Data Organization 
  - Data Blocks
  - Staging
  - Replication Pipelining
- Accessibility 
  - FS Shell
  - DFSAdmin
  - Browser Interface
- Space Reclamation 
  - File Deletes and Undeletes
  - Decrease Replication Factor
- References

## Introduction

The Hadoop Distributed File System (HDFS) is a distributed file system designed to run on commodity hardware. It has many similarities with existing distributed file systems. However, the differences from other distributed file systems are significant. HDFS is highly fault-tolerant and is designed to be deployed on low-cost hardware. HDFS provides high throughput access to application data and is suitable for applications that have large data sets. HDFS relaxes a few POSIX requirements to enable streaming access to file system data. HDFS was originally built as infrastructure for the Apache Nutch web search engine project. HDFS is part of the Apache Hadoop Core project. The project URL is http://hadoop.apache.org/core/.

## Assumptions and Goals

### Hardware Failure

Hardware failure is the norm rather than the exception. An HDFS instance may consist of hundreds or thousands of server machines, each storing part of the file system’s data. The fact that there are a huge number of components and that each component has a non-trivial probability of failure means that some component of HDFS is always non-functional. Therefore, detection of faults and quick, automatic recovery from them is a core architectural goal of HDFS.

### Streaming Data Access

Applications that run on HDFS need streaming access to their data sets. They are not general purpose applications that typically run on general purpose file systems. HDFS is designed more for batch processing rather than interactive use by users. The emphasis is on high throughput of data access rather than low latency of data access. POSIX imposes many hard requirements that are not needed for applications that are targeted for HDFS. POSIX semantics in a few key areas has been traded to increase data throughput rates.

### Large Data Sets

Applications that run on HDFS have large data sets. A typical file in HDFS is gigabytes to terabytes in size. Thus, HDFS is tuned to support large files. It should provide high aggregate data bandwidth and scale to hundreds of nodes in a single cluster. It should support tens of millions of files in a single instance.

### Simple Coherency Model

HDFS applications need a write-once-read-many access model for files. A file once created, written, and closed need not be changed. This assumption simplifies data coherency issues and enables high throughput data access. A Map/Reduce application or a web crawler application fits perfectly with this model. There is a plan to support appending-writes to files in the future.

### “Moving Computation is Cheaper than Moving Data”

A computation requested by an application is much more efficient if it is executed near the data it operates on. This is especially true when the size of the data set is huge. This minimizes network congestion and increases the overall throughput of the system. The assumption is that it is often better to migrate the computation closer to where the data is located rather than moving the data to where the application is running. HDFS provides interfaces for applications to move themselves closer to where the data is located.

### Portability Across Heterogeneous Hardware and Software Platforms

HDFS has been designed to be easily portable from one platform to another. This facilitates widespread adoption of HDFS as a platform of choice for a large set of applications.

## NameNode and DataNodes

HDFS has a master/slave architecture. An HDFS cluster consists of a single NameNode, a master server that manages the file system namespace and regulates access to files by clients. In addition, there are a number of DataNodes, usually one per node in the cluster, which manage storage attached to the nodes that they run on. HDFS exposes a file system namespace and allows user data to be stored in files. Internally, a file is split into one or more blocks and these blocks are stored in a set of DataNodes. The NameNode executes file system namespace operations like opening, closing, and renaming files and directories. It also determines the mapping of blocks to DataNodes. The DataNodes are responsible for serving read and write requests from the file system’s clients. The DataNodes also perform block creation, deletion, and replication upon instruction from the NameNode.

The NameNode and DataNode are pieces of software designed to run on commodity machines. These machines typically run a GNU/Linux operating system (OS). HDFS is built using the Java language; any machine that supports Java can run the NameNode or the DataNode software. Usage of the highly portable Java language means that HDFS can be deployed on a wide range of machines. A typical deployment has a dedicated machine that runs only the NameNode software. Each of the other machines in the cluster runs one instance of the DataNode software. The architecture does not preclude running multiple DataNodes on the same machine but in a real deployment that is rarely the case.

The existence of a single NameNode in a cluster greatly simplifies the architecture of the system. The NameNode is the arbitrator and repository for all HDFS metadata. The system is designed in such a way that user data never flows through the NameNode.

## The File System Namespace

HDFS supports a traditional hierarchical file organization. A user or an application can create directories and store files inside these directories. The file system namespace hierarchy is similar to most other existing file systems; one can create and remove files, move a file from one directory to another, or rename a file. HDFS does not yet implement user quotas or access permissions. HDFS does not support hard links or soft links. However, the HDFS architecture does not preclude implementing these features.

The NameNode maintains the file system namespace. Any change to the file system namespace or its properties is recorded by the NameNode. An application can specify the number of replicas of a file that should be maintained by HDFS. The number of copies of a file is called the replication factor of that file. This information is stored by the NameNode.

## Data Replication

HDFS is designed to reliably store very large files across machines in a large cluster. It stores each file as a sequence of blocks; all blocks in a file except the last block are the same size. The blocks of a file are replicated for fault tolerance. The block size and replication factor are configurable per file. An application can specify the number of replicas of a file. The replication factor can be specified at file creation time and can be changed later. Files in HDFS are write-once and have strictly one writer at any time.

The NameNode makes all decisions regarding replication of blocks. It periodically receives a Heartbeat and a Blockreport from each of the DataNodes in the cluster. Receipt of a Heartbeat implies that the DataNode is functioning properly. A Blockreport contains a list of all blocks on a DataNode.

### Replica Placement: The First Baby Steps

The placement of replicas is critical to HDFS reliability and performance. Optimizing replica placement distinguishes HDFS from most other distributed file systems. This is a feature that needs lots of tuning and experience. The purpose of a rack-aware replica placement policy is to improve data reliability, availability, and network bandwidth utilization. The current implementation for the replica placement policy is a first effort in this direction. The short-term goals of implementing this policy are to validate it on production systems, learn more about its behavior, and build a foundation to test and research more sophisticated policies.

Large HDFS instances run on a cluster of computers that commonly spread across many racks. Communication between two nodes in different racks has to go through switches. In most cases, network bandwidth between machines in the same rack is greater than network bandwidth between machines in different racks.

The NameNode determines the rack id each DataNode belongs to via the process outlined in Rack Awareness. A simple but non-optimal policy is to place replicas on unique racks. This prevents losing data when an entire rack fails and allows use of bandwidth from multiple racks when reading data. This policy evenly distributes replicas in the cluster which makes it easy to balance load on component failure. However, this policy increases the cost of writes because a write needs to transfer blocks to multiple racks.

For the common case, when the replication factor is three, HDFS’s placement policy is to put one replica on one node in the local rack, another on a different node in the local rack, and the last on a different node in a different rack. This policy cuts the inter-rack write traffic which generally improves write performance. The chance of rack failure is far less than that of node failure; this policy does not impact data reliability and availability guarantees. However, it does reduce the aggregate network bandwidth used when reading data since a block is placed in only two unique racks rather than three. With this policy, the replicas of a file do not evenly distribute across the racks. One third of replicas are on one node, two thirds of replicas are on one rack, and the other third are evenly distributed across the remaining racks. This policy improves write performance without compromising data reliability or read performance.

The current, default replica placement policy described here is a work in progress.

### Replica Selection

To minimize global bandwidth consumption and read latency, HDFS tries to satisfy a read request from a replica that is closest to the reader. If there exists a replica on the same rack as the reader node, then that replica is preferred to satisfy the read request. If angg/ HDFS cluster spans multiple data centers, then a replica that is resident in the local data center is preferred over any remote replica.

### Safemode

On startup, the NameNode enters a special state called Safemode. Replication of data blocks does not occur when the NameNode is in the Safemode state. The NameNode receives Heartbeat and Blockreport messages from the DataNodes. A Blockreport contains the list of data blocks that a DataNode is hosting. Each block has a specified minimum number of replicas. A block is considered safely replicated when the minimum number of replicas of that data block has checked in with the NameNode. After a configurable percentage of safely replicated data blocks checks in with the NameNode (plus an additional 30 seconds), the NameNode exits the Safemode state. It then determines the list of data blocks (if any) that still have fewer than the specified number of replicas. The NameNode then replicates these blocks to other DataNodes.

## The Persistence of File System Metadata

The HDFS namespace is stored by the NameNode. The NameNode uses a transaction log called the EditLog to persistently record every change that occurs to file system metadata. For example, creating a new file in HDFS causes the NameNode to insert a record into the EditLog indicating this. Similarly, changing the replication factor of a file causes a new record to be inserted into the EditLog. The NameNode uses a file in its local host OS file system to store the EditLog. The entire file system namespace, including the mapping of blocks to files and file system properties, is stored in a file called the FsImage. The FsImage is stored as a file in the NameNode’s local file system too.

The NameNode keeps an image of the entire file system namespace and file Blockmap in memory. This key metadata item is designed to be compact, such that a NameNode with 4 GB of RAM is plenty to support a huge number of files and directories. When the NameNode starts up, it reads the FsImage and EditLog from disk, applies all the transactions from the EditLog to the in-memory representation of the FsImage, and flushes out this new version into a new FsImage on disk. It can then truncate the old EditLog because its transactions have been applied to the persistent FsImage. This process is called a checkpoint. In the current implementation, a checkpoint only occurs when the NameNode starts up. Work is in progress to support periodic checkpointing in the near future.

The DataNode stores HDFS data in files in its local file system. The DataNode has no knowledge about HDFS files. It stores each block of HDFS data in a separate file in its local file system. The DataNode does not create all files in the same directory. Instead, it uses a heuristic to determine the optimal number of files per directory and creates subdirectories appropriately. It is not optimal to create all local files in the same directory because the local file system might not be able to efficiently support a huge number of files in a single directory. When a DataNode starts up, it scans through its local file system, generates a list of all HDFS data blocks that correspond to each of these local files and sends this report to the NameNode: this is the Blockreport.

## The Communication Protocols

All HDFS communication protocols are layered on top of the TCP/IP protocol. A client establishes a connection to a configurable TCP port on the NameNode machine. It talks the ClientProtocol with the NameNode. The DataNodes talk to the NameNode using the DataNode Protocol. A Remote Procedure Call (RPC) abstraction wraps both the Client Protocol and the DataNode Protocol. By design, the NameNode never initiates any RPCs. Instead, it only responds to RPC requests issued by DataNodes or clients.

## Robustness

The primary objective of HDFS is to store data reliably even in the presence of failures. The three common types of failures are NameNode failures, DataNode failures and network partitions.

### Data Disk Failure, Heartbeats and Re-Replication

Each DataNode sends a Heartbeat message to the NameNode periodically. A network partition can cause a subset of DataNodes to lose connectivity with the NameNode. The NameNode detects this condition by the absence of a Heartbeat message. The NameNode marks DataNodes without recent Heartbeats as dead and does not forward any new IO requests to them. Any data that was registered to a dead DataNode is not available to HDFS any more. DataNode death may cause the replication factor of some blocks to fall below their specified value. The NameNode constantly tracks which blocks need to be replicated and initiates replication whenever necessary. The necessity for re-replication may arise due to many reasons: a DataNode may become unavailable, a replica may become corrupted, a hard disk on a DataNode may fail, or the replication factor of a file may be increased.

### Cluster Rebalancing

The HDFS architecture is compatible with data rebalancing schemes. A scheme might automatically move data from one DataNode to another if the free space on a DataNode falls below a certain threshold. In the event of a sudden high demand for a particular file, a scheme might dynamically create additional replicas and rebalance other data in the cluster. These types of data rebalancing schemes are not yet implemented.

### Data Integrity

It is possible that a block of data fetched from a DataNode arrives corrupted. This corruption can occur because of faults in a storage device, network faults, or buggy software. The HDFS client software implements checksum checking on the contents of HDFS files. When a client creates an HDFS file, it computes a checksum of each block of the file and stores these checksums in a separate hidden file in the same HDFS namespace. When a client retrieves file contents it verifies that the data it received from each DataNode matches the checksum stored in the associated checksum file. If not, then the client can opt to retrieve that block from another DataNode that has a replica of that block.

### Metadata Disk Failure

The FsImage and the EditLog are central data structures of HDFS. A corruption of these files can cause the HDFS instance to be non-functional. For this reason, the NameNode can be configured to support maintaining multiple copies of the FsImage and EditLog. Any update to either the FsImage or EditLog causes each of the FsImages and EditLogs to get updated synchronously. This synchronous updating of multiple copies of the FsImage and EditLog may degrade the rate of namespace transactions per second that a NameNode can support. However, this degradation is acceptable because even though HDFS applications are very data intensive in nature, they are not metadata intensive. When a NameNode restarts, it selects the latest consistent FsImage and EditLog to use.

The NameNode machine is a single point of failure for an HDFS cluster. If the NameNode machine fails, manual intervention is necessary. Currently, automatic restart and failover of the NameNode software to another machine is not supported.

### Snapshots

Snapshots support storing a copy of data at a particular instant of time. One usage of the snapshot feature may be to roll back a corrupted HDFS instance to a previously known good point in time. HDFS does not currently support snapshots but will in a future release.

## Data Organization

### Data Blocks

HDFS is designed to support very large files. Applications that are compatible with HDFS are those that deal with large data sets. These applications write their data only once but they read it one or more times and require these reads to be satisfied at streaming speeds. HDFS supports write-once-read-many semantics on files. A typical block size used by HDFS is 64 MB. Thus, an HDFS file is chopped up into 64 MB chunks, and if possible, each chunk will reside on a different DataNode.

### Staging

A client request to create a file does not reach the NameNode immediately. In fact, initially the HDFS client caches the file data into a temporary local file. Application writes are transparently redirected to this temporary local file. When the local file accumulates data worth over one HDFS block size, the client contacts the NameNode. The NameNode inserts the file name into the file system hierarchy and allocates a data block for it. The NameNode responds to the client request with the identity of the DataNode and the destination data block. Then the client flushes the block of data from the local temporary file to the specified DataNode. When a file is closed, the remaining un-flushed data in the temporary local file is transferred to the DataNode. The client then tells the NameNode that the file is closed. At this point, the NameNode commits the file creation operation into a persistent store. If the NameNode dies before the file is closed, the file is lost.

The above approach has been adopted after careful consideration of target applications that run on HDFS. These applications need streaming writes to files. If a client writes to a remote file directly without any client side buffering, the network speed and the congestion in the network impacts throughput considerably. This approach is not without precedent. Earlier distributed file systems, e.g. AFS, have used client side caching to improve performance. A POSIX requirement has been relaxed to achieve higher performance of data uploads.

### Replication Pipelining

When a client is writing data to an HDFS file, its data is first written to a local file as explained in the previous section. Suppose the HDFS file has a replication factor of three. When the local file accumulates a full block of user data, the client retrieves a list of DataNodes from the NameNode. This list contains the DataNodes that will host a replica of that block. The client then flushes the data block to the first DataNode. The first DataNode starts receiving the data in small portions (4 KB), writes each portion to its local repository and transfers that portion to the second DataNode in the list. The second DataNode, in turn starts receiving each portion of the data block, writes that portion to its repository and then flushes that portion to the third DataNode. Finally, the third DataNode writes the data to its local repository. Thus, a DataNode can be receiving data from the previous one in the pipeline and at the same time forwarding data to the next one in the pipeline. Thus, the data is pipelined from one DataNode to the next.

## Accessibility

HDFS can be accessed from applications in many different ways. Natively, HDFS provides a Java API for applications to use. A C language wrapper for this Java API is also available. In addition, an HTTP browser can also be used to browse the files of an HDFS instance. Work is in progress to expose HDFS through the WebDAV protocol.

### FS Shell

HDFS allows user data to be organized in the form of files and directories. It provides a commandline interface called FS shell that lets a user interact with the data in HDFS. The syntax of this command set is similar to other shells (e.g. bash, csh) that users are already familiar with. Here are some sample action/command pairs:

| Action | Command |
| --- | --- |
| Create a directory named /foodir | bin/hadoop dfs -mkdir /foodir |
| Create a directory named /foodir | bin/hadoop dfs -mkdir /foodir |
| View the contents of a file named /foodir/myfile.txt | bin/hadoop dfs -cat /foodir/myfile.txt |

FS shell is targeted for applications that need a scripting language to interact with the stored data.

### DFSAdmin

The DFSAdmin command set is used for administering an HDFS cluster. These are commands that are used only by an HDFS administrator. Here are some sample action/command pairs:

| Action | Command |
| --- | --- |
| Put the cluster in Safemode | bin/hadoop dfsadmin -safemode enter |
| Generate a list of DataNodes | bin/hadoop dfsadmin -report |
| Decommission DataNode datanodename | bin/hadoop dfsadmin -decommission datanodename |

### Browser Interface

A typical HDFS install configures a web server to expose the HDFS namespace through a configurable TCP port. This allows a user to navigate the HDFS namespace and view the contents of its files using a web browser.

## Space Reclamation

### File Deletes and Undeletes

When a file is deleted by a user or an application, it is not immediately removed from HDFS. Instead, HDFS first renames it to a file in the /trash directory. The file can be restored quickly as long as it remains in /trash. A file remains in /trash for a configurable amount of time. After the expiry of its life in /trash, the NameNode deletes the file from the HDFS namespace. The deletion of a file causes the blocks associated with the file to be freed. Note that there could be an appreciable time delay between the time a file is deleted by a user and the time of the corresponding increase in free space in HDFS.

A user can Undelete a file after deleting it as long as it remains in the /trash directory. If a user wants to undelete a file that he/she has deleted, he/she can navigate the /trash directory and retrieve the file. The /trash directory contains only the latest copy of the file that was deleted. The /trash directory is just like any other directory with one special feature: HDFS applies specified policies to automatically delete files from this directory. The current default policy is to delete files from /trash that are more than 6 hours old. In the future, this policy will be configurable through a well defined interface.

### Decrease Replication Factor

When the replication factor of a file is reduced, the NameNode selects excess replicas that can be deleted. The next Heartbeat transfers this information to the DataNode. The DataNode then removes the corresponding blocks and the corresponding free space appears in the cluster. Once again, there might be a time delay between the completion of the setReplication API call and the appearance of free space in the cluster.

## References

HDFS Java API:
http://hadoop.apache.org/core/docs/current/api/

HDFS source code:
http://hadoop.apache.org/core/version\_control.html

by Dhruba Borthakur

Copyright ©
2007 The Apache Software Foundation.
