# Supplementary material for: Provenance based data integrity checking and verification in cloud environments
Source: PLoS One. 2017 May 17;12(5):e0177576. doi: 10.1371/journal.pone.0177576 (PMC5435237; doi:10.1371/journal.pone.0177576)
Supplement: S1 Dataset — Files in the Dataset are utilized for performance measurement. (ZIP) [file pone.0177576.s001.zip › dataset/html/hdfs_quota_admin_guide - Copy (2) - Copy.html]

Name Space Quotas Administrator Guide


Apache > Hadoop > Core

- Project
- Wiki
- Hadoop 0.18 Documentation

Documentation

Overview

Quickstart

Cluster Setup

HDFS Architecture

HDFS User Guide

HDFS Permissions Guide

HDFS Quotas Administrator Guide

Commands Manual

FS Shell Guide

DistCp Guide

Map-Reduce Tutorial

Native Hadoop Libraries

Streaming

Hadoop Archives

Hadoop On Demand

API Docs

Wiki

FAQ

Mailing Lists

Release Notes

All Changes


PDF

# Name Space Quotas Administrator Guide

The Hadoop Distributed File System (HDFS) allows the administrator to set quotas on individual directories.
Newly created directories have no associated quota.
The largest quota is Long.Max\_Value. A quota of one forces a directory
to remain empty.

The directory quota is a hard limit on the number of names in the tree
rooted at that directory. File and directory creations fault if the quota
would be exceeded. Quotas stick to renamed directories; the rename
operation faults if operation would result in a quota violation.
The attempt to set a quota faults if the directory would be in violation
of the new quota.

Quotas are persistent with the fsimage. When starting, if the fsimage
is immediately in violation of a quota (perhaps the fsimage was
surreptitiously modified), the startup operation fails with an error report.
Setting or removing a quota creates a journal entry.

The following new commands or new options are added to support quotas.
The first two are administration commands.

- dfsadmin -setquota <N> <directory>...<directory>
    
  Set the quota to be N for each directory. Best effort for each directory,
  with faults reported if N is not a positive long integer,
  the directory does not exist or it is a file, or the directory would
  immediately exceed the new quota.
- dfsadmin -clrquota <directory>...<director>
    
  Remove any quota for each directory. Best effort for each directory,
  with faults reported if the directory does not exist or it is a file.
  It is not a fault if the directory has no quota.
- fs -count -q <directory>...<directory>
    
  With the -q option, also report the quota value set for each
  directory, and the available quota remaining. If the directory does not have
  a quota set, the reported values are none and inf.

Copyright ©
2007 The Apache Software Foundation.
