# Supplementary material for: Provenance based data integrity checking and verification in cloud environments
Source: PLoS One. 2017 May 17;12(5):e0177576. doi: 10.1371/journal.pone.0177576 (PMC5435237; doi:10.1371/journal.pone.0177576)
Supplement: S1 Dataset — Files in the Dataset are utilized for performance measurement. (ZIP) [file pone.0177576.s001.zip › dataset/html/hdfs_shell - Copy (2) - Copy.html]

Hadoop Shell Commands


Apache > Hadoop > Core

- Project
- Wiki
- Hadoop 0.18 Documentation

Documentation

Overview

Quickstart

Cluster Setup

HDFS Architecture

HDFS User Guide

HDFS Permissions Guide

HDFS Quotas Administrator Guide

Commands Manual

FS Shell Guide

DistCp Guide

Map-Reduce Tutorial

Native Hadoop Libraries

Streaming

Hadoop Archives

Hadoop On Demand

API Docs

Wiki

FAQ

Mailing Lists

Release Notes

All Changes


PDF

# Hadoop Shell Commands

- FS Shell 
  - cat
  - chgrp
  - chmod
  - chown
  - copyFromLocal
  - copyToLocal
  - cp
  - du
  - dus
  - expunge
  - get
  - getmerge
  - ls
  - lsr
  - mkdir
  - movefromLocal
  - mv
  - put
  - rm
  - rmr
  - setrep
  - stat
  - tail
  - test
  - text
  - touchz

## FS Shell

The FileSystem (FS) shell is invoked by
bin/hadoop fs <args>.
All the FS shell commands take path URIs as arguments. The URI
format is *scheme://autority/path*. For HDFS the scheme
is *hdfs*, and for the local filesystem the scheme
is *file*. The scheme and authority are optional. If not
specified, the default scheme specified in the configuration is
used. An HDFS file or directory such as */parent/child*
can be specified as *hdfs://namenodehost/parent/child* or
simply as */parent/child* (given that your configuration
is set to point to *hdfs://namenodehost*). Most of the
commands in FS shell behave like corresponding Unix
commands. Differences are described with each of the
commands. Error information is sent to *stderr* and the
output is sent to *stdout*.

### cat

Usage: hadoop fs -cat URI [URI …]

Copies source paths to *stdout*.

Example:

- hadoop fs -cat hdfs://nn1.example.com/file1 hdfs://nn2.example.com/file2
- hadoop fs -cat file:///file3 /user/hadoop/file4

Exit Code:  
 Returns 0 on success and -1 on error.

### chgrp

Usage: hadoop fs -chgrp [-R] GROUP URI [URI …]

Change group association of files. With -R, make the change recursively through the directory structure. The user must be the owner of files, or else a super-user. Additional information is in the Permissions User Guide.

### chmod

Usage: hadoop fs -chmod [-R] <MODE[,MODE]... | OCTALMODE> URI [URI …]

Change the permissions of files. With -R, make the change recursively through the directory structure. The user must be the owner of the file, or else a super-user. Additional information is in the Permissions User Guide.

### chown

Usage: hadoop fs -chown [-R] [OWNER][:[GROUP]] URI [URI ]

Change the owner of files. With -R, make the change recursively through the directory structure. The user must be a super-user. Additional information is in the Permissions User Guide.

### copyFromLocal

Usage: hadoop fs -copyFromLocal <localsrc> URI

Similar to **put** command, except that the source is restricted to a local file reference.

### copyToLocal

Usage: hadoop fs -copyToLocal [-ignorecrc] [-crc] URI <localdst>

Similar to **get** command, except that the destination is restricted to a local file reference.

### cp

Usage: hadoop fs -cp URI [URI …] <dest>

Copy files from source to destination. This command allows multiple sources as well in which case the destination must be a directory.
  
Example:

- hadoop fs -cp /user/hadoop/file1 /user/hadoop/file2
- hadoop fs -cp /user/hadoop/file1 /user/hadoop/file2 /user/hadoop/dir

Exit Code:

Returns 0 on success and -1 on error.

### du

Usage: hadoop fs -du URI [URI …]

Displays aggregate length of files contained in the directory or the length of a file in case its just a file.  
Example:  
hadoop fs -du /user/hadoop/dir1 /user/hadoop/file1 hdfs://nn.example.com/user/hadoop/dir1
  
Exit Code:  
 Returns 0 on success and -1 on error.

### dus

Usage: hadoop fs -dus <args>

Displays a summary of file lengths.

### expunge

Usage: hadoop fs -expunge

Empty the Trash. Refer to HDFS Design for more information on Trash feature.

### get

Usage: hadoop fs -get [-ignorecrc] [-crc] <src> <localdst>

Copy files to the local file system. Files that fail the CRC check may be copied with the
-ignorecrc option. Files and CRCs may be copied using the
-crc option.

Example:

- hadoop fs -get /user/hadoop/file localfile
- hadoop fs -get hdfs://nn.example.com/user/hadoop/file localfile

Exit Code:

Returns 0 on success and -1 on error.

### getmerge

Usage: hadoop fs -getmerge <src> <localdst> [addnl]

Takes a source directory and a destination file as input and concatenates files in src into the destination local file. Optionally addnl can be set to enable adding a newline character at the end of each file.

### ls

Usage: hadoop fs -ls <args>

For a file returns stat on the file with the following format:  
filename <number of replicas> filesize modification\_date modification\_time permissions userid groupid
  
For a directory it returns list of its direct children as in unix.
A directory is listed as:   
dirname <dir> modification\_time modification\_time permissions userid groupid
  
Example:  
hadoop fs -ls /user/hadoop/file1 /user/hadoop/file2 hdfs://nn.example.com/user/hadoop/dir1 /nonexistentfile
  
Exit Code:  
 Returns 0 on success and -1 on error.

### lsr

Usage: hadoop fs -lsr <args>
  
Recursive version of ls. Similar to Unix ls -R.

### mkdir

Usage: hadoop fs -mkdir <paths>

Takes path uri's as argument and creates directories. The behavior is much like unix mkdir -p creating parent directories along the path.

Example:

- hadoop fs -mkdir /user/hadoop/dir1 /user/hadoop/dir2
- hadoop fs -mkdir hdfs://nn1.example.com/user/hadoop/dir hdfs://nn2.example.com/user/hadoop/dir

Exit Code:

Returns 0 on success and -1 on error.

### movefromLocal

Usage: dfs -moveFromLocal <src> <dst>

Displays a "not implemented" message.

### mv

Usage: hadoop fs -mv URI [URI …] <dest>

Moves files from source to destination. This command allows multiple sources as well in which case the destination needs to be a directory. Moving files across filesystems is not permitted.
  
Example:

- hadoop fs -mv /user/hadoop/file1 /user/hadoop/file2
- hadoop fs -mv hdfs://nn.example.com/file1 hdfs://nn.example.com/file2 hdfs://nn.example.com/file3 hdfs://nn.example.com/dir1

Exit Code:

Returns 0 on success and -1 on error.

### put

Usage: hadoop fs -put <localsrc> ... <dst>

Copy single src, or multiple srcs from local file system to the destination filesystem. Also reads input from stdin and writes to destination filesystem.

- hadoop fs -put localfile /user/hadoop/hadoopfile
- hadoop fs -put localfile1 localfile2 /user/hadoop/hadoopdir
- hadoop fs -put localfile hdfs://nn.example.com/hadoop/hadoopfile
- hadoop fs -put - hdfs://nn.example.com/hadoop/hadoopfile
    
  Reads the input from stdin.

Exit Code:

Returns 0 on success and -1 on error.

### rm

Usage: hadoop fs -rm URI [URI …]

Delete files specified as args. Only deletes non empty directory and files. Refer to rmr for recursive deletes.  
Example:

- hadoop fs -rm hdfs://nn.example.com/file /user/hadoop/emptydir

Exit Code:

Returns 0 on success and -1 on error.

### rmr

Usage: hadoop fs -rmr URI [URI …]

Recursive version of delete.  
Example:

- hadoop fs -rmr /user/hadoop/dir
- hadoop fs -rmr hdfs://nn.example.com/user/hadoop/dir

Exit Code:

Returns 0 on success and -1 on error.

### setrep

Usage: hadoop fs -setrep [-R] <path>

Changes the replication factor of a file. -R option is for recursively increasing the replication factor of files within a directory.

Example:

- hadoop fs -setrep -w 3 -R /user/hadoop/dir1

Exit Code:

Returns 0 on success and -1 on error.

### stat

Usage: hadoop fs -stat URI [URI …]

Returns the stat information on the path.

Example:

- hadoop fs -stat path

Exit Code:  
 Returns 0 on success and -1 on error.

### tail

Usage: hadoop fs -tail [-f] URI

Displays last kilobyte of the file to stdout. -f option can be used as in Unix.

Example:

- hadoop fs -tail pathname

Exit Code:   
 Returns 0 on success and -1 on error.

### test

Usage: hadoop fs -test -[ezd] URI

Options:   
-e check to see if the file exists. Return 0 if true.   
-z check to see if the file is zero length. Return 0 if true   
-d check return 1 if the path is directory else return 0.

Example:

- hadoop fs -test -e filename

### text

Usage: hadoop fs -text <src>

Takes a source file and outputs the file in text format. The allowed formats are zip and TextRecordInputStream.

### touchz

Usage: hadoop fs -touchz URI [URI …]

Create a file of zero length.

Example:

- hadoop -touchz pathname

Exit Code:  
 Returns 0 on success and -1 on error.

Copyright ©
2007 The Apache Software Foundation.
