# Supplementary material for: Provenance based data integrity checking and verification in cloud environments
Source: PLoS One. 2017 May 17;12(5):e0177576. doi: 10.1371/journal.pone.0177576 (PMC5435237; doi:10.1371/journal.pone.0177576)
Supplement: S1 Dataset — Files in the Dataset are utilized for performance measurement. (ZIP) [file pone.0177576.s001.zip › dataset/html/hdfs_user_guide - Copy (2) - Copy.html]

HDFS User Guide


Apache > Hadoop > Core

- Project
- Wiki
- Hadoop 0.18 Documentation

Documentation

Overview

Quickstart

Cluster Setup

HDFS Architecture

HDFS User Guide

HDFS Permissions Guide

HDFS Quotas Administrator Guide

Commands Manual

FS Shell Guide

DistCp Guide

Map-Reduce Tutorial

Native Hadoop Libraries

Streaming

Hadoop Archives

Hadoop On Demand

API Docs

Wiki

FAQ

Mailing Lists

Release Notes

All Changes


PDF

# HDFS User Guide

- Purpose
- Overview
- Pre-requisites
- Web Interface
- Shell Commands
  - DFSAdmin Command
- Secondary NameNode
- Rebalancer
- Rack Awareness
- Safemode
- fsck
- Upgrade and Rollback
- File Permissions and Security
- Scalability
- Related Documentation

## Purpose

This document is a starting point for users working with
Hadoop Distributed File System (HDFS) either as a part of a
Hadoop
cluster or as a stand-alone general purpose distributed file system.
While HDFS is designed to "just work" in many environments, a working
knowledge of HDFS helps greatly with configuration improvements and
diagnostics on a specific cluster.

## Overview

HDFS is the primary distributed storage used by Hadoop applications. A
HDFS cluster primarily consists of a NameNode that manages the
file system metadata and DataNodes that store the actual data. The
architecture of HDFS is described in detail
here. This user guide primarily deals with
interaction of users and administrators with HDFS clusters.
The diagram from
HDFS architecture depicts
basic interactions among NameNode, the DataNodes, and the clients.
Clients contact NameNode for file metadata or file modifications and perform
actual file I/O directly with the DataNodes.

The following are some of the salient features that could be of
interest to many users.

- Hadoop, including HDFS, is well suited for distributed storage
  and distributed processing using commodity hardware. It is fault
  tolerant, scalable, and extremely simple to expand.
  Map-Reduce,
  well known for its simplicity and applicability for large set of
  distributed applications, is an integral part of Hadoop.
- HDFS is highly configurable with a default configuration well
  suited for many installations. Most of the time, configuration
  needs to be tuned only for very large clusters.
- Hadoop is written in Java and is supported on all major platforms.
- Hadoop supports shell-like commands to interact with HDFS directly.
- The NameNode and Datanodes have built in web servers that makes it
  easy to check current status of the cluster.
- New features and improvements are regularly implemented in HDFS.
  The following is a subset of useful features in HDFS:
  - File permissions and authentication.
  - *Rack awareness*: to take a node's physical location into
    account while scheduling tasks and allocating storage.
  - Safemode: an administrative mode for maintenance.
  - fsck: a utility to diagnose health of the file system, to
    find missing files or blocks.
  - Rebalancer: tool to balance the cluster when the data is
    unevenly distributed among DataNodes.
  - Upgrade and rollback: after a software upgrade,
    it is possible to
    rollback to HDFS' state before the upgrade in case of unexpected
    problems.
  - Secondary NameNode: performs periodic checkpoints of the
    namespace and helps keep the size of file containing log of HDFS
    modifications within certain limits at the NameNode.

## Pre-requisites

The following documents describe installation and set up of a
Hadoop cluster :

- Hadoop Quick Start
  for first-time users.
- Hadoop Cluster Setup
  for large, distributed clusters.

The rest of this document assumes the user is able to set up and run a
HDFS with at least one DataNode. For the purpose of this document,
both the NameNode and DataNode could be running on the same physical
machine.

## Web Interface

NameNode and DataNode each run an internal web server in order to
display basic information about the current status of the cluster.
With the default configuration, the NameNode front page is at
http://namenode-name:50070/.
It lists the DataNodes in the cluster and basic statistics of the
cluster. The web interface can also be used to browse the file
system (using "Browse the file system" link on the NameNode front
page).

## Shell Commands

Hadoop includes various shell-like commands that directly
interact with HDFS and other file systems that Hadoop supports.
The command
bin/hadoop fs -help
lists the commands supported by Hadoop
shell. Furthermore, the command
bin/hadoop fs -help command-name
displays more detailed help for a command. These commands support
most of the normal files ystem operations like copying files,
changing file permissions, etc. It also supports a few HDFS
specific operations like changing replication of files.

### DFSAdmin Command

The bin/hadoop dfsadmin
command supports a few HDFS administration related operations.
The bin/hadoop dfsadmin -help command
lists all the commands currently supported. For e.g.:

- -report
  : reports basic statistics of HDFS. Some of this information is
  also available on the NameNode front page.
- -safemode
  : though usually not required, an administrator can manually enter
  or leave Safemode.
- -finalizeUpgrade
  : removes previous backup of the cluster made during last upgrade.

For command usage, see dfsadmin command.

## Secondary NameNode

The NameNode stores modifications to the file system as a log
appended to a native file system file (edits).
When a NameNode starts up, it reads HDFS state from an image
file (fsimage) and then applies edits from the
edits log file. It then writes new HDFS state to the fsimage
and starts normal
operation with an empty edits file. Since NameNode merges
fsimage and edits files only during start up,
the edits log file could get very large over time on a busy cluster.
Another side effect of a larger edits file is that next
restart of NameNode takes longer.

The secondary NameNode merges the fsimage and the edits log files periodically
and keeps edits log size within a limit. It is usually run on a
different machine than the primary NameNode since its memory requirements
are on the same order as the primary NameNode. The secondary
NameNode is started by bin/start-dfs.sh on the nodes
specified in conf/masters file.

The start of the checkpoint process on the secondary NameNode is
controlled by two configuration parameters.

- fs.checkpoint.period, set to 1 hour by default, specifies
  the maximum delay between two consecutive checkpoints, and
- fs.checkpoint.size, set to 64MB by default, defines the
  size of the edits log file that forces an urgent checkpoint even if
  the maximum checkpoint delay is not reached.

The secondary NameNode stores the latest checkpoint in a
directory which is structured the same way as the primary NameNode's
directory. So that the check pointed image is always ready to be
read by the primary NameNode if necessary.

The latest checkpoint can be imported to the primary NameNode if
all other copies of the image and the edits files are lost.
In order to do that one should:

- Create an empty directory specified in the
  dfs.name.dir configuration variable;
- Specify the location of the checkpoint directory in the
  configuration variable fs.checkpoint.dir;
- and start the NameNode with -importCheckpoint option.

The NameNode will upload the checkpoint from the
fs.checkpoint.dir directory and then save it to the NameNode
directory(s) set in dfs.name.dir.
The NameNode will fail if a legal image is contained in
dfs.name.dir.
The NameNode verifies that the image in fs.checkpoint.dir is
consistent, but does not modify it in any way.

For command usage, see secondarynamenode command.

## Rebalancer

HDFS data might not always be be placed uniformly across the
DataNode. One common reason is addition of new DataNodes to an
existing cluster. While placing new blocks (data for a file is
stored as a series of blocks), NameNode considers various
parameters before choosing the DataNodes to receive these blocks.
Some of the considerations are:

- Policy to keep one of the replicas of a block on the same node
  as the node that is writing the block.
- Need to spread different replicas of a block across the racks so
  that cluster can survive loss of whole rack.
- One of the replicas is usually placed on the same rack as the
  node writing to the file so that cross-rack network I/O is
  reduced.
- Spread HDFS data uniformly across the DataNodes in the cluster.

Due to multiple competing considerations, data might not be
uniformly placed across the DataNodes.
HDFS provides a tool for administrators that analyzes block
placement and rebalanaces data across the DataNode. A brief
administrator's guide for rebalancer as a
PDF
is attached to
HADOOP-1652.

For command usage, see balancer command.

## Rack Awareness

Typically large Hadoop clusters are arranged in racks and
network traffic between different nodes with in the same rack is
much more desirable than network traffic across the racks. In
addition NameNode tries to place replicas of block on
multiple racks for improved fault tolerance. Hadoop lets the
cluster administrators decide which rack a node belongs to
through configuration variable dfs.network.script. When this
script is configured, each node runs the script to determine its
rack id. A default installation assumes all the nodes belong to
the same rack. This feature and configuration is further described
in PDF
attached to
HADOOP-692.

## Safemode

During start up the NameNode loads the file system state from the
fsimage and the edits log file. It then waits for DataNodes
to report their blocks so that it does not prematurely start
replicating the blocks though enough replicas already exist in the
cluster. During this time NameNode stays in Safemode.
Safemode
for the NameNode is essentially a read-only mode for the HDFS cluster,
where it does not allow any modifications to file system or blocks.
Normally the NameNode leaves Safemode automatically after the DataNodes
have reported that most file system blocks are available.
If required, HDFS could be placed in Safemode explicitly
using 'bin/hadoop dfsadmin -safemode' command. NameNode front
page shows whether Safemode is on or off. A more detailed
description and configuration is maintained as JavaDoc for
setSafeMode().

## fsck

HDFS supports the fsck command to check for various
inconsistencies.
It it is designed for reporting problems with various
files, for example, missing blocks for a file or under-replicated
blocks. Unlike a traditional fsck utility for native file systems,
this command does not correct the errors it detects. Normally NameNode
automatically corrects most of the recoverable failures. By default
fsck ignores open files but provides an option to select all files during reporting.
The HDFS fsck command is not a
Hadoop shell command. It can be run as 'bin/hadoop fsck'.
For command usage, see fsck command.
fsck can be run on the whole file system or on a subset of files.

## Upgrade and Rollback

When Hadoop is upgraded on an existing cluster, as with any
software upgrade, it is possible there are new bugs or
incompatible changes that affect existing applications and were
not discovered earlier. In any non-trivial HDFS installation, it
is not an option to loose any data, let alone to restart HDFS from
scratch. HDFS allows administrators to go back to earlier version
of Hadoop and rollback the cluster to the state it was in
before
the upgrade. HDFS upgrade is described in more detail in
upgrade wiki.
HDFS can have one such backup at a time. Before upgrading,
administrators need to remove existing backup using bin/hadoop
dfsadmin -finalizeUpgrade command. The following
briefly describes the typical upgrade procedure:

- Before upgrading Hadoop software,
  *finalize* if there an existing backup.
  dfsadmin -upgradeProgress status
  can tell if the cluster needs to be *finalized*.
- Stop the cluster and distribute new version of Hadoop.
- Run the new version with -upgrade option
  (bin/start-dfs.sh -upgrade).
- Most of the time, cluster works just fine. Once the new HDFS is
  considered working well (may be after a few days of operation),
  finalize the upgrade. Note that until the cluster is finalized,
  deleting the files that existed before the upgrade does not free
  up real disk space on the DataNodes.
- If there is a need to move back to the old version,
  - stop the cluster and distribute earlier version of Hadoop.
  - start the cluster with rollback option.
    (bin/start-dfs.h -rollback).

## File Permissions and Security

The file permissions are designed to be similar to file permissions on
other familiar platforms like Linux. Currently, security is limited
to simple file permissions. The user that starts NameNode is
treated as the superuser for HDFS. Future versions of HDFS will
support network authentication protocols like Kerberos for user
authentication and encryption of data transfers. The details are discussed in the
Permissions User and Administrator Guide.

## Scalability

Hadoop currently runs on clusters with thousands of nodes.
Powered By Hadoop
lists some of the organizations that deploy Hadoop on large
clusters. HDFS has one NameNode for each cluster. Currently
the total memory available on NameNode is the primary scalability
limitation. On very large clusters, increasing average size of
files stored in HDFS helps with increasing cluster size without
increasing memory requirements on NameNode.
The default configuration may not suite very large clustes.
Hadoop FAQ page lists
suggested configuration improvements for large Hadoop clusters.

## Related Documentation

This user guide is a good starting point for
working with HDFS. While the user guide continues to improve,
there is a large wealth of documentation about Hadoop and HDFS.
The following list is a starting point for further exploration:

- Hadoop Home Page: The start page for everything Hadoop.
- Hadoop Wiki
  : Front page for Hadoop Wiki documentation. Unlike this
  guide which is part of Hadoop source tree, Hadoop Wiki is
  regularly edited by Hadoop Community.
- FAQ from Hadoop Wiki.
- Hadoop 
  JavaDoc API.
- Hadoop User Mailing List :
  core-user[at]hadoop.apache.org.
- Explore conf/hadoop-default.xml.
  It includes brief
  description of most of the configuration variables available.
- Commands Manual
  : commands usage.

Copyright ©
2007 The Apache Software Foundation.
