# Supplementary material for: Provenance based data integrity checking and verification in cloud environments
Source: PLoS One. 2017 May 17;12(5):e0177576. doi: 10.1371/journal.pone.0177576 (PMC5435237; doi:10.1371/journal.pone.0177576)
Supplement: S1 Dataset — Files in the Dataset are utilized for performance measurement. (ZIP) [file pone.0177576.s001.zip › dataset/html/hod_admin_guide - Copy (2) - Copy.html]

Hadoop On Demand


Apache > Hadoop > Core

- Project
- Wiki
- Hadoop 0.18 Documentation

Documentation

Overview

Quickstart

Cluster Setup

HDFS Architecture

HDFS User Guide

HDFS Permissions Guide

HDFS Quotas Administrator Guide

Commands Manual

FS Shell Guide

DistCp Guide

Map-Reduce Tutorial

Native Hadoop Libraries

Streaming

Hadoop Archives

Hadoop On Demand

API Docs

Wiki

FAQ

Mailing Lists

Release Notes

All Changes


PDF

# Hadoop On Demand

- Overview
- Pre-requisites
- Resource Manager
- Installing HOD
- Configuring HOD
  - Minimal Configuration
  - Advanced Configuration
- Running HOD
- Supporting Tools and Utilities
  - logcondense.py - Manage Log Files
    - Running logcondense.py
    - Command Line Options for logcondense.py
  - checklimits.sh - Monitor Resource Limits
    - Running checklimits.sh

## Overview

The Hadoop On Demand (HOD) project is a system for provisioning and
managing independent Hadoop Map/Reduce and Hadoop Distributed File System (HDFS)
instances on a shared cluster
of nodes. HOD is a tool that makes it easy for administrators and users to
quickly setup and use Hadoop. It is also a very useful tool for Hadoop developers
and testers who need to share a physical cluster for testing their own Hadoop
versions.

HOD relies on a resource manager (RM) for allocation of nodes that it can use for
running Hadoop instances. At present it runs with the Torque
resource manager.

The basic system architecture of HOD includes these components:

- A Resource manager (possibly together with a scheduler)
- Various HOD components
- Hadoop Map/Reduce and HDFS daemons

HOD provisions and maintains Hadoop Map/Reduce and, optionally, HDFS instances
through interaction with the above components on a given cluster of nodes. A cluster of
nodes can be thought of as comprising two sets of nodes:

- Submit nodes: Users use the HOD client on these nodes to allocate clusters, and then
  use the Hadoop client to submit Hadoop jobs.
- Compute nodes: Using the resource manager, HOD components are run on these nodes to
  provision the Hadoop daemons. After that Hadoop jobs run on them.

Here is a brief description of the sequence of operations in allocating a cluster and
running jobs on them.

- The user uses the HOD client on the Submit node to allocate a desired number of
  cluster nodes and to provision Hadoop on them.
- The HOD client uses a resource manager interface (qsub, in Torque) to submit a HOD
  process, called the RingMaster, as a Resource Manager job, to request the user's desired number
  of nodes. This job is submitted to the central server of the resource manager (pbs\_server, in Torque).
- On the compute nodes, the resource manager slave daemons (pbs\_moms in Torque) accept
  and run jobs that they are assigned by the central server (pbs\_server in Torque). The RingMaster
  process is started on one of the compute nodes (mother superior, in Torque).
- The RingMaster then uses another resource manager interface (pbsdsh, in Torque) to run
  the second HOD component, HodRing, as distributed tasks on each of the compute
  nodes allocated.
- The HodRings, after initializing, communicate with the RingMaster to get Hadoop commands,
  and run them accordingly. Once the Hadoop commands are started, they register with the RingMaster,
  giving information about the daemons.
- All the configuration files needed for Hadoop instances are generated by HOD itself,
  some obtained from options given by user in its own configuration file.
- The HOD client keeps communicating with the RingMaster to find out the location of the
  JobTracker and HDFS daemons.

The rest of this document describes how to setup HOD on a physical cluster of nodes.

## Pre-requisites

To use HOD, your system should include the following hardware and software
components.

Operating System: HOD is currently tested on RHEL4.  
Nodes : HOD requires a minimum of three nodes configured through a resource manager.

Software

The following components must be installed on ALL nodes before using HOD:

- Torque: Resource manager
- Python : HOD requires version 2.5.1 of Python.

The following components are optional and can be installed to obtain better
functionality from HOD:

- Twisted Python: This can be
  used for improving the scalability of HOD. If this module is detected to be
  installed, HOD uses it, else it falls back to default modules.
- Hadoop: HOD can automatically
  distribute Hadoop to all nodes in the cluster. However, it can also use a
  pre-installed version of Hadoop, if it is available on all nodes in the cluster.
  HOD currently supports Hadoop 0.15 and above.

NOTE: HOD configuration requires the location of installs of these
components to be the same on all nodes in the cluster. It will also
make the configuration simpler to have the same location on the submit
nodes.

## Resource Manager

Currently HOD works with the Torque resource manager, which it uses for its node
allocation and job submission. Torque is an open source resource manager from
Cluster Resources, a community effort
based on the PBS project. It provides control over batch jobs and distributed compute nodes. Torque is
freely available for download from here.

All documentation related to torque can be seen under
the section TORQUE Resource Manager here. You can
get wiki documentation from here.
Users may wish to subscribe to TORQUE’s mailing list or view the archive for questions,
comments here.

To use HOD with Torque:

- Install Torque components: pbs\_server on one node (head node), pbs\_mom on all
  compute nodes, and PBS client tools on all compute nodes and submit
  nodes. Perform at least a basic configuration so that the Torque system is up and
  running, that is, pbs\_server knows which machines to talk to. Look here
  for basic configuration.
  For advanced configuration, see here
- Create a queue for submitting jobs on the pbs\_server. The name of the queue is the
  same as the HOD configuration parameter, resource-manager.queue. The HOD client uses this queue to
  submit the RingMaster process as a Torque job.
- Specify a cluster name as a property for all nodes in the cluster.
  This can be done by using the qmgr command. For example:
  qmgr -c "set node node properties=cluster-name". The name of the cluster is the same as
  the HOD configuration parameter, hod.cluster.
- Make sure that jobs can be submitted to the nodes. This can be done by
  using the qsub command. For example:
  echo "sleep 30" | qsub -l nodes=3

## Installing HOD

Once the resource manager is set up, you can obtain and
install HOD.

- If you are getting HOD from the Hadoop tarball, it is available under the
  'contrib' section of Hadoop, under the root directory 'hod'.
- If you are building from source, you can run ant tar from the Hadoop root
  directory to generate the Hadoop tarball, and then get HOD from there,
  as described above.
- Distribute the files under this directory to all the nodes in the
  cluster. Note that the location where the files are copied should be
  the same on all the nodes.
- Note that compiling hadoop would build HOD with appropriate permissions
  set on all the required script files in HOD.

## Configuring HOD

You can configure HOD once it is installed. The minimal configuration needed
to run HOD is described below. More advanced configuration options are discussed
in the HOD Configuration Guide.

### Minimal Configuration

To get started using HOD, the following minimal configuration is
required:

- On the node from where you want to run HOD, edit the file hodrc
  located in the <install dir>/conf directory. This file
  contains the minimal set of values required to run hod.
- Specify values suitable to your environment for the following
  variables defined in the configuration file. Note that some of these
  variables are defined at more than one place in the file.

  - ${JAVA\_HOME}: Location of Java for Hadoop. Hadoop supports Sun JDK
    1.5.x and above.
  - ${CLUSTER\_NAME}: Name of the cluster which is specified in the
    'node property' as mentioned in resource manager configuration.
  - ${HADOOP\_HOME}: Location of Hadoop installation on the compute and
    submit nodes.
  - ${RM\_QUEUE}: Queue configured for submitting jobs in the resource
    manager configuration.
  - ${RM\_HOME}: Location of the resource manager installation on the
    compute and submit nodes.
- The following environment variables may need to be set depending on
  your environment. These variables must be defined where you run the
  HOD client and must also be specified in the HOD configuration file as the
  value of the key resource\_manager.env-vars. Multiple variables can be
  specified as a comma separated list of key=value pairs.

  - HOD\_PYTHON\_HOME: If you install python to a non-default location
    of the compute nodes, or submit nodes, then this variable must be
    defined to point to the python executable in the non-standard
    location.

### Advanced Configuration

You can review and modify other configuration options to suit
your specific needs. Refer to the Configuration
Guide for more information.

## Running HOD

You can run HOD once it is configured. Refer to the HOD User Guide for more information.

## Supporting Tools and Utilities

This section describes supporting tools and utilities that can be used to
manage HOD deployments.

### logcondense.py - Manage Log Files

As mentioned in the
HOD User Guide,
HOD can be configured to upload
Hadoop logs to a statically configured HDFS. Over time, the number of logs uploaded
to HDFS could increase. logcondense.py is a tool that helps
administrators to remove log files uploaded to HDFS.

#### Running logcondense.py

logcondense.py is available under hod\_install\_location/support folder. You can either
run it using python, for example, *python logcondense.py*, or give execute permissions
to the file, and directly run it as *logcondense.py*. logcondense.py needs to be
run by a user who has sufficient permissions to remove files from locations where log
files are uploaded in the HDFS, if permissions are enabled. For example as mentioned in the
configuration guide, the logs could
be configured to come under the user's home directory in HDFS. In that case, the user
running logcondense.py should have super user privileges to remove the files from under
all user home directories.

#### Command Line Options for logcondense.py

The following command line options are supported for logcondense.py.

|  |  |  |  |
| --- | --- | --- | --- |
| Short Option | Long option | Meaning | Example |
| -p | --package | Complete path to the hadoop script. The version of hadoop must be the same as the one running HDFS. | /usr/bin/hadoop |
| -d | --days | Delete log files older than the specified number of days | 7 |
| -c | --config | Path to the Hadoop configuration directory, under which hadoop-site.xml resides. The hadoop-site.xml must point to the HDFS NameNode from where logs are to be removed. | /home/foo/hadoop/conf |
| -l | --logs | A HDFS path, this must be the same HDFS path as specified for the log-destination-uri, as mentioned in the configuration guide, without the hdfs:// URI string | /user |
| -n | --dynamicdfs | If true, this will indicate that the logcondense.py script should delete HDFS logs in addition to Map/Reduce logs. Otherwise, it only deletes Map/Reduce logs, which is also the default if this option is not specified. This option is useful if dynamic HDFS installations are being provisioned by HOD, and the static HDFS installation is being used only to collect logs - a scenario that may be common in test clusters. | false |

So, for example, to delete all log files older than 7 days using a hadoop-site.xml stored in
~/hadoop-conf, using the hadoop installation under ~/hadoop-0.17.0, you could say:

*python logcondense.py -p ~/hadoop-0.17.0/bin/hadoop -d 7 -c ~/hadoop-conf -l /user*

### checklimits.sh - Monitor Resource Limits

checklimits.sh is a HOD tool specific to the Torque/Maui environment
(Maui Cluster Scheduler is an open source job
scheduler for clusters and supercomputers, from clusterresources). The
checklimits.sh script
updates the torque comment field when newly submitted job(s) violate or
exceed
over user limits set up in Maui scheduler. It uses qstat, does one pass
over the torque job-list to determine queued or unfinished jobs, runs Maui
tool checkjob on each job to see if user limits are violated and then
runs torque's qalter utility to update job attribute 'comment'. Currently
it updates the comment as *User-limits exceeded. Requested:([0-9]\*)
Used:([0-9]\*) MaxLimit:([0-9]\*)* for those jobs that violate limits.
This comment field is then used by HOD to behave accordingly depending on
the type of violation.

#### Running checklimits.sh

checklimits.sh is available under the hod\_install\_location/support
folder. This shell script can be run directly as *sh
checklimits.sh* or as *./checklimits.sh* after enabling
execute permissions. Torque and Maui binaries should be available
on the machine where the tool is run and should be in the path
of the shell script process. To update the
comment field of jobs from different users, this tool must be run with
torque administrative privileges. This tool must be run repeatedly
after specific intervals of time to frequently update jobs violating
constraints, for example via cron. Please note that the resource manager
and scheduler commands used in this script can be expensive and so
it is better not to run this inside a tight loop without sleeping.

Copyright ©
2007 The Apache Software Foundation.
