# Supplementary material for: Provenance based data integrity checking and verification in cloud environments
Source: PLoS One. 2017 May 17;12(5):e0177576. doi: 10.1371/journal.pone.0177576 (PMC5435237; doi:10.1371/journal.pone.0177576)
Supplement: S1 Dataset — Files in the Dataset are utilized for performance measurement. (ZIP) [file pone.0177576.s001.zip › dataset/html/hod_config_guide - Copy (2) - Copy.html]

Hadoop On Demand: Configuration Guide


Apache > Hadoop > Core

- Project
- Wiki
- Hadoop 0.18 Documentation

Documentation

Overview

Quickstart

Cluster Setup

HDFS Architecture

HDFS User Guide

HDFS Permissions Guide

HDFS Quotas Administrator Guide

Commands Manual

FS Shell Guide

DistCp Guide

Map-Reduce Tutorial

Native Hadoop Libraries

Streaming

Hadoop Archives

Hadoop On Demand

API Docs

Wiki

FAQ

Mailing Lists

Release Notes

All Changes


PDF

# Hadoop On Demand: Configuration Guide

- 1. Introduction
- 2. Sections
- 3. HOD Configuration Options
  - 3.1 Common configuration options
  - 3.2 hod options
  - 3.3 resource\_manager options
  - 3.4 ringmaster options
  - 3.5 gridservice-hdfs options
  - 3.6 gridservice-mapred options
  - 3.7 hodring options

## 1. Introduction

This document explains some of the most important and commonly used
Hadoop On Demand (HOD) configuration options. Configuration options
can be specified in two ways: a configuration file
in the INI format, and as command line options to the HOD shell,
specified in the format --section.option[=value]. If the same option is
specified in both places, the value specified on the command line
overrides the value in the configuration file.

To get a simple description of all configuration options, type:

$ hod --verbose-help

## 2. Sections

HOD organizes configuration options into these sections:

- hod: Options for the HOD client
- resource\_manager: Options for specifying which resource manager
  to use, and other parameters for using that resource manager
- ringmaster: Options for the RingMaster process,
- hodring: Options for the HodRing processes
- gridservice-mapred: Options for the Map/Reduce daemons
- gridservice-hdfs: Options for the HDFS daemons.

## 3. HOD Configuration Options

The following section describes configuration options common to most
HOD sections followed by sections that describe configuration options
specific to each HOD section.

### 3.1 Common configuration options

Certain configuration options are defined in most of the sections of
the HOD configuration. Options defined in a section, are used by the
process for which that section applies. These options have the same
meaning, but can have different values in each section.

- temp-dir: Temporary directory for usage by the HOD processes. Make
  sure that the users who will run hod have rights to create
  directories under the directory specified here.
- debug: Numeric value from 1-4. 4 produces the most log information,
  and 1 the least.
- log-dir: Directory where log files are stored. By default, this is
  <install-location>/logs/. The restrictions and notes for the
  temp-dir variable apply here too.
- xrs-port-range: Range of ports, among which an available port shall
  be picked for use to run an XML-RPC server.
- http-port-range: Range of ports, among which an available port shall
  be picked for use to run an HTTP server.
- java-home: Location of Java to be used by Hadoop.
- syslog-address: Address to which a syslog daemon is bound to. The format
  of the value is host:port. If configured, HOD log messages
  will be logged to syslog using this value.

### 3.2 hod options

- cluster: Descriptive name given to the cluster. For Torque, this is
  specified as a 'Node property' for every node in the cluster.
  HOD uses this value to compute the number of available nodes.
- client-params: Comma-separated list of hadoop config parameters
  specified as key-value pairs. These will be used to
  generate a hadoop-site.xml on the submit node that
  should be used for running Map/Reduce jobs.
- job-feasibility-attr: Regular expression string that specifies
  whether and how to check job feasibility - resource
  manager or scheduler limits. The current
  implementation corresponds to the torque job
  attribute 'comment' and by default is disabled.
  When set, HOD uses it to decide what type
  of limit violation is triggered and either
  deallocates the cluster or stays in queued state
  according as the request is beyond maximum limits or
  the cumulative usage has crossed maximum limits.
  The torque comment attribute may be updated
  periodically by an external mechanism. For example,
  comment attribute can be updated by running 
  checklimits.sh script in hod/support directory,
  and then setting job-feasibility-attr equal to the
  value TORQUE\_USER\_LIMITS\_COMMENT\_FIELD,
  "User-limits exceeded. Requested:([0-9]\*)
  Used:([0-9]\*) MaxLimit:([0-9]\*)", will make HOD
  behave accordingly.

### 3.3 resource\_manager options

- queue: Name of the queue configured in the resource manager to which
  jobs are to be submitted.
- batch-home: Install directory to which 'bin' is appended and under
  which the executables of the resource manager can be
  found.
- env-vars: Comma-separated list of key-value pairs,
  expressed as key=value, which would be passed to the jobs
  launched on the compute nodes.
  For example, if the python installation is
  in a non-standard location, one can set the environment
  variable 'HOD\_PYTHON\_HOME' to the path to the python
  executable. The HOD processes launched on the compute nodes
  can then use this variable.

### 3.4 ringmaster options

- work-dirs: Comma-separated list of paths that will serve
  as the root for directories that HOD generates and passes
  to Hadoop for use to store DFS and Map/Reduce data. For e.g.
  this is where DFS data blocks will be stored. Typically,
  as many paths are specified as there are disks available
  to ensure all disks are being utilized. The restrictions
  and notes for the temp-dir variable apply here too.
- max-master-failures: Number of times a hadoop master
  daemon can fail to launch, beyond which HOD will fail
  the cluster allocation altogether. In HOD clusters,
  sometimes there might be a single or few "bad" nodes due
  to issues like missing java, missing or incorrect version
  of Hadoop etc. When this configuration variable is set
  to a positive integer, the RingMaster returns an error
  to the client only when the number of times a hadoop
  master (JobTracker or NameNode) fails to start on these
  bad nodes because of above issues, exceeds the specified
  value. If the number is not exceeded, the next HodRing
  which requests for a command to launch is given the same
  hadoop master again. This way, HOD tries its best for a
  successful allocation even in the presence of a few bad
  nodes in the cluster.

### 3.5 gridservice-hdfs options

- external: If false, indicates that a HDFS cluster must be
  bought up by the HOD system, on the nodes which it
  allocates via the allocate command. Note that in that case,
  when the cluster is de-allocated, it will bring down the
  HDFS cluster, and all the data will be lost.
  If true, it will try and connect to an externally configured
  HDFS system.
  Typically, because input for jobs are placed into HDFS
  before jobs are run, and also the output from jobs in HDFS
  is required to be persistent, an internal HDFS cluster is
  of little value in a production system. However, it allows
  for quick testing.
- host: Hostname of the externally configured NameNode, if any
- fs\_port: Port to which NameNode RPC server is bound.
- info\_port: Port to which the NameNode web UI server is bound.
- pkgs: Installation directory, under which bin/hadoop executable is
  located. This can be used to use a pre-installed version of
  Hadoop on the cluster.
- server-params: Comma-separated list of hadoop config parameters
  specified key-value pairs. These will be used to
  generate a hadoop-site.xml that will be used by the
  NameNode and DataNodes.
- final-server-params: Same as above, except they will be marked final.

### 3.6 gridservice-mapred options

- external: If false, indicates that a Map/Reduce cluster must be
  bought up by the HOD system on the nodes which it allocates
  via the allocate command.
  If true, if will try and connect to an externally
  configured Map/Reduce system.
- host: Hostname of the externally configured JobTracker, if any
- tracker\_port: Port to which the JobTracker RPC server is bound
- info\_port: Port to which the JobTracker web UI server is bound.
- pkgs: Installation directory, under which bin/hadoop executable is
  located
- server-params: Comma-separated list of hadoop config parameters
  specified key-value pairs. These will be used to
  generate a hadoop-site.xml that will be used by the
  JobTracker and TaskTrackers
- final-server-params: Same as above, except they will be marked final.

### 3.7 hodring options

- mapred-system-dir-root: Directory in the DFS under which HOD will
  generate sub-directory names and pass the full path
  as the value of the 'mapred.system.dir' configuration
  parameter to Hadoop daemons. The format of the full
  path will be value-of-this-option/userid/mapredsystem/cluster-id.
  Note that the directory specified here should be such
  that all users can create directories under this, if
  permissions are enabled in HDFS. Setting the value of
  this option to /user will make HOD use the user's
  home directory to generate the mapred.system.dir value.
- log-destination-uri: URL describing a path in an external, static DFS or the
  cluster node's local file system where HOD will upload
  Hadoop logs when a cluster is deallocated. To specify a
  DFS path, use the format 'hdfs://path'. To specify a
  cluster node's local file path, use the format 'file://path'.
  When clusters are deallocated by HOD, the hadoop logs will
  be deleted as part of HOD's cleanup process. To ensure these
  logs persist, you can use this configuration option.
  The format of the path is
  value-of-this-option/userid/hod-logs/cluster-id
  Note that the directory you specify here must be such that all
  users can create sub-directories under this. Setting this value
  to hdfs://user will make the logs come in the user's home directory
  in DFS.
- pkgs: Installation directory, under which bin/hadoop executable is located. This will
  be used by HOD to upload logs if a HDFS URL is specified in log-destination-uri
  option. Note that this is useful if the users are using a tarball whose version
  may differ from the external, static HDFS version.

Copyright ©
2007 The Apache Software Foundation.
