# Supplementary material for: Provenance based data integrity checking and verification in cloud environments
Source: PLoS One. 2017 May 17;12(5):e0177576. doi: 10.1371/journal.pone.0177576 (PMC5435237; doi:10.1371/journal.pone.0177576)
Supplement: S1 Dataset — Files in the Dataset are utilized for performance measurement. (ZIP) [file pone.0177576.s001.zip › dataset/html/hod_user_guide - Copy (2) - Copy.html]

Hadoop On Demand User Guide


Apache > Hadoop > Core

- Project
- Wiki
- Hadoop 0.18 Documentation

Documentation

Overview

Quickstart

Cluster Setup

HDFS Architecture

HDFS User Guide

HDFS Permissions Guide

HDFS Quotas Administrator Guide

Commands Manual

FS Shell Guide

DistCp Guide

Map-Reduce Tutorial

Native Hadoop Libraries

Streaming

Hadoop Archives

Hadoop On Demand

API Docs

Wiki

FAQ

Mailing Lists

Release Notes

All Changes


PDF

# Hadoop On Demand User Guide

- Introduction
- Getting Started Using HOD 
  - A typical HOD session
  - Running hadoop scripts using HOD
- HOD Features 
  - Provisioning and Managing Hadoop Clusters
  - Using a tarball to distribute Hadoop
  - Using an external HDFS
  - Options for Configuring Hadoop
  - Viewing Hadoop Web-UIs
  - Collecting and Viewing Hadoop Logs
  - Auto-deallocation of Idle Clusters
  - Specifying Additional Job Attributes
  - Capturing HOD exit codes in Torque
  - Command Line
  - Options Configuring HOD
- Troubleshooting 
  - hod Hangs During Allocation
  - hod Hangs During Deallocation
  - hod Fails With an error code and error message
  - Hadoop Jobs Not Running on a Successfully Allocated Cluster
  - My Hadoop Job Got Killed
  - Hadoop Job Fails with Message: 'Job tracker still initializing'
  - The Exit Codes For HOD Are Not Getting Into Torque
  - The Hadoop Logs are Not Uploaded to DFS
  - Locating Ringmaster Logs
  - Locating Hodring Logs

## Introduction

Hadoop On Demand (HOD) is a system for provisioning virtual Hadoop clusters over a large physical cluster. It uses the Torque resource manager to do node allocation. On the allocated nodes, it can start Hadoop Map/Reduce and HDFS daemons. It automatically generates the appropriate configuration files (hadoop-site.xml) for the Hadoop daemons and client. HOD also has the capability to distribute Hadoop to the nodes in the virtual cluster that it allocates. In short, HOD makes it easy for administrators and users to quickly setup and use Hadoop. It is also a very useful tool for Hadoop developers and testers who need to share a physical cluster for testing their own Hadoop versions.

HOD supports Hadoop from version 0.15 onwards.

The rest of this document comprises of a quick-start guide that helps you get quickly started with using HOD, a more detailed guide of all HOD features, and a trouble-shooting section.

## Getting Started Using HOD

In this section, we shall see a step-by-step introduction on how to use HOD for the most basic operations. Before following these steps, it is assumed that HOD and its dependent hardware and software components are setup and configured correctly. This is a step that is generally performed by system administrators of the cluster.

The HOD user interface is a command line utility called hod. It is driven by a configuration file, that is typically setup for users by system administrators. Users can override this configuration when using the hod, which is described later in this documentation. The configuration file can be specified in two ways when using hod, as described below:

- Specify it on command line, using the -c option. Such as hod <operation> <required-args> -c path-to-the-configuration-file [other-options]
- Set up an environment variable *HOD\_CONF\_DIR* where hod will be run. This should be pointed to a directory on the local file system, containing a file called *hodrc*. Note that this is analogous to the *HADOOP\_CONF\_DIR* and *hadoop-site.xml* file for Hadoop. If no configuration file is specified on the command line, hod shall look for the *HOD\_CONF\_DIR* environment variable and a *hodrc* file under that.

In examples listed below, we shall not explicitly point to the configuration option, assuming it is correctly specified.

### A typical HOD session

A typical session of HOD will involve at least three steps: allocate, run hadoop jobs, deallocate. In order to do this, perform the following steps.

**Create a Cluster Directory**

The *cluster directory* is a directory on the local file system where hod will generate the Hadoop configuration, *hadoop-site.xml*, corresponding to the cluster it allocates. Pass this directory to the hod operations as stated below. If the cluster directory passed doesn't already exist, HOD will automatically try to create it and use it. Once a cluster is allocated, a user can utilize it to run Hadoop jobs by specifying the cluster directory as the Hadoop --config option.

**Operation *allocate***

The *allocate* operation is used to allocate a set of nodes and install and provision Hadoop on them. It has the following syntax. Note that it requires a cluster\_dir ( -d, --hod.clusterdir) and the number of nodes (-n, --hod.nodecount) needed to be allocated:

$ hod allocate -d cluster\_dir -n number\_of\_nodes [OPTIONS]

If the command completes successfully, then cluster\_dir/hadoop-site.xml will be generated and will contain information about the allocated cluster. It will also print out the information about the Hadoop web UIs.

An example run of this command produces the following output. Note in this example that ~/hod-clusters/test is the cluster directory, and we are allocating 5 nodes:

$ hod allocate -d ~/hod-clusters/test -n 5
  
INFO - HDFS UI on http://foo1.bar.com:53422
  
INFO - Mapred UI on http://foo2.bar.com:55380

**Running Hadoop jobs using the allocated cluster**

Now, one can run Hadoop jobs using the allocated cluster in the usual manner. This assumes variables like *JAVA\_HOME* and path to the Hadoop installation are set up correctly.:

$ hadoop --config cluster\_dir hadoop\_command hadoop\_command\_args

or

$ export HADOOP\_CONF\_DIR=cluster\_dir
  
$ hadoop hadoop\_command hadoop\_command\_args

Continuing our example, the following command will run a wordcount example on the allocated cluster:

$ hadoop --config ~/hod-clusters/test jar /path/to/hadoop/hadoop-examples.jar wordcount /path/to/input /path/to/output

or

$ export HADOOP\_CONF\_DIR=~/hod-clusters/test
  
$ hadoop jar /path/to/hadoop/hadoop-examples.jar wordcount /path/to/input /path/to/output

**Operation *deallocate***

The *deallocate* operation is used to release an allocated cluster. When finished with a cluster, deallocate must be run so that the nodes become free for others to use. The *deallocate* operation has the following syntax. Note that it requires the cluster\_dir (-d, --hod.clusterdir) argument:

$ hod deallocate -d cluster\_dir

Continuing our example, the following command will deallocate the cluster:

$ hod deallocate -d ~/hod-clusters/test

As can be seen, HOD allows the users to allocate a cluster, and use it flexibly for running Hadoop jobs. For example, users can run multiple jobs in parallel on the same cluster, by running hadoop from multiple shells pointing to the same configuration.

### Running hadoop scripts using HOD

The HOD *script operation* combines the operations of allocating, using and deallocating a cluster into a single operation. This is very useful for users who want to run a script of hadoop jobs and let HOD handle the cleanup automatically once the script completes. In order to run hadoop scripts using hod, do the following:

**Create a script file**

This will be a regular shell script that will typically contain hadoop commands, such as:

$ hadoop jar jar\_file options

However, the user can add any valid commands as part of the script. HOD will execute this script setting *HADOOP\_CONF\_DIR* automatically to point to the allocated cluster. So users do not need to worry about this. The users however need to specify a cluster directory just like when using the allocate operation.

**Running the script**

The syntax for the *script operation* as is as follows. Note that it requires a cluster directory ( -d, --hod.clusterdir), number of nodes (-n, --hod.nodecount) and a script file (-s, --hod.script):

$ hod script -d cluster\_directory -n number\_of\_nodes -s script\_file

Note that HOD will deallocate the cluster as soon as the script completes, and this means that the script must not complete until the hadoop jobs themselves are completed. Users must take care of this while writing the script.

## HOD Features

### Provisioning and Managing Hadoop Clusters

The primary feature of HOD is to provision Hadoop Map/Reduce and HDFS clusters. This is described above in the Getting Started section. Also, as long as nodes are available, and organizational policies allow, a user can use HOD to allocate multiple Map/Reduce clusters simultaneously. The user would need to specify different paths for the cluster\_dir parameter mentioned above for each cluster he/she allocates. HOD provides the *list* and the *info* operations to enable managing multiple clusters.

**Operation *list***

The list operation lists all the clusters allocated so far by a user. The cluster directory where the hadoop-site.xml is stored for the cluster, and its status vis-a-vis connectivity with the JobTracker and/or HDFS is shown. The list operation has the following syntax:

$ hod list

**Operation *info***

The info operation shows information about a given cluster. The information shown includes the Torque job id, and locations of the important daemons like the HOD Ringmaster process, and the Hadoop JobTracker and NameNode daemons. The info operation has the following syntax. Note that it requires a cluster directory (-d, --hod.clusterdir):

$ hod info -d cluster\_dir

The cluster\_dir should be a valid cluster directory specified in an earlier *allocate* operation.

### Using a tarball to distribute Hadoop

When provisioning Hadoop, HOD can use either a pre-installed Hadoop on the cluster nodes or distribute and install a Hadoop tarball as part of the provisioning operation. If the tarball option is being used, there is no need to have a pre-installed Hadoop on the cluster nodes, nor a need to use a pre-installed one. This is especially useful in a development / QE environment where individual developers may have different versions of Hadoop to test on a shared cluster.

In order to use a pre-installed Hadoop, you must specify, in the hodrc, the pkgs option in the gridservice-hdfs and gridservice-mapred sections. This must point to the path where Hadoop is installed on all nodes of the cluster.

The syntax for specifying tarball is as follows:

$ hod allocate -d cluster\_dir -n number\_of\_nodes -t hadoop\_tarball\_location

For example, the following command allocates Hadoop provided by the tarball ~/share/hadoop.tar.gz:

$ hod allocate -d ~/hadoop-cluster -n 10 -t ~/share/hadoop.tar.gz

Similarly, when using hod script, the syntax is as follows:

$ hod script -d cluster\_directory -s script\_file -n number\_of\_nodes -t hadoop\_tarball\_location

The hadoop\_tarball specified in the syntax above should point to a path on a shared file system that is accessible from all the compute nodes. Currently, HOD only supports NFS mounted file systems.

*Note:*

- For better distribution performance it is recommended that the Hadoop tarball contain only the libraries and binaries, and not the source or documentation.
- When you want to run jobs against a cluster allocated using the tarball, you must use a compatible version of hadoop to submit your jobs. The best would be to untar and use the version that is present in the tarball itself.
- You need to make sure that there are no Hadoop configuration files, hadoop-env.sh and hadoop-site.xml, present in the conf directory of the tarred distribution. The presence of these files with incorrect values could make the cluster allocation to fail.

### Using an external HDFS

In typical Hadoop clusters provisioned by HOD, HDFS is already set up statically (without using HOD). This allows data to persist in HDFS after the HOD provisioned clusters is deallocated. To use a statically configured HDFS, your hodrc must point to an external HDFS. Specifically, set the following options to the correct values in the section gridservice-hdfs of the hodrc:

external = true

host = Hostname of the HDFS NameNode

fs\_port = Port number of the HDFS NameNode

info\_port = Port number of the HDFS NameNode web UI

*Note:* You can also enable this option from command line. That is, to use a static HDFS, you will need to say:

$ hod allocate -d cluster\_dir -n number\_of\_nodes --gridservice-hdfs.external

HOD can be used to provision an HDFS cluster as well as a Map/Reduce cluster, if required. To do so, set the following option in the section gridservice-hdfs of the hodrc:

external = false

### Options for Configuring Hadoop

HOD provides a very convenient mechanism to configure both the Hadoop daemons that it provisions and also the hadoop-site.xml that it generates on the client side. This is done by specifying Hadoop configuration parameters in either the HOD configuration file, or from the command line when allocating clusters.

**Configuring Hadoop Daemons**

For configuring the Hadoop daemons, you can do the following:

For Map/Reduce, specify the options as a comma separated list of key-value pairs to the server-params option in the gridservice-mapred section. Likewise for a dynamically provisioned HDFS cluster, specify the options in the server-params option in the gridservice-hdfs section. If these parameters should be marked as *final*, then include these in the final-server-params option of the appropriate section.

For example:

server-params = mapred.reduce.parallel.copies=20,io.sort.factor=100,io.sort.mb=128,io.file.buffer.size=131072

final-server-params = mapred.child.java.opts=-Xmx512m,dfs.block.size=134217728,fs.inmemory.size.mb=128

In order to provide the options from command line, you can use the following syntax:

For configuring the Map/Reduce daemons use:

$ hod allocate -d cluster\_dir -n number\_of\_nodes -Mmapred.reduce.parallel.copies=20 -Mio.sort.factor=100

In the example above, the *mapred.reduce.parallel.copies* parameter and the *io.sort.factor* parameter will be appended to the other server-params or if they already exist in server-params, will override them. In order to specify these are *final* parameters, you can use:

$ hod allocate -d cluster\_dir -n number\_of\_nodes -Fmapred.reduce.parallel.copies=20 -Fio.sort.factor=100

However, note that final parameters cannot be overwritten from command line. They can only be appended if not already specified.

Similar options exist for configuring dynamically provisioned HDFS daemons. For doing so, replace -M with -H and -F with -S.

**Configuring Hadoop Job Submission (Client) Programs**

As mentioned above, if the allocation operation completes successfully then cluster\_dir/hadoop-site.xml will be generated and will contain information about the allocated cluster's JobTracker and NameNode. This configuration is used when submitting jobs to the cluster. HOD provides an option to include additional Hadoop configuration parameters into this file. The syntax for doing so is as follows:

$ hod allocate -d cluster\_dir -n number\_of\_nodes -Cmapred.userlog.limit.kb=200 -Cmapred.child.java.opts=-Xmx512m

In this example, the *mapred.userlog.limit.kb* and *mapred.child.java.opts* options will be included into the hadoop-site.xml that is generated by HOD.

### Viewing Hadoop Web-UIs

The HOD allocation operation prints the JobTracker and NameNode web UI URLs. For example:

$ hod allocate -d ~/hadoop-cluster -n 10 -c ~/hod-conf-dir/hodrc
  
INFO - HDFS UI on http://host242.foo.com:55391
  
INFO - Mapred UI on http://host521.foo.com:54874

The same information is also available via the *info* operation described above.

### Collecting and Viewing Hadoop Logs

To get the Hadoop logs of the daemons running on one of the allocated nodes:

- Log into the node of interest. If you want to look at the logs of the JobTracker or NameNode, then you can find the node running these by using the *list* and *info* operations mentioned above.
- Get the process information of the daemon of interest (for example, ps ux | grep TaskTracker)
- In the process information, search for the value of the variable -Dhadoop.log.dir. Typically this will be a decendent directory of the hodring.temp-dir value from the hod configuration file.
- Change to the hadoop.log.dir directory to view daemon and user logs.

HOD also provides a mechanism to collect logs when a cluster is being deallocated and persist them into a file system, or an externally configured HDFS. By doing so, these logs can be viewed after the jobs are completed and the nodes are released. In order to do so, configure the log-destination-uri to a URI as follows:

log-destination-uri = hdfs://host123:45678/user/hod/logs or

log-destination-uri = file://path/to/store/log/files

Under the root directory specified above in the path, HOD will create a path user\_name/torque\_jobid and store gzipped log files for each node that was part of the job.

Note that to store the files to HDFS, you may need to configure the hodring.pkgs option with the Hadoop version that matches the HDFS mentioned. If not, HOD will try to use the Hadoop version that it is using to provision the Hadoop cluster itself.

### Auto-deallocation of Idle Clusters

HOD automatically deallocates clusters that are not running Hadoop jobs for a given period of time. Each HOD allocation includes a monitoring facility that constantly checks for running Hadoop jobs. If it detects no running Hadoop jobs for a given period, it will automatically deallocate its own cluster and thus free up nodes which are not being used effectively.

*Note:* While the cluster is deallocated, the *cluster directory* is not cleaned up automatically. The user must deallocate this cluster through the regular *deallocate* operation to clean this up.

### Specifying Additional Job Attributes

HOD allows the user to specify a wallclock time and a name (or title) for a Torque job.

The wallclock time is the estimated amount of time for which the Torque job will be valid. After this time has expired, Torque will automatically delete the job and free up the nodes. Specifying the wallclock time can also help the job scheduler to better schedule jobs, and help improve utilization of cluster resources.

To specify the wallclock time, use the following syntax:

$ hod allocate -d cluster\_dir -n number\_of\_nodes -l time\_in\_seconds

The name or title of a Torque job helps in user friendly identification of the job. The string specified here will show up in all information where Torque job attributes are displayed, including the qstat command.

To specify the name or title, use the following syntax:

$ hod allocate -d cluster\_dir -n number\_of\_nodes -N name\_of\_job

*Note:* Due to restriction in the underlying Torque resource manager, names which do not start with an alphabet character or contain a 'space' will cause the job to fail. The failure message points to the problem being in the specified job name.

### Capturing HOD exit codes in Torque

HOD exit codes are captured in the Torque exit\_status field. This will help users and system administrators to distinguish successful runs from unsuccessful runs of HOD. The exit codes are 0 if allocation succeeded and all hadoop jobs ran on the allocated cluster correctly. They are non-zero if allocation failed or some of the hadoop jobs failed on the allocated cluster. The exit codes that are possible are mentioned in the table below. *Note: Hadoop job status is captured only if the version of Hadoop used is 16 or above.*

|  |  |
| --- | --- |
| Exit Code | Meaning |
| 6 | Ringmaster failure |
| 7 | DFS failure |
| 8 | Job tracker failure |
| 10 | Cluster dead |
| 12 | Cluster already allocated |
| 13 | HDFS dead |
| 14 | Mapred dead |
| 16 | All Map/Reduce jobs that ran on the cluster failed. Refer to hadoop logs for more details. |
| 17 | Some of the Map/Reduce jobs that ran on the cluster failed. Refer to hadoop logs for more details. |

### Command Line

HOD command line has the following general syntax:  
*hod <operation> [ARGS] [OPTIONS]* 
Allowed operations are 'allocate', 'deallocate', 'info', 'list', 'script' and 'help'. For help on a particular operation one can do : hod help <operation>. To have a look at possible options one can do a hod help options.

*allocate*
  
*Usage : hod allocate -d cluster\_dir -n number\_of\_nodes [OPTIONS]*
  
Allocates a cluster on the given number of cluster nodes, and store the allocation information in cluster\_dir for use with subsequent hadoop commands. Note that the cluster\_dir must exist before running the command.

*list*
  
*Usage : hod list [OPTIONS]*
  
Lists the clusters allocated by this user. Information provided includes the Torque job id corresponding to the cluster, the cluster directory where the allocation information is stored, and whether the Map/Reduce daemon is still active or not.

*info*
  
*Usage : hod info -d cluster\_dir [OPTIONS]*
  
Lists information about the cluster whose allocation information is stored in the specified cluster directory.

*deallocate*
  
*Usage : hod deallocate -d cluster\_dir [OPTIONS]*
  
Deallocates the cluster whose allocation information is stored in the specified cluster directory.

*script*
  
*Usage : hod script -s script\_file -d cluster\_directory -n number\_of\_nodes [OPTIONS]*
  
Runs a hadoop script using HOD*script* operation. Provisions Hadoop on a given number of nodes, executes the given script from the submitting node, and deallocates the cluster when the script completes.

*help*
  
*Usage : hod help [operation | 'options']*
  
When no argument is specified, hod help gives the usage and basic options, and is equivalent to hod --help (See below). When 'options' is given as argument, hod displays only the basic options that hod takes. When an operation is specified, it displays the usage and description corresponding to that particular operation. For e.g, to know about allocate operation, one can do a hod help allocate

Besides the operations, HOD can take the following command line options.

*--help*
  
Prints out the help message to see the usage and basic options.

*--verbose-help*
  
All configuration options provided in the hodrc file can be passed on the command line, using the syntax --section\_name.option\_name[=value]. When provided this way, the value provided on command line overrides the option provided in hodrc. The verbose-help command lists all the available options in the hodrc file. This is also a nice way to see the meaning of the configuration options.

See the next section for a description of most important hod configuration options. For basic options, one can do a hod help options and for all options possible in hod configuration, one can see hod --verbose-help. See config guide for a description of all options.

### Options Configuring HOD

As described above, HOD is configured using a configuration file that is usually set up by system administrators. This is a INI style configuration file that is divided into sections, and options inside each section. Each section relates to one of the HOD processes: client, ringmaster, hodring, mapreduce or hdfs. The options inside a section comprise of an option name and value.

Users can override the configuration defined in the default configuration in two ways:

- Users can supply their own configuration file to HOD in each of the commands, using the -c option
- Users can supply specific configuration options to HOD/ Options provided on command line *override* the values provided in the configuration file being used.

This section describes some of the most commonly used configuration options. These commonly used options are provided with a *short* option for convenience of specification. All other options can be specified using a *long* option that is also described below.

*-c config\_file*
  
Provides the configuration file to use. Can be used with all other options of HOD. Alternatively, the HOD\_CONF\_DIR environment variable can be defined to specify a directory that contains a file named hodrc, alleviating the need to specify the configuration file in each HOD command.

*-d cluster\_dir*
  
This is required for most of the hod operations. As described here, the *cluster directory* is a directory on the local file system where hod will generate the Hadoop configuration, *hadoop-site.xml*, corresponding to the cluster it allocates. Pass it to the hod operations as an argument to -d or --hod.clusterdir. If it doesn't already exist, HOD will automatically try to create it and use it. Once a cluster is allocated, a user can utilize it to run Hadoop jobs by specifying the clusterdirectory as the Hadoop --config option.

*-n number\_of\_nodes*
  
This is required for the hod 'allocation' operation and for script operation. This denotes the number of nodes to be allocated.

*-s script-file*
  
Required when using script operation, specifies the script file to execute.

*-b 1|2|3|4*
  
Enables the given debug level. Can be used with all other options of HOD. 4 is most verbose.

*-t hadoop\_tarball*
  
Provisions Hadoop from the given tar.gz file. This option is only applicable to the *allocate* operation. For better distribution performance it is strongly recommended that the Hadoop tarball is created *after* removing the source or documentation.

*-N job-name*
  
The Name to give to the resource manager job that HOD uses underneath. For e.g. in the case of Torque, this translates to the qsub -N option, and can be seen as the job name using the qstat command.

*-l wall-clock-time*
  
The amount of time for which the user expects to have work on the allocated cluster. This is passed to the resource manager underneath HOD, and can be used in more efficient scheduling and utilization of the cluster. Note that in the case of Torque, the cluster is automatically deallocated after this time expires.

*-j java-home*
  
Path to be set to the JAVA\_HOME environment variable. This is used in the *script* operation. HOD sets the JAVA\_HOME environment variable tot his value and launches the user script in that.

*-A account-string*
  
Accounting information to pass to underlying resource manager.

*-Q queue-name*
  
Name of the queue in the underlying resource manager to which the job must be submitted.

*-Mkey1=value1 -Mkey2=value2*
  
Provides configuration parameters for the provisioned Map/Reduce daemons (JobTracker and TaskTrackers). A hadoop-site.xml is generated with these values on the cluster nodes.   
*Note:* Values which have the following characters: space, comma, equal-to, semi-colon need to be escaped with a '\' character, and need to be enclosed within quotes. You can escape a '\' with a '\' too.

*-Hkey1=value1 -Hkey2=value2*
  
Provides configuration parameters for the provisioned HDFS daemons (NameNode and DataNodes). A hadoop-site.xml is generated with these values on the cluster nodes   
*Note:* Values which have the following characters: space, comma, equal-to, semi-colon need to be escaped with a '\' character, and need to be enclosed within quotes. You can escape a '\' with a '\' too.

*-Ckey1=value1 -Ckey2=value2*
  
Provides configuration parameters for the client from where jobs can be submitted. A hadoop-site.xml is generated with these values on the submit node.   
*Note:* Values which have the following characters: space, comma, equal-to, semi-colon need to be escaped with a '\' character, and need to be enclosed within quotes. You can escape a '\' with a '\' too.

*--section-name.option-name=value*
  
This is the method to provide options using the *long* format. For e.g. you could say *--hod.script-wait-time=20*

## Troubleshooting

The following section identifies some of the most likely error conditions users can run into when using HOD and ways to trouble-shoot them

### hod Hangs During Allocation

*Possible Cause:* One of the HOD or Hadoop components have failed to come up. In such a case, the hod command will return after a few minutes (typically 2-3 minutes) with an error code of either 7 or 8 as defined in the Error Codes section. Refer to that section for further details.

*Possible Cause:* A large allocation is fired with a tarball. Sometimes due to load in the network, or on the allocated nodes, the tarball distribution might be significantly slow and take a couple of minutes to come back. Wait for completion. Also check that the tarball does not have the Hadoop sources or documentation.

*Possible Cause:* A Torque related problem. If the cause is Torque related, the hod command will not return for more than 5 minutes. Running hod in debug mode may show the qstat command being executed repeatedly. Executing the qstat command from a separate shell may show that the job is in the Q (Queued) state. This usually indicates a problem with Torque. Possible causes could include some nodes being down, or new nodes added that Torque is not aware of. Generally, system administator help is needed to resolve this problem.

### hod Hangs During Deallocation

*Possible Cause:* A Torque related problem, usually load on the Torque server, or the allocation is very large. Generally, waiting for the command to complete is the only option.

### hod Fails With an error code and error message

If the exit code of the hod command is not 0, then refer to the following table of error exit codes to determine why the code may have occurred and how to debug the situation.

**Error Codes**

| Error Code | Meaning | Possible Causes and Remedial Actions |
| --- | --- | --- |
| 1 | Configuration error | Incorrect configuration values specified in hodrc, or other errors related to HOD configuration. The error messages in this case must be sufficient to debug and fix the problem. |
| 2 | Invalid operation | Do hod help for the list of valid operations. |
| 3 | Invalid operation arguments | Do hod help operation for listing the usage of a particular operation. |
| 4 | Scheduler failure | 1. Requested more resources than available. Run checknodes cluster\_name to see if enough nodes are available.   2. Requested resources exceed resource manager limits.   3. Torque is misconfigured, the path to Torque binaries is misconfigured, or other Torque problems. Contact system administrator. |
| 5 | Job execution failure | 1. Torque Job was deleted from outside. Execute the Torque qstat command to see if you have any jobs in the R (Running) state. If none exist, try re-executing HOD.   2. Torque problems such as the server momentarily going down, or becoming unresponsive. Contact system administrator. |
| 6 | Ringmaster failure | HOD prints the message "Cluster could not be allocated because of the following errors on the ringmaster host <hostname>". The actual error message may indicate one of the following:  1. Invalid configuration on the node running the ringmaster, specified by the hostname in the error message.  2. Invalid configuration in the ringmaster section,  3. Invalid pkgs option in gridservice-mapred or gridservice-hdfs section,  4. An invalid hadoop tarball, or a tarball which has bundled an invalid configuration file in the conf directory,  5. Mismatched version in Hadoop between the MapReduce and an external HDFS.  The Torque qstat command will most likely show a job in the C (Completed) state.   One can login to the ringmaster host as given by HOD failure message and debug the problem with the help of the error message. If the error message doesn't give complete information, ringmaster logs should help finding out the root cause of the problem. Refer to the section *Locating Ringmaster Logs* below for more information. |
| 7 | DFS failure | When HOD fails to allocate due to DFS failures (or Job tracker failures, error code 8, see below), it prints a failure message "Hodring at <hostname> failed with following errors:" and then gives the actual error message, which may indicate one of the following:  1. Problem in starting Hadoop clusters. Usually the actual cause in the error message will indicate the problem on the hostname mentioned. Also, review the Hadoop related configuration in the HOD configuration files. Look at the Hadoop logs using information specified in *Collecting and Viewing Hadoop Logs* section above.   2. Invalid configuration on the node running the hodring, specified by the hostname in the error message   3. Invalid configuration in the hodring section of hodrc. ssh to the hostname specified in the error message and grep for ERROR or CRITICAL in hodring logs. Refer to the section *Locating Hodring Logs* below for more information.   4. Invalid tarball specified which is not packaged correctly.   5. Cannot communicate with an externally configured HDFS.  When such DFS or Job tracker failure occurs, one can login into the host with hostname mentioned in HOD failure message and debug the problem. While fixing the problem, one should also review other log messages in the ringmaster log to see which other machines also might have had problems bringing up the jobtracker/namenode, apart from the hostname that is reported in the failure message. This possibility of other machines also having problems occurs because HOD continues to try and launch hadoop daemons on multiple machines one after another depending upon the value of the configuration variable ringmaster.max-master-failures. Refer to the section *Locating Ringmaster Logs* below to find more about ringmaster logs. |
| 8 | Job tracker failure | Similar to the causes in *DFS failure* case. |
| 10 | Cluster dead | 1. Cluster was auto-deallocated because it was idle for a long time.   2. Cluster was auto-deallocated because the wallclock time specified by the system administrator or user was exceeded.   3. Cannot communicate with the JobTracker and HDFS NameNode which were successfully allocated. Deallocate the cluster, and allocate again. |
| 12 | Cluster already allocated | The cluster directory specified has been used in a previous allocate operation and is not yet deallocated. Specify a different directory, or deallocate the previous allocation first. |
| 13 | HDFS dead | Cannot communicate with the HDFS NameNode. HDFS NameNode went down. |
| 14 | Mapred dead | 1. Cluster was auto-deallocated because it was idle for a long time.   2. Cluster was auto-deallocated because the wallclock time specified by the system administrator or user was exceeded.   3. Cannot communicate with the Map/Reduce JobTracker. JobTracker node went down. |
| 15 | Cluster not allocated | An operation which requires an allocated cluster is given a cluster directory with no state information. |
| Any non-zero exit code | HOD script error | If the hod script option was used, it is likely that the exit code is from the script. Unfortunately, this could clash with the exit codes of the hod command itself. In order to help users differentiate these two, hod writes the script's exit code to a file called script.exitcode in the cluster directory, if the script returned an exit code. You can cat this file to determine the script's exit code. If it does not exist, then it is a hod command exit code. |

### Hadoop Jobs Not Running on a Successfully Allocated Cluster

This scenario generally occurs when a cluster is allocated, and is left inactive for sometime, and then hadoop jobs are attempted to be run on them. Then Hadoop jobs fail with the following exception:

08/01/25 16:31:40 INFO ipc.Client: Retrying connect to server: foo.bar.com/1.1.1.1:53567. Already tried 1 time(s).

*Possible Cause:* No Hadoop jobs were run for a significant portion of time. Thus the cluster would have got deallocated as described in the section *Auto-deallocation of Idle Clusters*. Deallocate the cluster and allocate it again.

*Possible Cause:* The wallclock limit specified by the Torque administrator or the -l option defined in the section *Specifying Additional Job Attributes* was exceeded since allocation time. Thus the cluster would have got released. Deallocate the cluster and allocate it again.

*Possible Cause:* There is a version mismatch between the version of the hadoop being used in provisioning (typically via the tarball option) and the external HDFS. Ensure compatible versions are being used.

*Possible Cause:* There is a version mismatch between the version of the hadoop client being used to submit jobs and the hadoop used in provisioning (typically via the tarball option). Ensure compatible versions are being used.

*Possible Cause:* You used one of the options for specifying Hadoop configuration -M or -H, which had special characters like space or comma that were not escaped correctly. Refer to the section *Options Configuring HOD* for checking how to specify such options correctly.

### My Hadoop Job Got Killed

*Possible Cause:* The wallclock limit specified by the Torque administrator or the -l option defined in the section *Specifying Additional Job Attributes* was exceeded since allocation time. Thus the cluster would have got released. Deallocate the cluster and allocate it again, this time with a larger wallclock time.

*Possible Cause:* Problems with the JobTracker node. Refer to the section in *Collecting and Viewing Hadoop Logs* to get more information.

### Hadoop Job Fails with Message: 'Job tracker still initializing'

*Possible Cause:* The hadoop job was being run as part of the HOD script command, and it started before the JobTracker could come up fully. Allocate the cluster using a large value for the configuration option --hod.script-wait-time. Typically a value of 120 should work, though it is typically unnecessary to be that large.

### The Exit Codes For HOD Are Not Getting Into Torque

*Possible Cause:* Version 0.16 of hadoop is required for this functionality to work. The version of Hadoop used does not match. Use the required version of Hadoop.

*Possible Cause:* The deallocation was done without using the hod command; for e.g. directly using qdel. When the cluster is deallocated in this manner, the HOD processes are terminated using signals. This results in the exit code to be based on the signal number, rather than the exit code of the program.

### The Hadoop Logs are Not Uploaded to DFS

*Possible Cause:* There is a version mismatch between the version of the hadoop being used for uploading the logs and the external HDFS. Ensure that the correct version is specified in the hodring.pkgs option.

### Locating Ringmaster Logs

To locate the ringmaster logs, follow these steps:

- Execute hod in the debug mode using the -b option. This will print the Torque job id for the current run.
- Execute qstat -f torque\_job\_id and look up the value of the exec\_host parameter in the output. The first host in this list is the ringmaster node.
- Login to this node.
- The ringmaster log location is specified by the ringmaster.log-dir option in the hodrc. The name of the log file will be username.torque\_job\_id/ringmaster-main.log.
- If you don't get enough information, you may want to set the ringmaster debug level to 4. This can be done by passing --ringmaster.debug 4 to the hod command line.

### Locating Hodring Logs

To locate hodring logs, follow the steps below:

- Execute hod in the debug mode using the -b option. This will print the Torque job id for the current run.
- Execute qstat -f torque\_job\_id and look up the value of the exec\_host parameter in the output. All nodes in this list should have a hodring on them.
- Login to any of these nodes.
- The hodring log location is specified by the hodring.log-dir option in the hodrc. The name of the log file will be username.torque\_job\_id/hodring-main.log.
- If you don't get enough information, you may want to set the hodring debug level to 4. This can be done by passing --hodring.debug 4 to the hod command line.

Copyright ©
2007 The Apache Software Foundation.
