# Supplementary material for: Provenance based data integrity checking and verification in cloud environments
Source: PLoS One. 2017 May 17;12(5):e0177576. doi: 10.1371/journal.pone.0177576 (PMC5435237; doi:10.1371/journal.pone.0177576)
Supplement: S1 Dataset — Files in the Dataset are utilized for performance measurement. (ZIP) [file pone.0177576.s001.zip › dataset/html/index - Copy (2) - Copy.html]

Hadoop Documentation


Apache > Hadoop > Core

- Project
- Wiki
- Hadoop 0.18 Documentation

Documentation

Overview

Quickstart

Cluster Setup

HDFS Architecture

HDFS User Guide

HDFS Permissions Guide

HDFS Quotas Administrator Guide

Commands Manual

FS Shell Guide

DistCp Guide

Map-Reduce Tutorial

Native Hadoop Libraries

Streaming

Hadoop Archives

Hadoop On Demand

API Docs

Wiki

FAQ

Mailing Lists

Release Notes

All Changes

---


PDF

# Hadoop Documentation

The following documents provide concepts and procedures that will help you
get started using Hadoop. If you have more questions, you can ask the
mailing list or browse the archives.

- Hadoop Quickstart
- Hadoop Cluster Setup
- Hadoop Distributed File System
- Hadoop Map-Reduce Tutorial
- Native Hadoop Libraries
- API Docs
- Wiki
- FAQ

Copyright ©
2007 The Apache Software Foundation.
