# Supplementary material for: Provenance based data integrity checking and verification in cloud environments
Source: PLoS One. 2017 May 17;12(5):e0177576. doi: 10.1371/journal.pone.0177576 (PMC5435237; doi:10.1371/journal.pone.0177576)
Supplement: S1 Dataset — Files in the Dataset are utilized for performance measurement. (ZIP) [file pone.0177576.s001.zip › dataset/html/linkmap - Copy (2) - Copy.html]

Site Linkmap Table of Contents


Apache > Hadoop > Core

- Project
- Wiki
- Hadoop 0.18 Documentation

Documentation

Overview

Quickstart

Cluster Setup

HDFS Architecture

HDFS User Guide

HDFS Permissions Guide

HDFS Quotas Administrator Guide

Commands Manual

FS Shell Guide

DistCp Guide

Map-Reduce Tutorial

Native Hadoop Libraries

Streaming

Hadoop Archives

Hadoop On Demand

API Docs

Wiki

FAQ

Mailing Lists

Release Notes

All Changes


PDF

# Site Linkmap Table of Contents

This is a map of the complete site and its structure.

- Hadoop  \_\_\_\_\_\_\_\_\_\_\_\_\_\_\_\_\_\_\_  *site*

- Documentation  \_\_\_\_\_\_\_\_\_\_\_\_\_\_\_\_\_\_\_  *docs*

- Overview  \_\_\_\_\_\_\_\_\_\_\_\_\_\_\_\_\_\_\_  *overview*

- Quickstart  \_\_\_\_\_\_\_\_\_\_\_\_\_\_\_\_\_\_\_  *quickstart*

- Cluster Setup  \_\_\_\_\_\_\_\_\_\_\_\_\_\_\_\_\_\_\_  *setup*

- HDFS Architecture  \_\_\_\_\_\_\_\_\_\_\_\_\_\_\_\_\_\_\_  *hdfs*

- HDFS User Guide  \_\_\_\_\_\_\_\_\_\_\_\_\_\_\_\_\_\_\_  *hdfs*

- HDFS Permissions Guide  \_\_\_\_\_\_\_\_\_\_\_\_\_\_\_\_\_\_\_  *hdfs*

- HDFS Quotas Administrator Guide  \_\_\_\_\_\_\_\_\_\_\_\_\_\_\_\_\_\_\_  *hdfs*

- Commands Manual  \_\_\_\_\_\_\_\_\_\_\_\_\_\_\_\_\_\_\_  *commands*

- FS Shell Guide  \_\_\_\_\_\_\_\_\_\_\_\_\_\_\_\_\_\_\_  *fs*

- DistCp Guide  \_\_\_\_\_\_\_\_\_\_\_\_\_\_\_\_\_\_\_  *distcp*

- Map-Reduce Tutorial  \_\_\_\_\_\_\_\_\_\_\_\_\_\_\_\_\_\_\_  *mapred*

- Native Hadoop Libraries  \_\_\_\_\_\_\_\_\_\_\_\_\_\_\_\_\_\_\_  *mapred*

- Streaming  \_\_\_\_\_\_\_\_\_\_\_\_\_\_\_\_\_\_\_  *streaming*

- Hadoop Archives  \_\_\_\_\_\_\_\_\_\_\_\_\_\_\_\_\_\_\_  *archives*

- Hadoop On Demand  \_\_\_\_\_\_\_\_\_\_\_\_\_\_\_\_\_\_\_  *hod*

- API Docs  \_\_\_\_\_\_\_\_\_\_\_\_\_\_\_\_\_\_\_  *api*

- Wiki  \_\_\_\_\_\_\_\_\_\_\_\_\_\_\_\_\_\_\_  *wiki*

- FAQ  \_\_\_\_\_\_\_\_\_\_\_\_\_\_\_\_\_\_\_  *faq*

- Mailing Lists  \_\_\_\_\_\_\_\_\_\_\_\_\_\_\_\_\_\_\_  *lists*

- Release Notes  \_\_\_\_\_\_\_\_\_\_\_\_\_\_\_\_\_\_\_  *relnotes*

- All Changes  \_\_\_\_\_\_\_\_\_\_\_\_\_\_\_\_\_\_\_  *changes*

Copyright ©
2007 The Apache Software Foundation.
