# Supplementary material for: Provenance based data integrity checking and verification in cloud environments
Source: PLoS One. 2017 May 17;12(5):e0177576. doi: 10.1371/journal.pone.0177576 (PMC5435237; doi:10.1371/journal.pone.0177576)
Supplement: S1 Dataset — Files in the Dataset are utilized for performance measurement. (ZIP) [file pone.0177576.s001.zip › dataset/html/mapred_tutorial - Copy (2) - Copy.html]

Hadoop Map/Reduce Tutorial


Apache > Hadoop > Core

- Project
- Wiki
- Hadoop 0.18 Documentation

Documentation

Overview

Quickstart

Cluster Setup

HDFS Architecture

HDFS User Guide

HDFS Permissions Guide

HDFS Quotas Administrator Guide

Commands Manual

FS Shell Guide

DistCp Guide

Map-Reduce Tutorial

Native Hadoop Libraries

Streaming

Hadoop Archives

Hadoop On Demand

API Docs

Wiki

FAQ

Mailing Lists

Release Notes

All Changes


PDF

# Hadoop Map/Reduce Tutorial

- Purpose
- Pre-requisites
- Overview
- Inputs and Outputs
- Example: WordCount v1.0
  - Source Code
  - Usage
  - Walk-through
- Map/Reduce - User Interfaces
  - Payload
    - Mapper
    - Reducer
    - Partitioner
    - Reporter
    - OutputCollector
  - Job Configuration
  - Task Execution & Environment
  - Job Submission and Monitoring
    - Job Control
  - Job Input
    - InputSplit
    - RecordReader
  - Job Output
    - Task Side-Effect Files
    - RecordWriter
  - Other Useful Features
    - Counters
    - DistributedCache
    - Tool
    - IsolationRunner
    - Profiling
    - Debugging
    - JobControl
    - Data Compression
- Example: WordCount v2.0
  - Source Code
  - Sample Runs
  - Highlights

## Purpose

This document comprehensively describes all user-facing facets of the
Hadoop Map/Reduce framework and serves as a tutorial.

## Pre-requisites

Ensure that Hadoop is installed, configured and is running. More
details:

- Hadoop Quickstart for first-time users.
- Hadoop Cluster Setup for large,
  distributed clusters.

## Overview

Hadoop Map/Reduce is a software framework for easily writing
applications which process vast amounts of data (multi-terabyte data-sets)
in-parallel on large clusters (thousands of nodes) of commodity
hardware in a reliable, fault-tolerant manner.

A Map/Reduce *job* usually splits the input data-set into
independent chunks which are processed by the *map tasks* in a
completely parallel manner. The framework sorts the outputs of the maps,
which are then input to the *reduce tasks*. Typically both the
input and the output of the job are stored in a file-system. The framework
takes care of scheduling tasks, monitoring them and re-executes the failed
tasks.

Typically the compute nodes and the storage nodes are the same, that is,
the Map/Reduce framework and the Distributed
FileSystem are running on the same set of nodes. This configuration
allows the framework to effectively schedule tasks on the nodes where data
is already present, resulting in very high aggregate bandwidth across the
cluster.

The Map/Reduce framework consists of a single master
JobTracker and one slave TaskTracker per
cluster-node. The master is responsible for scheduling the jobs' component
tasks on the slaves, monitoring them and re-executing the failed tasks. The
slaves execute the tasks as directed by the master.

Minimally, applications specify the input/output locations and supply
*map* and *reduce* functions via implementations of
appropriate interfaces and/or abstract-classes. These, and other job
parameters, comprise the *job configuration*. The Hadoop
*job client* then submits the job (jar/executable etc.) and
configuration to the JobTracker which then assumes the
responsibility of distributing the software/configuration to the slaves,
scheduling tasks and monitoring them, providing status and diagnostic
information to the job-client.

Although the Hadoop framework is implemented in Java^TM^,
Map/Reduce applications need not be written in Java.

- Hadoop Streaming is a utility which allows users to create and run
  jobs with any executables (e.g. shell utilities) as the mapper and/or
  the reducer.
- Hadoop Pipes is a SWIG-
  compatible *C++ API* to implement Map/Reduce applications (non
  JNI^TM^ based).

## Inputs and Outputs

The Map/Reduce framework operates exclusively on
<key, value> pairs, that is, the framework views the
input to the job as a set of <key, value> pairs and
produces a set of <key, value> pairs as the output of
the job, conceivably of different types.

The key and value classes have to be
serializable by the framework and hence need to implement the
Writable
interface. Additionally, the key classes have to implement the
WritableComparable interface to facilitate sorting by the framework.

Input and Output types of a Map/Reduce job:

(input) <k1, v1>
->
**map**
->
<k2, v2>
->
**combine**
->
<k2, v2>
->
**reduce**
->
<k3, v3> (output)

## Example: WordCount v1.0

Before we jump into the details, lets walk through an example Map/Reduce
application to get a flavour for how they work.

WordCount is a simple application that counts the number of
occurences of each word in a given input set.

This works with a
local-standalone,
pseudo-distributed or
fully-distributed
Hadoop installation.

### Source Code

|  | WordCount.java |
| --- | --- |
| 1. | package org.myorg; |
| 2. |  |
| 3. | import java.io.IOException; |
| 4. | import java.util.\*; |
| 5. |  |
| 6. | import org.apache.hadoop.fs.Path; |
| 7. | import org.apache.hadoop.conf.\*; |
| 8. | import org.apache.hadoop.io.\*; |
| 9. | import org.apache.hadoop.mapred.\*; |
| 10. | import org.apache.hadoop.util.\*; |
| 11. |  |
| 12. | public class WordCount { |
| 13. |  |
| 14. | public static class Map extends MapReduceBase implements Mapper<LongWritable, Text, Text, IntWritable> { |
| 15. | private final static IntWritable one = new IntWritable(1); |
| 16. | private Text word = new Text(); |
| 17. |  |
| 18. | public void map(LongWritable key, Text value, OutputCollector<Text, IntWritable> output, Reporter reporter) throws IOException { |
| 19. | String line = value.toString(); |
| 20. | StringTokenizer tokenizer = new StringTokenizer(line); |
| 21. | while (tokenizer.hasMoreTokens()) { |
| 22. | word.set(tokenizer.nextToken()); |
| 23. | output.collect(word, one); |
| 24. | } |
| 25. | } |
| 26. | } |
| 27. |  |
| 28. | public static class Reduce extends MapReduceBase implements Reducer<Text, IntWritable, Text, IntWritable> { |
| 29. | public void reduce(Text key, Iterator<IntWritable> values, OutputCollector<Text, IntWritable> output, Reporter reporter) throws IOException { |
| 30. | int sum = 0; |
| 31. | while (values.hasNext()) { |
| 32. | sum += values.next().get(); |
| 33. | } |
| 34. | output.collect(key, new IntWritable(sum)); |
| 35. | } |
| 36. | } |
| 37. |  |
| 38. | public static void main(String[] args) throws Exception { |
| 39. | JobConf conf = new JobConf(WordCount.class); |
| 40. | conf.setJobName("wordcount"); |
| 41. |  |
| 42. | conf.setOutputKeyClass(Text.class); |
| 43. | conf.setOutputValueClass(IntWritable.class); |
| 44. |  |
| 45. | conf.setMapperClass(Map.class); |
| 46. | conf.setCombinerClass(Reduce.class); |
| 47. | conf.setReducerClass(Reduce.class); |
| 48. |  |
| 49. | conf.setInputFormat(TextInputFormat.class); |
| 50. | conf.setOutputFormat(TextOutputFormat.class); |
| 51. |  |
| 52. | FileInputFormat.setInputPaths(conf, new Path(args[0])); |
| 53. | FileOutputFormat.setOutputPath(conf, new Path(args[1])); |
| 54. |  |
| 55. | JobClient.runJob(conf); |
| 57. | } |
| 58. | } |
| 59. |  |

### Usage

Assuming HADOOP\_HOME is the root of the installation and
HADOOP\_VERSION is the Hadoop version installed, compile
WordCount.java and create a jar:

$ mkdir wordcount\_classes
  

$ javac -classpath ${HADOOP\_HOME}/hadoop-${HADOOP\_VERSION}-core.jar
-d wordcount\_classes WordCount.java
  
$ jar -cvf /usr/joe/wordcount.jar -C wordcount\_classes/ .

Assuming that:

- /usr/joe/wordcount/input - input directory in HDFS
- /usr/joe/wordcount/output - output directory in HDFS

Sample text-files as input:

$ bin/hadoop dfs -ls /usr/joe/wordcount/input/
  
/usr/joe/wordcount/input/file01
  
/usr/joe/wordcount/input/file02
  
  
$ bin/hadoop dfs -cat /usr/joe/wordcount/input/file01
  
Hello World Bye World
  
  
$ bin/hadoop dfs -cat /usr/joe/wordcount/input/file02
  
Hello Hadoop Goodbye Hadoop

Run the application:

$ bin/hadoop jar /usr/joe/wordcount.jar org.myorg.WordCount
/usr/joe/wordcount/input /usr/joe/wordcount/output

Output:

$ bin/hadoop dfs -cat /usr/joe/wordcount/output/part-00000
  
Bye 1
  
Goodbye 1
  
Hadoop 2
  
Hello 2
  
World 2

Applications can specify a comma separated list of paths which
would be present in the current working directory of the task
using the option -files. The -libjars
option allows applications to add jars to the classpaths of the maps
and reduces. The -archives allows them to pass archives
as arguments that are unzipped/unjarred and a link with name of the
jar/zip are created in the current working directory of tasks. More
details about the command line options are available at
Commands manual

Running wordcount example with
-libjars and -files:  
 hadoop jar hadoop-examples.jar wordcount -files cachefile.txt
-libjars mylib.jar input output

### Walk-through

The WordCount application is quite straight-forward.

The Mapper implementation (lines 14-26), via the
map method (lines 18-25), processes one line at a time,
as provided by the specified TextInputFormat (line 49).
It then splits the line into tokens separated by whitespaces, via the
StringTokenizer, and emits a key-value pair of
< <word>, 1>.

For the given sample input the first map emits:  
< Hello, 1>
  
< World, 1>
  
< Bye, 1>
  
< World, 1>

The second map emits:  
< Hello, 1>
  
< Hadoop, 1>
  
< Goodbye, 1>
  
< Hadoop, 1>

We'll learn more about the number of maps spawned for a given job, and
how to control them in a fine-grained manner, a bit later in the
tutorial.

WordCount also specifies a combiner (line
46). Hence, the output of each map is passed through the local combiner
(which is same as the Reducer as per the job
configuration) for local aggregation, after being sorted on the
*key*s.

The output of the first map:  
< Bye, 1>
  
< Hello, 1>
  
< World, 2>

The output of the second map:  
< Goodbye, 1>
  
< Hadoop, 2>
  
< Hello, 1>

The Reducer implementation (lines 28-36), via the
reduce method (lines 29-35) just sums up the values,
which are the occurence counts for each key (i.e. words in this example).

Thus the output of the job is:  
< Bye, 1>
  
< Goodbye, 1>
  
< Hadoop, 2>
  
< Hello, 2>
  
< World, 2>

The run method specifies various facets of the job, such
as the input/output paths (passed via the command line), key/value
types, input/output formats etc., in the JobConf.
It then calls the JobClient.runJob (line 55) to submit the
and monitor its progress.

We'll learn more about JobConf, JobClient,
Tool and other interfaces and classes a bit later in the
tutorial.

## Map/Reduce - User Interfaces

This section provides a reasonable amount of detail on every user-facing
aspect of the Map/Reduce framwork. This should help users implement,
configure and tune their jobs in a fine-grained manner. However, please
note that the javadoc for each class/interface remains the most
comprehensive documentation available; this is only meant to be a tutorial.

Let us first take the Mapper and Reducer
interfaces. Applications typically implement them to provide the
map and reduce methods.

We will then discuss other core interfaces including
JobConf, JobClient, Partitioner,
OutputCollector, Reporter,
InputFormat, OutputFormat and others.

Finally, we will wrap up by discussing some useful features of the
framework such as the DistributedCache,
IsolationRunner etc.

### Payload

Applications typically implement the Mapper and
Reducer interfaces to provide the map and
reduce methods. These form the core of the job.

#### Mapper

Mapper maps input key/value pairs to a set of intermediate
key/value pairs.

Maps are the individual tasks that transform input records into
intermediate records. The transformed intermediate records do not need
to be of the same type as the input records. A given input pair may
map to zero or many output pairs.

The Hadoop Map/Reduce framework spawns one map task for each
InputSplit generated by the InputFormat for
the job.

Overall, Mapper implementations are passed the
JobConf for the job via the
JobConfigurable.configure(JobConf) method and override it to
initialize themselves. The framework then calls
map(WritableComparable, Writable, OutputCollector, Reporter) for
each key/value pair in the InputSplit for that task.
Applications can then override the
Closeable.close() method to perform any required cleanup.

Output pairs do not need to be of the same types as input pairs. A
given input pair may map to zero or many output pairs. Output pairs
are collected with calls to
OutputCollector.collect(WritableComparable,Writable).

Applications can use the Reporter to report
progress, set application-level status messages and update
Counters, or just indicate that they are alive.

All intermediate values associated with a given output key are
subsequently grouped by the framework, and passed to the
Reducer(s) to determine the final output. Users can
control the grouping by specifying a Comparator via
JobConf.setOutputKeyComparatorClass(Class).

The Mapper outputs are sorted and then
partitioned per Reducer. The total number of partitions is
the same as the number of reduce tasks for the job. Users can control
which keys (and hence records) go to which Reducer by
implementing a custom Partitioner.

Users can optionally specify a combiner, via
JobConf.setCombinerClass(Class), to perform local aggregation of
the intermediate outputs, which helps to cut down the amount of data
transferred from the Mapper to the Reducer.

The intermediate, sorted outputs are always stored in a simple
(key-len, key, value-len, value) format.
Applications can control if, and how, the
intermediate outputs are to be compressed and the
CompressionCodec to be used via the JobConf.

##### How Many Maps?

The number of maps is usually driven by the total size of the
inputs, that is, the total number of blocks of the input files.

The right level of parallelism for maps seems to be around 10-100
maps per-node, although it has been set up to 300 maps for very
cpu-light map tasks. Task setup takes awhile, so it is best if the
maps take at least a minute to execute.

Thus, if you expect 10TB of input data and have a blocksize of
128MB, you'll end up with 82,000 maps, unless
setNumMapTasks(int) (which only provides a hint to the framework)
is used to set it even higher.

#### Reducer

Reducer reduces a set of intermediate values which share a key to
a smaller set of values.

The number of reduces for the job is set by the user
via 
JobConf.setNumReduceTasks(int).

Overall, Reducer implementations are passed the
JobConf for the job via the
JobConfigurable.configure(JobConf) method and can override it to
initialize themselves. The framework then calls
reduce(WritableComparable, Iterator, OutputCollector, Reporter)
method for each <key, (list of values)>
pair in the grouped inputs. Applications can then override the
Closeable.close() method to perform any required cleanup.

Reducer has 3 primary phases: shuffle, sort and reduce.

##### Shuffle

Input to the Reducer is the sorted output of the
mappers. In this phase the framework fetches the relevant partition
of the output of all the mappers, via HTTP.

##### Sort

The framework groups Reducer inputs by keys (since
different mappers may have output the same key) in this stage.

The shuffle and sort phases occur simultaneously; while
map-outputs are being fetched they are merged.

##### Secondary Sort

If equivalence rules for grouping the intermediate keys are
required to be different from those for grouping keys before
reduction, then one may specify a Comparator via
JobConf.setOutputValueGroupingComparator(Class). Since
JobConf.setOutputKeyComparatorClass(Class) can be used to
control how intermediate keys are grouped, these can be used in
conjunction to simulate *secondary sort on values*.

##### Reduce

In this phase the
reduce(WritableComparable, Iterator, OutputCollector, Reporter)
method is called for each <key, (list of values)>
pair in the grouped inputs.

The output of the reduce task is typically written to the
FileSystem via
OutputCollector.collect(WritableComparable, Writable).

Applications can use the Reporter to report
progress, set application-level status messages and update
Counters, or just indicate that they are alive.

The output of the Reducer is *not sorted*.

##### How Many Reduces?

The right number of reduces seems to be 0.95 or
1.75 multiplied by (<*no. of nodes*> \*
mapred.tasktracker.reduce.tasks.maximum).

With 0.95 all of the reduces can launch immediately
and start transfering map outputs as the maps finish. With
1.75 the faster nodes will finish their first round of
reduces and launch a second wave of reduces doing a much better job
of load balancing.

Increasing the number of reduces increases the framework overhead,
but increases load balancing and lowers the cost of failures.

The scaling factors above are slightly less than whole numbers to
reserve a few reduce slots in the framework for speculative-tasks and
failed tasks.

##### Reducer NONE

It is legal to set the number of reduce-tasks to *zero* if
no reduction is desired.

In this case the outputs of the map-tasks go directly to the
FileSystem, into the output path set by
setOutputPath(Path). The framework does not sort the
map-outputs before writing them out to the FileSystem.

#### Partitioner

Partitioner partitions the key space.

Partitioner controls the partitioning of the keys of the
intermediate map-outputs. The key (or a subset of the key) is used to
derive the partition, typically by a *hash function*. The total
number of partitions is the same as the number of reduce tasks for the
job. Hence this controls which of the m reduce tasks the
intermediate key (and hence the record) is sent to for reduction.

HashPartitioner is the default Partitioner.

#### Reporter

Reporter is a facility for Map/Reduce applications to report
progress, set application-level status messages and update
Counters.

Mapper and Reducer implementations can use
the Reporter to report progress or just indicate
that they are alive. In scenarios where the application takes a
significant amount of time to process individual key/value pairs,
this is crucial since the framework might assume that the task has
timed-out and kill that task. Another way to avoid this is to
set the configuration parameter mapred.task.timeout to a
high-enough value (or even set it to *zero* for no time-outs).

Applications can also update Counters using the
Reporter.

#### OutputCollector

OutputCollector is a generalization of the facility provided by
the Map/Reduce framework to collect data output by the
Mapper or the Reducer (either the
intermediate outputs or the output of the job).

Hadoop Map/Reduce comes bundled with a
library of generally useful mappers, reducers, and partitioners.

### Job Configuration

JobConf represents a Map/Reduce job configuration.

JobConf is the primary interface for a user to describe
a Map/Reduce job to the Hadoop framework for execution. The framework
tries to faithfully execute the job as described by JobConf,
however:

- f
  Some configuration parameters may have been marked as
  final by administrators and hence cannot be altered.
- While some job parameters are straight-forward to set (e.g.
  setNumReduceTasks(int)), other parameters interact subtly with
  the rest of the framework and/or job configuration and are
  more complex to set (e.g.
  setNumMapTasks(int)).

JobConf is typically used to specify the
Mapper, combiner (if any), Partitioner,
Reducer, InputFormat and
OutputFormat implementations. JobConf also
indicates the set of input files
(setInputPaths(JobConf, Path...)
/addInputPath(JobConf, Path))
and (setInputPaths(JobConf, String)
/addInputPaths(JobConf, String))
and where the output files should be written
(setOutputPath(Path)).

Optionally, JobConf is used to specify other advanced
facets of the job such as the Comparator to be used, files
to be put in the DistributedCache, whether intermediate
and/or job outputs are to be compressed (and how), debugging via
user-provided scripts
(setMapDebugScript(String)/setReduceDebugScript(String))
, whether job tasks can be executed in a *speculative* manner
(setMapSpeculativeExecution(boolean))/(setReduceSpeculativeExecution(boolean))
, maximum number of attempts per task
(setMaxMapAttempts(int)/setMaxReduceAttempts(int))
, percentage of tasks failure which can be tolerated by the job
(setMaxMapTaskFailuresPercent(int)/setMaxReduceTaskFailuresPercent(int))
etc.

Of course, users can use
set(String, String)/get(String, String)
to set/get arbitrary parameters needed by applications. However, use the
DistributedCache for large amounts of (read-only) data.

### Task Execution & Environment

The TaskTracker executes the Mapper/
Reducer *task* as a child process in a separate jvm.

The child-task inherits the environment of the parent
TaskTracker. The user can specify additional options to the
child-jvm via the mapred.child.java.opts configuration
parameter in the JobConf such as non-standard paths for the
run-time linker to search shared libraries via
-Djava.library.path=<> etc. If the
mapred.child.java.opts contains the symbol *@taskid@*
it is interpolated with value of taskid of the map/reduce
task.

Here is an example with multiple arguments and substitutions,
showing jvm GC logging, and start of a passwordless JVM JMX agent so that
it can connect with jconsole and the likes to watch child memory,
threads and get thread dumps. It also sets the maximum heap-size of the
child jvm to 512MB and adds an additional path to the
java.library.path of the child-jvm.

<property>
  
  <name>mapred.child.java.opts</name>
  
  <value>
  
    
-Xmx512M -Djava.library.path=/home/mycompany/lib
-verbose:gc -Xloggc:/tmp/@taskid@.gc
  
    
-Dcom.sun.management.jmxremote.authenticate=false
-Dcom.sun.management.jmxremote.ssl=false
  
  </value>
  
</property>

Users/admins can also specify the maximum virtual memory
of the launched child-task using mapred.child.ulimit.
The value for mapred.child.ulimit should be specified
in kilo bytes (KB). And also the value must be greater than
or equal to the -Xmx passed to JavaVM, else the VM might not start.

Note: mapred.child.java.opts are used only for
configuring the launched child tasks from task tracker. Configuring
the memory options for daemons is documented in
cluster\_setup.html

The task tracker has local directory,
 ${mapred.local.dir}/taskTracker/ to create localized
cache and localized job. It can define multiple local directories
(spanning multiple disks) and then each filename is assigned to a
semi-random local directory. When the job starts, task tracker
creates a localized job directory relative to the local directory
specified in the configuration. Thus the task tracker directory
structure looks the following:

- ${mapred.local.dir}/taskTracker/archive/ :
  The distributed cache. This directory holds the localized distributed
  cache. Thus localized distributed cache is shared among all
  the tasks and jobs
- ${mapred.local.dir}/taskTracker/jobcache/$jobid/ :
  The localized job directory
  - ${mapred.local.dir}/taskTracker/jobcache/$jobid/work/
    : The job-specific shared directory. The tasks can use this space as
    scratch space and share files among them. This directory is exposed
    to the users through the configuration property
    job.local.dir. The directory can accessed through
    api 
    JobConf.getJobLocalDir(). It is available as System property also.
    So, users (streaming etc.) can call
    System.getProperty("job.local.dir") to access the
    directory.
  - ${mapred.local.dir}/taskTracker/jobcache/$jobid/jars/
    : The jars directory, which has the job jar file and expanded jar.
    The job.jar is the application's jar file that is
    automatically distributed to each machine. It is expanded in jars
    directory before the tasks for the job start. The job.jar location
    is accessible to the application through the api
    JobConf.getJar() . To access the unjarred directory,
    JobConf.getJar().getParent() can be called.
  - ${mapred.local.dir}/taskTracker/jobcache/$jobid/job.xml
    : The job.xml file, the generic job configuration, localized for
    the job.
  - ${mapred.local.dir}/taskTracker/jobcache/$jobid/$taskid
    : The task direcrory for each task attempt. Each task directory
    again has the following structure :
    - ${mapred.local.dir}/taskTracker/jobcache/$jobid/$taskid/job.xml
      : A job.xml file, task localized job configuration, Task localization
      means that properties have been set that are specific to
      this particular task within the job. The properties localized for
      each task are described below.
    - ${mapred.local.dir}/taskTracker/jobcache/$jobid/$taskid/output
      : A directory for intermediate output files. This contains the
      temporary map reduce data generated by the framework
      such as map output files etc.
    - ${mapred.local.dir}/taskTracker/jobcache/$jobid/$taskid/work
      : The curernt working directory of the task.
    - ${mapred.local.dir}/taskTracker/jobcache/$jobid/$taskid/work/tmp
      : The temporary directory for the task.
      (User can specify the property mapred.child.tmp to set
      the value of temporary directory for map and reduce tasks. This
      defaults to ./tmp. If the value is not an absolute path,
      it is prepended with task's working directory. Otherwise, it is
      directly assigned. The directory will be created if it doesn't exist.
      Then, the child java tasks are executed with option
      -Djava.io.tmpdir='the absolute path of the tmp dir'.
      Anp pipes and streaming are set with environment variable,
      TMPDIR='the absolute path of the tmp dir'). This
      directory is created, if mapred.child.tmp has the value
      ./tmp

The following properties are localized in the job configuration
for each task's execution:

| Name | Type | Description |
| --- | --- | --- |
| mapred.job.id | String | The job id |
| mapred.jar | String | job.jar location in job directory |
| job.local.dir | String | The job specific shared scratch space |
| mapred.tip.id | String | The task id |
| mapred.task.id | String | The task attempt id |
| mapred.task.is.map | boolean | Is this a map task |
| mapred.task.partition | int | The id of the task within the job |
| map.input.file | String | The filename that the map is reading from |
| map.input.start | long | The offset of the start of the map input split |
| map.input.length | long | The number of bytes in the map input split |
| mapred.work.output.dir | String | The task's temporary output directory |

The standard output (stdout) and error (stderr) streams of the task
are read by the TaskTracker and logged to
${HADOOP\_LOG\_DIR}/userlogs

The DistributedCache can also be used
to distribute both jars and native libraries for use in the map
and/or reduce tasks. The child-jvm always has its
*current working directory* added to the
java.library.path and LD\_LIBRARY\_PATH.
And hence the cached libraries can be loaded via
System.loadLibrary or
System.load. More details on how to load shared libraries through
distributed cache are documented at
native\_libraries.html

### Job Submission and Monitoring

JobClient is the primary interface by which user-job interacts
with the JobTracker.

JobClient provides facilities to submit jobs, track their
progress, access component-tasks' reports and logs, get the Map/Reduce
cluster's status information and so on.

The job submission process involves:

1. Checking the input and output specifications of the job.
2. Computing the InputSplit values for the job.
3. Setting up the requisite accounting information for the
   DistributedCache of the job, if necessary.
4. Copying the job's jar and configuration to the Map/Reduce system
   directory on the FileSystem.
5. Submitting the job to the JobTracker and optionally
   monitoring it's status.

Job history files are also logged to user specified directory
hadoop.job.history.user.location
which defaults to job output directory. The files are stored in
"\_logs/history/" in the specified directory. Hence, by default they
will be in mapred.output.dir/\_logs/history. User can stop
logging by giving the value none for
hadoop.job.history.user.location

User can view the history logs summary in specified directory
using the following command   
$ bin/hadoop job -history output-dir
  
This command will print job details, failed and killed tip
details.   
More details about the job such as successful tasks and
task attempts made for each task can be viewed using the
following command   
$ bin/hadoop job -history all output-dir

User can use
OutputLogFilter
to filter log files from the output directory listing.

Normally the user creates the application, describes various facets
of the job via JobConf, and then uses the
JobClient to submit the job and monitor its progress.

#### Job Control

Users may need to chain Map/Reduce jobs to accomplish complex
tasks which cannot be done via a single Map/Reduce job. This is fairly
easy since the output of the job typically goes to distributed
file-system, and the output, in turn, can be used as the input for the
next job.

However, this also means that the onus on ensuring jobs are
complete (success/failure) lies squarely on the clients. In such
cases, the various job-control options are:

- runJob(JobConf) : Submits the job and returns only after the
  job has completed.
- submitJob(JobConf) : Only submits the job, then poll the
  returned handle to the
  RunningJob to query status and make scheduling decisions.
- JobConf.setJobEndNotificationURI(String) : Sets up a
  notification upon job-completion, thus avoiding polling.

### Job Input

InputFormat describes the input-specification for a Map/Reduce job.

The Map/Reduce framework relies on the InputFormat of
the job to:

1. Validate the input-specification of the job.
2. Split-up the input file(s) into logical InputSplit
   instances, each of which is then assigned to an individual
   Mapper.
3. Provide the RecordReader implementation used to
   glean input records from the logical InputSplit for
   processing by the Mapper.

The default behavior of file-based InputFormat
implementations, typically sub-classes of
FileInputFormat, is to split the input into *logical*
InputSplit instances based on the total size, in bytes, of
the input files. However, the FileSystem blocksize of the
input files is treated as an upper bound for input splits. A lower bound
on the split size can be set via mapred.min.split.size.

Clearly, logical splits based on input-size is insufficient for many
applications since record boundaries must be respected. In such cases,
the application should implement a RecordReader, who is
responsible for respecting record-boundaries and presents a
record-oriented view of the logical InputSplit to the
individual task.

TextInputFormat is the default InputFormat.

If TextInputFormat is the InputFormat for a
given job, the framework detects input-files with the *.gz* and
*.lzo* extensions and automatically decompresses them using the
appropriate CompressionCodec. However, it must be noted that
compressed files with the above extensions cannot be *split* and
each compressed file is processed in its entirety by a single mapper.

#### InputSplit

InputSplit represents the data to be processed by an individual
Mapper.

Typically InputSplit presents a byte-oriented view of
the input, and it is the responsibility of RecordReader
to process and present a record-oriented view.

FileSplit is the default InputSplit. It sets
map.input.file to the path of the input file for the
logical split.

#### RecordReader

RecordReader reads <key, value> pairs from an
InputSplit.

Typically the RecordReader converts the byte-oriented
view of the input, provided by the InputSplit, and
presents a record-oriented to the Mapper implementations
for processing. RecordReader thus assumes the
responsibility of processing record boundaries and presents the tasks
with keys and values.

### Job Output

OutputFormat describes the output-specification for a Map/Reduce
job.

The Map/Reduce framework relies on the OutputFormat of
the job to:

1. Validate the output-specification of the job; for example, check that
   the output directory doesn't already exist.
2. Provide the RecordWriter implementation used to
   write the output files of the job. Output files are stored in a
   FileSystem.

TextOutputFormat is the default
OutputFormat.

#### Task Side-Effect Files

In some applications, component tasks need to create and/or write to
side-files, which differ from the actual job-output files.

In such cases there could be issues with two instances of the same
Mapper or Reducer running simultaneously (for
example, speculative tasks) trying to open and/or write to the same
file (path) on the FileSystem. Hence the
application-writer will have to pick unique names per task-attempt
(using the attemptid, say attempt\_200709221812\_0001\_m\_000000\_0),
not just per task.

To avoid these issues the Map/Reduce framework maintains a special
${mapred.output.dir}/\_temporary/\_${taskid} sub-directory
accessible via ${mapred.work.output.dir}
for each task-attempt on the FileSystem where the output
of the task-attempt is stored. On successful completion of the
task-attempt, the files in the
${mapred.output.dir}/\_temporary/\_${taskid} (only)
are *promoted* to ${mapred.output.dir}. Of course,
the framework discards the sub-directory of unsuccessful task-attempts.
This process is completely transparent to the application.

The application-writer can take advantage of this feature by
creating any side-files required in ${mapred.work.output.dir}
during execution of a task via
FileOutputFormat.getWorkOutputPath(), and the framework will promote them
similarly for succesful task-attempts, thus eliminating the need to
pick unique paths per task-attempt.

Note: The value of ${mapred.work.output.dir} during
execution of a particular task-attempt is actually
${mapred.output.dir}/\_temporary/\_{$taskid}, and this value is
set by the Map/Reduce framework. So, just create any side-files in the
path returned by
FileOutputFormat.getWorkOutputPath() from map/reduce
task to take advantage of this feature.

The entire discussion holds true for maps of jobs with
reducer=NONE (i.e. 0 reduces) since output of the map, in that case,
goes directly to HDFS.

#### RecordWriter

RecordWriter writes the output <key, value>
pairs to an output file.

RecordWriter implementations write the job outputs to the
FileSystem.

### Other Useful Features

#### Counters

Counters represent global counters, defined either by
the Map/Reduce framework or applications. Each Counter can
be of any Enum type. Counters of a particular
Enum are bunched into groups of type
Counters.Group.

Applications can define arbitrary Counters (of type
Enum) and update them via
Reporter.incrCounter(Enum, long) or
Reporter.incrCounter(String, String, long)
in the map and/or
reduce methods. These counters are then globally
aggregated by the framework.

#### DistributedCache

DistributedCache distributes application-specific, large, read-only
files efficiently.

DistributedCache is a facility provided by the
Map/Reduce framework to cache files (text, archives, jars and so on)
needed by applications.

Applications specify the files to be cached via urls (hdfs://)
in the JobConf. The DistributedCache
assumes that the files specified via hdfs:// urls are already present
on the FileSystem.

The framework will copy the necessary files to the slave node
before any tasks for the job are executed on that node. Its
efficiency stems from the fact that the files are only copied once
per job and the ability to cache archives which are un-archived on
the slaves.

DistributedCache tracks the modification timestamps of
the cached files. Clearly the cache files should not be modified by
the application or externally while the job is executing.

DistributedCache can be used to distribute simple,
read-only data/text files and more complex types such as archives and
jars. Archives (zip, tar, tgz and tar.gz files) are
*un-archived* at the slave nodes. Files
have *execution permissions* set.

The files/archives can be distributed by setting the property
mapred.cache.{files|archives}. If more than one
file/archive has to be distributed, they can be added as comma
separated paths. The properties can also be set by APIs
DistributedCache.addCacheFile(URI,conf)/
DistributedCache.addCacheArchive(URI,conf) and
DistributedCache.setCacheFiles(URIs,conf)/
DistributedCache.setCacheArchives(URIs,conf)
where URI is of the form
hdfs://host:port/absolute-path#link-name.
In Streaming, the files can be distributed through command line
option -cacheFile/-cacheArchive.

Optionally users can also direct the DistributedCache
to *symlink* the cached file(s) into the current working
directory of the task via the
DistributedCache.createSymlink(Configuration) api. Or by setting
the configuration property mapred.create.symlink
as yes. The DistributedCache will use the
fragment of the URI as the name of the symlink.
For example, the URI
hdfs://namenode:port/lib.so.1#lib.so
will have the symlink name as lib.so in task's cwd
for the file lib.so.1 in distributed cache.

The DistributedCache can also be used as a
rudimentary software distribution mechanism for use in the
map and/or reduce tasks. It can be used to distribute both
jars and native libraries. The
DistributedCache.addArchiveToClassPath(Path, Configuration) or
DistributedCache.addFileToClassPath(Path, Configuration) api
can be used to cache files/jars and also add them to the
*classpath* of child-jvm. The same can be done by setting
the configuration properties
mapred.job.classpath.{files|archives}. Similarly the
cached files that are symlinked into the working directory of the
task can be used to distribute native libraries and load them.

#### Tool

The Tool
interface supports the handling of generic Hadoop command-line options.

Tool is the standard for any Map/Reduce tool or
application. The application should delegate the handling of
standard command-line options to
GenericOptionsParser via
ToolRunner.run(Tool, String[]) and only handle its custom
arguments.

The generic Hadoop command-line options are:  

-conf <configuration file>
  

-D <property=value>
  

-fs <local|namenode:port>
  

-jt <local|jobtracker:port>

#### IsolationRunner

IsolationRunner is a utility to help debug Map/Reduce programs.

To use the IsolationRunner, first set
keep.failed.tasks.files to true
(also see keep.tasks.files.pattern).

Next, go to the node on which the failed task ran and go to the
TaskTracker's local directory and run the
IsolationRunner:  
$ cd <local path>/taskTracker/${taskid}/work
  

$ bin/hadoop org.apache.hadoop.mapred.IsolationRunner ../job.xml

IsolationRunner will run the failed task in a single
jvm, which can be in the debugger, over precisely the same input.

#### Profiling

Profiling is a utility to get a representative (2 or 3) sample
of built-in java profiler for a sample of maps and reduces.

User can specify whether the system should collect profiler
information for some of the tasks in the job by setting the
configuration property mapred.task.profile. The
value can be set using the api
JobConf.setProfileEnabled(boolean). If the value is set
true, the task profiling is enabled. The profiler
information is stored in the the user log directory. By default,
profiling is not enabled for the job.

Once user configures that profiling is needed, she/he can use
the configuration property
mapred.task.profile.{maps|reduces} to set the ranges
of map/reduce tasks to profile. The value can be set using the api
JobConf.setProfileTaskRange(boolean,String).
By default, the specified range is 0-2.

User can also specify the profiler configuration arguments by
setting the configuration property
mapred.task.profile.params. The value can be specified
using the api
JobConf.setProfileParams(String). If the string contains a
%s, it will be replaced with the name of the profiling
output file when the task runs. These parameters are passed to the
task child JVM on the command line. The default value for
the profiling parameters is
-agentlib:hprof=cpu=samples,heap=sites,force=n,thread=y,verbose=n,file=%s

#### Debugging

Map/Reduce framework provides a facility to run user-provided
scripts for debugging. When map/reduce task fails, user can run
script for doing post-processing on task logs i.e task's stdout,
stderr, syslog and jobconf. The stdout and stderr of the
user-provided debug script are printed on the diagnostics.
These outputs are also displayed on job UI on demand.

In the following sections we discuss how to submit debug script
along with the job. For submitting debug script, first it has to
distributed. Then the script has to supplied in Configuration.

##### How to distribute script file:

The user has to use
DistributedCache
mechanism to *distribute* and *symlink* the
debug script file.

##### How to submit script:

A quick way to submit debug script is to set values for the
properties "mapred.map.task.debug.script" and
"mapred.reduce.task.debug.script" for debugging map task and reduce
task respectively. These properties can also be set by using APIs
JobConf.setMapDebugScript(String)  and
JobConf.setReduceDebugScript(String) . For streaming, debug
script can be submitted with command-line options -mapdebug,
-reducedebug for debugging mapper and reducer respectively.

The arguments of the script are task's stdout, stderr,
syslog and jobconf files. The debug command, run on the node where
the map/reduce failed, is:   
 $script $stdout $stderr $syslog $jobconf

Pipes programs have the c++ program name as a fifth argument
for the command. Thus for the pipes programs the command is   
$script $stdout $stderr $syslog $jobconf $program

##### Default Behavior:

For pipes, a default script is run to process core dumps under
gdb, prints stack trace and gives info about running threads.

#### JobControl

JobControl is a utility which encapsulates a set of Map/Reduce jobs
and their dependencies.

#### Data Compression

Hadoop Map/Reduce provides facilities for the application-writer to
specify compression for both intermediate map-outputs and the
job-outputs i.e. output of the reduces. It also comes bundled with
CompressionCodec implementations for the
zlib and lzo compression
algorithms. The gzip file format is also
supported.

Hadoop also provides native implementations of the above compression
codecs for reasons of both performance (zlib) and non-availability of
Java libraries (lzo). More details on their usage and availability are
available here.

##### Intermediate Outputs

Applications can control compression of intermediate map-outputs
via the
JobConf.setCompressMapOutput(boolean) api and the
CompressionCodec to be used via the
JobConf.setMapOutputCompressorClass(Class) api.

##### Job Outputs

Applications can control compression of job-outputs via the
FileOutputFormat.setCompressOutput(JobConf, boolean) api and the
CompressionCodec to be used can be specified via the
FileOutputFormat.setOutputCompressorClass(JobConf, Class) api.

If the job outputs are to be stored in the
SequenceFileOutputFormat, the required
SequenceFile.CompressionType (i.e. RECORD /
BLOCK - defaults to RECORD) can be
specified via the
SequenceFileOutputFormat.setOutputCompressionType(JobConf,
SequenceFile.CompressionType) api.

## Example: WordCount v2.0

Here is a more complete WordCount which uses many of the
features provided by the Map/Reduce framework we discussed so far.

This needs the HDFS to be up and running, especially for the
DistributedCache-related features. Hence it only works with a
pseudo-distributed or
fully-distributed
Hadoop installation.

### Source Code

|  | WordCount.java |
| --- | --- |
| 1. | package org.myorg; |
| 2. |  |
| 3. | import java.io.\*; |
| 4. | import java.util.\*; |
| 5. |  |
| 6. | import org.apache.hadoop.fs.Path; |
| 7. | import org.apache.hadoop.filecache.DistributedCache; |
| 8. | import org.apache.hadoop.conf.\*; |
| 9. | import org.apache.hadoop.io.\*; |
| 10. | import org.apache.hadoop.mapred.\*; |
| 11. | import org.apache.hadoop.util.\*; |
| 12. |  |
| 13. | public class WordCount extends Configured implements Tool { |
| 14. |  |
| 15. | public static class Map extends MapReduceBase implements Mapper<LongWritable, Text, Text, IntWritable> { |
| 16. |  |
| 17. | static enum Counters { INPUT\_WORDS } |
| 18. |  |
| 19. | private final static IntWritable one = new IntWritable(1); |
| 20. | private Text word = new Text(); |
| 21. |  |
| 22. | private boolean caseSensitive = true; |
| 23. | private Set<String> patternsToSkip = new HashSet<String>(); |
| 24. |  |
| 25. | private long numRecords = 0; |
| 26. | private String inputFile; |
| 27. |  |
| 28. | public void configure(JobConf job) { |
| 29. | caseSensitive = job.getBoolean("wordcount.case.sensitive", true); |
| 30. | inputFile = job.get("map.input.file"); |
| 31. |  |
| 32. | if (job.getBoolean("wordcount.skip.patterns", false)) { |
| 33. | Path[] patternsFiles = new Path[0]; |
| 34. | try { |
| 35. | patternsFiles = DistributedCache.getLocalCacheFiles(job); |
| 36. | } catch (IOException ioe) { |
| 37. | System.err.println("Caught exception while getting cached files: " + StringUtils.stringifyException(ioe)); |
| 38. | } |
| 39. | for (Path patternsFile : patternsFiles) { |
| 40. | parseSkipFile(patternsFile); |
| 41. | } |
| 42. | } |
| 43. | } |
| 44. |  |
| 45. | private void parseSkipFile(Path patternsFile) { |
| 46. | try { |
| 47. | BufferedReader fis = new BufferedReader(new FileReader(patternsFile.toString())); |
| 48. | String pattern = null; |
| 49. | while ((pattern = fis.readLine()) != null) { |
| 50. | patternsToSkip.add(pattern); |
| 51. | } |
| 52. | } catch (IOException ioe) { |
| 53. | System.err.println("Caught exception while parsing the cached file '" + patternsFile + "' : " + StringUtils.stringifyException(ioe)); |
| 54. | } |
| 55. | } |
| 56. |  |
| 57. | public void map(LongWritable key, Text value, OutputCollector<Text, IntWritable> output, Reporter reporter) throws IOException { |
| 58. | String line = (caseSensitive) ? value.toString() : value.toString().toLowerCase(); |
| 59. |  |
| 60. | for (String pattern : patternsToSkip) { |
| 61. | line = line.replaceAll(pattern, ""); |
| 62. | } |
| 63. |  |
| 64. | StringTokenizer tokenizer = new StringTokenizer(line); |
| 65. | while (tokenizer.hasMoreTokens()) { |
| 66. | word.set(tokenizer.nextToken()); |
| 67. | output.collect(word, one); |
| 68. | reporter.incrCounter(Counters.INPUT\_WORDS, 1); |
| 69. | } |
| 70. |  |
| 71. | if ((++numRecords % 100) == 0) { |
| 72. | reporter.setStatus("Finished processing " + numRecords + " records " + "from the input file: " + inputFile); |
| 73. | } |
| 74. | } |
| 75. | } |
| 76. |  |
| 77. | public static class Reduce extends MapReduceBase implements Reducer<Text, IntWritable, Text, IntWritable> { |
| 78. | public void reduce(Text key, Iterator<IntWritable> values, OutputCollector<Text, IntWritable> output, Reporter reporter) throws IOException { |
| 79. | int sum = 0; |
| 80. | while (values.hasNext()) { |
| 81. | sum += values.next().get(); |
| 82. | } |
| 83. | output.collect(key, new IntWritable(sum)); |
| 84. | } |
| 85. | } |
| 86. |  |
| 87. | public int run(String[] args) throws Exception { |
| 88. | JobConf conf = new JobConf(getConf(), WordCount.class); |
| 89. | conf.setJobName("wordcount"); |
| 90. |  |
| 91. | conf.setOutputKeyClass(Text.class); |
| 92. | conf.setOutputValueClass(IntWritable.class); |
| 93. |  |
| 94. | conf.setMapperClass(Map.class); |
| 95. | conf.setCombinerClass(Reduce.class); |
| 96. | conf.setReducerClass(Reduce.class); |
| 97. |  |
| 98. | conf.setInputFormat(TextInputFormat.class); |
| 99. | conf.setOutputFormat(TextOutputFormat.class); |
| 100. |  |
| 101. | List<String> other\_args = new ArrayList<String>(); |
| 102. | for (int i=0; i < args.length; ++i) { |
| 103. | if ("-skip".equals(args[i])) { |
| 104. | DistributedCache.addCacheFile(new Path(args[++i]).toUri(), conf); |
| 105. | conf.setBoolean("wordcount.skip.patterns", true); |
| 106. | } else { |
| 107. | other\_args.add(args[i]); |
| 108. | } |
| 109. | } |
| 110. |  |
| 111. | FileInputFormat.setInputPaths(conf, new Path(other\_args.get(0))); |
| 112. | FileOutputFormat.setOutputPath(conf, new Path(other\_args.get(1))); |
| 113. |  |
| 114. | JobClient.runJob(conf); |
| 115. | return 0; |
| 116. | } |
| 117. |  |
| 118. | public static void main(String[] args) throws Exception { |
| 119. | int res = ToolRunner.run(new Configuration(), new WordCount(), args); |
| 120. | System.exit(res); |
| 121. | } |
| 122. | } |
| 123. |  |

### Sample Runs

Sample text-files as input:

$ bin/hadoop dfs -ls /usr/joe/wordcount/input/
  
/usr/joe/wordcount/input/file01
  
/usr/joe/wordcount/input/file02
  
  
$ bin/hadoop dfs -cat /usr/joe/wordcount/input/file01
  
Hello World, Bye World!
  
  
$ bin/hadoop dfs -cat /usr/joe/wordcount/input/file02
  
Hello Hadoop, Goodbye to hadoop.

Run the application:

$ bin/hadoop jar /usr/joe/wordcount.jar org.myorg.WordCount
/usr/joe/wordcount/input /usr/joe/wordcount/output

Output:

$ bin/hadoop dfs -cat /usr/joe/wordcount/output/part-00000
  
Bye 1
  
Goodbye 1
  
Hadoop, 1
  
Hello 2
  
World! 1
  
World, 1
  
hadoop. 1
  
to 1

Notice that the inputs differ from the first version we looked at,
and how they affect the outputs.

Now, lets plug-in a pattern-file which lists the word-patterns to be
ignored, via the DistributedCache.

$ hadoop dfs -cat /user/joe/wordcount/patterns.txt
  
\.
  
\,
  
\!
  
to

Run it again, this time with more options:

$ bin/hadoop jar /usr/joe/wordcount.jar org.myorg.WordCount
-Dwordcount.case.sensitive=true /usr/joe/wordcount/input
/usr/joe/wordcount/output -skip /user/joe/wordcount/patterns.txt

As expected, the output:

$ bin/hadoop dfs -cat /usr/joe/wordcount/output/part-00000
  
Bye 1
  
Goodbye 1
  
Hadoop 1
  
Hello 2
  
World 2
  
hadoop 1

Run it once more, this time switch-off case-sensitivity:

$ bin/hadoop jar /usr/joe/wordcount.jar org.myorg.WordCount
-Dwordcount.case.sensitive=false /usr/joe/wordcount/input
/usr/joe/wordcount/output -skip /user/joe/wordcount/patterns.txt

Sure enough, the output:

$ bin/hadoop dfs -cat /usr/joe/wordcount/output/part-00000
  
bye 1
  
goodbye 1
  
hadoop 2
  
hello 2
  
world 2

### Highlights

The second version of WordCount improves upon the
previous one by using some features offered by the Map/Reduce framework:

- Demonstrates how applications can access configuration parameters
  in the configure method of the Mapper (and
  Reducer) implementations (lines 28-43).
- Demonstrates how the DistributedCache can be used to
  distribute read-only data needed by the jobs. Here it allows the user
  to specify word-patterns to skip while counting (line 104).
- Demonstrates the utility of the Tool interface and the
  GenericOptionsParser to handle generic Hadoop
  command-line options (lines 87-116, 119).
- Demonstrates how applications can use Counters (line 68)
  and how they can set application-specific status information via
  the Reporter instance passed to the map (and
  reduce) method (line 72).

*Java and JNI are trademarks or registered trademarks of
Sun Microsystems, Inc. in the United States and other countries.*

Copyright ©
2007 The Apache Software Foundation.
