# Supplementary material for: Provenance based data integrity checking and verification in cloud environments
Source: PLoS One. 2017 May 17;12(5):e0177576. doi: 10.1371/journal.pone.0177576 (PMC5435237; doi:10.1371/journal.pone.0177576)
Supplement: S1 Dataset — Files in the Dataset are utilized for performance measurement. (ZIP) [file pone.0177576.s001.zip › dataset/html/native_libraries - Copy (2) - Copy.html]

Native Hadoop Libraries


Apache > Hadoop > Core

- Project
- Wiki
- Hadoop 0.18 Documentation

Documentation

Overview

Quickstart

Cluster Setup

HDFS Architecture

HDFS User Guide

HDFS Permissions Guide

HDFS Quotas Administrator Guide

Commands Manual

FS Shell Guide

DistCp Guide

Map-Reduce Tutorial

Native Hadoop Libraries

Streaming

Hadoop Archives

Hadoop On Demand

API Docs

Wiki

FAQ

Mailing Lists

Release Notes

All Changes


PDF

# Native Hadoop Libraries

- Purpose
- Components
- Usage
- Supported Platforms
- Building Native Hadoop Libraries
  - Notes
- Loading native libraries through DistributedCache

## Purpose

Hadoop has native implementations of certain components for reasons of
both performance and non-availability of Java implementations. These
components are available in a single, dynamically-linked, native library.
On the \*nix platform it is *libhadoop.so*. This document describes
the usage and details on how to build the native libraries.

## Components

Hadoop currently has the following
compression codecs as the native components:

- zlib
- gzip
- lzo

Of the above, the availability of native hadoop libraries is imperative
for the lzo and gzip compression codecs to work.

## Usage

It is fairly simple to use the native hadoop libraries:

- Take a look at the
  supported platforms.
- Either download the pre-built
  32-bit i386-Linux native hadoop libraries (available as part of hadoop
  distribution in lib/native directory) or
  build them yourself.
- Make sure you have either or both of **>zlib-1.2** and
  **>lzo2.0** packages for your platform installed;
  depending on your needs.

The bin/hadoop script ensures that the native hadoop
library is on the library path via the system property
*-Djava.library.path=<path>*.

To check everything went alright check the hadoop log files for:

DEBUG util.NativeCodeLoader - Trying to load the custom-built
native-hadoop library...
  

INFO util.NativeCodeLoader - Loaded the native-hadoop library

If something goes wrong, then:

INFO util.NativeCodeLoader - Unable to load native-hadoop library for
your platform... using builtin-java classes where applicable

## Supported Platforms

Hadoop native library is supported only on \*nix platforms only.
Unfortunately it is known not to work on Cygwin
and Mac OS X and has mainly been used on the
GNU/Linux platform.

It has been tested on the following GNU/Linux distributions:

- RHEL4/Fedora
- Ubuntu
- Gentoo

On all the above platforms a 32/64 bit Hadoop native library will work
with a respective 32/64 bit jvm.

## Building Native Hadoop Libraries

Hadoop native library is written in
ANSI C and built using
the GNU autotools-chain (autoconf, autoheader, automake, autoscan, libtool).
This means it should be straight-forward to build them on any platform with
a standards compliant C compiler and the GNU autotools-chain.
See supported platforms.

In particular the various packages you would need on the target
platform are:

- C compiler (e.g. GNU C Compiler)
- GNU Autools Chain:
  autoconf,
  automake,
  libtool
- zlib-development package (stable version >= 1.2.0)
- lzo-development package (stable version >= 2.0)

Once you have the pre-requisites use the standard build.xml
and pass along the compile.native flag (set to
true) to build the native hadoop library:

$ ant -Dcompile.native=true <target>

The native hadoop library is not built by default since not everyone is
interested in building them.

You should see the newly-built native hadoop library in:

$ build/native/<platform>/lib

where <platform> is combination of the system-properties:
${os.name}-${os.arch}-${sun.arch.data.model}; for e.g.
Linux-i386-32.

### Notes

- It is **mandatory** to have both the zlib and lzo
  development packages on the target platform for building the
  native hadoop library; however for deployment it is sufficient to
  install zlib or lzo if you wish to use only one of them.
- It is necessary to have the correct 32/64 libraries of both zlib/lzo
  depending on the 32/64 bit jvm for the target platform for
  building/deployment of the native hadoop library.

## Loading native libraries through DistributedCache

User can load native shared libraries through
DistributedCache
for *distributing* and *symlinking* the library files

Here is an example, describing how to distribute the library and
load it from map/reduce task.

1. First copy the library to the HDFS.   
   bin/hadoop fs -copyFromLocal mylib.so.1 /libraries/mylib.so.1
2. The job launching program should contain the following:   
    DistributedCache.createSymlink(conf); 
     
    DistributedCache.addCacheFile("hdfs://host:port/libraries/mylib.so.1#mylib.so", conf);
3. The map/reduce task can contain:   
    System.loadLibrary("mylib.so");

Copyright ©
2007 The Apache Software Foundation.
