# Supplementary material for: Provenance based data integrity checking and verification in cloud environments
Source: PLoS One. 2017 May 17;12(5):e0177576. doi: 10.1371/journal.pone.0177576 (PMC5435237; doi:10.1371/journal.pone.0177576)
Supplement: S1 Dataset — Files in the Dataset are utilized for performance measurement. (ZIP) [file pone.0177576.s001.zip › dataset/html/package-frame - Copy (2).html]

org.apache.hadoop (Hadoop 0.18.0 API)


org.apache.hadoop

Annotation Types 
  
HadoopVersionAnnotation
