# Supplementary material for: Provenance based data integrity checking and verification in cloud environments
Source: PLoS One. 2017 May 17;12(5):e0177576. doi: 10.1371/journal.pone.0177576 (PMC5435237; doi:10.1371/journal.pone.0177576)
Supplement: S1 Dataset — Files in the Dataset are utilized for performance measurement. (ZIP) [file pone.0177576.s001.zip › dataset/html/quickstart - Copy (2) - Copy.html]

Hadoop Quickstart


Apache > Hadoop > Core

- Project
- Wiki
- Hadoop 0.18 Documentation

Documentation

Overview

Quickstart

Cluster Setup

HDFS Architecture

HDFS User Guide

HDFS Permissions Guide

HDFS Quotas Administrator Guide

Commands Manual

FS Shell Guide

DistCp Guide

Map-Reduce Tutorial

Native Hadoop Libraries

Streaming

Hadoop Archives

Hadoop On Demand

API Docs

Wiki

FAQ

Mailing Lists

Release Notes

All Changes


PDF

# Hadoop Quickstart

- Purpose
- Pre-requisites
  - Supported Platforms
  - Required Software
  - Installing Software
- Download
- Prepare to Start the Hadoop Cluster
- Standalone Operation
- Pseudo-Distributed Operation
  - Configuration
  - Setup passphraseless ssh
  - Execution
- Fully-Distributed Operation

## Purpose

The purpose of this document is to help you get a single-node Hadoop
installation up and running very quickly so that you can get a flavour
of the Hadoop Distributed File System
(HDFS) and
the Map/Reduce framework; that is, perform simple operations on HDFS and
run example jobs.

## Pre-requisites

### Supported Platforms

- GNU/Linux is supported as a development and production platform.
  Hadoop has been demonstrated on GNU/Linux clusters with 2000 nodes.
- Win32 is supported as a *development platform*. Distributed
  operation has not been well tested on Win32, so it is not
  supported as a *production platform*.

### Required Software

Required software for Linux and Windows include:

1. Java^TM^ 1.5.x, preferably from Sun, must be installed.
2. **ssh** must be installed and **sshd** must
   be running to use the Hadoop scripts that manage remote Hadoop
   daemons.

Additional requirements for Windows include:

1. Cygwin - Required for shell
   support in addition to the required software above.

### Installing Software

If your cluster doesn't have the requisite software you will need to
install it.

For example on Ubuntu Linux:

$ sudo apt-get install ssh
  
$ sudo apt-get install rsync

On Windows, if you did not install the required software when you
installed cygwin, start the cygwin installer and select the packages:

- openssh - the *Net* category

## Download

To get a Hadoop distribution, download a recent
stable release from one of the Apache Download
Mirrors.

## Prepare to Start the Hadoop Cluster

Unpack the downloaded Hadoop distribution. In the distribution, edit the
file conf/hadoop-env.sh to define at least
JAVA\_HOME to be the root of your Java installation.

Try the following command:  
$ bin/hadoop
  
This will display the usage documentation for the **hadoop**
script.

Now you are ready to start your Hadoop cluster in one of the three supported
modes:

- Local (Standalone) Mode
- Pseudo-Distributed Mode
- Fully-Distributed Mode

## Standalone Operation

By default, Hadoop is configured to run in a non-distributed
mode, as a single Java process. This is useful for debugging.

The following example copies the unpacked conf directory to
use as input and then finds and displays every match of the given regular
expression. Output is written to the given output directory.
  
$ mkdir input
  
$ cp conf/\*.xml input
  

$ bin/hadoop jar hadoop-\*-examples.jar grep input output 'dfs[a-z.]+'
  
$ cat output/\*

## Pseudo-Distributed Operation

Hadoop can also be run on a single-node in a pseudo-distributed mode
where each Hadoop daemon runs in a separate Java process.

### Configuration

Use the following conf/hadoop-site.xml:

<configuration>

<property>

<name>fs.default.name</name>

<value>localhost:9000</value>

</property>

<property>

<name>mapred.job.tracker</name>

<value>localhost:9001</value>

</property>

<property>

<name>dfs.replication</name>

<value>1</value>

</property>

</configuration>

### Setup passphraseless ssh

Now check that you can ssh to the localhost without a passphrase:  
$ ssh localhost

If you cannot ssh to localhost without a passphrase, execute the
following commands:  
$ ssh-keygen -t dsa -P '' -f ~/.ssh/id\_dsa
  
$ cat ~/.ssh/id\_dsa.pub >> ~/.ssh/authorized\_keys

### Execution

Format a new distributed-filesystem:  
$ bin/hadoop namenode -format

Start the hadoop daemons:  
$ bin/start-all.sh

The hadoop daemon log output is written to the
${HADOOP\_LOG\_DIR} directory (defaults to
${HADOOP\_HOME}/logs).

Browse the web interface for the NameNode and the JobTracker; by
default they are available at:

- NameNode -
  http://localhost:50070/
- JobTracker -
  http://localhost:50030/

Copy the input files into the distributed filesystem:  
$ bin/hadoop fs -put conf input

Run some of the examples provided:  

$ bin/hadoop jar hadoop-\*-examples.jar grep input output 'dfs[a-z.]+'

Examine the output files:

Copy the output files from the distributed filesystem to the local
filesytem and examine them:  
$ bin/hadoop fs -get output output
  
$ cat output/\*

or

View the output files on the distributed filesystem:  
$ bin/hadoop fs -cat output/\*

When you're done, stop the daemons with:  
$ bin/stop-all.sh

## Fully-Distributed Operation

Information on setting up fully-distributed, non-trivial clusters
can be found here.

*Java and JNI are trademarks or registered trademarks of
Sun Microsystems, Inc. in the United States and other countries.*

Copyright ©
2007 The Apache Software Foundation.
