# Supplementary material for: Provenance based data integrity checking and verification in cloud environments
Source: PLoS One. 2017 May 17;12(5):e0177576. doi: 10.1371/journal.pone.0177576 (PMC5435237; doi:10.1371/journal.pone.0177576)
Supplement: S1 Dataset — Files in the Dataset are utilized for performance measurement. (ZIP) [file pone.0177576.s001.zip › dataset/html/releasenotes - Copy (2) - Copy.html]

Hadoop 0.18.0 Release Notes


# Hadoop 0.18.0 Release Notes

These release notes include new developer and user facing incompatibilities, features, and major improvements.
The table below is sorted by Component.


## Changes Since Hadoop 0.17.2

|  |  |  |
| --- | --- | --- |
| **Issue** | **Component** | **Notes** |
| HADOOP-3355 | conf | Added support for hexadecimal values in Configuration |
| HADOOP-1702 | dfs | Reduced buffer copies as data is written to HDFS. The order of sending data bytes and control information has changed, but this will not be observed by client applications. |
| HADOOP-2065 | dfs | Added "corrupt" flag to LocatedBlock to indicate that all replicas of the block thought to be corrupt. |
| HADOOP-2585 | dfs | Improved management of replicas of the name space image. If all replicas on the Name Node are lost, the latest check point can be loaded from the secondary Name Node. Use parameter "-importCheckpoint" and specify the location with "fs.checkpoint.dir." The directory structure on the secondary Name Node has changed to match the primary Name Node. |
| HADOOP-2656 | dfs | Associated a generation stamp with each block. On data nodes, the generation stamp is stored as part of the file name of the block's meta-data file. |
| HADOOP-2703 | dfs | Changed fsck to ignore files opened for writing. Introduced new option "-openforwrite" to explicitly show open files. |
| HADOOP-2797 | dfs | Withdrew the upgrade-to-CRC facility. HDFS will no longer support upgrades from versions without CRCs for block data. Users upgrading from version 0.13 or earlier must first upgrade to an intermediate (0.14, 0.15, 0.16, 0.17) version before doing upgrade to version 0.18 or later. |
| HADOOP-2865 | dfs | Changed the output of the "fs -ls" command to more closely match familiar Linux format. Additional changes were made by HADOOP-3459. Applications that parse the command output should be reviewed. |
| HADOOP-3035 | dfs | Changed protocol for transferring blocks between data nodes to report corrupt blocks to data node for re-replication from a good replica. |
| HADOOP-3113 | dfs | Added sync() method to FSDataOutputStream to really, really persist data in HDFS. InterDatanodeProtocol to implement this feature. |
| HADOOP-3164 | dfs | Changed data node to use FileChannel.tranferTo() to transfer block data. |
| HADOOP-3177 | dfs | Added a new public interface Syncable which declares the sync() operation. FSDataOutputStream implements Syncable. If the wrappedStream in FSDataOutputStream is Syncalbe, calling FSDataOutputStream.sync() is equivalent to call wrappedStream.sync(). Otherwise, FSDataOutputStream.sync() is a no-op. Both DistributedFileSystem and LocalFileSystem support the sync() operation. |
| HADOOP-3187 | dfs | Introduced directory quota as hard limits on the number of names in the tree rooted at that directory. An administrator may set quotas on individual directories explicitly. Newly created directories have no associated quota. File/directory creations fault if the quota would be exceeded. The attempt to set a quota faults if the directory would be in violation of the new quota. |
| HADOOP-3193 | dfs | Added reporter to FSNamesystem stateChangeLog, and a new metric to track the number of corrupted replicas. |
| HADOOP-3232 | dfs | Changed 'du' command to run in a seperate thread so that it does not block user. |
| HADOOP-3310 | dfs | Implemented Lease Recovery to sync the last bock of a file. Added ClientDatanodeProtocol for client trigging block recovery. Changed DatanodeProtocol to support block synchronization. Changed InterDatanodeProtocol to support block update. |
| HADOOP-3317 | dfs | Changed the default port for "hdfs:" URIs to be 8020, so that one may simply use URIs of the form "hdfs://example.com/dir/file". |
| HADOOP-3329 | dfs | Changed format of file system image to not store locations of last block. |
| HADOOP-3336 | dfs | Added a log4j appender that emits events from FSNamesystem for audit logging |
| HADOOP-3339 | dfs | Improved failure handling of last Data Node in write pipeline. |
| HADOOP-3390 | dfs | Removed deprecated ClientProtocol.abandonFileInProgress(). |
| HADOOP-3452 | dfs | Changed exit status of fsck to report whether the files system is healthy or corrupt. |
| HADOOP-3459 | dfs | Changed the output of the "fs -ls" command to more closely match familiar Linux format. Applications that parse the command output should be reviewed. |
| HADOOP-3486 | dfs | Changed the default value of dfs.blockreport.initialDelay to be 0 seconds. |
| HADOOP-3677 | dfs | Simplify generation stamp upgrade by making is a local upgrade on datandodes. Deleted distributed upgrade. |
| HADOOP-2188 | dfs   ipc | Replaced timeouts with pings to check that client connection is alive. Removed the property ipc.client.timeout from the default Hadoop configuration. Removed the metric RpcOpsDiscardedOPsNum. |
| HADOOP-3283 | dfs   ipc | Added an IPC server in DataNode and a new IPC protocol InterDatanodeProtocol. Added conf properties dfs.datanode.ipc.address and dfs.datanode.handler.count with defaults "0.0.0.0:50020" and 3, respectively.   Changed the serialization in DatanodeRegistration and DatanodeInfo, and therefore, updated the versionID in ClientProtocol, DatanodeProtocol, NamenodeProtocol. |
| HADOOP-3058 | dfs   metrics | Added FSNamesystem status metrics. |
| HADOOP-3683 | dfs   metrics | Change FileListed to getNumGetListingOps and add CreateFileOps, DeleteFileOps and AddBlockOps metrics. |
| HADOOP-3265 | fs | Removed deprecated API getFileCacheHints |
| HADOOP-3307 | fs | Introduced archive feature to Hadoop. A Map/Reduce job can be run to create an archive with indexes. A FileSystem abstraction is provided over the archive. |
| HADOOP-930 | fs | Added support for reading and writing native S3 files. Native S3 files are referenced using s3n URIs. See http://wiki.apache.org/hadoop/AmazonS3 for more details. |
| HADOOP-3095 | fs   fs/s3 | Added overloaded method getFileBlockLocations(FileStatus, long, long). This is an incompatible change for FileSystem implementations which override getFileBlockLocations(Path, long, long). They should have the signature of this method changed to getFileBlockLocations(FileStatus, long, long) to work correctly. |
| HADOOP-4 | fuse-dfs | Introduced FUSE module for HDFS. Module allows mount of HDFS as a Unix filesystem, and optionally the export of that mount point to other machines. Writes are disabled. rmdir, mv, mkdir, rm are supported, but not cp, touch, and the like. Usage information is attached to the Jira record. |
| HADOOP-3184 | hod | Modified HOD to handle master (NameNode or JobTracker) failures on bad nodes by trying to bring them up on another node in the ring. Introduced new property ringmaster.max-master-failures to specify the maximum number of times a master is allowed to fail. |
| HADOOP-3266 | hod | Moved HOD change items from CHANGES.txt to a new file src/contrib/hod/CHANGES.txt. |
| HADOOP-3376 | hod | Modified HOD client to look for specific messages related to resource limit overruns and take appropriate actions - such as either failing to allocate the cluster, or issuing a warning to the user. A tool is provided, specific to Maui and Torque, that will set these specific messages. |
| HADOOP-3464 | hod | Implemented a mechanism to transfer HOD errors that occur on compute nodes to the submit node running the HOD client, so users have good feedback on why an allocation failed. |
| HADOOP-3483 | hod | Modified HOD to create a cluster directory if one does not exist and to auto-deallocate a cluster while reallocating it, if it is already dead. |
| HADOOP-3564 | hod | Modifed HOD to generate the dfs.datanode.ipc.address parameter in the hadoop-site.xml of datanodes that it launches. |
| HADOOP-3610 | hod | Modified HOD to automatically create a cluster directory if the one specified with the script command does not exist. |
| HADOOP-3703 | hod | Modified logcondense.py to use the new format of hadoop dfs -lsr output. This version of logcondense would not work with previous versions of Hadoop and hence is incompatible. |
| HADOOP-3061 | io | Introduced ByteWritable and DoubleWritable (implementing WritableComparable) implementations for Byte and Double. |
| HADOOP-3299 | io   mapred | Changed the TextInputFormat and KeyValueTextInput classes to initialize the compressionCodecs member variable before dereferencing it. |
| HADOOP-2909 | ipc | Removed property ipc.client.maxidletime from the default configuration. The allowed idle time is twice ipc.client.connection.maxidletime. |
| HADOOP-3569 | KFS | Fixed KFS to have read() read and return 1 byte instead of 4. |
| HADOOP-1915 | mapred | Provided a new method to update counters. "incrCounter(String group, String counter, long amount)" |
| HADOOP-2019 | mapred | Added support for .tar, .tgz and .tar.gz files in DistributedCache. File sizes are limited to 2GB. |
| HADOOP-2095 | mapred | Reduced in-memory copies of keys and values as they flow through the Map-Reduce framework. Changed the storage of intermediate map outputs to use new IFile instead of SequenceFile for better compression. |
| HADOOP-2132 | mapred | Changed "job -kill" to only allow a job that is in the RUNNING or PREP state to be killed. |
| HADOOP-2181 | mapred | Added logging for input splits in job tracker log and job history log. Added web UI for viewing input splits in the job UI and history UI. |
| HADOOP-236 | mapred | Changed connection protocol job tracker and task tracker so that task tracker will not connect to a job tracker with a different build version. |
| HADOOP-2427 | mapred | The current working directory of a task, i.e. ${mapred.local.dir}/taskTracker/jobcache/<jobid>/<task\_dir>/work is cleanedup, as soon as the task is finished. |
| HADOOP-2867 | mapred | Added task's cwd to its LD\_LIBRARY\_PATH. |
| HADOOP-3135 | mapred | Changed job submission protocol to not allow submission if the client's value of mapred.system.dir does not match the job tracker's. Deprecated JobConf.getSystemDir(); use JobClient.getSystemDir(). |
| HADOOP-3221 | mapred | Added org.apache.hadoop.mapred.lib.NLineInputFormat, which splits N lines of input as one split. N can be specified by configuration property "mapred.line.input.format.linespermap", which defaults to 1. |
| HADOOP-3226 | mapred | Changed policy for running combiner. The combiner may be run multiple times as the map's output is sorted and merged. Additionally, it may be run on the reduce side as data is merged. The old semantics are available in Hadoop 0.18 if the user calls:   job.setCombineOnlyOnce(true); |
| HADOOP-3326 | mapred | Changed fetchOutputs() so that LocalFSMerger and InMemFSMergeThread threads are spawned only once. The thread gets notified when something is ready for merge. The merge happens when thresholds are met. |
| HADOOP-3366 | mapred | Improved shuffle so that all fetched map-outputs are kept in-memory before being merged by stalling the shuffle so that the in-memory merge executes and frees up memory for the shuffle. |
| HADOOP-3405 | mapred | Refactored previously public classes MapTaskStatus, ReduceTaskStatus, JobSubmissionProtocol, CompletedJobStatusStore to be package local. |
| HADOOP-3417 | mapred | Removed the public class org.apache.hadoop.mapred.JobShell.   Command line options -libjars, -files and -archives are moved to GenericCommands. Thus applications have to implement org.apache.hadoop.util.Tool to use the options. |
| HADOOP-3427 | mapred | Changed shuffle scheduler policy to wait for notifications from shuffle threads before scheduling more. |
| HADOOP-3460 | mapred | Created SequenceFileAsBinaryOutputFormat to write raw bytes as keys and values to a SequenceFile. |
| HADOOP-3512 | mapred | Separated Distcp, Logalyzer and Archiver into a tools jar. |
| HADOOP-3565 | mapred | Change the Java serialization framework, which is not enabled by default, to correctly make the objects independent of the previous objects. |
| HADOOP-3598 | mapred | Changed Map-Reduce framework to no longer create temporary task output directories for staging outputs if staging outputs isn't necessary. ${mapred.out.dir}/\_temporary/\_${taskid} |
| HADOOP-544 | mapred | Introduced new classes JobID, TaskID and TaskAttemptID, which should be used instead of their string counterparts. Deprecated functions in JobClient, TaskReport, RunningJob, jobcontrol.Job and TaskCompletionEvent that use string arguments. Applications can use xxxID.toString() and xxxID.forName() methods to convert/restore objects to/from strings. |
| HADOOP-3230 | scripts | Added command line tool "job -counter <job-id> <group-name> <counter-name>" to access counters. |
| HADOOP-1328 | streaming | Introduced a way for a streaming process to update global counters and status using stderr stream to emit information. Use "reporter:counter:<group>,<counter>,<amount> " to update a counter. Use "reporter:status:<message>" to update status. |
| HADOOP-3429 | streaming | Increased the size of the buffer used in the communication between the Java task and the Streaming process to 128KB. |
| HADOOP-3379 | streaming   documentation | Set default value for configuration property "stream.non.zero.exit.status.is.failure" to be "true". |
| HADOOP-3246 | util | Introduced an FTPFileSystem backed by Apache Commons FTPClient to directly store data into HDFS. |
