# Supplementary material for: Provenance based data integrity checking and verification in cloud environments
Source: PLoS One. 2017 May 17;12(5):e0177576. doi: 10.1371/journal.pone.0177576 (PMC5435237; doi:10.1371/journal.pone.0177576)
Supplement: S1 Dataset — Files in the Dataset are utilized for performance measurement. (ZIP) [file pone.0177576.s001.zip › dataset/html/streaming - Copy (2) - Copy.html]

Hadoop Streaming


Apache > Hadoop > Core

- Project
- Wiki
- Hadoop 0.18 Documentation

Documentation

Overview

Quickstart

Cluster Setup

HDFS Architecture

HDFS User Guide

HDFS Permissions Guide

HDFS Quotas Administrator Guide

Commands Manual

FS Shell Guide

DistCp Guide

Map-Reduce Tutorial

Native Hadoop Libraries

Streaming

Hadoop Archives

Hadoop On Demand

API Docs

Wiki

FAQ

Mailing Lists

Release Notes

All Changes


PDF

# Hadoop Streaming

- Hadoop Streaming
- How Does Streaming Work
- Package Files With Job Submissions
- Streaming Options and Usage 
  - Mapper-Only Jobs
  - Specifying Other Plugins for Jobs
  - Large files and archives in Hadoop Streaming
  - Specifying Additional Configuration Variables for Jobs
  - Other Supported Options
- More usage examples 
  - Customizing the Way to Split Lines into Key/Value Pairs
  - A Useful Partitioner Class (secondary sort, the -partitioner org.apache.hadoop.mapred.lib.KeyFieldBasedPartitioner option)
  - Working with the Hadoop Aggregate Package (the -reduce aggregate option)
  - Field Selection ( similar to unix 'cut' command)
- Frequently Asked Questions 
  - How do I use Hadoop Streaming to run an arbitrary set of (semi-)independent tasks?
  - How do I process files, one per map?
  - How many reducers should I use?
  - If I set up an alias in my shell script, will that work after -mapper, i.e. say I do: alias c1='cut -f1'. Will -mapper "c1" work?
  - Can I use UNIX pipes? For example, will -mapper "cut -f1 | sed s/foo/bar/g" work?
  - When I run a streaming job by distributing large executables (for example, 3.6G) through the -file option, I get a "No space left on device" error. What do I do?
  - How do I specify multiple input directories?
  - How do I generate output files with gzip format?
  - How do I provide my own input/output format with streaming?
  - How do I parse XML documents using streaming?
  - How do I update counters in streaming applications?
  - How do I update status in streaming applications?

## Hadoop Streaming

Hadoop streaming is a utility that comes with the Hadoop distribution. The utility allows you to create and run Map/Reduce jobs with any executable or script as the mapper and/or the reducer. For example:

```
$HADOOP_HOME/bin/hadoop  jar $HADOOP_HOME/hadoop-streaming.jar \
    -input myInputDirs \
    -output myOutputDir \
    -mapper /bin/cat \
    -reducer /bin/wc
```

## How Does Streaming Work

In the above example, both the mapper and the reducer are executables that read the input from stdin (line by line) and emit the output to stdout. The utility will create a Map/Reduce job, submit the job to an appropriate cluster, and monitor the progress of the job until it completes.

When an executable is specified for mappers, each mapper task will launch the executable as a separate process when the mapper is initialized. As the mapper task runs, it converts its inputs into lines and feed the lines to the stdin of the process. In the meantime, the mapper collects the line oriented outputs from the stdout of the process and converts each line into a key/value pair, which is collected as the output of the mapper. By default, the
*prefix of a line up to the first tab character* is the **key** and the the rest of the line (excluding the tab character) will be the **value**.
If there is no tab character in the line, then entire line is considered as key and the value is null. However, this can be customized, as discussed later.

When an executable is specified for reducers, each reducer task will launch the executable as a separate process then the reducer is initialized. As the reducer task runs, it converts its input key/values pairs into lines and feeds the lines to the stdin of the process. In the meantime, the reducer collects the line oriented outputs from the stdout of the process, converts each line into a key/value pair, which is collected as the output of the reducer. By default, the prefix of a line up to the first tab character is the key and the the rest of the line (excluding the tab character) is the value. However, this can be customized, as discussed later.

This is the basis for the communication protocol between the Map/Reduce framework and the streaming mapper/reducer.

You can supply a Java class as the mapper and/or the reducer. The above example is equivalent to:

```
$HADOOP_HOME/bin/hadoop  jar $HADOOP_HOME/hadoop-streaming.jar \
    -input myInputDirs \
    -output myOutputDir \
    -mapper org.apache.hadoop.mapred.lib.IdentityMapper \
    -reducer /bin/wc
```

User can specify stream.non.zero.exit.is.failure as
true or false to make a streaming task that exits
with a non-zero status to be Failure
or Success respectively. By default, streaming tasks exiting
with non-zero status are considered to be failed tasks.

## Package Files With Job Submissions

You can specify any executable as the mapper and/or the reducer. The executables do not need to pre-exist on the machines in the cluster; however, if they don't, you will need to use "-file" option to tell the framework to pack your executable files as a part of job submission. For example:

```
$HADOOP_HOME/bin/hadoop  jar $HADOOP_HOME/hadoop-streaming.jar \
    -input myInputDirs \
    -output myOutputDir \
    -mapper myPythonScript.py \
    -reducer /bin/wc \
    -file myPythonScript.py
```

The above example specifies a user defined Python executable as the mapper. The option "-file myPythonScript.py" causes the python executable shipped to the cluster machines as a part of job submission.

In addition to executable files, you can also package other auxiliary files (such as dictionaries, configuration files, etc) that may be used by the mapper and/or the reducer. For example:

```
$HADOOP_HOME/bin/hadoop  jar $HADOOP_HOME/hadoop-streaming.jar \
    -input myInputDirs \
    -output myOutputDir \
    -mapper myPythonScript.py \
    -reducer /bin/wc \
    -file myPythonScript.py \
    -file myDictionary.txt
```

## Streaming Options and Usage

### Mapper-Only Jobs

Often, you may want to process input data using a map function only. To do this, simply set mapred.reduce.tasks to zero. The Map/Reduce framework will not create any reducer tasks. Rather, the outputs of the mapper tasks will be the final output of the job.

To be backward compatible, Hadoop Streaming also supports the "-reduce NONE" option, which is equivalent to "-jobconf mapred.reduce.tasks=0".

### Specifying Other Plugins for Jobs

Just as with a normal Map/Reduce job, you can specify other plugins for a streaming job:

```
   -inputformat JavaClassName
   -outputformat JavaClassName
   -partitioner JavaClassName
   -combiner JavaClassName
```

The class you supply for the input format should return key/value pairs of Text class. If you do not specify an input format class, the TextInputFormat is used as the default. Since the TextInputFormat returns keys of LongWritable class, which are actually not part of the input data, the keys will be discarded; only the values will be piped to the streaming mapper.

The class you supply for the output format is expected to take key/value pairs of Text class. If you do not specify an output format class, the TextOutputFormat is used as the default.

### Large files and archives in Hadoop Streaming

The -cacheFile and -cacheArchive options allow you to make files and archives available to the tasks. The argument is a URI to the file or archive that you have already uploaded to HDFS. These files and archives are cached across jobs. You can retrieve the host and fs\_port values from the fs.default.name config variable.

Here are examples of the -cacheFile option:

```
-cacheFile hdfs://host:fs_port/user/testfile.txt#testlink
```

In the above example, the part of the url after # is used as the symlink name that is created in the current working directory of tasks. So the tasks will have a symlink called testlink in the cwd that points to a local copy of testfile.txt. Multiple entries can be specified as:

```
-cacheFile hdfs://host:fs_port/user/testfile1.txt#testlink1 -cacheFile hdfs://host:fs_port/user/testfile2.txt#testlink2
```

The -cacheArchive option allows you to copy jars locally to the cwd of tasks and automatically unjar the files. For example:

```
-cacheArchive hdfs://host:fs_port/user/testfile.jar#testlink3
```

In the example above, a symlink testlink3 is created in the current working directory of tasks. This symlink points to the directory that stores the unjarred contents of the uploaded jar file.

Here's another example of the -cacheArchive option. Here, the input.txt file has two lines specifying the names of the two files: testlink/cache.txt and testlink/cache2.txt. "testlink" is a symlink to the archived directory, which has the files "cache.txt" and "cache2.txt".

```
$HADOOP_HOME/bin/hadoop  jar $HADOOP_HOME/hadoop-streaming.jar \
                  -input "/user/me/samples/cachefile/input.txt"  \
                  -mapper "xargs cat"  \
                  -reducer "cat"  \
                  -output "/user/me/samples/cachefile/out" \  
                  -cacheArchive 'hdfs://hadoop-nn1.example.com/user/me/samples/cachefile/cachedir.jar#testlink' \  
                  -jobconf mapred.map.tasks=1 \
                  -jobconf mapred.reduce.tasks=1 \ 
                  -jobconf mapred.job.name="Experiment"

$ ls test_jar/
cache.txt  cache2.txt

$ jar cvf cachedir.jar -C test_jar/ .
added manifest
adding: cache.txt(in = 30) (out= 29)(deflated 3%)
adding: cache2.txt(in = 37) (out= 35)(deflated 5%)

$ hadoop dfs -put cachedir.jar samples/cachefile

$ hadoop dfs -cat /user/me/samples/cachefile/input.txt
testlink/cache.txt
testlink/cache2.txt

$ cat test_jar/cache.txt 
This is just the cache string

$ cat test_jar/cache2.txt 
This is just the second cache string

$ hadoop dfs -ls /user/me/samples/cachefile/out      
Found 1 items
/user/me/samples/cachefile/out/part-00000  <r 3>   69

$ hadoop dfs -cat /user/me/samples/cachefile/out/part-00000
This is just the cache string   
This is just the second cache string
```

### Specifying Additional Configuration Variables for Jobs

You can specify additional configuration variables by using "-jobconf <n>=<v>". For example:

```
$HADOOP_HOME/bin/hadoop  jar $HADOOP_HOME/hadoop-streaming.jar \
    -input myInputDirs \
    -output myOutputDir \
    -mapper org.apache.hadoop.mapred.lib.IdentityMapper\
    -reducer /bin/wc \
    -jobconf mapred.reduce.tasks=2
```

The -jobconf mapred.reduce.tasks=2 in the above example specifies to use two reducers for the job.

For more details on the jobconf parameters see:
hadoop-default.html

### Other Supported Options

Other options you may specify for a streaming job are described here:

| Parameter | Optional/Required | Description |
| --- | --- | --- |
| -cluster name | Optional | Switch between local Hadoop and one or more remote clusters |
| -dfs host:port or local | Optional | Override the HDFS configuration for the job |
| -jt host:port or local | Optional | Override the JobTracker configuration for the job |
| -additionalconfspec specfile | Optional | Specifies a set of configuration variables in an XML file like hadoop-site.xml, instead of using multiple options of type "-jobconf name=value" |
| -cmdenv name=value | Optional | Pass env var to streaming commands |
| -cacheFile fileNameURI | Optional | Specify a file to be uploaded to the HDFS |
| -cacheArchive fileNameURI | Optional | Specify a jar file to be uploaded to the HDFS. This jar file is unjarred automatically in the cwd of the task |
| -inputreader JavaClassName | Optional | For backwards-compatibility: specifies a record reader class (instead of an input format class) |
| -verbose | Optional | Verbose output |

To switch between "local" Hadoop and one or more remote Hadoop clusters use -cluster <name>.
By default, hadoop-default.xml and hadoop-site.xml are used. The -cluster <name> option will cause $HADOOP\_HOME/conf/hadoop-<name>.xml to be used instead.

To change the local temp directory use:

```
  -jobconf dfs.data.dir=/tmp
```

To specify additional local temp directories use:

```
   -jobconf mapred.local.dir=/tmp/local
   -jobconf mapred.system.dir=/tmp/system
   -jobconf mapred.temp.dir=/tmp/temp
```

For more details on jobconf parameters see:
hadoop-default.html

To set an environment variable in a streaming command use:

```
-cmdenv EXAMPLE_DIR=/home/example/dictionaries/
```

## More usage examples

### Customizing the Way to Split Lines into Key/Value Pairs

As noted earlier, when the Map/Reduce framework reads a line from the stdout of the mapper, it splits the line into a key/value pair. By default, the prefix of the line up to the first tab character is the key and the the rest of the line (excluding the tab character) is the value.

However, you can customize this default. You can specify a field separator other than the tab character (the default), and you can specify the nth (n >= 1) character rather than the first character in a line (the default) as the separator between the key and value. For example:

```
$HADOOP_HOME/bin/hadoop  jar $HADOOP_HOME/hadoop-streaming.jar \
    -input myInputDirs \
    -output myOutputDir \
    -mapper org.apache.hadoop.mapred.lib.IdentityMapper \
    -reducer org.apache.hadoop.mapred.lib.IdentityReducer \
    -jobconf stream.map.output.field.separator=. \
    -jobconf stream.num.map.output.key.fields=4
```

In the above example, "-jobconf stream.map.output.field.separator=." specifies "." as the field separator for the map outputs, and the prefix up to the fourth "." in a line will be the key and the rest of the line (excluding the fourth ".") will be the value. If a line has less than four "."s, then the whole line will be the key and the value will be an empty Text object (like the one created by new Text("")).

Similarly, you can use "-jobconf stream.reduce.output.field.separator=SEP" and "-jobconf stream.num.reduce.output.fields=NUM" to specify the nth field separator in a line of the reduce outputs as the separator between the key and the value.

### A Useful Partitioner Class (secondary sort, the -partitioner org.apache.hadoop.mapred.lib.KeyFieldBasedPartitioner option)

Hadoop has a library class, org.apache.hadoop.mapred.lib.KeyFieldBasedPartitioner, that is useful for many applications. This class allows the Map/Reduce framework to partition the map outputs based on prefixes of keys, not the whole keys. For example:

```
$HADOOP_HOME/bin/hadoop  jar $HADOOP_HOME/hadoop-streaming.jar \
    -input myInputDirs \
    -output myOutputDir \
    -mapper org.apache.hadoop.mapred.lib.IdentityMapper \
    -reducer org.apache.hadoop.mapred.lib.IdentityReducer \
    -partitioner org.apache.hadoop.mapred.lib.KeyFieldBasedPartitioner \
    -jobconf stream.map.output.field.separator=. \
    -jobconf stream.num.map.output.key.fields=4 \
    -jobconf map.output.key.field.separator=. \
    -jobconf num.key.fields.for.partition=2 \
    -jobconf mapred.reduce.tasks=12
```

Here, *-jobconf stream.map.output.field.separator=.* and *-jobconf stream.num.map.output.key.fields=4* are as explained in previous example. The two variables are used by streaming to identify the key/value pair of mapper.

The map output keys of the above Map/Reduce job normally have four fields separated by ".". However, the Map/Reduce framework will partition the map outputs by the first two fields of the keys using the *-jobconf num.key.fields.for.partition=2* option. Here, *-jobconf map.output.key.field.separator=.* specifies the separator for the partition. This guarantees that all the key/value pairs with the same first two fields in the keys will be partitioned into the same reducer.

*This is effectively equivalent to specifying the first two fields as the primary key and the next two fields as the secondary. The primary key is used for partitioning, and the combination of the primary and secondary keys is used for sorting.* A simple illustration is shown here:

Output of map (the keys)

```
11.12.1.2
11.14.2.3
11.11.4.1
11.12.1.1
11.14.2.2
```

Partition into 3 reducers (the first 2 fields are used as keys for partition)

```
11.11.4.1
-----------
11.12.1.2
11.12.1.1
-----------
11.14.2.3
11.14.2.2
```

Sorting within each partition for the reducer(all 4 fields used for sorting)

```
11.11.4.1
-----------
11.12.1.1
11.12.1.2
-----------
11.14.2.2
11.14.2.3
```

### Working with the Hadoop Aggregate Package (the -reduce aggregate option)

Hadoop has a library package called "Aggregate" (
https://svn.apache.org/repos/asf/hadoop/core/trunk/src/mapred/org/apache/hadoop/mapred/lib/aggregate).
Aggregate provides a special reducer class and a special combiner class, and
a list of simple aggregators that perform aggregations such as "sum", "max",
"min" and so on over a sequence of values. Aggregate allows you to define a
mapper plugin class that is expected to generate "aggregatable items" for each
input key/value pair of the mappers. The combiner/reducer will aggregate those
aggregatable items by invoking the appropriate aggregators.

To use Aggregate, simply specify "-reducer aggregate":

```
$HADOOP_HOME/bin/hadoop  jar $HADOOP_HOME/hadoop-streaming.jar \
    -input myInputDirs \
    -output myOutputDir \
    -mapper myAggregatorForKeyCount.py \
    -reducer aggregate \
    -file myAggregatorForKeyCount.py \
    -jobconf mapred.reduce.tasks=12
```

The python program myAggregatorForKeyCount.py looks like:

```
#!/usr/bin/python

import sys;

def generateLongCountToken(id):
    return "LongValueSum:" + id + "\t" + "1"

def main(argv):
    line = sys.stdin.readline();
    try:
        while line:
            line = line[:-1];
            fields = line.split("\t");
            print generateLongCountToken(fields[0]);
            line = sys.stdin.readline();
    except "end of file":
        return None
if __name__ == "__main__":
     main(sys.argv)
```

### Field Selection ( similar to unix 'cut' command)

Hadoop has a library class, org.apache.hadoop.mapred.lib.FieldSelectionMapReduce, that effectively allows you to process text data like the unix "cut" utility. The map function defined in the class treats each input key/value pair as a list of fields. You can specify the field separator (the default is the tab character). You can select an arbitrary list of fields as the map output key, and an arbitrary list of fields as the map output value. Similarly, the reduce function defined in the class treats each input key/value pair as a list of fields. You can select an arbitrary list of fields as the reduce output key, and an arbitrary list of fields as the reduce output value. For example:

```
$HADOOP_HOME/bin/hadoop  jar $HADOOP_HOME/hadoop-streaming.jar \
    -input myInputDirs \
    -output myOutputDir \
    -mapper org.apache.hadoop.mapred.lib.FieldSelectionMapReduce\
    -reducer org.apache.hadoop.mapred.lib.FieldSelectionMapReduce\
    -partitioner org.apache.hadoop.mapred.lib.KeyFieldBasedPartitioner \
    -jobconf map.output.key.field.separa=. \
    -jobconf num.key.fields.for.partition=2 \
    -jobconf mapred.data.field.separator=. \
    -jobconf map.output.key.value.fields.spec=6,5,1-3:0- \
    -jobconf reduce.output.key.value.fields.spec=0-2:5- \
    -jobconf mapred.reduce.tasks=12
```

The option "-jobconf map.output.key.value.fields.spec=6,5,1-3:0-" specifies key/value selection for the map outputs. Key selection spec and value selection spec are separated by ":". In this case, the map output key will consist of fields 6, 5, 1, 2, and 3. The map output value will consist of all fields (0- means field 0 and all
the subsequent fields).

The option "-jobconf reduce.output.key.value.fields.spec=0-2:0-" specifies key/value selection for the reduce outputs. In this case, the reduce output key will consist of fields 0, 1, 2 (corresponding to the original fields 6, 5, 1). The reduce output value will consist of all fields starting from field 5 (corresponding to all the original fields).

## Frequently Asked Questions

### How do I use Hadoop Streaming to run an arbitrary set of (semi-)independent tasks?

Often you do not need the full power of Map Reduce, but only need to run multiple instances of the same program - either on different parts of the data, or on the same data, but with different parameters. You can use Hadoop Streaming to do this.

### How do I process files, one per map?

As an example, consider the problem of zipping (compressing) a set of files across the hadoop cluster. You can achieve this using either of these methods:

1. Hadoop Streaming and custom mapper script:
   - Generate a file containing the full HDFS path of the input files. Each map task would get one file name as input.
   - Create a mapper script which, given a filename, will get the file to local disk, gzip the file and put it back in the desired output directory
2. The existing Hadoop Framework:
   - Add these commands to your main function:

     ```
            FileOutputFormat.setCompressOutput(conf, true);
            FileOutputFormat.setOutputCompressorClass(conf, org.apache.hadoop.io.compress.GzipCodec.class);
            conf.setOutputFormat(NonSplitableTextInputFormat.class);
            conf.setNumReduceTasks(0);
     ```
   - Write your map function:

     ```
            public void map(WritableComparable key, Writable value, 
                                    OutputCollector output, 
                                    Reporter reporter) throws IOException {
                 output.collect((Text)value, null);
            }
     ```
   - Note that the output filename will not be the same as the original filename

### How many reducers should I use?

See the Hadoop Wiki for details: Reducer

### If I set up an alias in my shell script, will that work after -mapper, i.e. say I do: alias c1='cut -f1'. Will -mapper "c1" work?

Using an alias will not work, but variable substitution is allowed as shown in this example:

```
$ hadoop dfs -cat samples/student_marks
alice   50
bruce   70
charlie 80
dan     75

$ c2='cut -f2'; $HADOOP_HOME/bin/hadoop jar $HADOOP_HOME/hadoop-streaming.jar \
    -input /user/me/samples/student_marks 
    -mapper \"$c2\" -reducer 'cat'  
    -output /user/me/samples/student_out 
    -jobconf mapred.job.name='Experiment'

$ hadoop dfs -ls samples/student_out
Found 1 items/user/me/samples/student_out/part-00000    <r 3>   16

$ hadoop dfs -cat samples/student_out/part-00000
50
70
75
80
```

### Can I use UNIX pipes? For example, will -mapper "cut -f1 | sed s/foo/bar/g" work?

Currently this does not work and gives an "java.io.IOException: Broken pipe" error. This is probably a bug that needs to be investigated.

### When I run a streaming job by distributing large executables (for example, 3.6G) through the -file option, I get a "No space left on device" error. What do I do?

The jar packaging happens in a directory pointed to by the configuration variable stream.tmpdir. The default value of stream.tmpdir is /tmp. Set the value to a directory with more space:

```
-jobconf stream.tmpdir=/export/bigspace/...
```

### How do I specify multiple input directories?

You can specify multiple input directories with multiple '-input' options:

```
 hadoop jar hadoop-streaming.jar -input '/user/foo/dir1' -input '/user/foo/dir2'
```

### How do I generate output files with gzip format?

Instead of plain text files, you can generate gzip files as your generated output. Pass '-jobconf mapred.output.compress=true -jobconf mapred.output.compression.codec=org.apache.hadoop.io.compress.GzipCode' as option to your streaming job.

### How do I provide my own input/output format with streaming?

At least as late as version 0.14, Hadoop does not support multiple jar files. So, when specifying your own custom classes you will have to pack them along with the streaming jar and use the custom jar instead of the default hadoop streaming jar.

### How do I parse XML documents using streaming?

You can use the record reader StreamXmlRecordReader to process XML documents.

```
hadoop jar hadoop-streaming.jar -inputreader "StreamXmlRecord,begin=BEGIN_STRING,end=END_STRING" ..... (rest of the command)
```

Anything found between BEGIN\_STRING and END\_STRING would be treated as one record for map tasks.

### How do I update counters in streaming applications?

A streaming process can use the stderr to emit counter information.
reporter:counter:<group>,<counter>,<amount>
should be sent to stderr to update the counter.

### How do I update status in streaming applications?

A streaming process can use the stderr to emit status information.
To set a status, reporter:status:<message> should be sent
to stderr.

Copyright ©
2007 The Apache Software Foundation.
