# Supplementary material for: Provenance based data integrity checking and verification in cloud environments
Source: PLoS One. 2017 May 17;12(5):e0177576. doi: 10.1371/journal.pone.0177576 (PMC5435237; doi:10.1371/journal.pone.0177576)
Supplement: S1 Dataset — Files in the Dataset are utilized for performance measurement. (ZIP) [file pone.0177576.s001.zip › dataset/pdf/cluster_setup - Copy (2) - Copy.pdf]

Hadoop Cluster Setup

Table of contents

1 Purpose......2
2 Pre-requisites......2
3 Installation......2
4 Configuration......2
4.1 Configuration Files......2
4.2 Site Configuration......3
5 Hadoop Rack Awareness......7
6 Hadoop Startup......7
7 Hadoop Shutdown......7

Copyright © 2007 The Apache Software Foundation. All rights reserved.

1. Purpose

This document describes how to install, configure and manage non-trivial Hadoop clusters ranging from a few nodes to extremely large clusters with thousands of nodes.

If you are looking to install Hadoop on a single machine to play with it, you can find relevant details here.

2. Pre-requisites

1. Make sure all requisite software is installed on all nodes in your cluster.
2. Get the Hadoop software.

3. Installation

Installing a Hadoop cluster typically involves unpacking the software on all the machines in the cluster.

Typically one machine in the cluster is designated as the `NameNode` and another machine the as `JobTracker`, exclusively. These are the *masters*. The rest of the machines in the cluster act as both `DataNode` *and* `TaskTracker`. These are the *slaves*.

The root of the distribution is referred to as `HADOOP_HOME`. All machines in the cluster usually have the same `HADOOP_HOME` path.

4. Configuration

The following sections describe how to configure a Hadoop cluster.

4.1. Configuration Files

Hadoop configuration is driven by two important configuration files found in the `conf/` directory of the distribution:

1. hadoop-default.xml - Read-only default configuration.
2. *hadoop-site.xml* - Site-specific configuration.

To learn more about how the Hadoop framework is controlled by these configuration files, look here.

Additionally, you can control the Hadoop scripts found in the `bin/` directory of the distribution, by setting site-specific values via the `conf/hadoop-env.sh`.

Copyright © 2007 The Apache Software Foundation. All rights reserved.

4.2. Site Configuration

To configure the the Hadoop cluster you will need to configure the *environment* in which the Hadoop daemons execute as well as the *configuration parameters* for the Hadoop daemons.

The Hadoop daemons are `NameNode`/`DataNode` and `JobTracker`/`TaskTracker`.

4.2.1. Configuring the Environment of the Hadoop Daemons

Administrators should use the `conf/hadoop-env.sh` script to do site-specific customization of the Hadoop daemons' process environment.

At the very least you should specify the `JAVA_HOME` so that it is correctly defined on each remote node.

Administrators can configure individual daemons using the configuration options `HADOOP_*_OPTS`. Various options available are shown below in the table.

Daemon	Configure Options
NameNode	HADOOP_NAMENODE_OPTS
DataNode	HADOOP_DATANODE_OPTS
SecondaryNamenode	HADOOP_SECONDARYNAMENODE_OPTS
JobTracker	HADOOP_JOBTRACKER_OPTS
TaskTracker	HADOOP_TASKTRACKER_OPTS

For example, To configure Namenode to use parallelGC, the following statement should be added in `hadoop-env.sh` :

```
export HADOOP_NAMENODE_OPTS="-XX:+UseParallelGC
${HADOOP_NAMENODE_OPTS}"
```

Other useful configuration parameters that you can customize include:

- `HADOOP_LOG_DIR` - The directory where the daemons' log files are stored. They are automatically created if they don't exist.
- `HADOOP_HEAPSIZE` - The maximum amount of heapsize to use, in MB e.g. `1000MB`. This is used to configure the heap size for the hadoop daemon. By default, the value is `1000MB`.

4.2.2. Configuring the Hadoop Daemons

Copyright © 2007 The Apache Software Foundation. All rights reserved.

This section deals with important parameters to be specified in the `conf/hadoop-site.xml` for the Hadoop cluster.

Parameter	Value	Notes
fs.default.name	URI of `NameNode`.	*hdfs://hostname/*
mapred.job.tracker	Host or IP and port of `JobTracker`.	*host:port* pair.
dfs.name.dir	Path on the local filesystem where the `NameNode` stores the namespace and transactions logs persistently.	If this is a comma-delimited list of directories then the name table is replicated in all of the directories, for redundancy.
dfs.data.dir	Comma separated list of paths on the local filesystem of a `DataNode` where it should store its blocks.	If this is a comma-delimited list of directories, then data will be stored in all named directories, typically on different devices.
mapred.system.dir	Path on the HDFS where where the Map/Reduce framework stores system files e.g. `/hadoop/mapred/system/`.	This is in the default filesystem (HDFS) and must be accessible from both the server and client machines.
mapred.local.dir	Comma-separated list of paths on the local filesystem where temporary Map/Reduce data is written.	Multiple paths help spread disk i/o.
mapred.tasktracker.{map|reduce	The maximum number of Map/Reduce tasks, which are run simultaneously on a given `TaskTracker`, individually.	Defaults to 2 (2 maps and 2 reduces), but vary it depending on your hardware.
dfs.hosts/dfs.hosts.exclude	List of permitted/excluded DataNodes.	If necessary, use these files to control the list of allowable datanodes.
mapred.hosts/mapred.hosts.excl	List of permitted/excluded TaskTrackers.	If necessary, use these files to control the list of allowable TaskTrackers.

Typically all the above parameters are marked as [final](#) to ensure that they cannot be overriden by user-applications.

4.2.2.1. Real-World Cluster Configurations

Copyright © 2007 The Apache Software Foundation. All rights reserved.

This section lists some non-default configuration parameters which have been used to run the *sort* benchmark on very large clusters.

- Some non-default configuration values used to run sort900, that is 9TB of data sorted on a cluster with 900 nodes:

Parameter	Value	Notes
dfs.block.size	134217728	HDFS blocksize of 128MB for large file-systems.
dfs.namenode.handler.count	40	More NameNode server threads to handle RPCs from large number of DataNodes.
mapred.reduce.parallel.copie	20	Higher number of parallel copies run by reduces to fetch outputs from very large number of maps.
mapred.child.java.opts	-Xmx512M	Larger heap-size for child jvms of maps/reduces.
fs.inmemory.size.mb	200	Larger amount of memory allocated for the in-memory file-system used to merge map-outputs at the reduces.
io.sort.factor	100	More streams merged at once while sorting files.
io.sort.mb	200	Higher memory-limit while sorting data.
io.file.buffer.size	131072	Size of read/write buffer used in SequenceFiles.

- Updates to some configuration values to run sort1400 and sort2000, that is 14TB of data sorted on 1400 nodes and 20TB of data sorted on 2000 nodes:

Parameter	Value	Notes
mapred.job.tracker.handler.c	60	More JobTracker server threads to handle RPCs from large number of TaskTrackers.
mapred.reduce.parallel.copie	50	

Copyright © 2007 The Apache Software Foundation. All rights reserved.

tasktracker.http.threads	50	More worker threads for the TaskTracker's http server. The http server is used by reduces to fetch intermediate map-outputs.
mapred.child.java.opts	-Xmx1024M	Larger heap-size for child jvms of maps/reduces.

4.2.3. Slaves

Typically you choose one machine in the cluster to act as the `NameNode` and one machine as to act as the `JobTracker`, exclusively. The rest of the machines act as both a `DataNode` and `TaskTracker` and are referred to as *slaves*.

List all slave hostnames or IP addresses in your `conf/slaves` file, one per line.

4.2.4. Logging

Hadoop uses the [Apache log4j](http://logging.apache.org/log4j/) via the [Apache Commons Logging](http://commons.apache.org/logging/) framework for logging. Edit the `conf/log4j.properties` file to customize the Hadoop daemons' logging configuration (log-formats and so on).

4.2.4.1. History Logging

The job history files are stored in central location `hadoop.job.history.location` which can be on DFS also, whose default value is `${HADOOP_LOG_DIR}/history`. The history web UI is accessible from job tracker web UI.

The history files are also logged to user specified directory `hadoop.job.history.user.location` which defaults to job output directory. The files are stored in "_logs/history/" in the specified directory. Hence, by default they will be in "mapred.output.dir/_logs/history/". User can stop logging by giving the value `none` for `hadoop.job.history.user.location`

User can view the history logs summary in specified directory using the following command
`$ bin/hadoop job -history output-dir`
This command will print job details, failed and killed tip details.
More details about the job such as successful tasks and task attempts made for each task can be viewed using the following command
`$ bin/hadoop job -history all output-dir`

Once all the necessary configuration is complete, distribute the files to the

Copyright © 2007 The Apache Software Foundation. All rights reserved.

HADOOP_CONF_DIR directory on all the machines, typically ${HADOOP_HOME}/conf.

5. Hadoop Rack Awareness

The HDFS and the Map/Reduce components are rack-aware.

The NameNode and the JobTracker obtains the rack id of the slaves in the cluster by invoking an API resolve in an administrator configured module. The API resolves the slave's DNS name (also IP address) to a rack id. What module to use can be configured using the configuration item topology.node.switch.mapping.impl. The default implementation of the same runs a script/command configured using topology.script.file.name. If topology.script.file.name is not set, the rack id /default-rack is returned for any passed IP address. The additional configuration in the Map/Reduce part is mapred.cache.task.levels which determines the number of levels (in the network topology) of caches. So, for example, if it is the default value of 2, two levels of caches will be constructed - one for hosts (host -> task mapping) and another for racks (rack -> task mapping).

6. Hadoop Startup

To start a Hadoop cluster you will need to start both the HDFS and Map/Reduce cluster.

Format a new distributed filesystem:

```
$ bin/hadoop namenode -format
```

Start the HDFS with the following command, run on the designated NameNode:

```
$ bin/start-dfs.sh
```

The bin/start-dfs.sh script also consults the ${HADOOP_CONF_DIR}/slaves file on the NameNode and starts the DataNode daemon on all the listed slaves.

Start Map-Reduce with the following command, run on the designated JobTracker:

```
$ bin/start-mapred.sh
```

The bin/start-mapred.sh script also consults the ${HADOOP_CONF_DIR}/slaves file on the JobTracker and starts the TaskTracker daemon on all the listed slaves.

7. Hadoop Shutdown

Stop HDFS with the following command, run on the designated NameNode:

```
$ bin/stop-dfs.sh
```


Copyright © 2007 The Apache Software Foundation. All rights reserved.

The `bin/stop-dfs.sh` script also consults the `${HADOOP_CONF_DIR}/slaves` file on the `NameNode` and stops the `DataNode` daemon on all the listed slaves.

Stop Map/Reduce with the following command, run on the designated the designated `JobTracker`:
`$ bin/stop-mapred.sh`

The `bin/stop-mapred.sh` script also consults the `${HADOOP_CONF_DIR}/slaves` file on the `JobTracker` and stops the `TaskTracker` daemon on all the listed slaves.

Copyright © 2007 The Apache Software Foundation. All rights reserved.
