# Supplementary material for: Provenance based data integrity checking and verification in cloud environments
Source: PLoS One. 2017 May 17;12(5):e0177576. doi: 10.1371/journal.pone.0177576 (PMC5435237; doi:10.1371/journal.pone.0177576)
Supplement: S1 Dataset — Files in the Dataset are utilized for performance measurement. (ZIP) [file pone.0177576.s001.zip › dataset/pdf/commands_manual - Copy (2) - Copy.pdf]

Commands Manual

Table of contents

1 Overview...2
1.1 Generic Options...2
2 User Commands...3
2.1 archive...3
2.2 distcp...3
2.3 fs...3
2.4 fsck...3
2.5 jar...4
2.6 job...4
2.7 pipes...5
2.8 version...6
2.9 CLASSNAME...6
3 Administration Commands...6
3.1 balancer...6
3.2 daemonlog...6
3.3 datanode...7
3.4 dfsadmin...7
3.5 jobtracker...9
3.6 namenode...9
3.7 secondarynamenode...9
3.8 tasktracker...10

Copyright © 2007 The Apache Software Foundation. All rights reserved.

1. Overview

All the hadoop commands are invoked by the bin/hadoop script. Running hadoop script without any arguments prints the description for all commands.

```
Usage: hadoop [--config confdir] [COMMAND] [GENERIC_OPTIONS]
[COMMAND_OPTIONS]
```

Hadoop has an option parsing framework that employs parsing generic options as well as running classes.

COMMAND_OPTION	Description
`--config confdir`	Overwrites the default Configuration directory. Default is ${HADOOP_HOME}/conf.
`GENERIC_OPTIONS`	The common set of options supported by multiple commands.
`COMMAND`<br>`COMMAND_OPTIONS`	Various commands with their options are described in the following sections. The commands have been grouped into User Commands and Administration Commands.

1.1. Generic Options

Following are supported by dfsadmin, fs, fsck and job. Applications should implement Tool to support GenericOptions.

GENERIC_OPTION	Description
-conf <configuration file>	Specify an application configuration file.
-D <property=value>	Use value for given property.
-fs <local|namenode:port>	Specify a namenode.
-jt <local|jobtracker:port>	Specify a job tracker. Applies only to job.
-files <comma separated list of files>	Specify comma separated files to be copied to the map reduce cluster. Applies only to job.
-libjars <comma seperated list of jars>	Specify comma separated jar files to include in the classpath. Applies only to job.
-archives <comma separated list of archives>	Specify comma separated archives to be unarchived on the compute machines. Applies only to job.

Copyright © 2007 The Apache Software Foundation. All rights reserved.

2. User Commands

Commands useful for users of a hadoop cluster.

2.1. archive

Creates a hadoop archive. More information can be found at Hadoop Archives.

`Usage: hadoop archive -archiveName NAME <src>* <dest>`

COMMAND_OPTION	Description
`-archiveName NAME`	Name of the archive to be created.
`src`	Filesystem pathnames which work as usual with regular expressions.
`dest`	Destination directory which would contain the archive.

2.2. distcp

Copy file or directories recursively. More information can be found at DistCp Guide.

`Usage: hadoop distcp <srcurl> <desturl>`

COMMAND_OPTION	Description
`srcurl`	Source Url
`desturl`	Destination Url

2.3. fs

`Usage: hadoop fs [GENERIC_OPTIONS] [COMMAND_OPTIONS]`

Runs a generic filesystem user client.

The various COMMAND_OPTIONS can be found at HDFS Shell Guide.

2.4. fsck

Runs a HDFS filesystem checking utility. See Fsck for more info.

`Usage: hadoop fsck [GENERIC_OPTIONS] <path> [-move | -delete | -openforwrite] [-files [-blocks [-locations | -racks]]]`

Copyright © 2007 The Apache Software Foundation. All rights reserved.

COMMAND_OPTION	Description
`<path>`	Start checking from this path.
`-move`	Move corrupted files to /lost+found
`-delete`	Delete corrupted files.
`-openforwrite`	Print out files opened for write.
`-files`	Print out files being checked.
`-blocks`	Print out block report.
`-locations`	Print out locations for every block.
`-racks`	Print out network topology for data-node locations.

2.5. jar

Runs a jar file. Users can bundle their Map Reduce code in a jar file and execute it using this command.

`Usage: hadoop jar <jar> [mainClass] args...`

The streaming jobs are run via this command. Examples can be referred from Streaming examples

Word count example is also run using jar command. It can be referred from Wordcount example

2.6. job

Command to interact with Map Reduce Jobs.

`Usage: hadoop job [GENERIC_OPTIONS] [-submit <job-file>] | [-status <job-id>] | [-counter <job-id> <group-name> <counter-name>] | [-kill <job-id>] | [-events <job-id> <from-event-#> <#-of-events>] | [-history [all] <jobOutputDir>] | [-list [all]] | [-kill-task <task-id>] | [-fail-task <task-id>]`

COMMAND_OPTION	Description
`-submit <job-file>`	Submits the job.
`-status <job-id>`	Prints the map and reduce completion

Copyright © 2007 The Apache Software Foundation. All rights reserved.

	percentage and all job counters.
`-counter <job-id> <group-name> <counter-name>`	Prints the counter value.
`-kill <job-id>`	Kills the job.
`-events <job-id> <from-event-#> <#-of-events>`	Prints the events' details received by jobtracker for the given range.
`-history [all] <jobOutputDir>`	-history <jobOutputDir> prints job details, failed and killed tip details. More details about the job such as successful tasks and task attempts made for each task can be viewed by specifying the [all] option.
`-list [all]`	-list all displays all jobs. -list displays only jobs which are yet to complete.
`-kill-task <task-id>`	Kills the task. Killed tasks are NOT counted against failed attempts.
`-fail-task <task-id>`	Fails the task. Failed tasks are counted against failed attempts.

2.7. pipes

Runs a pipes job.

```
Usage: hadoop pipes [-conf <path>] [-jobconf <key=value>,
<key=value>, ...] [-input <path>] [-output <path>] [-jar <jar
file>] [-inputformat <class>] [-map <class>] [-partitioner
<class>] [-reduce <class>] [-writer <class>] [-program
<executable>] [-reduces <num>]
```

COMMAND_OPTION	Description
`-conf <path>`	Configuration for job
`-jobconf <key=value>, <key=value>, ...`	Add/override configuration for job
`-input <path>`	Input directory
`-output <path>`	Output directory
`-jar <jar file>`	Jar filename
`-inputformat <class>`	InputFormat class

Copyright © 2007 The Apache Software Foundation. All rights reserved.

`-map <class>`	Java Map class
`-partitioner <class>`	Java Partitioner
`-reduce <class>`	Java Reduce class
`-writer <class>`	Java RecordWriter
`-program <executable>`	Executable URI
`-reduces <num>`	Number of reduces

2.8. version

Prints the version.

```
Usage: hadoop version
```

2.9. CLASSNAME

hadoop script can be used to invoke any class.

```
Usage: hadoop CLASSNAME
```

Runs the class named CLASSNAME.

3. Administration Commands

Commands useful for administrators of a hadoop cluster.

3.1. balancer

Runs a cluster balancing utility. An administrator can simply press Ctrl-C to stop the rebalancing process. See Rebalancer for more details.

```
Usage: hadoop balancer [-threshold <threshold>]
```

COMMAND_OPTION	Description
`-threshold <threshold>`	Percentage of disk capacity. This overwrites the default threshold.

3.2. daemonlog

Get/Set the log level for each daemon.

Copyright © 2007 The Apache Software Foundation. All rights reserved.

```
Usage: hadoop daemonlog -getlevel <host:port> <name>
Usage: hadoop daemonlog -setlevel <host:port> <name> <level>
```

COMMAND_OPTION	Description
`-getlevel <host:port> <name>`	Prints the log level of the daemon running at <host:port>. This command internally connects to http://<host:port>/logLevel?log=<name>
`-setlevel <host:port> <name> <level>`	Sets the log level of the daemon running at <host:port>. This command internally connects to http://<host:port>/logLevel?log=<name>

3.3. datanode

Runs a HDFS datanode.

```
Usage: hadoop datanode [-rollback]
```

COMMAND_OPTION	Description
`-rollback`	Rollsback the datanode to the previous version. This should be used after stopping the datanode and distributing the old hadoop version.

3.4. dfsadmin

Runs a HDFS dfsadmin client.

```
Usage: hadoop dfsadmin [GENERIC_OPTIONS] [-report] [-safemode
enter | leave | get | wait] [-refreshNodes] [-finalizeUpgrade]
[-upgradeProgress status | details | force] [-metasave
filename] [-setQuota <quota> <dirname>...<dirname>] [-clrQuota
<dirname>...<dirname>] [-help [cmd]]
```

COMMAND_OPTION	Description
`-report`	Reports basic filesystem information and statistics.
`-safemode enter | leave | get | wait`	Safe mode maintenance command. Safe mode is a Namenode state in which it<br>1. does not accept changes to the name space (read-only)<br>2. does not replicate or delete blocks.<br>Safe mode is entered automatically at Namenode startup, and leaves safe mode

Copyright © 2007 The Apache Software Foundation. All rights reserved.

	automatically when the configured minimum percentage of blocks satisfies the minimum replication condition. Safe mode can also be entered manually, but then it can only be turned off manually as well.
`-refreshNodes`	Re-read the hosts and exclude files to update the set of Datanodes that are allowed to connect to the Namenode and those that should be decommissioned or recommissioned.
`-finalizeUpgrade`	Finalize upgrade of HDFS. Datanodes delete their previous version working directories, followed by Namenode doing the same. This completes the upgrade process.
`-upgradeProgress status | details | force`	Request current distributed upgrade status, a detailed status or force the upgrade to proceed.
`-metasave filename`	Save Namenode's primary data structures to <filename> in the directory specified by hadoop.log.dir property. <filename> will contain one line for each of the following<br>1. Datanodes heart beating with Namenode<br>2. Blocks waiting to be replicated<br>3. Blocks currrently being replicated<br>4. Blocks waiting to be deleted
`-setQuota <quota> <dirname>...<dirname>`	Set the quota <quota> for each directory <dirname>. The directory quota is a long integer that puts a hard limit on the number of names in the directory tree.<br>Best effort for the directory, with faults reported if<br>1. N is not a positive integer, or<br>2. user is not an administrator, or<br>3. the directory does not exist or is a file, or<br>4. the directory would immediately exceed the new quota.
`-clrQuota <dirname>...<dirname>`	Clear the quota for each directory <dirname>.<br>Best effort for the directory. with fault reported if<br>1. the directory does not exist or is a file, or<br>2. user is not an administrator.<br>It does not fault if the directory has no quota.
`-help [cmd]`	Displays help for the given command or all commands if none is specified.

Copyright © 2007 The Apache Software Foundation. All rights reserved.

3.5. jobtracker

Runs the MapReduce job Tracker node.

`Usage: hadoop jobtracker`

3.6. namenode

Runs the namenode. More info about the upgrade, rollback and finalize is at Upgrade Rollback

`Usage: hadoop namenode [-format] | [-upgrade] | [-rollback] | [-finalize] | [-importCheckpoint]`

COMMAND_OPTION	Description
`-format`	Formats the namenode. It starts the namenode, formats it and then shut it down.
`-upgrade`	Namenode should be started with upgrade option after the distribution of new hadoop version.
`-rollback`	Rollsback the namenode to the previous version. This should be used after stopping the cluster and distributing the old hadoop version.
`-finalize`	Finalize will remove the previous state of the files system. Recent upgrade will become permanent. Rollback option will not be available anymore. After finalization it shuts the namenode down.
`-importCheckpoint`	Loads image from a checkpoint directory and save it into the current one. Checkpoint dir is read from property fs.checkpoint.dir

3.7. secondarynamenode

Runs the HDFS secondary namenode. See Secondary Namenode for more info.

`Usage: hadoop secondarynamenode [-checkpoint [force]] | [-geteditsize]`

COMMAND_OPTION	Description
`-checkpoint [force]`	Checkpoints the Secondary namenode if

Copyright © 2007 The Apache Software Foundation. All rights reserved.

	EditLog size >= fs.checkpoint.size. If -force is used, checkpoint irrespective of EditLog size.
`-geteditsize`	Prints the EditLog size.

3.8. tasktracker

Runs a MapReduce task Tracker node.

`Usage: hadoop tasktracker`

Copyright © 2007 The Apache Software Foundation. All rights reserved.
