# Supplementary material for: Provenance based data integrity checking and verification in cloud environments
Source: PLoS One. 2017 May 17;12(5):e0177576. doi: 10.1371/journal.pone.0177576 (PMC5435237; doi:10.1371/journal.pone.0177576)
Supplement: S1 Dataset — Files in the Dataset are utilized for performance measurement. (ZIP) [file pone.0177576.s001.zip › dataset/pdf/distcp - Copy (2) - Copy.pdf]

DistCp

Table of contents

1 Overview....................2
2 Usage....................2
2.1 Basic....................2
2.2 Options....................3
3 Appendix....................5
3.1 Map sizing....................5
3.2 Copying between versions of HDFS....................6
3.3 Map/Reduce and other side-effects....................6

Copyright © 2007 The Apache Software Foundation. All rights reserved.

1. Overview

DistCp (distributed copy) is a tool used for large inter/intra-cluster copying. It uses Map/Reduce to effect its distribution, error handling and recovery, and reporting. It expands a list of files and directories into input to map tasks, each of which will copy a partition of the files specified in the source list. Its Map/Reduce pedigree has endowed it with some quirks in both its semantics and execution. The purpose of this document is to offer guidance for common tasks and to elucidate its model.

2. Usage

2.1. Basic

The most common invocation of DistCp is an inter-cluster copy:

```
bash$ hadoop distcp hdfs://nn1:8020/foo/bar \
                    hdfs://nn2:8020/bar/foo
```

This will expand the namespace under `/foo/bar` on nn1 into a temporary file, partition its contents among a set of map tasks, and start a copy on each TaskTracker from nn1 to nn2. Note that DistCp expects absolute paths.

One can also specify multiple source directories on the command line:

```
bash$ hadoop distcp hdfs://nn1:8020/foo/a \
                    hdfs://nn1:8020/foo/b \
                    hdfs://nn2:8020/bar/foo
```

Or, equivalently, from a file using the `-f` option:

```
bash$ hadoop distcp -f hdfs://nn1:8020/srclist \
                       hdfs://nn2:8020/bar/foo
```

Where `srclist` contains

```
    hdfs://nn1:8020/foo/a
    hdfs://nn1:8020/foo/b
```

When copying from multiple sources, DistCp will abort the copy with an error message if two sources collide, but collisions at the destination are resolved per the options specified. By default, files already existing at the destination are skipped (i.e. not replaced by the source file). A count of skipped files is reported at the end of each job, but it may be inaccurate if a copier failed for some subset of its files, but succeeded on a later attempt (see Appendix).

It is important that each TaskTracker can reach and communicate with both the source and

Copyright © 2007 The Apache Software Foundation. All rights reserved.

destination file systems. For HDFS, both the source and destination must be running the same version of the protocol or use a backwards-compatible protocol (see Copying Between Versions).

After a copy, it is recommended that one generates and cross-checks a listing of the source and destination to verify that the copy was truly successful. Since DistCp employs both Map/Reduce and the FileSystem API, issues in or between any of the three could adversely and silently affect the copy. Some have had success running with `-update` enabled to perform a second pass, but users should be acquainted with its semantics before attempting this.

It's also worth noting that if another client is still writing to a source file, the copy will likely fail. Attempting to overwrite a file being written at the destination should also fail on HDFS. If a source file is (re)moved before it is copied, the copy will fail with a FileNotFoundException.

2.2. Options

2.2.1. Option Index

Flag	Description	Notes
`-p[rbugp]`	Preserve<br>r: replication number<br>b: block size<br>u: user<br>g: group<br>p: permission	Modification times are not preserved. Also, when `-update` is specified, status updates will **not** be synchronized unless the file sizes also differ (i.e. unless the file is re-created).
`-i`	Ignore failures	As explained in the Appendix, this option will keep more accurate statistics about the copy than the default case. It also preserves logs from failed copies, which can be valuable for debugging. Finally, a failing map will not cause the job to fail before all splits are attempted.
`-log <logdir>`	Write logs to <logdir>	DistCp keeps logs of each file it attempts to copy as map output. If a map fails, the log output will not be retained if it is

Copyright © 2007 The Apache Software Foundation. All rights reserved.

		re-executed.
`-m <num_maps>`	Maximum number of simultaneous copies	Specify the number of maps to copy data. Note that more maps may not necessarily improve throughput.
`-overwrite`	Overwrite destination	If a map fails and `-i` is not specified, all the files in the split, not only those that failed, will be recopied. As discussed in the following, it also changes the semantics for generating destination paths, so users should use this carefully.
`-update`	Overwrite if src size different from dst size	As noted in the preceding, this is not a "sync" operation. The only criterion examined is the source and destination file sizes; if they differ, the source file replaces the destination file. As discussed in the following, it also changes the semantics for generating destination paths, so users should use this carefully.
`-f <urilist_uri>`	Use list at <urilist_uri> as src list	This is equivalent to listing each source on the command line. The `urilist_uri` list should be a fully qualified URI.

2.2.2. Update and Overwrite

It's worth giving some examples of `-update` and `-overwrite`. Consider a copy from `/foo/a` and `/foo/b` to `/bar/foo`, where the sources contain the following:

```
hdfs://nn1:8020/foo/a
hdfs://nn1:8020/foo/a/aa
hdfs://nn1:8020/foo/a/ab
hdfs://nn1:8020/foo/b
hdfs://nn1:8020/foo/b/ba
hdfs://nn1:8020/foo/b/ab
```

If either `-update` or `-overwrite` is set, then both sources will map an entry to

Copyright © 2007 The Apache Software Foundation. All rights reserved.

`/bar/foo/ab` at the destination. For both options, the contents of each source directory are compared with the **contents** of the destination directory. Rather than permit this conflict, DistCp will abort.

In the default case, both `/bar/foo/a` and `/bar/foo/b` will be created and neither will collide.

Now consider a legal copy using `-update`:

```
distcp -update hdfs://nn1:8020/foo/a \
               hdfs://nn1:8020/foo/b \
               hdfs://nn2:8020/bar
```

With sources/sizes:

```
hdfs://nn1:8020/foo/a
hdfs://nn1:8020/foo/a/aa 32
hdfs://nn1:8020/foo/a/ab 32
hdfs://nn1:8020/foo/b
hdfs://nn1:8020/foo/b/ba 64
hdfs://nn1:8020/foo/b/bb 32
```

And destination/sizes:

```
hdfs://nn2:8020/bar
hdfs://nn2:8020/bar/aa 32
hdfs://nn2:8020/bar/ba 32
hdfs://nn2:8020/bar/bb 64
```

Will effect:

```
hdfs://nn2:8020/bar
hdfs://nn2:8020/bar/aa 32
hdfs://nn2:8020/bar/ab 32
hdfs://nn2:8020/bar/ba 64
hdfs://nn2:8020/bar/bb 32
```

Only `aa` is not overwritten on nn2. If `-overwrite` were specified, all elements would be overwritten.

3. Appendix

3.1. Map sizing

DistCp makes a faint attempt to size each map comparably so that each copies roughly the

Copyright © 2007 The Apache Software Foundation. All rights reserved.

same number of bytes. Note that files are the finest level of granularity, so increasing the number of simultaneous copiers (i.e. maps) may not always increase the number of simultaneous copies nor the overall throughput.

If `-m` is not specified, DistCp will attempt to schedule work for `min (total_bytes / bytes.per.map, 20 * num_task_trackers)` where `bytes.per.map` defaults to 256MB.

Tuning the number of maps to the size of the source and destination clusters, the size of the copy, and the available bandwidth is recommended for long-running and regularly run jobs.

3.2. Copying between versions of HDFS

For copying between two different versions of Hadoop, one will usually use HftpFileSystem. This is a read-only FileSystem, so DistCp must be run on the destination cluster (more specifically, on TaskTrackers that can write to the destination cluster). Each source is specified as `hftp://<dfs.http.address>/<path>` (the default `dfs.http.address` is <namenode>:50070).

3.3. Map/Reduce and other side-effects

As has been mentioned in the preceding, should a map fail to copy one of its inputs, there will be several side-effects.

- Unless `-i` is specified, the logs generated by that task attempt will be replaced by the previous attempt.
- Unless `-overwrite` is specified, files successfully copied by a previous map on a re-execution will be marked as "skipped".
- If a map fails `mapred.map.max.attempts` times, the remaining map tasks will be killed (unless `-i` is set).
- If `mapred.speculative.execution` is set set `final` and `true`, the result of the copy is undefined.

Copyright © 2007 The Apache Software Foundation. All rights reserved.
