# Supplementary material for: Provenance based data integrity checking and verification in cloud environments
Source: PLoS One. 2017 May 17;12(5):e0177576. doi: 10.1371/journal.pone.0177576 (PMC5435237; doi:10.1371/journal.pone.0177576)
Supplement: S1 Dataset — Files in the Dataset are utilized for performance measurement. (ZIP) [file pone.0177576.s001.zip › dataset/pdf/hadoop_archives - Copy (2) - Copy.pdf]

# Hadoop Archives

## Table of contents

1 What are Hadoop archives? ............................................................................................ 2
2 How to create an archive? ............................................................................................2
3 How to look up files in archives? ..................................................................................2

Copyright © 2007 The Apache Software Foundation. All rights reserved.

## 1. What are Hadoop archives?

Hadoop archives are special format archives. A Hadoop archive maps to a file system directory. A Hadoop archive always has a *.har extension. A Hadoop archive directory contains metadata (in the form of _index and _masterindex) and data (part-*) files. The _index file contains the name of the files that are part of the archive and the location within the part files.

## 2. How to create an archive?

```
Usage: hadoop archive -archiveName name <src>* <dest>
```

-archiveName is the name of the archive you would like to create. An example would be foo.har. The name should have a *.har extension. The inputs are file system pathnames which work as usual with regular expressions. The destination directory would contain the archive. Note that this is a Map/Reduce job that creates the archives. You would need a map reduce cluster to run this. The following is an example:

```
hadoop archive -archiveName foo.har /user/hadoop/dir1
/user/hadoop/dir2 /user/zoo/
```

In the above example /user/hadoop/dir1 and /user/hadoop/dir2 will be archived in the following file system directory -- /user/zoo/foo.har. The sources are not changed or removed when an archive is created.

## 3. How to look up files in archives?

The archive exposes itself as a file system layer. So all the fs shell commands in the archives work but with a different URI. Also, note that archives are immutable. So, rename's, deletes and creates return an error. URI for Hadoop Archives is

```
har://scheme-hostname:port/archivepath/fileinarchive
```

If no scheme is provided it assumes the underlying filesystem. In that case the URI would look like

```
har:///archivepath/fileinarchive
```

Here is an example of archive. The input to the archives is /dir. The directory dir contains files filea, fileb. To archive /dir to /user/hadoop/foo.har, the command is

```
hadoop archive -archiveName foo.har /dir /user/hadoop
```



Copyright © 2007 The Apache Software Foundation. All rights reserved.

To get file listing for files in the created archive

```
hadoop dfs -lsr har:///user/hadoop/foo.har
```

To cat filea in archive -

```
hadoop dfs -cat har:///user/hadoop/foo.har/dir/filea
```



*Copyright © 2007 The Apache Software Foundation. All rights reserved.*
