# Supplementary material for: Provenance based data integrity checking and verification in cloud environments
Source: PLoS One. 2017 May 17;12(5):e0177576. doi: 10.1371/journal.pone.0177576 (PMC5435237; doi:10.1371/journal.pone.0177576)
Supplement: S1 Dataset — Files in the Dataset are utilized for performance measurement. (ZIP) [file pone.0177576.s001.zip › dataset/pdf/hdfs_permissions_guide - Copy (2) - Copy.pdf]

Permissions User and Administrator Guide

Table of contents

1 Overview..2
2 User Identity..2
3 Understanding the Implementation..3
4 Changes to the File System API..3
5 Changes to the Application Shell..4
6 The Super-User..4
7 The Web Server..5
8 On-line Upgrade..5
9 Configuration Parameters..5

Copyright © 2007 The Apache Software Foundation. All rights reserved.

1. Overview

The Hadoop Distributed File System implements a permissions model for files and directories that shares much of the POSIX model. Each file and directory is associated with an *owner* and a *group*. The file or directory has separate permissions for the user that is the owner, for other users that are members of the group, and for all other users. For files, the *r* permission is required to read the file, and the *w* permission is required to write or append to the file. For directories, the *r* permission is required to list the contents of the directory, the *w* permission is required to create or delete files or directories, and the *x* permission is required to access a child of the directory. In contrast to the POSIX model, there are no *sticky*, *setuid* or *setgid* bits for files as there is no notion of executable files. For directories, there no *sticky*, *setuid* or *setgid* bits directory as a simplification. Collectively, the permissions of a file or directory are its *mode*. In general, Unix customs for representing and displaying modes will be used, including the use of octal numbers in this description. When a file or directory is created, its owner is the user identity of the client process, and its group is the group of the parent directory (the BSD rule).

Each client process that accesses HDFS has a two-part identity composed of the *user name*, and *groups list*. Whenever HDFS must do a permissions check for a file or directory `foo` accessed by a client process,

- If the user name matches the owner of `foo`, then the owner permissions are tested;
- Else if the group of `foo` matches any of member of the groups list, then the group permissions are tested;
- Otherwise the the other permissions of `foo` are tested.

If a permissions check fails, the the client operation fails.

2. User Identity

In this release of Hadoop the identity of a client process is just whatever the host operating system says it is. For Unix-like systems,

- The user name is the equivalent of `` `whoami` ``;
- The group list is the equivalent of `` `bash -c groups` ``.

In the future there will be other ways of establishing user identity (think Kerberos, LDAP, and others). There is no expectation that this first method is secure in protecting one user from impersonating another. This user identity mechanism combined with the permissions model allows a cooperative community to share file system resources in an organized fashion.

Copyright © 2007 The Apache Software Foundation. All rights reserved.

In any case, the user identity mechanism is extrinsic to HDFS itself. There is no provision within HDFS for creating user identities, establishing groups, or processing user credentials.

3. Understanding the Implementation

Each file or directory operation passes the full path name to the name node, and the permissions checks are applied along the path for each operation. The client framework will implicitly associate the user identity with the connection to the name node, reducing the need for changes to the existing client API. It has always been the case that when one operation on a file succeeds, the operation might fail when repeated because the file, or some directory on the path, no longer exists. For instance, when the client first begins reading a file, it makes a first request to the name node to discover the location of the first blocks of the file. A second request made to find additional blocks may fail. On the other hand, deleting a file does not revoke access by a client that already knows the blocks of the file. With the addition of permissions, a client's access to a file may be withdrawn between requests. Again, changing permissions does not revoke the access of a client that already knows the file's blocks.

The map-reduce framework delegates the user identity by passing strings without special concern for confidentiality. The owner and group of a file or directory are stored as strings; there is no conversion from user and group identity numbers as is conventional in Unix.

The permissions features of this release did not require any changes to the behavior of data nodes. Blocks on the data nodes do not have any of the *Hadoop* ownership or permissions attributes associated with them.

4. Changes to the File System API

All methods that use a path parameter will throw `AccessControlException` if permission checking fails.

New methods:

- `public FSDataOutputStream create(Path f, FsPermission permission, boolean overwrite, int bufferSize, short replication, long blockSize, Progressable progress) throws IOException;`
- `public boolean mkdirs(Path f, FsPermission permission) throws IOException;`
- `public void setPermission(Path p, FsPermission permission) throws IOException;`
- `public void setOwner(Path p, String username, String groupname) throws IOException;`

Copyright © 2007 The Apache Software Foundation. All rights reserved.

- `public FileStatus getFileStatus(Path f) throws IOException;` will additionally return the user, group and mode associated with the path.

The mode of a new file or directory is restricted my the `umask` set as a configuration parameter. When the existing `create(path, …)` method (*without* the permission parameter) is used, the mode of the new file is `666&^umask`. When the new `create(path, `*permission*`, …)` method (*with* the permission parameter *P*) is used, the mode of the new file is `P&^umask&666`. When a new directory is created with the existing `mkdirs(path)` method (*without* the permission parameter), the mode of the new directory is `777&^umask`. When the new `mkdirs(path, `*permission*` )` method (*with* the permission parameter *P*) is used, the mode of new directory is `P&^umask&777`.

5. Changes to the Application Shell

New operations:

`chmod [-R]` ***mode file …***
Only the owner of a file or the super-user is permitted to change the mode of a file.
`chgrp [-R]` ***group file …***
The user invoking `chgrp` must belong to the specified group and be the owner of the file, or be the super-user.
`chown [-R]` ***[owner][:[group]] file …***
The owner of a file may only be altered by a super-user.
`ls` ***file …***
`lsr` ***file …***
The output is reformatted to display the owner, group and mode.

6. The Super-User

The super-user is the user with the same identity as name node process itself. Loosely, if you started the name node, then you are the super-user. The super-user can do anything in that permissions checks never fail for the super-user. There is no persistent notion of who *was* the super-user; when the name node is started the process identity determines who is the super-user *for now*. The HDFS super-user does not have to be the super-user of the name node host, nor is it necessary that all clusters have the same super-user. Also, an experimenter running HDFS on a personal workstation, conveniently becomes that installation's super-user without any configuration.

In addition, the administrator my identify a distinguished group using a configuration parameter. If set, members of this group are also super-users.

Copyright © 2007 The Apache Software Foundation. All rights reserved.

7. The Web Server

The identity of the web server is a configuration parameter. That is, the name node has no notion of the identity of the *real* user, but the web server behaves as if it has the identity (user and groups) of a user chosen by the administrator. Unless the chosen identity matches the super-user, parts of the name space may be invisible to the web server.

8. On-line Upgrade

If a cluster starts with a version 0.15 data set (`fsimage`), all files and directories will have owner *O*, group *G*, and mode *M*, where *O* and *G* are the user and group identity of the super-user, and *M* is a configuration parameter.

9. Configuration Parameters

`dfs.permissions = true`
If `yes` use the permissions system as described here. If `no`, permission *checking* is turned off, but all other behavior is unchanged. Switching from one parameter value to the other does not change the mode, owner or group of files or directories.

Regardless of whether permissions are on or off, `chmod`, `chgrp` and `chown` *always* check permissions. These functions are only useful in the permissions context, and so there is no backwards compatibility issue. Furthermore, this allows administrators to reliably set owners and permissions in advance of turning on regular permissions checking.

`dfs.web.ugi = webuser,webgroup`
The user name to be used by the web server. Setting this to the name of the super-user allows any web client to see everything. Changing this to an otherwise unused identity allows web clients to see only those things visible using "other" permissions. Additional groups may be added to the comma-separated list.

`dfs.permissions.supergroup = supergroup`
The name of the group of super-users.

`dfs.upgrade.permission = 777`
The choice of initial mode during upgrade. The *x* permission is *never* set for files. For configuration files, the decimal value *51110* may be used.

`dfs.umask = 022`
The `umask` used when creating files and directories. For configuration files, the decimal value *1810* may be used.

Copyright © 2007 The Apache Software Foundation. All rights reserved.

Copyright © 2007 The Apache Software Foundation. All rights reserved.
