# Supplementary material for: Provenance based data integrity checking and verification in cloud environments
Source: PLoS One. 2017 May 17;12(5):e0177576. doi: 10.1371/journal.pone.0177576 (PMC5435237; doi:10.1371/journal.pone.0177576)
Supplement: S1 Dataset — Files in the Dataset are utilized for performance measurement. (ZIP) [file pone.0177576.s001.zip › dataset/pdf/hdfs_quota_admin_guide - Copy (2) - Copy.pdf]

Name Space Quotas Administrator Guide

The Hadoop Distributed File System (HDFS) allows the administrator to set quotas on individual directories. Newly created directories have no associated quota. The largest quota is `Long.Max_Value`. A quota of one forces a directory to remain empty.

The directory quota is a hard limit on the number of names in the tree rooted at that directory. File and directory creations fault if the quota would be exceeded. Quotas stick to renamed directories; the rename operation faults if operation would result in a quota violation. The attempt to set a quota faults if the directory would be in violation of the new quota.

Quotas are persistent with the fsimage. When starting, if the fsimage is immediately in violation of a quota (perhaps the fsimage was surreptitiously modified), the startup operation fails with an error report. Setting or removing a quota creates a journal entry.

The following new commands or new options are added to support quotas. The first two are administration commands.

- `dfsadmin -setquota <N> <directory>...<directory>`
  Set the quota to be `N` for each directory. Best effort for each directory, with faults reported if `N` is not a positive long integer, the directory does not exist or it is a file, or the directory would immediately exceed the new quota.
- `dfsadmin -clrquota <directory>...<director>`
  Remove any quota for each directory. Best effort for each directory, with faults reported if the directory does not exist or it is a file. It is not a fault if the directory has no quota.
- `fs -count -q <directory>...<directory>`
  With the `-q` option, also report the quota value set for each directory, and the available quota remaining. If the directory does not have a quota set, the reported values are `none` and `inf`.

Copyright © 2007 The Apache Software Foundation. All rights reserved.
