# Supplementary material for: Provenance based data integrity checking and verification in cloud environments
Source: PLoS One. 2017 May 17;12(5):e0177576. doi: 10.1371/journal.pone.0177576 (PMC5435237; doi:10.1371/journal.pone.0177576)
Supplement: S1 Dataset — Files in the Dataset are utilized for performance measurement. (ZIP) [file pone.0177576.s001.zip › dataset/pdf/hdfs_shell - Copy (2) - Copy.pdf]

Hadoop Shell Commands

Table of contents

1 FS Shell 3
1.1 cat 3
1.2 chgrp 3
1.3 chmod 3
1.4 chown 4
1.5 copyFromLocal........ 4
1.6 copyToLocal........ 4
1.7 cp 4
1.8 du........ 4
1.9 dus 5
1.10 expunge 5
1.11 get 5
1.12 getmerge 5
1.13 ls 5
1.14 lsr........ 6
1.15 mkdir 6
1.16 movefromLocal 6
1.17 mv 6
1.18 put 7
1.19 rm 7
1.20 rmr 8
1.21 setrep 8
1.22 stat 8
1.23 tail 8

Copyright © 2007 The Apache Software Foundation. All rights reserved.

1.24 test 9
1.25 text 9
1.26 touchz 9

Copyright © 2007 The Apache Software Foundation. All rights reserved.

1. FS Shell

The FileSystem (FS) shell is invoked by `bin/hadoop fs <args>`. All the FS shell commands take path URIs as arguments. The URI format is *scheme://autority/path*. For HDFS the scheme is *hdfs*, and for the local filesystem the scheme is *file*. The scheme and authority are optional. If not specified, the default scheme specified in the configuration is used. An HDFS file or directory such as */parent/child* can be specified as *hdfs://namenodehost/parent/child* or simply as */parent/child* (given that your configuration is set to point to *hdfs://namenodehost*). Most of the commands in FS shell behave like corresponding Unix commands. Differences are described with each of the commands. Error information is sent to *stderr* and the output is sent to *stdout*.

1.1. cat

`Usage: hadoop fs -cat URI [URI …]`

Copies source paths to *stdout*.

Example:

- `hadoop fs -cat hdfs://nn1.example.com/file1 hdfs://nn2.example.com/file2`
- `hadoop fs -cat file:///file3 /user/hadoop/file4`

Exit Code:
`Returns 0 on success and -1 on error.`

1.2. chgrp

`Usage: hadoop fs -chgrp [-R] GROUP URI [URI …]`

Change group association of files. With `-R`, make the change recursively through the directory structure. The user must be the owner of files, or else a super-user. Additional information is in the Permissions User Guide.

1.3. chmod

`Usage: hadoop fs -chmod [-R] <MODE[,MODE]... | OCTALMODE> URI [URI …]`

Change the permissions of files. With `-R`, make the change recursively through the directory structure. The user must be the owner of the file, or else a super-user. Additional information is in the Permissions User Guide.

Copyright © 2007 The Apache Software Foundation. All rights reserved.

1.4. chown

`Usage: hadoop fs -chown [-R] [OWNER][:[GROUP]] URI [URI ]`

Change the owner of files. With `-R`, make the change recursively through the directory structure. The user must be a super-user. Additional information is in the Permissions User Guide.

1.5. copyFromLocal

`Usage: hadoop fs -copyFromLocal <localsrc> URI`

Similar to **put** command, except that the source is restricted to a local file reference.

1.6. copyToLocal

`Usage: hadoop fs -copyToLocal [-ignorecrc] [-crc] URI <localdst>`

Similar to **get** command, except that the destination is restricted to a local file reference.

1.7. cp

`Usage: hadoop fs -cp URI [URI …] <dest>`

Copy files from source to destination. This command allows multiple sources as well in which case the destination must be a directory.
Example:

- `hadoop fs -cp /user/hadoop/file1 /user/hadoop/file2`
- `hadoop fs -cp /user/hadoop/file1 /user/hadoop/file2 /user/hadoop/dir`

Exit Code:

`Returns 0 on success and -1 on error.`

1.8. du

`Usage: hadoop fs -du URI [URI …]`

Displays aggregate length of files contained in the directory or the length of a file in case its just a file.
Example:

Copyright © 2007 The Apache Software Foundation. All rights reserved.

```
hadoop fs -du /user/hadoop/dir1 /user/hadoop/file1
hdfs://nn.example.com/user/hadoop/dir1
```

Exit Code:

```
Returns 0 on success and -1 on error.
```

1.9. dus

```
Usage: hadoop fs -dus <args>
```

Displays a summary of file lengths.

1.10. expunge

```
Usage: hadoop fs -expunge
```

Empty the Trash. Refer to HDFS Design for more information on Trash feature.

1.11. get

```
Usage: hadoop fs -get [-ignorecrc] [-crc] <src> <localdst>
```

Copy files to the local file system. Files that fail the CRC check may be copied with the `-ignorecrc` option. Files and CRCs may be copied using the `-crc` option.

Example:

- `hadoop fs -get /user/hadoop/file localfile`
- `hadoop fs -get hdfs://nn.example.com/user/hadoop/file localfile`

Exit Code:

```
Returns 0 on success and -1 on error.
```

1.12. getmerge

```
Usage: hadoop fs -getmerge <src> <localdst> [addnl]
```

Takes a source directory and a destination file as input and concatenates files in src into the destination local file. Optionally `addnl` can be set to enable adding a newline character at the end of each file.

1.13. ls

Copyright © 2007 The Apache Software Foundation. All rights reserved.

```
Usage: hadoop fs -ls <args>
```

For a file returns stat on the file with the following format:
`filename <number of replicas> filesize modification_date modification_time permissions userid groupid`
For a directory it returns list of its direct children as in unix. A directory is listed as:
`dirname <dir> modification_time modification_time permissions userid groupid`
Example:
`hadoop fs -ls /user/hadoop/file1 /user/hadoop/file2 hdfs://nn.example.com/user/hadoop/dir1 /nonexistentfile`
Exit Code:
`Returns 0 on success and -1 on error.`

1.14. lsr

`Usage: hadoop fs -lsr <args>`
Recursive version of `ls`. Similar to Unix `ls -R`.

1.15. mkdir

```
Usage: hadoop fs -mkdir <paths>
```

Takes path uri's as argument and creates directories. The behavior is much like unix mkdir -p creating parent directories along the path.

Example:

- `hadoop fs -mkdir /user/hadoop/dir1 /user/hadoop/dir2`
- `hadoop fs -mkdir hdfs://nn1.example.com/user/hadoop/dir hdfs://nn2.example.com/user/hadoop/dir`

Exit Code:

`Returns 0 on success and -1 on error.`

1.16. movefromLocal

```
Usage: dfs -moveFromLocal <src> <dst>
```

Displays a "not implemented" message.

1.17. mv

Copyright © 2007 The Apache Software Foundation. All rights reserved.

`Usage: hadoop fs -mv URI [URI …] <dest>`

Moves files from source to destination. This command allows multiple sources as well in which case the destination needs to be a directory. Moving files across filesystems is not permitted.
Example:

- `hadoop fs -mv /user/hadoop/file1 /user/hadoop/file2`
- `hadoop fs -mv hdfs://nn.example.com/file1 hdfs://nn.example.com/file2 hdfs://nn.example.com/file3 hdfs://nn.example.com/dir1`

Exit Code:

`Returns 0 on success and -1 on error.`

1.18. put

`Usage: hadoop fs -put <localsrc> ... <dst>`

Copy single src, or multiple srcs from local file system to the destination filesystem. Also reads input from stdin and writes to destination filesystem.

- `hadoop fs -put localfile /user/hadoop/hadoopfile`
- `hadoop fs -put localfile1 localfile2 /user/hadoop/hadoopdir`
- `hadoop fs -put localfile hdfs://nn.example.com/hadoop/hadoopfile`
- `hadoop fs -put - hdfs://nn.example.com/hadoop/hadoopfile` Reads the input from stdin.

Exit Code:

`Returns 0 on success and -1 on error.`

1.19. rm

`Usage: hadoop fs -rm URI [URI …]`

Delete files specified as args. Only deletes non empty directory and files. Refer to rmr for recursive deletes.
Example:

- `hadoop fs -rm hdfs://nn.example.com/file /user/hadoop/emptydir`

Copyright © 2007 The Apache Software Foundation. All rights reserved.

Exit Code:

`Returns 0 on success and -1 on error.`

1.20. rmr

`Usage: hadoop fs -rmr URI [URI …]`

Recursive version of delete.
Example:

- `hadoop fs -rmr /user/hadoop/dir`
- `hadoop fs -rmr hdfs://nn.example.com/user/hadoop/dir`

Exit Code:

`Returns 0 on success and -1 on error.`

1.21. setrep

`Usage: hadoop fs -setrep [-R] <path>`

Changes the replication factor of a file. -R option is for recursively increasing the replication factor of files within a directory.

Example:

- `hadoop fs -setrep -w 3 -R /user/hadoop/dir1`

Exit Code:

`Returns 0 on success and -1 on error.`

1.22. stat

`Usage: hadoop fs -stat URI [URI …]`

Returns the stat information on the path.

Example:

- `hadoop fs -stat path`

Exit Code:
`Returns 0 on success and -1 on error.`

Copyright © 2007 The Apache Software Foundation. All rights reserved.

1.23. tail

`Usage: hadoop fs -tail [-f] URI`

Displays last kilobyte of the file to stdout. -f option can be used as in Unix.

Example:

- `hadoop fs -tail pathname`

Exit Code:
`Returns 0 on success and -1 on error.`

1.24. test

`Usage: hadoop fs -test -[ezd] URI`

Options:
-e check to see if the file exists. Return 0 if true.
-z check to see if the file is zero length. Return 0 if true
-d check return 1 if the path is directory else return 0.

Example:

- `hadoop fs -test -e filename`

1.25. text

`Usage: hadoop fs -text <src>`

Takes a source file and outputs the file in text format. The allowed formats are zip and TextRecordInputStream.

1.26. touchz

`Usage: hadoop fs -touchz URI [URI …]`

Create a file of zero length.

Example:

- `hadoop -touchz pathname`

Exit Code:
`Returns 0 on success and -1 on error.`

Copyright © 2007 The Apache Software Foundation. All rights reserved.

Copyright © 2007 The Apache Software Foundation. All rights reserved.
