# Supplementary material for: Provenance based data integrity checking and verification in cloud environments
Source: PLoS One. 2017 May 17;12(5):e0177576. doi: 10.1371/journal.pone.0177576 (PMC5435237; doi:10.1371/journal.pone.0177576)
Supplement: S1 Dataset — Files in the Dataset are utilized for performance measurement. (ZIP) [file pone.0177576.s001.zip › dataset/pdf/hdfs_user_guide - Copy (2) - Copy.pdf]

HDFS User Guide

Table of contents

1 Purpose..2
2 Overview 2
3 Pre-requisites 3
4 Web Interface 3
5 Shell Commands... 3
5.1 DFSAdmin Command 3
6 Secondary NameNode 4
7 Rebalancer 5
8 Rack Awareness 5
9 Safemode 5
10 fsck 6
11 Upgrade and Rollback 6
12 File Permissions and Security 7
13 Scalability 7
14 Related Documentation 7

Copyright © 2007 The Apache Software Foundation. All rights reserved.

1. Purpose

This document is a starting point for users working with Hadoop Distributed File System (HDFS) either as a part of a Hadoop cluster or as a stand-alone general purpose distributed file system. While HDFS is designed to "just work" in many environments, a working knowledge of HDFS helps greatly with configuration improvements and diagnostics on a specific cluster.

2. Overview

HDFS is the primary distributed storage used by Hadoop applications. A HDFS cluster primarily consists of a NameNode that manages the file system metadata and DataNodes that store the actual data. The architecture of HDFS is described in detail here. This user guide primarily deals with interaction of users and administrators with HDFS clusters. The diagram from HDFS architecture depicts basic interactions among NameNode, the DataNodes, and the clients. Clients contact NameNode for file metadata or file modifications and perform actual file I/O directly with the DataNodes.

The following are some of the salient features that could be of interest to many users.

- Hadoop, including HDFS, is well suited for distributed storage and distributed processing using commodity hardware. It is fault tolerant, scalable, and extremely simple to expand. Map-Reduce, well known for its simplicity and applicability for large set of distributed applications, is an integral part of Hadoop.
- HDFS is highly configurable with a default configuration well suited for many installations. Most of the time, configuration needs to be tuned only for very large clusters.
- Hadoop is written in Java and is supported on all major platforms.
- Hadoop supports shell-like commands to interact with HDFS directly.
- The NameNode and Datanodes have built in web servers that makes it easy to check current status of the cluster.
- New features and improvements are regularly implemented in HDFS. The following is a subset of useful features in HDFS:
  - File permissions and authentication.
  - *Rack awareness*: to take a node's physical location into account while scheduling tasks and allocating storage.
  - Safemode: an administrative mode for maintenance.
  - `fsck`: a utility to diagnose health of the file system, to find missing files or blocks.
  - Rebalancer: tool to balance the cluster when the data is unevenly distributed among DataNodes.
  - Upgrade and rollback: after a software upgrade, it is possible to rollback to HDFS'

Copyright © 2007 The Apache Software Foundation. All rights reserved.

state before the upgrade in case of unexpected problems.
- Secondary NameNode: performs periodic checkpoints of the namespace and helps keep the size of file containing log of HDFS modifications within certain limits at the NameNode.

3. Pre-requisites

The following documents describe installation and set up of a Hadoop cluster :

- Hadoop Quick Start for first-time users.
- Hadoop Cluster Setup for large, distributed clusters.

The rest of this document assumes the user is able to set up and run a HDFS with at least one DataNode. For the purpose of this document, both the NameNode and DataNode could be running on the same physical machine.

4. Web Interface

NameNode and DataNode each run an internal web server in order to display basic information about the current status of the cluster. With the default configuration, the NameNode front page is at `http://namenode-name:50070/`. It lists the DataNodes in the cluster and basic statistics of the cluster. The web interface can also be used to browse the file system (using "Browse the file system" link on the NameNode front page).

5. Shell Commands

Hadoop includes various shell-like commands that directly interact with HDFS and other file systems that Hadoop supports. The command `bin/hadoop fs -help` lists the commands supported by Hadoop shell. Furthermore, the command `bin/hadoop fs -help command-name` displays more detailed help for a command. These commands support most of the normal files ystem operations like copying files, changing file permissions, etc. It also supports a few HDFS specific operations like changing replication of files.

5.1. DFSAdmin Command

The `bin/hadoop dfsadmin` command supports a few HDFS administration related operations. The `bin/hadoop dfsadmin -help` command lists all the commands currently supported. For e.g.:

- `-report` : reports basic statistics of HDFS. Some of this information is also available on the NameNode front page.

Copyright © 2007 The Apache Software Foundation. All rights reserved.

- `-safemode` : though usually not required, an administrator can manually enter or leave Safemode.
- `-finalizeUpgrade` : removes previous backup of the cluster made during last upgrade.

For command usage, see dfsadmin command.

6. Secondary NameNode

The NameNode stores modifications to the file system as a log appended to a native file system file (`edits`). When a NameNode starts up, it reads HDFS state from an image file (`fsimage`) and then applies edits from the edits log file. It then writes new HDFS state to the `fsimage` and starts normal operation with an empty edits file. Since NameNode merges `fsimage` and `edits` files only during start up, the edits log file could get very large over time on a busy cluster. Another side effect of a larger edits file is that next restart of NameNode takes longer.

The secondary NameNode merges the fsimage and the edits log files periodically and keeps edits log size within a limit. It is usually run on a different machine than the primary NameNode since its memory requirements are on the same order as the primary NameNode. The secondary NameNode is started by `bin/start-dfs.sh` on the nodes specified in `conf/masters` file.

The start of the checkpoint process on the secondary NameNode is controlled by two configuration parameters.

- `fs.checkpoint.period`, set to 1 hour by default, specifies the maximum delay between two consecutive checkpoints, and
- `fs.checkpoint.size`, set to 64MB by default, defines the size of the edits log file that forces an urgent checkpoint even if the maximum checkpoint delay is not reached.

The secondary NameNode stores the latest checkpoint in a directory which is structured the same way as the primary NameNode's directory. So that the check pointed image is always ready to be read by the primary NameNode if necessary.

The latest checkpoint can be imported to the primary NameNode if all other copies of the image and the edits files are lost. In order to do that one should:

- Create an empty directory specified in the `dfs.name.dir` configuration variable;
- Specify the location of the checkpoint directory in the configuration variable `fs.checkpoint.dir`;
- and start the NameNode with `-importCheckpoint` option.

The NameNode will upload the checkpoint from the `fs.checkpoint.dir` directory and

Copyright © 2007 The Apache Software Foundation. All rights reserved.

then save it to the NameNode directory(s) set in `dfs.name.dir`. The NameNode will fail if a legal image is contained in `dfs.name.dir`. The NameNode verifies that the image in `fs.checkpoint.dir` is consistent, but does not modify it in any way.

For command usage, see `secondarynamenode` command.

7. Rebalancer

HDFS data might not always be be placed uniformly across the DataNode. One common reason is addition of new DataNodes to an existing cluster. While placing new blocks (data for a file is stored as a series of blocks), NameNode considers various parameters before choosing the DataNodes to receive these blocks. Some of the considerations are:

- Policy to keep one of the replicas of a block on the same node as the node that is writing the block.
- Need to spread different replicas of a block across the racks so that cluster can survive loss of whole rack.
- One of the replicas is usually placed on the same rack as the node writing to the file so that cross-rack network I/O is reduced.
- Spread HDFS data uniformly across the DataNodes in the cluster.

Due to multiple competing considerations, data might not be uniformly placed across the DataNodes. HDFS provides a tool for administrators that analyzes block placement and rebalanaces data across the DataNode. A brief administrator's guide for rebalancer as a PDF is attached to HADOOP-1652.

For command usage, see balancer command.

8. Rack Awareness

Typically large Hadoop clusters are arranged in racks and network traffic between different nodes with in the same rack is much more desirable than network traffic across the racks. In addition NameNode tries to place replicas of block on multiple racks for improved fault tolerance. Hadoop lets the cluster administrators decide which rack a node belongs to through configuration variable `dfs.network.script`. When this script is configured, each node runs the script to determine its rack id. A default installation assumes all the nodes belong to the same rack. This feature and configuration is further described in PDF attached to HADOOP-692.

9. Safemode

During start up the NameNode loads the file system state from the fsimage and the edits log

Copyright © 2007 The Apache Software Foundation. All rights reserved.

file. It then waits for DataNodes to report their blocks so that it does not prematurely start replicating the blocks though enough replicas already exist in the cluster. During this time NameNode stays in Safemode. Safemode for the NameNode is essentially a read-only mode for the HDFS cluster, where it does not allow any modifications to file system or blocks. Normally the NameNode leaves Safemode automatically after the DataNodes have reported that most file system blocks are available. If required, HDFS could be placed in Safemode explicitly using `'bin/hadoop dfsadmin -safemode'` command. NameNode front page shows whether Safemode is on or off. A more detailed description and configuration is maintained as JavaDoc for `setSafeMode()`.

10. fsck

HDFS supports the `fsck` command to check for various inconsistencies. It it is designed for reporting problems with various files, for example, missing blocks for a file or under-replicated blocks. Unlike a traditional `fsck` utility for native file systems, this command does not correct the errors it detects. Normally NameNode automatically corrects most of the recoverable failures. By default `fsck` ignores open files but provides an option to select all files during reporting. The HDFS `fsck` command is not a Hadoop shell command. It can be run as '`bin/hadoop fsck`'. For command usage, see `fsck` command. `fsck` can be run on the whole file system or on a subset of files.

11. Upgrade and Rollback

When Hadoop is upgraded on an existing cluster, as with any software upgrade, it is possible there are new bugs or incompatible changes that affect existing applications and were not discovered earlier. In any non-trivial HDFS installation, it is not an option to loose any data, let alone to restart HDFS from scratch. HDFS allows administrators to go back to earlier version of Hadoop and rollback the cluster to the state it was in before the upgrade. HDFS upgrade is described in more detail in upgrade wiki. HDFS can have one such backup at a time. Before upgrading, administrators need to remove existing backup using `bin/hadoop dfsadmin -finalizeUpgrade` command. The following briefly describes the typical upgrade procedure:

- Before upgrading Hadoop software, *finalize* if there an existing backup. `dfsadmin -upgradeProgress status` can tell if the cluster needs to be *finalized*.
- Stop the cluster and distribute new version of Hadoop.
- Run the new version with `-upgrade` option (`bin/start-dfs.sh -upgrade`).
- Most of the time, cluster works just fine. Once the new HDFS is considered working well (may be after a few days of operation), finalize the upgrade. Note that until the cluster is finalized, deleting the files that existed before the upgrade does not free up real disk space on the DataNodes.

Copyright © 2007 The Apache Software Foundation. All rights reserved.

- If there is a need to move back to the old version,
  - stop the cluster and distribute earlier version of Hadoop.
  - start the cluster with rollback option. (`bin/start-dfs.h -rollback`).

12. File Permissions and Security

The file permissions are designed to be similar to file permissions on other familiar platforms like Linux. Currently, security is limited to simple file permissions. The user that starts NameNode is treated as the superuser for HDFS. Future versions of HDFS will support network authentication protocols like Kerberos for user authentication and encryption of data transfers. The details are discussed in the Permissions User and Administrator Guide.

13. Scalability

Hadoop currently runs on clusters with thousands of nodes. Powered By Hadoop lists some of the organizations that deploy Hadoop on large clusters. HDFS has one NameNode for each cluster. Currently the total memory available on NameNode is the primary scalability limitation. On very large clusters, increasing average size of files stored in HDFS helps with increasing cluster size without increasing memory requirements on NameNode. The default configuration may not suite very large clustes. Hadoop FAQ page lists suggested configuration improvements for large Hadoop clusters.

14. Related Documentation

This user guide is a good starting point for working with HDFS. While the user guide continues to improve, there is a large wealth of documentation about Hadoop and HDFS. The following list is a starting point for further exploration:

- Hadoop Home Page: The start page for everything Hadoop.
- Hadoop Wiki : Front page for Hadoop Wiki documentation. Unlike this guide which is part of Hadoop source tree, Hadoop Wiki is regularly edited by Hadoop Community.
- FAQ from Hadoop Wiki.
- Hadoop JavaDoc API.
- Hadoop User Mailing List : core-user[at]hadoop.apache.org.
- Explore `conf/hadoop-default.xml`. It includes brief description of most of the configuration variables available.
- Commands Manual : commands usage.

Copyright © 2007 The Apache Software Foundation. All rights reserved.
