# Supplementary material for: Provenance based data integrity checking and verification in cloud environments
Source: PLoS One. 2017 May 17;12(5):e0177576. doi: 10.1371/journal.pone.0177576 (PMC5435237; doi:10.1371/journal.pone.0177576)
Supplement: S1 Dataset — Files in the Dataset are utilized for performance measurement. (ZIP) [file pone.0177576.s001.zip › dataset/pdf/hod - Copy (2) - Copy.pdf]

Hadoop On Demand

Table of contents

1 Introduction 2
2 Documentation.. 2

Copyright © 2007 The Apache Software Foundation. All rights reserved.

1. Introduction

Hadoop On Demand (HOD) is a system for provisioning virtual Hadoop clusters over a large physical cluster. It uses the Torque resource manager to do node allocation. On the allocated nodes, it can start Hadoop Map/Reduce and HDFS daemons. It automatically generates the appropriate configuration files (hadoop-site.xml) for the Hadoop daemons and client. HOD also has the capability to distribute Hadoop to the nodes in the virtual cluster that it allocates. In short, HOD makes it easy for administrators and users to quickly setup and use Hadoop. It is also a very useful tool for Hadoop developers and testers who need to share a physical cluster for testing their own Hadoop versions.

2. Documentation

Please go through the following to know more about using HOD

- HOD Admin Guide : This guide provides an overview of the HOD architecture, Torque resource manager, and various support tools and utilities, and shows you how to install, configure, and run HOD.
- Hod Configuration Guide : This guide discusses HOD configuration sections and shows you how to work with the most important and commonly used configuration options.
- Hod User Guide : This guide shows you how to get started using HOD, reviews various HOD features and command line options, and provides detailed troubleshooting help.

Copyright © 2007 The Apache Software Foundation. All rights reserved.
