# Supplementary material for: Provenance based data integrity checking and verification in cloud environments
Source: PLoS One. 2017 May 17;12(5):e0177576. doi: 10.1371/journal.pone.0177576 (PMC5435237; doi:10.1371/journal.pone.0177576)
Supplement: S1 Dataset — Files in the Dataset are utilized for performance measurement. (ZIP) [file pone.0177576.s001.zip › dataset/pdf/hod_admin_guide - Copy (2) - Copy.pdf]

Hadoop On Demand

Table of contents

1 Overview..2
2 Pre-requisites..3
3 Resource Manager..3
4 Installing HOD...4
5 Configuring HOD... 4
5.1 Minimal Configuration...4
5.2 Advanced Configuration... 5
6 Running HOD.. 5
7 Supporting Tools and Utilities..5
7.1 logcondense.py - Manage Log Files..5
7.2 checklimits.sh - Monitor Resource Limits.. 7

Copyright © 2007 The Apache Software Foundation. All rights reserved.

1. Overview

The Hadoop On Demand (HOD) project is a system for provisioning and managing independent Hadoop Map/Reduce and Hadoop Distributed File System (HDFS) instances on a shared cluster of nodes. HOD is a tool that makes it easy for administrators and users to quickly setup and use Hadoop. It is also a very useful tool for Hadoop developers and testers who need to share a physical cluster for testing their own Hadoop versions.

HOD relies on a resource manager (RM) for allocation of nodes that it can use for running Hadoop instances. At present it runs with the Torque resource manager.

The basic system architecture of HOD includes these components:

- A Resource manager (possibly together with a scheduler)
- Various HOD components
- Hadoop Map/Reduce and HDFS daemons

HOD provisions and maintains Hadoop Map/Reduce and, optionally, HDFS instances through interaction with the above components on a given cluster of nodes. A cluster of nodes can be thought of as comprising two sets of nodes:

- Submit nodes: Users use the HOD client on these nodes to allocate clusters, and then use the Hadoop client to submit Hadoop jobs.
- Compute nodes: Using the resource manager, HOD components are run on these nodes to provision the Hadoop daemons. After that Hadoop jobs run on them.

Here is a brief description of the sequence of operations in allocating a cluster and running jobs on them.

- The user uses the HOD client on the Submit node to allocate a desired number of cluster nodes and to provision Hadoop on them.
- The HOD client uses a resource manager interface (qsub, in Torque) to submit a HOD process, called the RingMaster, as a Resource Manager job, to request the user's desired number of nodes. This job is submitted to the central server of the resource manager (pbs_server, in Torque).
- On the compute nodes, the resource manager slave daemons (pbs_moms in Torque) accept and run jobs that they are assigned by the central server (pbs_server in Torque). The RingMaster process is started on one of the compute nodes (mother superior, in Torque).
- The RingMaster then uses another resource manager interface (pbsdsh, in Torque) to run the second HOD component, HodRing, as distributed tasks on each of the compute nodes allocated.
- The HodRings, after initializing, communicate with the RingMaster to get Hadoop

Copyright © 2007 The Apache Software Foundation. All rights reserved.

commands, and run them accordingly. Once the Hadoop commands are started, they register with the RingMaster, giving information about the daemons.
- All the configuration files needed for Hadoop instances are generated by HOD itself, some obtained from options given by user in its own configuration file.
- The HOD client keeps communicating with the RingMaster to find out the location of the JobTracker and HDFS daemons.

The rest of this document describes how to setup HOD on a physical cluster of nodes.

2. Pre-requisites

To use HOD, your system should include the following hardware and software components.

Operating System: HOD is currently tested on RHEL4.
Nodes : HOD requires a minimum of three nodes configured through a resource manager.

Software

The following components must be installed on ALL nodes before using HOD:

- Torque: Resource manager
- Python : HOD requires version 2.5.1 of Python.

The following components are optional and can be installed to obtain better functionality from HOD:

- Twisted Python: This can be used for improving the scalability of HOD. If this module is detected to be installed, HOD uses it, else it falls back to default modules.
- Hadoop: HOD can automatically distribute Hadoop to all nodes in the cluster. However, it can also use a pre-installed version of Hadoop, if it is available on all nodes in the cluster. HOD currently supports Hadoop 0.15 and above.

NOTE: HOD configuration requires the location of installs of these components to be the same on all nodes in the cluster. It will also make the configuration simpler to have the same location on the submit nodes.

3. Resource Manager

Currently HOD works with the Torque resource manager, which it uses for its node allocation and job submission. Torque is an open source resource manager from Cluster Resources, a community effort based on the PBS project. It provides control over batch jobs and distributed compute nodes. Torque is freely available for download from here.

All documentation related to torque can be seen under the section TORQUE Resource

Copyright © 2007 The Apache Software Foundation. All rights reserved.

Manager here. You can get wiki documentation from here. Users may wish to subscribe to TORQUE's mailing list or view the archive for questions, comments here.

To use HOD with Torque:

- Install Torque components: pbs_server on one node (head node), pbs_mom on all compute nodes, and PBS client tools on all compute nodes and submit nodes. Perform at least a basic configuration so that the Torque system is up and running, that is, pbs_server knows which machines to talk to. Look here for basic configuration. For advanced configuration, see here
- Create a queue for submitting jobs on the pbs_server. The name of the queue is the same as the HOD configuration parameter, resource-manager.queue. The HOD client uses this queue to submit the RingMaster process as a Torque job.
- Specify a cluster name as a property for all nodes in the cluster. This can be done by using the qmgr command. For example: `qmgr -c "set node node properties=cluster-name"`. The name of the cluster is the same as the HOD configuration parameter, hod.cluster.
- Make sure that jobs can be submitted to the nodes. This can be done by using the qsub command. For example: `echo "sleep 30" | qsub -l nodes=3`

4. Installing HOD

Once the resource manager is set up, you can obtain and install HOD.

- If you are getting HOD from the Hadoop tarball, it is available under the 'contrib' section of Hadoop, under the root directory 'hod'.
- If you are building from source, you can run ant tar from the Hadoop root directory to generate the Hadoop tarball, and then get HOD from there, as described above.
- Distribute the files under this directory to all the nodes in the cluster. Note that the location where the files are copied should be the same on all the nodes.
- Note that compiling hadoop would build HOD with appropriate permissions set on all the required script files in HOD.

5. Configuring HOD

You can configure HOD once it is installed. The minimal configuration needed to run HOD is described below. More advanced configuration options are discussed in the HOD Configuration Guide.

5.1. Minimal Configuration

To get started using HOD, the following minimal configuration is required:

Copyright © 2007 The Apache Software Foundation. All rights reserved.

- On the node from where you want to run HOD, edit the file hodrc located in the <install dir>/conf directory. This file contains the minimal set of values required to run hod.
- Specify values suitable to your environment for the following variables defined in the configuration file. Note that some of these variables are defined at more than one place in the file.
  - ${JAVA_HOME}: Location of Java for Hadoop. Hadoop supports Sun JDK 1.5.x and above.
  - ${CLUSTER_NAME}: Name of the cluster which is specified in the 'node property' as mentioned in resource manager configuration.
  - ${HADOOP_HOME}: Location of Hadoop installation on the compute and submit nodes.
  - ${RM_QUEUE}: Queue configured for submitting jobs in the resource manager configuration.
  - ${RM_HOME}: Location of the resource manager installation on the compute and submit nodes.
- The following environment variables may need to be set depending on your environment. These variables must be defined where you run the HOD client and must also be specified in the HOD configuration file as the value of the key resource_manager.env-vars. Multiple variables can be specified as a comma separated list of key=value pairs.
  - HOD_PYTHON_HOME: If you install python to a non-default location of the compute nodes, or submit nodes, then this variable must be defined to point to the python executable in the non-standard location.

5.2. Advanced Configuration

You can review and modify other configuration options to suit your specific needs. Refer to the Configuration Guide for more information.

6. Running HOD

You can run HOD once it is configured. Refer to the HOD User Guide for more information.

7. Supporting Tools and Utilities

This section describes supporting tools and utilities that can be used to manage HOD deployments.

7.1. logcondense.py - Manage Log Files

Copyright © 2007 The Apache Software Foundation. All rights reserved.

As mentioned in the HOD User Guide, HOD can be configured to upload Hadoop logs to a statically configured HDFS. Over time, the number of logs uploaded to HDFS could increase. logcondense.py is a tool that helps administrators to remove log files uploaded to HDFS.

7.1.1. Running logcondense.py

logcondense.py is available under hod_install_location/support folder. You can either run it using python, for example, *python logcondense.py*, or give execute permissions to the file, and directly run it as *logcondense.py*. logcondense.py needs to be run by a user who has sufficient permissions to remove files from locations where log files are uploaded in the HDFS, if permissions are enabled. For example as mentioned in the configuration guide, the logs could be configured to come under the user's home directory in HDFS. In that case, the user running logcondense.py should have super user privileges to remove the files from under all user home directories.

7.1.2. Command Line Options for logcondense.py

The following command line options are supported for logcondense.py.

Short Option	Long option	Meaning	Example
-p	--package	Complete path to the hadoop script. The version of hadoop must be the same as the one running HDFS.	/usr/bin/hadoop
-d	--days	Delete log files older than the specified number of days	7
-c	--config	Path to the Hadoop configuration directory, under which hadoop-site.xml resides. The hadoop-site.xml must point to the HDFS NameNode from where logs are to be removed.	/home/foo/hadoop/conf
-l	--logs	A HDFS path, this must be the same HDFS path as	/user

Copyright © 2007 The Apache Software Foundation. All rights reserved.

		specified for the log-destination-uri, as mentioned in the configuration guide, without the hdfs:// URI string	
-n	--dynamicdfs	If true, this will indicate that the logcondense.py script should delete HDFS logs in addition to Map/Reduce logs. Otherwise, it only deletes Map/Reduce logs, which is also the default if this option is not specified. This option is useful if dynamic HDFS installations are being provisioned by HOD, and the static HDFS installation is being used only to collect logs - a scenario that may be common in test clusters.	false

So, for example, to delete all log files older than 7 days using a hadoop-site.xml stored in ~/hadoop-conf, using the hadoop installation under ~/hadoop-0.17.0, you could say:

python logcondense.py -p ~/hadoop-0.17.0/bin/hadoop -d 7 -c ~/hadoop-conf -l /user

7.2. checklimits.sh - Monitor Resource Limits

checklimits.sh is a HOD tool specific to the Torque/Maui environment (Maui Cluster Scheduler is an open source job scheduler for clusters and supercomputers, from clusterresources). The checklimits.sh script updates the torque comment field when newly submitted job(s) violate or exceed over user limits set up in Maui scheduler. It uses qstat, does one pass over the torque job-list to determine queued or unfinished jobs, runs Maui tool checkjob on each job to see if user limits are violated and then runs torque's qalter utility to update job attribute 'comment'. Currently it updates the comment as *User-limits exceeded. Requested:([0-9]*) Used:([0-9]*) MaxLimit:([0-9]*)* for those jobs that violate limits. This comment field is then used by HOD to behave accordingly depending on the type of

Copyright © 2007 The Apache Software Foundation. All rights reserved.

violation.

7.2.1. Running checklimits.sh

checklimits.sh is available under the hod_install_location/support folder. This shell script can be run directly as *sh checklimits.sh* or as *./checklimits.sh* after enabling execute permissions. Torque and Maui binaries should be available on the machine where the tool is run and should be in the path of the shell script process. To update the comment field of jobs from different users, this tool must be run with torque administrative privileges. This tool must be run repeatedly after specific intervals of time to frequently update jobs violating constraints, for example via cron. Please note that the resource manager and scheduler commands used in this script can be expensive and so it is better not to run this inside a tight loop without sleeping.

Copyright © 2007 The Apache Software Foundation. All rights reserved.
