# Supplementary material for: Provenance based data integrity checking and verification in cloud environments
Source: PLoS One. 2017 May 17;12(5):e0177576. doi: 10.1371/journal.pone.0177576 (PMC5435237; doi:10.1371/journal.pone.0177576)
Supplement: S1 Dataset — Files in the Dataset are utilized for performance measurement. (ZIP) [file pone.0177576.s001.zip › dataset/pdf/hod_config_guide - Copy (2) - Copy.pdf]

Hadoop On Demand: Configuration Guide

Table of contents

1 1. Introduction..2
2 2. Sections...2
3 3. HOD Configuration Options...2
3.1 3.1 Common configuration options...2
3.2 3.2 hod options...3
3.3 3.3 resource_manager options..3
3.4 3.4 ringmaster options..3
3.5 3.5 gridservice-hdfs options...4
3.6 3.6 gridservice-mapred options...4
3.7 3.7 hodring options...5

Copyright © 2007 The Apache Software Foundation. All rights reserved.

1. 1. Introduction

This document explains some of the most important and commonly used Hadoop On Demand (HOD) configuration options. Configuration options can be specified in two ways: a configuration file in the INI format, and as command line options to the HOD shell, specified in the format --section.option[=value]. If the same option is specified in both places, the value specified on the command line overrides the value in the configuration file.

To get a simple description of all configuration options, type:

```
$ hod --verbose-help
```

2. 2. Sections

HOD organizes configuration options into these sections:

- hod: Options for the HOD client
- resource_manager: Options for specifying which resource manager to use, and other parameters for using that resource manager
- ringmaster: Options for the RingMaster process,
- hodring: Options for the HodRing processes
- gridservice-mapred: Options for the Map/Reduce daemons
- gridservice-hdfs: Options for the HDFS daemons.

3. 3. HOD Configuration Options

The following section describes configuration options common to most HOD sections followed by sections that describe configuration options specific to each HOD section.

3.1. 3.1 Common configuration options

Certain configuration options are defined in most of the sections of the HOD configuration. Options defined in a section, are used by the process for which that section applies. These options have the same meaning, but can have different values in each section.

- temp-dir: Temporary directory for usage by the HOD processes. Make sure that the users who will run hod have rights to create directories under the directory specified here.
- debug: Numeric value from 1-4. 4 produces the most log information, and 1 the least.
- log-dir: Directory where log files are stored. By default, this is <install-location>/logs/. The restrictions and notes for the temp-dir variable apply here too.
- xrs-port-range: Range of ports, among which an available port shall be picked for use to run an XML-RPC server.

Copyright © 2007 The Apache Software Foundation. All rights reserved.

- http-port-range: Range of ports, among which an available port shall be picked for use to run an HTTP server.
- java-home: Location of Java to be used by Hadoop.
- syslog-address: Address to which a syslog daemon is bound to. The format of the value is host:port. If configured, HOD log messages will be logged to syslog using this value.

3.2. 3.2 hod options

- cluster: Descriptive name given to the cluster. For Torque, this is specified as a 'Node property' for every node in the cluster. HOD uses this value to compute the number of available nodes.
- client-params: Comma-separated list of hadoop config parameters specified as key-value pairs. These will be used to generate a hadoop-site.xml on the submit node that should be used for running Map/Reduce jobs.
- job-feasibility-attr: Regular expression string that specifies whether and how to check job feasibility - resource manager or scheduler limits. The current implementation corresponds to the torque job attribute 'comment' and by default is disabled. When set, HOD uses it to decide what type of limit violation is triggered and either deallocates the cluster or stays in queued state according as the request is beyond maximum limits or the cumulative usage has crossed maximum limits. The torque comment attribute may be updated periodically by an external mechanism. For example, comment attribute can be updated by running checklimits.sh script in hod/support directory, and then setting job-feasibility-attr equal to the value TORQUE_USER_LIMITS_COMMENT_FIELD, "User-limits exceeded. Requested:([0-9]*) Used:([0-9]*) MaxLimit:([0-9]*)", will make HOD behave accordingly.

3.3. 3.3 resource_manager options

- queue: Name of the queue configured in the resource manager to which jobs are to be submitted.
- batch-home: Install directory to which 'bin' is appended and under which the executables of the resource manager can be found.
- env-vars: Comma-separated list of key-value pairs, expressed as key=value, which would be passed to the jobs launched on the compute nodes. For example, if the python installation is in a non-standard location, one can set the environment variable 'HOD_PYTHON_HOME' to the path to the python executable. The HOD processes launched on the compute nodes can then use this variable.

3.4. 3.4 ringmaster options

- work-dirs: Comma-separated list of paths that will serve as the root for directories that

Copyright © 2007 The Apache Software Foundation. All rights reserved.

HOD generates and passes to Hadoop for use to store DFS and Map/Reduce data. For e.g. this is where DFS data blocks will be stored. Typically, as many paths are specified as there are disks available to ensure all disks are being utilized. The restrictions and notes for the temp-dir variable apply here too.
- max-master-failures: Number of times a hadoop master daemon can fail to launch, beyond which HOD will fail the cluster allocation altogether. In HOD clusters, sometimes there might be a single or few "bad" nodes due to issues like missing java, missing or incorrect version of Hadoop etc. When this configuration variable is set to a positive integer, the RingMaster returns an error to the client only when the number of times a hadoop master (JobTracker or NameNode) fails to start on these bad nodes because of above issues, exceeds the specified value. If the number is not exceeded, the next HodRing which requests for a command to launch is given the same hadoop master again. This way, HOD tries its best for a successful allocation even in the presence of a few bad nodes in the cluster.

3.5. 3.5 gridservice-hdfs options

- external: If false, indicates that a HDFS cluster must be bought up by the HOD system, on the nodes which it allocates via the allocate command. Note that in that case, when the cluster is de-allocated, it will bring down the HDFS cluster, and all the data will be lost. If true, it will try and connect to an externally configured HDFS system. Typically, because input for jobs are placed into HDFS before jobs are run, and also the output from jobs in HDFS is required to be persistent, an internal HDFS cluster is of little value in a production system. However, it allows for quick testing.
- host: Hostname of the externally configured NameNode, if any
- fs_port: Port to which NameNode RPC server is bound.
- info_port: Port to which the NameNode web UI server is bound.
- pkgs: Installation directory, under which bin/hadoop executable is located. This can be used to use a pre-installed version of Hadoop on the cluster.
- server-params: Comma-separated list of hadoop config parameters specified key-value pairs. These will be used to generate a hadoop-site.xml that will be used by the NameNode and DataNodes.
- final-server-params: Same as above, except they will be marked final.

3.6. 3.6 gridservice-mapred options

- external: If false, indicates that a Map/Reduce cluster must be bought up by the HOD system on the nodes which it allocates via the allocate command. If true, if will try and connect to an externally configured Map/Reduce system.
- host: Hostname of the externally configured JobTracker, if any
- tracker_port: Port to which the JobTracker RPC server is bound

Copyright © 2007 The Apache Software Foundation. All rights reserved.

- info_port: Port to which the JobTracker web UI server is bound.
- pkgs: Installation directory, under which bin/hadoop executable is located
- server-params: Comma-separated list of hadoop config parameters specified key-value pairs. These will be used to generate a hadoop-site.xml that will be used by the JobTracker and TaskTrackers
- final-server-params: Same as above, except they will be marked final.

3.7. 3.7 hodring options

- mapred-system-dir-root: Directory in the DFS under which HOD will generate sub-directory names and pass the full path as the value of the 'mapred.system.dir' configuration parameter to Hadoop daemons. The format of the full path will be value-of-this-option/userid/mapredsystem/cluster-id. Note that the directory specified here should be such that all users can create directories under this, if permissions are enabled in HDFS. Setting the value of this option to /user will make HOD use the user's home directory to generate the mapred.system.dir value.
- log-destination-uri: URL describing a path in an external, static DFS or the cluster node's local file system where HOD will upload Hadoop logs when a cluster is deallocated. To specify a DFS path, use the format 'hdfs://path'. To specify a cluster node's local file path, use the format 'file://path'. When clusters are deallocated by HOD, the hadoop logs will be deleted as part of HOD's cleanup process. To ensure these logs persist, you can use this configuration option. The format of the path is value-of-this-option/userid/hod-logs/cluster-id Note that the directory you specify here must be such that all users can create sub-directories under this. Setting this value to hdfs://user will make the logs come in the user's home directory in DFS.
- pkgs: Installation directory, under which bin/hadoop executable is located. This will be used by HOD to upload logs if a HDFS URL is specified in log-destination-uri option. Note that this is useful if the users are using a tarball whose version may differ from the external, static HDFS version.

Copyright © 2007 The Apache Software Foundation. All rights reserved.
