# Supplementary material for: Provenance based data integrity checking and verification in cloud environments
Source: PLoS One. 2017 May 17;12(5):e0177576. doi: 10.1371/journal.pone.0177576 (PMC5435237; doi:10.1371/journal.pone.0177576)
Supplement: S1 Dataset — Files in the Dataset are utilized for performance measurement. (ZIP) [file pone.0177576.s001.zip › dataset/pdf/hod_user_guide - Copy (2) - Copy.pdf]

Hadoop On Demand User Guide

Table of contents

1 Introduction 3
2 Getting Started Using HOD 3
2.1 A typical HOD session.......... 3
2.2 Running hadoop scripts using HOD.......... 5
3 HOD Features 6
3.1 Provisioning and Managing Hadoop Clusters 6
3.2 Using a tarball to distribute Hadoop 6
3.3 Using an external HDFS 7
3.4 Options for Configuring Hadoop 8
3.5 Viewing Hadoop Web-UIs 9
3.6 Collecting and Viewing Hadoop Logs 9
3.7 Auto-deallocation of Idle Clusters 10
3.8 Specifying Additional Job Attributes 10
3.9 Capturing HOD exit codes in Torque 11
3.10 Command Line.......... 12
3.11 Options Configuring HOD 13
4 Troubleshooting 15
4.1 hod Hangs During Allocation 15
4.2 hod Hangs During Deallocation 16
4.3 hod Fails With an error code and error message 16
4.4 Hadoop Jobs Not Running on a Successfully Allocated Cluster 20
4.5 My Hadoop Job Got Killed 21
4.6 Hadoop Job Fails with Message: 'Job tracker still initializing' 21
4.7 The Exit Codes For HOD Are Not Getting Into Torque 22

Copyright © 2007 The Apache Software Foundation. All rights reserved.

4.8 The Hadoop Logs are Not Uploaded to DFS 22
4.9 Locating Ringmaster Logs 22
4.10 Locating Hodring Logs 22

Copyright © 2007 The Apache Software Foundation. All rights reserved.

1. Introduction

Hadoop On Demand (HOD) is a system for provisioning virtual Hadoop clusters over a large physical cluster. It uses the Torque resource manager to do node allocation. On the allocated nodes, it can start Hadoop Map/Reduce and HDFS daemons. It automatically generates the appropriate configuration files (hadoop-site.xml) for the Hadoop daemons and client. HOD also has the capability to distribute Hadoop to the nodes in the virtual cluster that it allocates. In short, HOD makes it easy for administrators and users to quickly setup and use Hadoop. It is also a very useful tool for Hadoop developers and testers who need to share a physical cluster for testing their own Hadoop versions.

HOD supports Hadoop from version 0.15 onwards.

The rest of this document comprises of a quick-start guide that helps you get quickly started with using HOD, a more detailed guide of all HOD features, and a trouble-shooting section.

2. Getting Started Using HOD

In this section, we shall see a step-by-step introduction on how to use HOD for the most basic operations. Before following these steps, it is assumed that HOD and its dependent hardware and software components are setup and configured correctly. This is a step that is generally performed by system administrators of the cluster.

The HOD user interface is a command line utility called `hod`. It is driven by a configuration file, that is typically setup for users by system administrators. Users can override this configuration when using the `hod`, which is described later in this documentation. The configuration file can be specified in two ways when using `hod`, as described below:

- Specify it on command line, using the -c option. Such as `hod <operation> <required-args> -c path-to-the-configuration-file [other-options]`
- Set up an environment variable *HOD_CONF_DIR* where `hod` will be run. This should be pointed to a directory on the local file system, containing a file called *hodrc*. Note that this is analogous to the *HADOOP_CONF_DIR* and *hadoop-site.xml* file for Hadoop. If no configuration file is specified on the command line, `hod` shall look for the *HOD_CONF_DIR* environment variable and a *hodrc* file under that.

In examples listed below, we shall not explicitly point to the configuration option, assuming it is correctly specified.

2.1. A typical HOD session

Copyright © 2007 The Apache Software Foundation. All rights reserved.

A typical session of HOD will involve at least three steps: allocate, run hadoop jobs, deallocate. In order to do this, perform the following steps.

Create a Cluster Directory

The *cluster directory* is a directory on the local file system where `hod` will generate the Hadoop configuration, *hadoop-site.xml*, corresponding to the cluster it allocates. Pass this directory to the `hod` operations as stated below. If the cluster directory passed doesn't already exist, HOD will automatically try to create it and use it. Once a cluster is allocated, a user can utilize it to run Hadoop jobs by specifying the cluster directory as the Hadoop --config option.

Operation *allocate*

The *allocate* operation is used to allocate a set of nodes and install and provision Hadoop on them. It has the following syntax. Note that it requires a cluster_dir (-d, --hod.clusterdir) and the number of nodes (-n, --hod.nodecount) needed to be allocated:

```
$ hod allocate -d cluster_dir -n number_of_nodes [OPTIONS]
```

If the command completes successfully, then `cluster_dir/hadoop-site.xml` will be generated and will contain information about the allocated cluster. It will also print out the information about the Hadoop web UIs.

An example run of this command produces the following output. Note in this example that `~/hod-clusters/test` is the cluster directory, and we are allocating 5 nodes:

```
$ hod allocate -d ~/hod-clusters/test -n 5
INFO - HDFS UI on http://foo1.bar.com:53422
INFO - Mapred UI on http://foo2.bar.com:55380
```

Running Hadoop jobs using the allocated cluster

Now, one can run Hadoop jobs using the allocated cluster in the usual manner. This assumes variables like *JAVA_HOME* and path to the Hadoop installation are set up correctly.:

```
$ hadoop --config cluster_dir hadoop_command hadoop_command_args
```

or

```
$ export HADOOP_CONF_DIR=cluster_dir
$ hadoop hadoop_command hadoop_command_args
```

Continuing our example, the following command will run a wordcount example on the allocated cluster:

```
$ hadoop --config ~/hod-clusters/test jar
```


Copyright © 2007 The Apache Software Foundation. All rights reserved.

```
/path/to/hadoop/hadoop-examples.jar wordcount /path/to/input
/path/to/output
```

or

```
$ export HADOOP_CONF_DIR=~/hod-clusters/test
$ hadoop jar /path/to/hadoop/hadoop-examples.jar wordcount /path/to/input
/path/to/output
```

Operation *deallocate*

The *deallocate* operation is used to release an allocated cluster. When finished with a cluster, deallocate must be run so that the nodes become free for others to use. The *deallocate* operation has the following syntax. Note that it requires the cluster_dir (-d, --hod.clusterdir) argument:

```
$ hod deallocate -d cluster_dir
```

Continuing our example, the following command will deallocate the cluster:

```
$ hod deallocate -d ~/hod-clusters/test
```

As can be seen, HOD allows the users to allocate a cluster, and use it flexibly for running Hadoop jobs. For example, users can run multiple jobs in parallel on the same cluster, by running hadoop from multiple shells pointing to the same configuration.

2.2. Running hadoop scripts using HOD

The HOD *script operation* combines the operations of allocating, using and deallocating a cluster into a single operation. This is very useful for users who want to run a script of hadoop jobs and let HOD handle the cleanup automatically once the script completes. In order to run hadoop scripts using `hod`, do the following:

Create a script file

This will be a regular shell script that will typically contain hadoop commands, such as:

```
$ hadoop jar jar_file options
```

However, the user can add any valid commands as part of the script. HOD will execute this script setting *HADOOP_CONF_DIR* automatically to point to the allocated cluster. So users do not need to worry about this. The users however need to specify a cluster directory just like when using the allocate operation.

Running the script

Copyright © 2007 The Apache Software Foundation. All rights reserved.

The syntax for the *script operation* as is as follows. Note that it requires a cluster directory (-d, --hod.clusterdir), number of nodes (-n, --hod.nodecount) and a script file (-s, --hod.script):

```
$ hod script -d cluster_directory -n number_of_nodes -s script_file
```

Note that HOD will deallocate the cluster as soon as the script completes, and this means that the script must not complete until the hadoop jobs themselves are completed. Users must take care of this while writing the script.

3. HOD Features

3.1. Provisioning and Managing Hadoop Clusters

The primary feature of HOD is to provision Hadoop Map/Reduce and HDFS clusters. This is described above in the Getting Started section. Also, as long as nodes are available, and organizational policies allow, a user can use HOD to allocate multiple Map/Reduce clusters simultaneously. The user would need to specify different paths for the `cluster_dir` parameter mentioned above for each cluster he/she allocates. HOD provides the *list* and the *info* operations to enable managing multiple clusters.

Operation *list*

The list operation lists all the clusters allocated so far by a user. The cluster directory where the hadoop-site.xml is stored for the cluster, and its status vis-a-vis connectivity with the JobTracker and/or HDFS is shown. The list operation has the following syntax:

```
$ hod list
```

Operation *info*

The info operation shows information about a given cluster. The information shown includes the Torque job id, and locations of the important daemons like the HOD Ringmaster process, and the Hadoop JobTracker and NameNode daemons. The info operation has the following syntax. Note that it requires a cluster directory (-d, --hod.clusterdir):

```
$ hod info -d cluster_dir
```

The `cluster_dir` should be a valid cluster directory specified in an earlier *allocate* operation.

3.2. Using a tarball to distribute Hadoop

When provisioning Hadoop, HOD can use either a pre-installed Hadoop on the cluster nodes or distribute and install a Hadoop tarball as part of the provisioning operation. If the tarball

Copyright © 2007 The Apache Software Foundation. All rights reserved.

option is being used, there is no need to have a pre-installed Hadoop on the cluster nodes, nor a need to use a pre-installed one. This is especially useful in a development / QE environment where individual developers may have different versions of Hadoop to test on a shared cluster.

In order to use a pre-installed Hadoop, you must specify, in the hodrc, the `pkgs` option in the `gridservice-hdfs` and `gridservice-mapred` sections. This must point to the path where Hadoop is installed on all nodes of the cluster.

The syntax for specifying tarball is as follows:

```
$ hod allocate -d cluster_dir -n number_of_nodes -t
hadoop_tarball_location
```

For example, the following command allocates Hadoop provided by the tarball `~/share/hadoop.tar.gz`:

```
$ hod allocate -d ~/hadoop-cluster -n 10 -t ~/share/hadoop.tar.gz
```

Similarly, when using hod script, the syntax is as follows:

```
$ hod script -d cluster_directory -s script_file -n number_of_nodes -t
hadoop_tarball_location
```

The hadoop_tarball specified in the syntax above should point to a path on a shared file system that is accessible from all the compute nodes. Currently, HOD only supports NFS mounted file systems.

Note:

- For better distribution performance it is recommended that the Hadoop tarball contain only the libraries and binaries, and not the source or documentation.
- When you want to run jobs against a cluster allocated using the tarball, you must use a compatible version of hadoop to submit your jobs. The best would be to untar and use the version that is present in the tarball itself.
- You need to make sure that there are no Hadoop configuration files, hadoop-env.sh and hadoop-site.xml, present in the conf directory of the tarred distribution. The presence of these files with incorrect values could make the cluster allocation to fail.

3.3. Using an external HDFS

In typical Hadoop clusters provisioned by HOD, HDFS is already set up statically (without using HOD). This allows data to persist in HDFS after the HOD provisioned clusters is deallocated. To use a statically configured HDFS, your hodrc must point to an external HDFS. Specifically, set the following options to the correct values in the section

Copyright © 2007 The Apache Software Foundation. All rights reserved.

`gridservice-hdfs` of the hodrc:

external = true
host = Hostname of the HDFS NameNode
fs_port = Port number of the HDFS NameNode
info_port = Port number of the HDFS NameNode web UI

Note: You can also enable this option from command line. That is, to use a static HDFS, you will need to say:

```
$ hod allocate -d cluster_dir -n number_of_nodes
--gridservice-hdfs.external
```

HOD can be used to provision an HDFS cluster as well as a Map/Reduce cluster, if required. To do so, set the following option in the section `gridservice-hdfs` of the hodrc:

external = false

3.4. Options for Configuring Hadoop

HOD provides a very convenient mechanism to configure both the Hadoop daemons that it provisions and also the hadoop-site.xml that it generates on the client side. This is done by specifying Hadoop configuration parameters in either the HOD configuration file, or from the command line when allocating clusters.

Configuring Hadoop Daemons

For configuring the Hadoop daemons, you can do the following:

For Map/Reduce, specify the options as a comma separated list of key-value pairs to the `server-params` option in the `gridservice-mapred` section. Likewise for a dynamically provisioned HDFS cluster, specify the options in the `server-params` option in the `gridservice-hdfs` section. If these parameters should be marked as *final*, then include these in the `final-server-params` option of the appropriate section.

For example:

```
server-params =
mapred.reduce.parallel.copies=20,io.sort.factor=100,io.sort.mb=128,io.file.buffer.size=[illegible]
```

```
final-server-params =
mapred.child.java.opts=-Xmx512m,dfs.block.size=134217728,fs.inmemory.size.mb=128
```

In order to provide the options from command line, you can use the following syntax:

Copyright © 2007 The Apache Software Foundation. All rights reserved.

For configuring the Map/Reduce daemons use:

```
$ hod allocate -d cluster_dir -n number_of_nodes
-Mmapred.reduce.parallel.copies=20 -Mio.sort.factor=100
```

In the example above, the *mapred.reduce.parallel.copies* parameter and the *io.sort.factor* parameter will be appended to the other `server-params` or if they already exist in `server-params`, will override them. In order to specify these are *final* parameters, you can use:

```
$ hod allocate -d cluster_dir -n number_of_nodes
-Fmapred.reduce.parallel.copies=20 -Fio.sort.factor=100
```

However, note that final parameters cannot be overwritten from command line. They can only be appended if not already specified.

Similar options exist for configuring dynamically provisioned HDFS daemons. For doing so, replace -M with -H and -F with -S.

Configuring Hadoop Job Submission (Client) Programs

As mentioned above, if the allocation operation completes successfully then `cluster_dir/hadoop-site.xml` will be generated and will contain information about the allocated cluster's JobTracker and NameNode. This configuration is used when submitting jobs to the cluster. HOD provides an option to include additional Hadoop configuration parameters into this file. The syntax for doing so is as follows:

```
$ hod allocate -d cluster_dir -n number_of_nodes
-Cmapred.userlog.limit.kb=200 -Cmapred.child.java.opts=-Xmx512m
```

In this example, the *mapred.userlog.limit.kb* and *mapred.child.java.opts* options will be included into the hadoop-site.xml that is generated by HOD.

3.5. Viewing Hadoop Web-UIs

The HOD allocation operation prints the JobTracker and NameNode web UI URLs. For example:

```
$ hod allocate -d ~/hadoop-cluster -n 10 -c ~/hod-conf-dir/hodrc
INFO - HDFS UI on http://host242.foo.com:55391
INFO - Mapred UI on http://host521.foo.com:54874
```

The same information is also available via the *info* operation described above.

3.6. Collecting and Viewing Hadoop Logs

Copyright © 2007 The Apache Software Foundation. All rights reserved.

To get the Hadoop logs of the daemons running on one of the allocated nodes:

- Log into the node of interest. If you want to look at the logs of the JobTracker or NameNode, then you can find the node running these by using the *list* and *info* operations mentioned above.
- Get the process information of the daemon of interest (for example, `ps ux | grep TaskTracker`)
- In the process information, search for the value of the variable `-Dhadoop.log.dir`. Typically this will be a decendent directory of the `hodring.temp-dir` value from the hod configuration file.
- Change to the `hadoop.log.dir` directory to view daemon and user logs.

HOD also provides a mechanism to collect logs when a cluster is being deallocated and persist them into a file system, or an externally configured HDFS. By doing so, these logs can be viewed after the jobs are completed and the nodes are released. In order to do so, configure the log-destination-uri to a URI as follows:

`log-destination-uri = hdfs://host123:45678/user/hod/logs` or
`log-destination-uri = file://path/to/store/log/files`

Under the root directory specified above in the path, HOD will create a path user_name/torque_jobid and store gzipped log files for each node that was part of the job.

Note that to store the files to HDFS, you may need to configure the `hodring.pkgs` option with the Hadoop version that matches the HDFS mentioned. If not, HOD will try to use the Hadoop version that it is using to provision the Hadoop cluster itself.

3.7. Auto-deallocation of Idle Clusters

HOD automatically deallocates clusters that are not running Hadoop jobs for a given period of time. Each HOD allocation includes a monitoring facility that constantly checks for running Hadoop jobs. If it detects no running Hadoop jobs for a given period, it will automatically deallocate its own cluster and thus free up nodes which are not being used effectively.

Note: While the cluster is deallocated, the *cluster directory* is not cleaned up automatically. The user must deallocate this cluster through the regular *deallocate* operation to clean this up.

3.8. Specifying Additional Job Attributes

HOD allows the user to specify a wallclock time and a name (or title) for a Torque job.

Copyright © 2007 The Apache Software Foundation. All rights reserved.

The wallclock time is the estimated amount of time for which the Torque job will be valid. After this time has expired, Torque will automatically delete the job and free up the nodes. Specifying the wallclock time can also help the job scheduler to better schedule jobs, and help improve utilization of cluster resources.

To specify the wallclock time, use the following syntax:

```
$ hod allocate -d cluster_dir -n number_of_nodes -l time_in_seconds
```

The name or title of a Torque job helps in user friendly identification of the job. The string specified here will show up in all information where Torque job attributes are displayed, including the `qstat` command.

To specify the name or title, use the following syntax:

```
$ hod allocate -d cluster_dir -n number_of_nodes -N name_of_job
```

Note: Due to restriction in the underlying Torque resource manager, names which do not start with an alphabet character or contain a 'space' will cause the job to fail. The failure message points to the problem being in the specified job name.

3.9. Capturing HOD exit codes in Torque

HOD exit codes are captured in the Torque exit_status field. This will help users and system administrators to distinguish successful runs from unsuccessful runs of HOD. The exit codes are 0 if allocation succeeded and all hadoop jobs ran on the allocated cluster correctly. They are non-zero if allocation failed or some of the hadoop jobs failed on the allocated cluster. The exit codes that are possible are mentioned in the table below. *Note: Hadoop job status is captured only if the version of Hadoop used is 16 or above.*

Exit Code	Meaning
6	Ringmaster failure
7	DFS failure
8	Job tracker failure
10	Cluster dead
12	Cluster already allocated
13	HDFS dead
14	Mapred dead
16	All Map/Reduce jobs that ran on the cluster

Copyright © 2007 The Apache Software Foundation. All rights reserved.

	failed. Refer to hadoop logs for more details.
17	Some of the Map/Reduce jobs that ran on the cluster failed. Refer to hadoop logs for more details.

3.10. Command Line

HOD command line has the following general syntax:
hod <operation> [ARGS] [OPTIONS]
Allowed operations are 'allocate', 'deallocate', 'info', 'list', 'script' and 'help'. For help on a particular operation one can do : `hod help <operation>`. To have a look at possible options one can do a `hod help options.`

allocate
Usage : hod allocate -d cluster_dir -n number_of_nodes [OPTIONS]
Allocates a cluster on the given number of cluster nodes, and store the allocation information in cluster_dir for use with subsequent `hadoop` commands. Note that the `cluster_dir` must exist before running the command.

list
Usage : hod list [OPTIONS]
Lists the clusters allocated by this user. Information provided includes the Torque job id corresponding to the cluster, the cluster directory where the allocation information is stored, and whether the Map/Reduce daemon is still active or not.

info
Usage : hod info -d cluster_dir [OPTIONS]
Lists information about the cluster whose allocation information is stored in the specified cluster directory.

deallocate
Usage : hod deallocate -d cluster_dir [OPTIONS]
Deallocates the cluster whose allocation information is stored in the specified cluster directory.

script
Usage : hod script -s script_file -d cluster_directory -n number_of_nodes [OPTIONS]
Runs a hadoop script using HOD*script* operation. Provisions Hadoop on a given number of nodes, executes the given script from the submitting node, and deallocates the cluster when the script completes.

help

Copyright © 2007 The Apache Software Foundation. All rights reserved.

Usage : hod help [operation | 'options']
When no argument is specified, `hod help` gives the usage and basic options, and is equivalent to `hod --help` (See below). When 'options' is given as argument, hod displays only the basic options that hod takes. When an operation is specified, it displays the usage and description corresponding to that particular operation. For e.g, to know about allocate operation, one can do a `hod help allocate`

Besides the operations, HOD can take the following command line options.

--help
Prints out the help message to see the usage and basic options.

--verbose-help
All configuration options provided in the hodrc file can be passed on the command line, using the syntax `--section_name.option_name[=value]`. When provided this way, the value provided on command line overrides the option provided in hodrc. The verbose-help command lists all the available options in the hodrc file. This is also a nice way to see the meaning of the configuration options.

See the next section for a description of most important hod configuration options. For basic options, one can do a `hod help options` and for all options possible in hod configuration, one can see `hod --verbose-help`. See config guide for a description of all options.

3.11. Options Configuring HOD

As described above, HOD is configured using a configuration file that is usually set up by system administrators. This is a INI style configuration file that is divided into sections, and options inside each section. Each section relates to one of the HOD processes: client, ringmaster, hodring, mapreduce or hdfs. The options inside a section comprise of an option name and value.

Users can override the configuration defined in the default configuration in two ways:

- Users can supply their own configuration file to HOD in each of the commands, using the `-c` option
- Users can supply specific configuration options to HOD/ Options provided on command line *override* the values provided in the configuration file being used.

This section describes some of the most commonly used configuration options. These commonly used options are provided with a *short* option for convenience of specification. All other options can be specified using a *long* option that is also described below.

-c config_file

Copyright © 2007 The Apache Software Foundation. All rights reserved.

Provides the configuration file to use. Can be used with all other options of HOD. Alternatively, the `HOD_CONF_DIR` environment variable can be defined to specify a directory that contains a file named `hodrc`, alleviating the need to specify the configuration file in each HOD command.

-d cluster_dir
This is required for most of the hod operations. As described here, the *cluster directory* is a directory on the local file system where `hod` will generate the Hadoop configuration, *hadoop-site.xml*, corresponding to the cluster it allocates. Pass it to the `hod` operations as an argument to -d or --hod.clusterdir. If it doesn't already exist, HOD will automatically try to create it and use it. Once a cluster is allocated, a user can utilize it to run Hadoop jobs by specifying the clusterdirectory as the Hadoop --config option.

-n number_of_nodes
This is required for the hod 'allocation' operation and for script operation. This denotes the number of nodes to be allocated.

-s script-file
Required when using script operation, specifies the script file to execute.

-b 1|2|3|4
Enables the given debug level. Can be used with all other options of HOD. 4 is most verbose.

-t hadoop_tarball
Provisions Hadoop from the given tar.gz file. This option is only applicable to the *allocate* operation. For better distribution performance it is strongly recommended that the Hadoop tarball is created *after* removing the source or documentation.

-N job-name
The Name to give to the resource manager job that HOD uses underneath. For e.g. in the case of Torque, this translates to the `qsub -N` option, and can be seen as the job name using the `qstat` command.

-l wall-clock-time
The amount of time for which the user expects to have work on the allocated cluster. This is passed to the resource manager underneath HOD, and can be used in more efficient scheduling and utilization of the cluster. Note that in the case of Torque, the cluster is automatically deallocated after this time expires.

-j java-home
Path to be set to the JAVA_HOME environment variable. This is used in the *script* operation. HOD sets the JAVA_HOME environment variable tot his value and launches the user script in that.

Copyright © 2007 The Apache Software Foundation. All rights reserved.

-A account-string
Accounting information to pass to underlying resource manager.

-Q queue-name
Name of the queue in the underlying resource manager to which the job must be submitted.

-Mkey1=value1 -Mkey2=value2
Provides configuration parameters for the provisioned Map/Reduce daemons (JobTracker and TaskTrackers). A hadoop-site.xml is generated with these values on the cluster nodes. *Note:* Values which have the following characters: space, comma, equal-to, semi-colon need to be escaped with a '\' character, and need to be enclosed within quotes. You can escape a '\' with a '\' too.

-Hkey1=value1 -Hkey2=value2
Provides configuration parameters for the provisioned HDFS daemons (NameNode and DataNodes). A hadoop-site.xml is generated with these values on the cluster nodes
Note: Values which have the following characters: space, comma, equal-to, semi-colon need to be escaped with a '\' character, and need to be enclosed within quotes. You can escape a '\' with a '\' too.

-Ckey1=value1 -Ckey2=value2
Provides configuration parameters for the client from where jobs can be submitted. A hadoop-site.xml is generated with these values on the submit node.
Note: Values which have the following characters: space, comma, equal-to, semi-colon need to be escaped with a '\' character, and need to be enclosed within quotes. You can escape a '\' with a '\' too.

--section-name.option-name=value
This is the method to provide options using the *long* format. For e.g. you could say *--hod.script-wait-time=20*

4. Troubleshooting

The following section identifies some of the most likely error conditions users can run into when using HOD and ways to trouble-shoot them

4.1. hod Hangs During Allocation

Possible Cause: One of the HOD or Hadoop components have failed to come up. In such a case, the `hod` command will return after a few minutes (typically 2-3 minutes) with an error code of either 7 or 8 as defined in the Error Codes section. Refer to that section for further details.

Copyright © 2007 The Apache Software Foundation. All rights reserved.

Possible Cause: A large allocation is fired with a tarball. Sometimes due to load in the network, or on the allocated nodes, the tarball distribution might be significantly slow and take a couple of minutes to come back. Wait for completion. Also check that the tarball does not have the Hadoop sources or documentation.

Possible Cause: A Torque related problem. If the cause is Torque related, the `hod` command will not return for more than 5 minutes. Running `hod` in debug mode may show the `qstat` command being executed repeatedly. Executing the `qstat` command from a separate shell may show that the job is in the `Q` (Queued) state. This usually indicates a problem with Torque. Possible causes could include some nodes being down, or new nodes added that Torque is not aware of. Generally, system administator help is needed to resolve this problem.

4.2. hod Hangs During Deallocation

Possible Cause: A Torque related problem, usually load on the Torque server, or the allocation is very large. Generally, waiting for the command to complete is the only option.

4.3. hod Fails With an error code and error message

If the exit code of the `hod` command is not `0`, then refer to the following table of error exit codes to determine why the code may have occurred and how to debug the situation.

Error Codes

Error Code	Meaning	Possible Causes and Remedial Actions
1	Configuration error	Incorrect configuration values specified in hodrc, or other errors related to HOD configuration. The error messages in this case must be sufficient to debug and fix the problem.
2	Invalid operation	Do `hod help` for the list of valid operations.
3	Invalid operation arguments	Do `hod help operation` for listing the usage of a particular operation.
4	Scheduler failure	1. Requested more resources than available. Run `checknodes cluster_name`

Copyright © 2007 The Apache Software Foundation. All rights reserved.

		to see if enough nodes are available.<br>2. Requested resources exceed resource manager limits.<br>3. Torque is misconfigured, the path to Torque binaries is misconfigured, or other Torque problems. Contact system administrator.
5	Job execution failure	1. Torque Job was deleted from outside. Execute the Torque `qstat` command to see if you have any jobs in the `R` (Running) state. If none exist, try re-executing HOD.<br>2. Torque problems such as the server momentarily going down, or becoming unresponsive. Contact system administrator.
6	Ringmaster failure	HOD prints the message "Cluster could not be allocated because of the following errors on the ringmaster host <hostname>". The actual error message may indicate one of the following:<br>1. Invalid configuration on the node running the ringmaster, specified by the hostname in the error message.<br>2. Invalid configuration in the `ringmaster` section,<br>3. Invalid `pkgs` option in `gridservice-mapred or gridservice-hdfs` section,<br>4. An invalid hadoop tarball, or a tarball which has bundled an invalid configuration file in the conf directory,<br>5. Mismatched version in Hadoop between the MapReduce and an external HDFS.<br>The Torque `qstat` command

Copyright © 2007 The Apache Software Foundation. All rights reserved.

		will most likely show a job in the `C` (Completed) state.<br>One can login to the ringmaster host as given by HOD failure message and debug the problem with the help of the error message. If the error message doesn't give complete information, ringmaster logs should help finding out the root cause of the problem. Refer to the section *Locating Ringmaster Logs* below for more information.
7	DFS failure	When HOD fails to allocate due to DFS failures (or Job tracker failures, error code 8, see below), it prints a failure message "Hodring at <hostname> failed with following errors:" and then gives the actual error message, which may indicate one of the following:<br>1. Problem in starting Hadoop clusters. Usually the actual cause in the error message will indicate the problem on the hostname mentioned. Also, review the Hadoop related configuration in the HOD configuration files. Look at the Hadoop logs using information specified in *Collecting and Viewing Hadoop Logs* section above.<br>2. Invalid configuration on the node running the hodring, specified by the hostname in the error message<br>3. Invalid configuration in the `hodring` section of hodrc. `ssh` to the hostname specified in the error message and grep for `ERROR` or `CRITICAL` in hodring logs. Refer to the section *Locating Hodring Logs* below

Copyright © 2007 The Apache Software Foundation. All rights reserved.

		for more information.<br>4. Invalid tarball specified which is not packaged correctly.<br>5. Cannot communicate with an externally configured HDFS.<br>When such DFS or Job tracker failure occurs, one can login into the host with hostname mentioned in HOD failure message and debug the problem. While fixing the problem, one should also review other log messages in the ringmaster log to see which other machines also might have had problems bringing up the jobtracker/namenode, apart from the hostname that is reported in the failure message. This possibility of other machines also having problems occurs because HOD continues to try and launch hadoop daemons on multiple machines one after another depending upon the value of the configuration variable ringmaster.max-master-failures. Refer to the section *Locating Ringmaster Logs* below to find more about ringmaster logs.
8	Job tracker failure	Similar to the causes in *DFS failure* case.
10	Cluster dead	1. Cluster was auto-deallocated because it was idle for a long time.<br>2. Cluster was auto-deallocated because the wallclock time specified by the system administrator or user was exceeded.<br>3. Cannot communicate with the JobTracker and HDFS NameNode which were successfully allocated. Deallocate the cluster, and

Copyright © 2007 The Apache Software Foundation. All rights reserved.

		allocate again.
12	Cluster already allocated	The cluster directory specified has been used in a previous allocate operation and is not yet deallocated. Specify a different directory, or deallocate the previous allocation first.
13	HDFS dead	Cannot communicate with the HDFS NameNode. HDFS NameNode went down.
14	Mapred dead	1. Cluster was auto-deallocated because it was idle for a long time.<br>2. Cluster was auto-deallocated because the wallclock time specified by the system administrator or user was exceeded.<br>3. Cannot communicate with the Map/Reduce JobTracker. JobTracker node went down.
15	Cluster not allocated	An operation which requires an allocated cluster is given a cluster directory with no state information.
Any non-zero exit code	HOD script error	If the hod script option was used, it is likely that the exit code is from the script. Unfortunately, this could clash with the exit codes of the hod command itself. In order to help users differentiate these two, hod writes the script's exit code to a file called script.exitcode in the cluster directory, if the script returned an exit code. You can cat this file to determine the script's exit code. If it does not exist, then it is a hod command exit code.

4.4. Hadoop Jobs Not Running on a Successfully Allocated Cluster

Copyright © 2007 The Apache Software Foundation. All rights reserved.

This scenario generally occurs when a cluster is allocated, and is left inactive for sometime, and then hadoop jobs are attempted to be run on them. Then Hadoop jobs fail with the following exception:

```
08/01/25 16:31:40 INFO ipc.Client: Retrying connect to server:
foo.bar.com/1.1.1.1:53567. Already tried 1 time(s).
```

Possible Cause: No Hadoop jobs were run for a significant portion of time. Thus the cluster would have got deallocated as described in the section *Auto-deallocation of Idle Clusters*. Deallocate the cluster and allocate it again.

Possible Cause: The wallclock limit specified by the Torque administrator or the `-l` option defined in the section *Specifying Additional Job Attributes* was exceeded since allocation time. Thus the cluster would have got released. Deallocate the cluster and allocate it again.

Possible Cause: There is a version mismatch between the version of the hadoop being used in provisioning (typically via the tarball option) and the external HDFS. Ensure compatible versions are being used.

Possible Cause: There is a version mismatch between the version of the hadoop client being used to submit jobs and the hadoop used in provisioning (typically via the tarball option). Ensure compatible versions are being used.

Possible Cause: You used one of the options for specifying Hadoop configuration `-M or -H`, which had special characters like space or comma that were not escaped correctly. Refer to the section *Options Configuring HOD* for checking how to specify such options correctly.

4.5. My Hadoop Job Got Killed

Possible Cause: The wallclock limit specified by the Torque administrator or the `-l` option defined in the section *Specifying Additional Job Attributes* was exceeded since allocation time. Thus the cluster would have got released. Deallocate the cluster and allocate it again, this time with a larger wallclock time.

Possible Cause: Problems with the JobTracker node. Refer to the section in *Collecting and Viewing Hadoop Logs* to get more information.

4.6. Hadoop Job Fails with Message: 'Job tracker still initializing'

Possible Cause: The hadoop job was being run as part of the HOD script command, and it started before the JobTracker could come up fully. Allocate the cluster using a large value for the configuration option `--hod.script-wait-time`. Typically a value of 120 should work, though it is typically unnecessary to be that large.

Copyright © 2007 The Apache Software Foundation. All rights reserved.

4.7. The Exit Codes For HOD Are Not Getting Into Torque

Possible Cause: Version 0.16 of hadoop is required for this functionality to work. The version of Hadoop used does not match. Use the required version of Hadoop.

Possible Cause: The deallocation was done without using the `hod` command; for e.g. directly using `qdel`. When the cluster is deallocated in this manner, the HOD processes are terminated using signals. This results in the exit code to be based on the signal number, rather than the exit code of the program.

4.8. The Hadoop Logs are Not Uploaded to DFS

Possible Cause: There is a version mismatch between the version of the hadoop being used for uploading the logs and the external HDFS. Ensure that the correct version is specified in the `hodring.pkgs` option.

4.9. Locating Ringmaster Logs

To locate the ringmaster logs, follow these steps:

- Execute hod in the debug mode using the -b option. This will print the Torque job id for the current run.
- Execute `qstat -f torque_job_id` and look up the value of the `exec_host` parameter in the output. The first host in this list is the ringmaster node.
- Login to this node.
- The ringmaster log location is specified by the `ringmaster.log-dir` option in the hodrc. The name of the log file will be `username.torque_job_id/ringmaster-main.log`.
- If you don't get enough information, you may want to set the ringmaster debug level to 4. This can be done by passing `--ringmaster.debug 4` to the hod command line.

4.10. Locating Hodring Logs

To locate hodring logs, follow the steps below:

- Execute hod in the debug mode using the -b option. This will print the Torque job id for the current run.
- Execute `qstat -f torque_job_id` and look up the value of the `exec_host` parameter in the output. All nodes in this list should have a hodring on them.
- Login to any of these nodes.
- The hodring log location is specified by the `hodring.log-dir` option in the hodrc. The name of the log file will be

Copyright © 2007 The Apache Software Foundation. All rights reserved.

`username.torque_job_id/hodring-main.log`.

- If you don't get enough information, you may want to set the hodring debug level to 4. This can be done by passing `--hodring.debug 4` to the hod command line.

Copyright © 2007 The Apache Software Foundation. All rights reserved.
