# Supplementary material for: Provenance based data integrity checking and verification in cloud environments
Source: PLoS One. 2017 May 17;12(5):e0177576. doi: 10.1371/journal.pone.0177576 (PMC5435237; doi:10.1371/journal.pone.0177576)
Supplement: S1 Dataset — Files in the Dataset are utilized for performance measurement. (ZIP) [file pone.0177576.s001.zip › dataset/pdf/index - Copy (2) - Copy.pdf]

Hadoop Documentation

The following documents provide concepts and procedures that will help you get started using Hadoop. If you have more questions, you can ask the mailing list or browse the archives.

- Hadoop Quickstart
- Hadoop Cluster Setup
- Hadoop Distributed File System
- Hadoop Map-Reduce Tutorial
- Native Hadoop Libraries
- API Docs
- Wiki
- FAQ

Copyright © 2007 The Apache Software Foundation. All rights reserved.
