# Supplementary material for: Provenance based data integrity checking and verification in cloud environments
Source: PLoS One. 2017 May 17;12(5):e0177576. doi: 10.1371/journal.pone.0177576 (PMC5435237; doi:10.1371/journal.pone.0177576)
Supplement: S1 Dataset — Files in the Dataset are utilized for performance measurement. (ZIP) [file pone.0177576.s001.zip › dataset/pdf/linkmap - Copy (2) - Copy.pdf]

Site Linkmap Table of Contents

This is a map of the complete site and its structure.

- Hadoop ___________________ *site*
    - Documentation ___________________ *docs*
        - Overview ___________________ *overview*
        - Quickstart ___________________ *quickstart*
        - Cluster Setup ___________________ *setup*
        - HDFS Architecture ___________________ *hdfs*
        - HDFS User Guide ___________________ *hdfs*
        - HDFS Permissions Guide ___________________ *hdfs*
        - HDFS Quotas Administrator Guide ___________________ *hdfs*
        - Commands Manual ___________________ *commands*
        - FS Shell Guide ___________________ *fs*
        - DistCp Guide ___________________ *distcp*
        - Map-Reduce Tutorial ___________________ *mapred*
        - Native Hadoop Libraries ___________________ *mapred*
        - Streaming ___________________ *streaming*
        - Hadoop Archives ___________________ *archives*
        - Hadoop On Demand ___________________ *hod*
        - API Docs ___________________ *api*
        - Wiki ___________________ *wiki*
        - FAQ ___________________ *faq*
        - Mailing Lists ___________________ *lists*
        - Release Notes ___________________ *relnotes*
        - All Changes ___________________ *changes*

Copyright © 2007 The Apache Software Foundation. All rights reserved.

Copyright © 2007 The Apache Software Foundation. All rights reserved.
