# Supplementary material for: Provenance based data integrity checking and verification in cloud environments
Source: PLoS One. 2017 May 17;12(5):e0177576. doi: 10.1371/journal.pone.0177576 (PMC5435237; doi:10.1371/journal.pone.0177576)
Supplement: S1 Dataset — Files in the Dataset are utilized for performance measurement. (ZIP) [file pone.0177576.s001.zip › dataset/pdf/mapred_tutorial - Copy (2) - Copy.pdf]

Hadoop Map/Reduce Tutorial

Table of contents

1 Purpose...2
2 Pre-requisites...2
3 Overview...2
4 Inputs and Outputs...3
5 Example: WordCount v1.0...3
5.1 Source Code...3
5.2 Usage...6
5.3 Walk-through...7
6 Map/Reduce - User Interfaces...9
6.1 Payload...9
6.2 Job Configuration...13
6.3 Task Execution & Environment...14
6.4 Job Submission and Monitoring...16
6.5 Job Input...18
6.6 Job Output...19
6.7 Other Useful Features...20
7 Example: WordCount v2.0...24
7.1 Source Code...24
7.2 Sample Runs...31
7.3 Highlights...32

Copyright © 2007 The Apache Software Foundation. All rights reserved.

1. Purpose

This document comprehensively describes all user-facing facets of the Hadoop Map/Reduce framework and serves as a tutorial.

2. Pre-requisites

Ensure that Hadoop is installed, configured and is running. More details:

- Hadoop Quickstart for first-time users.
- Hadoop Cluster Setup for large, distributed clusters.

3. Overview

Hadoop Map/Reduce is a software framework for easily writing applications which process vast amounts of data (multi-terabyte data-sets) in-parallel on large clusters (thousands of nodes) of commodity hardware in a reliable, fault-tolerant manner.

A Map/Reduce *job* usually splits the input data-set into independent chunks which are processed by the *map tasks* in a completely parallel manner. The framework sorts the outputs of the maps, which are then input to the *reduce tasks*. Typically both the input and the output of the job are stored in a file-system. The framework takes care of scheduling tasks, monitoring them and re-executes the failed tasks.

Typically the compute nodes and the storage nodes are the same, that is, the Map/Reduce framework and the Distributed FileSystem are running on the same set of nodes. This configuration allows the framework to effectively schedule tasks on the nodes where data is already present, resulting in very high aggregate bandwidth across the cluster.

The Map/Reduce framework consists of a single master `JobTracker` and one slave `TaskTracker` per cluster-node. The master is responsible for scheduling the jobs' component tasks on the slaves, monitoring them and re-executing the failed tasks. The slaves execute the tasks as directed by the master.

Minimally, applications specify the input/output locations and supply *map* and *reduce* functions via implementations of appropriate interfaces and/or abstract-classes. These, and other job parameters, comprise the *job configuration*. The Hadoop *job client* then submits the job (jar/executable etc.) and configuration to the `JobTracker` which then assumes the responsibility of distributing the software/configuration to the slaves, scheduling tasks and monitoring them, providing status and diagnostic information to the job-client.

Although the Hadoop framework is implemented in JavaTM, Map/Reduce applications need

Copyright © 2007 The Apache Software Foundation. All rights reserved.

not be written in Java.

- Hadoop Streaming is a utility which allows users to create and run jobs with any executables (e.g. shell utilities) as the mapper and/or the reducer.
- Hadoop Pipes is a SWIG- compatible *C++ API* to implement Map/Reduce applications (non JNITM based).

4. Inputs and Outputs

The Map/Reduce framework operates exclusively on `<key, value>` pairs, that is, the framework views the input to the job as a set of `<key, value>` pairs and produces a set of `<key, value>` pairs as the output of the job, conceivably of different types.

The `key` and `value` classes have to be serializable by the framework and hence need to implement the Writable interface. Additionally, the `key` classes have to implement the WritableComparable interface to facilitate sorting by the framework.

Input and Output types of a Map/Reduce job:

(input) `<k1, v1>` -> **map** -> `<k2, v2>` -> **combine** -> `<k2, v2>` -> **reduce** -> `<k3, v3>` (output)

5. Example: WordCount v1.0

Before we jump into the details, lets walk through an example Map/Reduce application to get a flavour for how they work.

`WordCount` is a simple application that counts the number of occurences of each word in a given input set.

This works with a local-standalone, pseudo-distributed or fully-distributed Hadoop installation.

5.1. Source Code

WordCount.java

```
package org.myorg;

import java.io.IOException;
import java.util.*;

```


Copyright © 2007 The Apache Software Foundation. All rights reserved.

```
import org.apache.hadoop.fs.Path;
import org.apache.hadoop.conf.*;
import org.apache.hadoop.io.*;
import org.apache.hadoop.mapred.*;
import org.apache.hadoop.util.*;

public class WordCount {

  public static class Map extends MapReduceBase implements Mapper<LongWritable, Text, Text, IntWritable> {
    private final static IntWritable one = new IntWritable(1);
    private Text word = new Text();

    public void map(LongWritable key, Text value, OutputCollector<Text, IntWritable> output, Reporter reporter) throws IOException {
      String line = value.toString();
      StringTokenizer tokenizer = new StringTokenizer(line);
      while (tokenizer.hasMoreTokens()) {
        word.set(tokenizer.nextToken());
        output.collect(word, one);
      }
    }
  }
```


Copyright © 2007 The Apache Software Foundation. All rights reserved.

```
 public static class Reduce extends MapReduceBase implements Reducer<Text, IntWritable, Text, IntWritable> {
  public void reduce(Text key, Iterator<IntWritable> values, OutputCollector<Text, IntWritable> output, Reporter reporter) throws IOException {
   int sum = 0;
   while (values.hasNext()) {
    sum += values.next().get();
   }
   output.collect(key, new IntWritable(sum));
  }
 }

 public static void main(String[] args) throws Exception {
  JobConf conf = new JobConf(WordCount.class);
  conf.setJobName("wordcount");

  conf.setOutputKeyClass(Text.class);
  conf.setOutputValueClass(IntWritable.class);

  conf.setMapperClass(Map.class);

```


Copyright © 2007 The Apache Software Foundation. All rights reserved.

```
    conf.setCombinerClass(Reduce.class);
    conf.setReducerClass(Reduce.class);

    conf.setInputFormat(TextInputFormat.class);
    conf.setOutputFormat(TextOutputFormat.class);

    FileInputFormat.setInputPaths(conf, new Path(args[0]));
    FileOutputFormat.setOutputPath(conf, new Path(args[1]));

    JobClient.runJob(conf);
  }
}

```

5.2. Usage

Assuming `HADOOP_HOME` is the root of the installation and `HADOOP_VERSION` is the Hadoop version installed, compile `WordCount.java` and create a jar:

```
$ mkdir wordcount_classes
$ javac -classpath
${HADOOP_HOME}/hadoop-${HADOOP_VERSION}-core.jar -d
wordcount_classes WordCount.java
$ jar -cvf /usr/joe/wordcount.jar -C wordcount_classes/ .
```

Assuming that:

- `/usr/joe/wordcount/input` - input directory in HDFS
- `/usr/joe/wordcount/output` - output directory in HDFS

Copyright © 2007 The Apache Software Foundation. All rights reserved.

Sample text-files as input:

```
$ bin/hadoop dfs -ls /usr/joe/wordcount/input/
/usr/joe/wordcount/input/file01
/usr/joe/wordcount/input/file02
$ bin/hadoop dfs -cat /usr/joe/wordcount/input/file01
Hello World Bye World
$ bin/hadoop dfs -cat /usr/joe/wordcount/input/file02
Hello Hadoop Goodbye Hadoop
```

Run the application:

```
$ bin/hadoop jar /usr/joe/wordcount.jar org.myorg.WordCount
/usr/joe/wordcount/input /usr/joe/wordcount/output
```

Output:

```
$ bin/hadoop dfs -cat /usr/joe/wordcount/output/part-00000
Bye 1
Goodbye 1
Hadoop 2
Hello 2
World 2
```

Applications can specify a comma separated list of paths which would be present in the current working directory of the task using the option `-files`. The `-libjars` option allows applications to add jars to the classpaths of the maps and reduces. The `-archives` allows them to pass archives as arguments that are unzipped/unjarred and a link with name of the jar/zip are created in the current working directory of tasks. More details about the command line options are available at Commands manual

Running `wordcount` example with `-libjars` and `-files`:

```
hadoop jar hadoop-examples.jar wordcount -files cachefile.txt
-libjars mylib.jar input output
```

5.3. Walk-through

The `WordCount` application is quite straight-forward.

The `Mapper` implementation (lines 14-26), via the `map` method (lines 18-25), processes one line at a time, as provided by the specified `TextInputFormat` (line 49). It then splits the line into tokens separated by whitespaces, via the `StringTokenizer`, and emits a key-value pair of `< <word>, 1>`.

Copyright © 2007 The Apache Software Foundation. All rights reserved.

For the given sample input the first map emits:
```
< Hello, 1>
< World, 1>
< Bye, 1>
< World, 1>
```

The second map emits:
```
< Hello, 1>
< Hadoop, 1>
< Goodbye, 1>
< Hadoop, 1>
```

We'll learn more about the number of maps spawned for a given job, and how to control them in a fine-grained manner, a bit later in the tutorial.

`WordCount` also specifies a `combiner` (line 46). Hence, the output of each map is passed through the local combiner (which is same as the `Reducer` as per the job configuration) for local aggregation, after being sorted on the *key*s.

The output of the first map:
```
< Bye, 1>
< Hello, 1>
< World, 2>
```

The output of the second map:
```
< Goodbye, 1>
< Hadoop, 2>
< Hello, 1>
```

The `Reducer` implementation (lines 28-36), via the `reduce` method (lines 29-35) just sums up the values, which are the occurence counts for each key (i.e. words in this example).

Thus the output of the job is:
```
< Bye, 1>
< Goodbye, 1>
< Hadoop, 2>
< Hello, 2>
< World, 2>
```

The `run` method specifies various facets of the job, such as the input/output paths (passed via the command line), key/value types, input/output formats etc., in the `JobConf`. It then calls the `JobClient.runJob` (line 55) to submit the and monitor its progress.

We'll learn more about `JobConf`, `JobClient`, `Tool` and other interfaces and classes a bit

Copyright © 2007 The Apache Software Foundation. All rights reserved.

later in the tutorial.

6. Map/Reduce - User Interfaces

This section provides a reasonable amount of detail on every user-facing aspect of the Map/Reduce framwork. This should help users implement, configure and tune their jobs in a fine-grained manner. However, please note that the javadoc for each class/interface remains the most comprehensive documentation available; this is only meant to be a tutorial.

Let us first take the `Mapper` and `Reducer` interfaces. Applications typically implement them to provide the `map` and `reduce` methods.

We will then discuss other core interfaces including `JobConf`, `JobClient`, `Partitioner`, `OutputCollector`, `Reporter`, `InputFormat`, `OutputFormat` and others.

Finally, we will wrap up by discussing some useful features of the framework such as the `DistributedCache`, `IsolationRunner` etc.

6.1. Payload

Applications typically implement the `Mapper` and `Reducer` interfaces to provide the `map` and `reduce` methods. These form the core of the job.

6.1.1. Mapper

Mapper maps input key/value pairs to a set of intermediate key/value pairs.

Maps are the individual tasks that transform input records into intermediate records. The transformed intermediate records do not need to be of the same type as the input records. A given input pair may map to zero or many output pairs.

The Hadoop Map/Reduce framework spawns one map task for each `InputSplit` generated by the `InputFormat` for the job.

Overall, `Mapper` implementations are passed the `JobConf` for the job via the JobConfigurable.configure(JobConf) method and override it to initialize themselves. The framework then calls map(WritableComparable, Writable, OutputCollector, Reporter) for each key/value pair in the `InputSplit` for that task. Applications can then override the Closeable.close() method to perform any required cleanup.

Output pairs do not need to be of the same types as input pairs. A given input pair may map to zero or many output pairs. Output pairs are collected with calls to

Copyright © 2007 The Apache Software Foundation. All rights reserved.

OutputCollector.collect(WritableComparable,Writable).

Applications can use the `Reporter` to report progress, set application-level status messages and update `Counters`, or just indicate that they are alive.

All intermediate values associated with a given output key are subsequently grouped by the framework, and passed to the `Reducer`(s) to determine the final output. Users can control the grouping by specifying a `Comparator` via JobConf.setOutputKeyComparatorClass(Class).

The `Mapper` outputs are sorted and then partitioned per `Reducer`. The total number of partitions is the same as the number of reduce tasks for the job. Users can control which keys (and hence records) go to which `Reducer` by implementing a custom `Partitioner`.

Users can optionally specify a `combiner`, via JobConf.setCombinerClass(Class), to perform local aggregation of the intermediate outputs, which helps to cut down the amount of data transferred from the `Mapper` to the `Reducer`.

The intermediate, sorted outputs are always stored in a simple (key-len, key, value-len, value) format. Applications can control if, and how, the intermediate outputs are to be compressed and the CompressionCodec to be used via the `JobConf`.

6.1.1.1. How Many Maps?

The number of maps is usually driven by the total size of the inputs, that is, the total number of blocks of the input files.

The right level of parallelism for maps seems to be around 10-100 maps per-node, although it has been set up to 300 maps for very cpu-light map tasks. Task setup takes awhile, so it is best if the maps take at least a minute to execute.

Thus, if you expect 10TB of input data and have a blocksize of `128MB`, you'll end up with 82,000 maps, unless setNumMapTasks(int) (which only provides a hint to the framework) is used to set it even higher.

6.1.2. Reducer

Reducer reduces a set of intermediate values which share a key to a smaller set of values.

The number of reduces for the job is set by the user via JobConf.setNumReduceTasks(int).

Overall, `Reducer` implementations are passed the `JobConf` for the job via the JobConfigurable.configure(JobConf) method and can override it to initialize themselves. The framework then calls reduce(WritableComparable, Iterator, OutputCollector, Reporter)

Copyright © 2007 The Apache Software Foundation. All rights reserved.

method for each `<key, (list of values)>` pair in the grouped inputs. Applications can then override the Closeable.close() method to perform any required cleanup.

`Reducer` has 3 primary phases: shuffle, sort and reduce.

6.1.2.1. Shuffle

Input to the `Reducer` is the sorted output of the mappers. In this phase the framework fetches the relevant partition of the output of all the mappers, via HTTP.

6.1.2.2. Sort

The framework groups `Reducer` inputs by keys (since different mappers may have output the same key) in this stage.

The shuffle and sort phases occur simultaneously; while map-outputs are being fetched they are merged.

Secondary Sort

If equivalence rules for grouping the intermediate keys are required to be different from those for grouping keys before reduction, then one may specify a `Comparator` via JobConf.setOutputValueGroupingComparator(Class). Since JobConf.setOutputKeyComparatorClass(Class) can be used to control how intermediate keys are grouped, these can be used in conjunction to simulate *secondary sort on values*.

6.1.2.3. Reduce

In this phase the reduce(WritableComparable, Iterator, OutputCollector, Reporter) method is called for each `<key, (list of values)>` pair in the grouped inputs.

The output of the reduce task is typically written to the FileSystem via OutputCollector.collect(WritableComparable, Writable).

Applications can use the `Reporter` to report progress, set application-level status messages and update `Counters`, or just indicate that they are alive.

The output of the `Reducer` is *not sorted*.

6.1.2.4. How Many Reduces?

The right number of reduces seems to be `0.95` or `1.75` multiplied by (*<no. of nodes>* * `mapred.tasktracker.reduce.tasks.maximum`).

Copyright © 2007 The Apache Software Foundation. All rights reserved.

With `0.95` all of the reduces can launch immediately and start transfering map outputs as the maps finish. With `1.75` the faster nodes will finish their first round of reduces and launch a second wave of reduces doing a much better job of load balancing.

Increasing the number of reduces increases the framework overhead, but increases load balancing and lowers the cost of failures.

The scaling factors above are slightly less than whole numbers to reserve a few reduce slots in the framework for speculative-tasks and failed tasks.

6.1.2.5. Reducer NONE

It is legal to set the number of reduce-tasks to *zero* if no reduction is desired.

In this case the outputs of the map-tasks go directly to the `FileSystem`, into the output path set by setOutputPath(Path). The framework does not sort the map-outputs before writing them out to the `FileSystem`.

6.1.3. Partitioner

Partitioner partitions the key space.

Partitioner controls the partitioning of the keys of the intermediate map-outputs. The key (or a subset of the key) is used to derive the partition, typically by a *hash function*. The total number of partitions is the same as the number of reduce tasks for the job. Hence this controls which of the `m` reduce tasks the intermediate key (and hence the record) is sent to for reduction.

HashPartitioner is the default `Partitioner`.

6.1.4. Reporter

Reporter is a facility for Map/Reduce applications to report progress, set application-level status messages and update `Counters`.

`Mapper` and `Reducer` implementations can use the `Reporter` to report progress or just indicate that they are alive. In scenarios where the application takes a significant amount of time to process individual key/value pairs, this is crucial since the framework might assume that the task has timed-out and kill that task. Another way to avoid this is to set the configuration parameter `mapred.task.timeout` to a high-enough value (or even set it to *zero* for no time-outs).

Applications can also update `Counters` using the `Reporter`.

Copyright © 2007 The Apache Software Foundation. All rights reserved.

6.1.5. OutputCollector

OutputCollector is a generalization of the facility provided by the Map/Reduce framework to collect data output by the `Mapper` or the `Reducer` (either the intermediate outputs or the output of the job).

Hadoop Map/Reduce comes bundled with a library of generally useful mappers, reducers, and partitioners.

6.2. Job Configuration

JobConf represents a Map/Reduce job configuration.

`JobConf` is the primary interface for a user to describe a Map/Reduce job to the Hadoop framework for execution. The framework tries to faithfully execute the job as described by `JobConf`, however:

- f Some configuration parameters may have been marked as final by administrators and hence cannot be altered.
- While some job parameters are straight-forward to set (e.g. setNumReduceTasks(int)), other parameters interact subtly with the rest of the framework and/or job configuration and are more complex to set (e.g. setNumMapTasks(int)).

`JobConf` is typically used to specify the `Mapper`, combiner (if any), `Partitioner`, `Reducer`, `InputFormat` and `OutputFormat` implementations. `JobConf` also indicates the set of input files (setInputPaths(JobConf, Path...) /addInputPath(JobConf, Path)) and (setInputPaths(JobConf, String) /addInputPaths(JobConf, String)) and where the output files should be written (setOutputPath(Path)).

Optionally, `JobConf` is used to specify other advanced facets of the job such as the `Comparator` to be used, files to be put in the `DistributedCache`, whether intermediate and/or job outputs are to be compressed (and how), debugging via user-provided scripts (setMapDebugScript(String)/setReduceDebugScript(String)) , whether job tasks can be executed in a *speculative* manner (setMapSpeculativeExecution(boolean))/(setReduceSpeculativeExecution(boolean)) , maximum number of attempts per task (setMaxMapAttempts(int)/setMaxReduceAttempts(int)) , percentage of tasks failure which can be tolerated by the job (setMaxMapTaskFailuresPercent(int)/setMaxReduceTaskFailuresPercent(int)) etc.

Of course, users can use set(String, String)/get(String, String) to set/get arbitrary parameters needed by applications. However, use the `DistributedCache` for large amounts of

Copyright © 2007 The Apache Software Foundation. All rights reserved.

(read-only) data.

6.3. Task Execution & Environment

The `TaskTracker` executes the `Mapper`/ `Reducer` *task* as a child process in a separate jvm.

The child-task inherits the environment of the parent `TaskTracker`. The user can specify additional options to the child-jvm via the `mapred.child.java.opts` configuration parameter in the `JobConf` such as non-standard paths for the run-time linker to search shared libraries via `-Djava.library.path=<>` etc. If the `mapred.child.java.opts` contains the symbol *@taskid@* it is interpolated with value of `taskid` of the map/reduce task.

Here is an example with multiple arguments and substitutions, showing jvm GC logging, and start of a passwordless JVM JMX agent so that it can connect with jconsole and the likes to watch child memory, threads and get thread dumps. It also sets the maximum heap-size of the child jvm to 512MB and adds an additional path to the `java.library.path` of the child-jvm.

```
<property>
 <name>mapred.child.java.opts</name>
 <value>
   -Xmx512M -Djava.library.path=/home/mycompany/lib
-verbose:gc -Xloggc:/tmp/@taskid@.gc
   -Dcom.sun.management.jmxremote.authenticate=false
-Dcom.sun.management.jmxremote.ssl=false
 </value>
</property>
```

Users/admins can also specify the maximum virtual memory of the launched child-task using `mapred.child.ulimit`. The value for `mapred.child.ulimit` should be specified in kilo bytes (KB). And also the value must be greater than or equal to the -Xmx passed to JavaVM, else the VM might not start.

Note: `mapred.child.java.opts` are used only for configuring the launched child tasks from task tracker. Configuring the memory options for daemons is documented in cluster_setup.html

The task tracker has local directory, `${mapred.local.dir}/taskTracker/` to create localized cache and localized job. It can define multiple local directories (spanning multiple disks) and then each filename is assigned to a semi-random local directory. When the job starts, task tracker creates a localized job directory relative to the local directory specified in

Copyright © 2007 The Apache Software Foundation. All rights reserved.

the configuration. Thus the task tracker directory structure looks the following:

- `${mapred.local.dir}/taskTracker/archive/` : The distributed cache. This directory holds the localized distributed cache. Thus localized distributed cache is shared among all the tasks and jobs
- `${mapred.local.dir}/taskTracker/jobcache/$jobid/` : The localized job directory
  - `${mapred.local.dir}/taskTracker/jobcache/$jobid/work/` : The job-specific shared directory. The tasks can use this space as scratch space and share files among them. This directory is exposed to the users through the configuration property `job.local.dir`. The directory can accessed through api [JobConf.getJobLocalDir()](). It is available as System property also. So, users (streaming etc.) can call `System.getProperty("job.local.dir")` to access the directory.
  - `${mapred.local.dir}/taskTracker/jobcache/$jobid/jars/` : The jars directory, which has the job jar file and expanded jar. The `job.jar` is the application's jar file that is automatically distributed to each machine. It is expanded in jars directory before the tasks for the job start. The job.jar location is accessible to the application through the api [JobConf.getJar()]() . To access the unjarred directory, JobConf.getJar().getParent() can be called.
  - `${mapred.local.dir}/taskTracker/jobcache/$jobid/job.xml` : The job.xml file, the generic job configuration, localized for the job.
  - `${mapred.local.dir}/taskTracker/jobcache/$jobid/$taskid` : The task direcrory for each task attempt. Each task directory again has the following structure :
    - `${mapred.local.dir}/taskTracker/jobcache/$jobid/$taskid/job.xml` : A job.xml file, task localized job configuration, Task localization means that properties have been set that are specific to this particular task within the job. The properties localized for each task are described below.
    - `${mapred.local.dir}/taskTracker/jobcache/$jobid/$taskid/output` : A directory for intermediate output files. This contains the temporary map reduce data generated by the framework such as map output files etc.
    - `${mapred.local.dir}/taskTracker/jobcache/$jobid/$taskid/work` : The curernt working directory of the task.
    - `${mapred.local.dir}/taskTracker/jobcache/$jobid/$taskid/work/tmp` : The temporary directory for the task. (User can specify the property `mapred.child.tmp` to set the value of temporary directory for map and reduce tasks. This defaults to `./tmp`. If the value is not an absolute path, it is prepended with task's working directory. Otherwise, it is directly assigned. The directory will be created if it doesn't exist. Then, the child java tasks are executed with option `-Djava.io.tmpdir='the absolute path of the tmp`

Copyright © 2007 The Apache Software Foundation. All rights reserved.

`dir'`. Anp pipes and streaming are set with environment variable, `TMPDIR='the absolute path of the tmp dir'`). This directory is created, if `mapred.child.tmp` has the value `./tmp`

The following properties are localized in the job configuration for each task's execution:

Name	Type	Description
mapred.job.id	String	The job id
mapred.jar	String	job.jar location in job directory
job.local.dir	String	The job specific shared scratch space
mapred.tip.id	String	The task id
mapred.task.id	String	The task attempt id
mapred.task.is.map	boolean	Is this a map task
mapred.task.partition	int	The id of the task within the job
map.input.file	String	The filename that the map is reading from
map.input.start	long	The offset of the start of the map input split
map.input.length	long	The number of bytes in the map input split
mapred.work.output.dir	String	The task's temporary output directory

The standard output (stdout) and error (stderr) streams of the task are read by the TaskTracker and logged to `${HADOOP_LOG_DIR}/userlogs`

The DistributedCache can also be used to distribute both jars and native libraries for use in the map and/or reduce tasks. The child-jvm always has its *current working directory* added to the `java.library.path` and `LD_LIBRARY_PATH`. And hence the cached libraries can be loaded via System.loadLibrary or System.load. More details on how to load shared libraries through distributed cache are documented at native_libraries.html

6.4. Job Submission and Monitoring

JobClient is the primary interface by which user-job interacts with the `JobTracker`.

Copyright © 2007 The Apache Software Foundation. All rights reserved.

`JobClient` provides facilities to submit jobs, track their progress, access component-tasks' reports and logs, get the Map/Reduce cluster's status information and so on.

The job submission process involves:

1. Checking the input and output specifications of the job.
2. Computing the `InputSplit` values for the job.
3. Setting up the requisite accounting information for the `DistributedCache` of the job, if necessary.
4. Copying the job's jar and configuration to the Map/Reduce system directory on the `FileSystem`.
5. Submitting the job to the `JobTracker` and optionally monitoring it's status.

Job history files are also logged to user specified directory `hadoop.job.history.user.location` which defaults to job output directory. The files are stored in "_logs/history/" in the specified directory. Hence, by default they will be in mapred.output.dir/_logs/history. User can stop logging by giving the value `none` for `hadoop.job.history.user.location`

User can view the history logs summary in specified directory using the following command
`$ bin/hadoop job -history output-dir`
This command will print job details, failed and killed tip details.
More details about the job such as successful tasks and task attempts made for each task can be viewed using the following command
`$ bin/hadoop job -history all output-dir`

User can use OutputLogFilter to filter log files from the output directory listing.

Normally the user creates the application, describes various facets of the job via `JobConf`, and then uses the `JobClient` to submit the job and monitor its progress.

6.4.1. Job Control

Users may need to chain Map/Reduce jobs to accomplish complex tasks which cannot be done via a single Map/Reduce job. This is fairly easy since the output of the job typically goes to distributed file-system, and the output, in turn, can be used as the input for the next job.

However, this also means that the onus on ensuring jobs are complete (success/failure) lies squarely on the clients. In such cases, the various job-control options are:

- runJob(JobConf) : Submits the job and returns only after the job has completed.
- submitJob(JobConf) : Only submits the job, then poll the returned handle to the RunningJob to query status and make scheduling decisions.

Copyright © 2007 The Apache Software Foundation. All rights reserved.

- JobConf.setJobEndNotificationURI(String) : Sets up a notification upon job-completion, thus avoiding polling.

6.5. Job Input

InputFormat describes the input-specification for a Map/Reduce job.

The Map/Reduce framework relies on the `InputFormat` of the job to:

1. Validate the input-specification of the job.
2. Split-up the input file(s) into logical `InputSplit` instances, each of which is then assigned to an individual `Mapper`.
3. Provide the `RecordReader` implementation used to glean input records from the logical `InputSplit` for processing by the `Mapper`.

The default behavior of file-based `InputFormat` implementations, typically sub-classes of FileInputFormat, is to split the input into *logical* `InputSplit` instances based on the total size, in bytes, of the input files. However, the `FileSystem` blocksize of the input files is treated as an upper bound for input splits. A lower bound on the split size can be set via `mapred.min.split.size`.

Clearly, logical splits based on input-size is insufficient for many applications since record boundaries must be respected. In such cases, the application should implement a `RecordReader`, who is responsible for respecting record-boundaries and presents a record-oriented view of the logical `InputSplit` to the individual task.

TextInputFormat is the default `InputFormat`.

If `TextInputFormat` is the `InputFormat` for a given job, the framework detects input-files with the *.gz* and *.lzo* extensions and automatically decompresses them using the appropriate `CompressionCodec`. However, it must be noted that compressed files with the above extensions cannot be *split* and each compressed file is processed in its entirety by a single mapper.

6.5.1. InputSplit

InputSplit represents the data to be processed by an individual `Mapper`.

Typically `InputSplit` presents a byte-oriented view of the input, and it is the responsibility of `RecordReader` to process and present a record-oriented view.

FileSplit is the default `InputSplit`. It sets `map.input.file` to the path of the input file for the logical split.

Copyright © 2007 The Apache Software Foundation. All rights reserved.

6.5.2. RecordReader

RecordReader reads `<key, value>` pairs from an `InputSplit`.

Typically the `RecordReader` converts the byte-oriented view of the input, provided by the `InputSplit`, and presents a record-oriented to the `Mapper` implementations for processing. `RecordReader` thus assumes the responsibility of processing record boundaries and presents the tasks with keys and values.

6.6. Job Output

OutputFormat describes the output-specification for a Map/Reduce job.

The Map/Reduce framework relies on the `OutputFormat` of the job to:

1. Validate the output-specification of the job; for example, check that the output directory doesn't already exist.
2. Provide the `RecordWriter` implementation used to write the output files of the job. Output files are stored in a `FileSystem`.

`TextOutputFormat` is the default `OutputFormat`.

6.6.1. Task Side-Effect Files

In some applications, component tasks need to create and/or write to side-files, which differ from the actual job-output files.

In such cases there could be issues with two instances of the same `Mapper` or `Reducer` running simultaneously (for example, speculative tasks) trying to open and/or write to the same file (path) on the `FileSystem`. Hence the application-writer will have to pick unique names per task-attempt (using the attemptid, say `attempt_200709221812_0001_m_000000_0`), not just per task.

To avoid these issues the Map/Reduce framework maintains a special `${mapred.output.dir}/_temporary/_${taskid}` sub-directory accessible via `${mapred.work.output.dir}` for each task-attempt on the `FileSystem` where the output of the task-attempt is stored. On successful completion of the task-attempt, the files in the `${mapred.output.dir}/_temporary/_${taskid}` (only) are *promoted* to `${mapred.output.dir}`. Of course, the framework discards the sub-directory of unsuccessful task-attempts. This process is completely transparent to the application.

The application-writer can take advantage of this feature by creating any side-files required in `${mapred.work.output.dir}` during execution of a task via FileOutputFormat.getWorkOutputPath(), and the framework will promote them similarly for

Copyright © 2007 The Apache Software Foundation. All rights reserved.

succesful task-attempts, thus eliminating the need to pick unique paths per task-attempt.

Note: The value of `${mapred.work.output.dir}` during execution of a particular task-attempt is actually `${mapred.output.dir}/_temporary/_{$taskid}`, and this value is set by the Map/Reduce framework. So, just create any side-files in the path returned by FileOutputFormat.getWorkOutputPath() from map/reduce task to take advantage of this feature.

The entire discussion holds true for maps of jobs with reducer=NONE (i.e. 0 reduces) since output of the map, in that case, goes directly to HDFS.

6.6.2. RecordWriter

RecordWriter writes the output `<key, value>` pairs to an output file.

RecordWriter implementations write the job outputs to the `FileSystem`.

6.7. Other Useful Features

6.7.1. Counters

`Counters` represent global counters, defined either by the Map/Reduce framework or applications. Each `Counter` can be of any `Enum` type. Counters of a particular `Enum` are bunched into groups of type `Counters.Group`.

Applications can define arbitrary `Counters` (of type `Enum`) and update them via Reporter.incrCounter(Enum, long) or Reporter.incrCounter(String, String, long) in the `map` and/or `reduce` methods. These counters are then globally aggregated by the framework.

6.7.2. DistributedCache

DistributedCache distributes application-specific, large, read-only files efficiently.

`DistributedCache` is a facility provided by the Map/Reduce framework to cache files (text, archives, jars and so on) needed by applications.

Applications specify the files to be cached via urls (hdfs://) in the `JobConf`. The `DistributedCache` assumes that the files specified via hdfs:// urls are already present on the `FileSystem`.

The framework will copy the necessary files to the slave node before any tasks for the job are executed on that node. Its efficiency stems from the fact that the files are only copied once per job and the ability to cache archives which are un-archived on the slaves.

Copyright © 2007 The Apache Software Foundation. All rights reserved.

`DistributedCache` tracks the modification timestamps of the cached files. Clearly the cache files should not be modified by the application or externally while the job is executing.

`DistributedCache` can be used to distribute simple, read-only data/text files and more complex types such as archives and jars. Archives (zip, tar, tgz and tar.gz files) are *un-archived* at the slave nodes. Files have *execution permissions* set.

The files/archives can be distributed by setting the property `mapred.cache.{files|archives}`. If more than one file/archive has to be distributed, they can be added as comma separated paths. The properties can also be set by APIs DistributedCache.addCacheFile(URI,conf)/ DistributedCache.addCacheArchive(URI,conf) and DistributedCache.setCacheFiles(URIs,conf)/ DistributedCache.setCacheArchives(URIs,conf) where URI is of the form `hdfs://host:port/absolute-path#link-name`. In Streaming, the files can be distributed through command line option `-cacheFile/-cacheArchive`.

Optionally users can also direct the `DistributedCache` to *symlink* the cached file(s) into the `current working directory` of the task via the DistributedCache.createSymlink(Configuration) api. Or by setting the configuration property `mapred.create.symlink` as `yes`. The DistributedCache will use the `fragment` of the URI as the name of the symlink. For example, the URI `hdfs://namenode:port/lib.so.1#lib.so` will have the symlink name as `lib.so` in task's cwd for the file `lib.so.1` in distributed cache.

The `DistributedCache` can also be used as a rudimentary software distribution mechanism for use in the map and/or reduce tasks. It can be used to distribute both jars and native libraries. The DistributedCache.addArchiveToClassPath(Path, Configuration) or DistributedCache.addFileToClassPath(Path, Configuration) api can be used to cache files/jars and also add them to the *classpath* of child-jvm. The same can be done by setting the configuration properties `mapred.job.classpath.{files|archives}`. Similarly the cached files that are symlinked into the working directory of the task can be used to distribute native libraries and load them.

6.7.3. Tool

The Tool interface supports the handling of generic Hadoop command-line options.

`Tool` is the standard for any Map/Reduce tool or application. The application should delegate the handling of standard command-line options to GenericOptionsParser via ToolRunner.run(Tool, String[]) and only handle its custom arguments.

The generic Hadoop command-line options are:

Copyright © 2007 The Apache Software Foundation. All rights reserved.

```
-conf <configuration file>
-D <property=value>
-fs <local|namenode:port>
-jt <local|jobtracker:port>
```

6.7.4. IsolationRunner

IsolationRunner is a utility to help debug Map/Reduce programs.

To use the `IsolationRunner`, first set `keep.failed.tasks.files` to `true` (also see `keep.tasks.files.pattern`).

Next, go to the node on which the failed task ran and go to the `TaskTracker`'s local directory and run the `IsolationRunner`:

```
$ cd <local path>/taskTracker/${taskid}/work
$ bin/hadoop org.apache.hadoop.mapred.IsolationRunner
../job.xml
```

`IsolationRunner` will run the failed task in a single jvm, which can be in the debugger, over precisely the same input.

6.7.5. Profiling

Profiling is a utility to get a representative (2 or 3) sample of built-in java profiler for a sample of maps and reduces.

User can specify whether the system should collect profiler information for some of the tasks in the job by setting the configuration property `mapred.task.profile`. The value can be set using the api JobConf.setProfileEnabled(boolean). If the value is set `true`, the task profiling is enabled. The profiler information is stored in the the user log directory. By default, profiling is not enabled for the job.

Once user configures that profiling is needed, she/he can use the configuration property `mapred.task.profile.{maps|reduces}` to set the ranges of map/reduce tasks to profile. The value can be set using the api JobConf.setProfileTaskRange(boolean,String). By default, the specified range is `0-2`.

User can also specify the profiler configuration arguments by setting the configuration property `mapred.task.profile.params`. The value can be specified using the api JobConf.setProfileParams(String). If the string contains a `%s`, it will be replaced with the name of the profiling output file when the task runs. These parameters are passed to the task child JVM on the command line. The default value for the profiling parameters is `-agentlib:hprof=cpu=samples,heap=sites,force=n,thread=y,verbose=n,file=%s`

Copyright © 2007 The Apache Software Foundation. All rights reserved.

6.7.6. Debugging

Map/Reduce framework provides a facility to run user-provided scripts for debugging. When map/reduce task fails, user can run script for doing post-processing on task logs i.e task's stdout, stderr, syslog and jobconf. The stdout and stderr of the user-provided debug script are printed on the diagnostics. These outputs are also displayed on job UI on demand.

In the following sections we discuss how to submit debug script along with the job. For submitting debug script, first it has to distributed. Then the script has to supplied in Configuration.

6.7.6.1. How to distribute script file:

The user has to use DistributedCache mechanism to *distribute* and *symlink* the debug script file.

6.7.6.2. How to submit script:

A quick way to submit debug script is to set values for the properties "mapred.map.task.debug.script" and "mapred.reduce.task.debug.script" for debugging map task and reduce task respectively. These properties can also be set by using APIs JobConf.setMapDebugScript(String) and JobConf.setReduceDebugScript(String) . For streaming, debug script can be submitted with command-line options -mapdebug, -reducedebug for debugging mapper and reducer respectively.

The arguments of the script are task's stdout, stderr, syslog and jobconf files. The debug command, run on the node where the map/reduce failed, is:

```
$script $stdout $stderr $syslog $jobconf
```

Pipes programs have the c++ program name as a fifth argument for the command. Thus for the pipes programs the command is

```
$script $stdout $stderr $syslog $jobconf $program
```

6.7.6.3. Default Behavior:

For pipes, a default script is run to process core dumps under gdb, prints stack trace and gives info about running threads.

6.7.7. JobControl

JobControl is a utility which encapsulates a set of Map/Reduce jobs and their dependencies.

Copyright © 2007 The Apache Software Foundation. All rights reserved.

6.7.8. Data Compression

Hadoop Map/Reduce provides facilities for the application-writer to specify compression for both intermediate map-outputs and the job-outputs i.e. output of the reduces. It also comes bundled with CompressionCodec implementations for the zlib and lzo compression algorithms. The gzip file format is also supported.

Hadoop also provides native implementations of the above compression codecs for reasons of both performance (zlib) and non-availability of Java libraries (lzo). More details on their usage and availability are available here.

6.7.8.1. Intermediate Outputs

Applications can control compression of intermediate map-outputs via the JobConf.setCompressMapOutput(boolean) api and the `CompressionCodec` to be used via the JobConf.setMapOutputCompressorClass(Class) api.

6.7.8.2. Job Outputs

Applications can control compression of job-outputs via the FileOutputFormat.setCompressOutput(JobConf, boolean) api and the `CompressionCodec` to be used can be specified via the FileOutputFormat.setOutputCompressorClass(JobConf, Class) api.

If the job outputs are to be stored in the SequenceFileOutputFormat, the required `SequenceFile.CompressionType` (i.e. `RECORD` / `BLOCK` - defaults to `RECORD`) can be specified via the SequenceFileOutputFormat.setOutputCompressionType(JobConf, SequenceFile.CompressionType) api.

7. Example: WordCount v2.0

Here is a more complete `WordCount` which uses many of the features provided by the Map/Reduce framework we discussed so far.

This needs the HDFS to be up and running, especially for the `DistributedCache`-related features. Hence it only works with a pseudo-distributed or fully-distributed Hadoop installation.

7.1. Source Code

	WordCount.java
1.	`package org.myorg;`

Copyright © 2007 The Apache Software Foundation. All rights reserved.

```

import java.io.*;
import java.util.*;

import org.apache.hadoop.fs.Path;
import org.apache.hadoop.filecache.DistributedCache;
import org.apache.hadoop.conf.*;
import org.apache.hadoop.io.*;
import org.apache.hadoop.mapred.*;
import org.apache.hadoop.util.*;

public class WordCount extends Configured implements Tool {

  public static class Map extends MapReduceBase implements Mapper<LongWritable, Text, Text, IntWritable> {

    static enum Counters { INPUT_WORDS }

    private final static IntWritable one = new IntWritable(1);
    private Text word = new Text();

    private boolean caseSensitive = true;
    private Set<String> patternsToSkip = new
```


Copyright © 2007 The Apache Software Foundation. All rights reserved.

```
HashSet<String>();

  private long numRecords = 0;
  private String inputFile;

  public void configure(JobConf job) {
    caseSensitive = job.getBoolean("wordcount.case.sensitive", true);
    inputFile = job.get("map.input.file");

    if (job.getBoolean("wordcount.skip.patterns", false)) {
      Path[] patternsFiles = new Path[0];
      try {
        patternsFiles = DistributedCache.getLocalCacheFiles(job);
      } catch (IOException ioe) {
        System.err.println("Caught exception while getting cached files: " + StringUtils.stringifyException(ioe));
      }
      for (Path patternsFile : patternsFiles) {
        parseSkipFile(patternsFile);
      }
    }
```


Copyright © 2007 The Apache Software Foundation. All rights reserved.

```
  }

  private void parseSkipFile(Path patternsFile) {
   try {
    BufferedReader fis = new BufferedReader(new FileReader(patternsFile.toString()));
    String pattern = null;
    while ((pattern = fis.readLine()) != null) {
     patternsToSkip.add(pattern);
    }
   } catch (IOException ioe) {
    System.err.println("Caught exception while parsing the cached file '" + patternsFile + "' : " + StringUtils.stringifyException(ioe));
   }
  }

  public void map(LongWritable key, Text value, OutputCollector<Text, IntWritable> output, Reporter reporter) throws IOException {
   String line = (caseSensitive) ? value.toString() : value.toString().toLowerCase();

   for (String pattern : patternsToSkip) {
    line = line.replaceAll(pattern, "");
```


Copyright © 2007 The Apache Software Foundation. All rights reserved.

```
    }

    StringTokenizer tokenizer = new StringTokenizer(line);
    while (tokenizer.hasMoreTokens()) {
      word.set(tokenizer.nextToken());
      output.collect(word, one);
      reporter.incrCounter(Counters.INPUT_WORDS, 1);
    }

    if ((++numRecords % 100) == 0) {
      reporter.setStatus("Finished processing " + numRecords + " records " + "from the input file: " + inputFile);
    }
   }
  }

  public static class Reduce extends MapReduceBase implements Reducer<Text, IntWritable, Text, IntWritable> {
   public void reduce(Text key, Iterator<IntWritable> values, OutputCollector<Text, IntWritable> output, Reporter reporter) throws IOException {
    int sum = 0;
```


Copyright © 2007 The Apache Software Foundation. All rights reserved.

```
      while (values.hasNext()) {
        sum += values.next().get();
      }
      output.collect(key, new IntWritable(sum));
    }
  }

  public int run(String[] args) throws Exception {
    JobConf conf = new JobConf(getConf(), WordCount.class);
    conf.setJobName("wordcount");

    conf.setOutputKeyClass(Text.class);
    conf.setOutputValueClass(IntWritable.class);

    conf.setMapperClass(Map.class);
    conf.setCombinerClass(Reduce.class);
    conf.setReducerClass(Reduce.class);

    conf.setInputFormat(TextInputFormat.class);
    conf.setOutputFormat(TextOutputFormat.class);

    List<String> other_args = new
```


Copyright © 2007 The Apache Software Foundation. All rights reserved.

```
ArrayList<String>();
    for (int i=0; i < args.length; ++i) {
      if ("-skip".equals(args[i])) {
        DistributedCache.addCacheFile(new Path(args[++i]).toUri(), conf);
        conf.setBoolean("wordcount.skip.patterns", true);
      } else {
        other_args.add(args[i]);
      }
    }

    FileInputFormat.setInputPaths(conf, new Path(other_args.get(0)));
    FileOutputFormat.setOutputPath(conf, new Path(other_args.get(1)));

    JobClient.runJob(conf);
    return 0;
  }

  public static void main(String[] args) throws Exception {
    int res = ToolRunner.run(new Configuration(), new WordCount(), args);
    System.exit(res);
```


Copyright © 2007 The Apache Software Foundation. All rights reserved.

121.	}
122.	}
123.	

7.2. Sample Runs

Sample text-files as input:

```
$ bin/hadoop dfs -ls /usr/joe/wordcount/input/
/usr/joe/wordcount/input/file01
/usr/joe/wordcount/input/file02
$ bin/hadoop dfs -cat /usr/joe/wordcount/input/file01
Hello World, Bye World!
$ bin/hadoop dfs -cat /usr/joe/wordcount/input/file02
Hello Hadoop, Goodbye to hadoop.
```

Run the application:

```
$ bin/hadoop jar /usr/joe/wordcount.jar org.myorg.WordCount
/usr/joe/wordcount/input /usr/joe/wordcount/output
```

Output:

```
$ bin/hadoop dfs -cat /usr/joe/wordcount/output/part-00000
Bye 1
Goodbye 1
Hadoop, 1
Hello 2
World! 1
World, 1
hadoop. 1
to 1
```

Notice that the inputs differ from the first version we looked at, and how they affect the outputs.

Now, lets plug-in a pattern-file which lists the word-patterns to be ignored, via the `DistributedCache`.

```
$ hadoop dfs -cat /user/joe/wordcount/patterns.txt
\.
\,
```


Copyright © 2007 The Apache Software Foundation. All rights reserved.

```
\!
to
```

Run it again, this time with more options:

```
$ bin/hadoop jar /usr/joe/wordcount.jar org.myorg.WordCount
-Dwordcount.case.sensitive=true /usr/joe/wordcount/input
/usr/joe/wordcount/output -skip
/user/joe/wordcount/patterns.txt
```

As expected, the output:

```
$ bin/hadoop dfs -cat /usr/joe/wordcount/output/part-00000
Bye 1
Goodbye 1
Hadoop 1
Hello 2
World 2
hadoop 1
```

Run it once more, this time switch-off case-sensitivity:

```
$ bin/hadoop jar /usr/joe/wordcount.jar org.myorg.WordCount
-Dwordcount.case.sensitive=false /usr/joe/wordcount/input
/usr/joe/wordcount/output -skip
/user/joe/wordcount/patterns.txt
```

Sure enough, the output:

```
$ bin/hadoop dfs -cat /usr/joe/wordcount/output/part-00000
bye 1
goodbye 1
hadoop 2
hello 2
world 2
```

7.3. Highlights

The second version of `WordCount` improves upon the previous one by using some features offered by the Map/Reduce framework:

- Demonstrates how applications can access configuration parameters in the `configure` method of the `Mapper` (and `Reducer`) implementations (lines 28-43).
- Demonstrates how the `DistributedCache` can be used to distribute read-only data

Copyright © 2007 The Apache Software Foundation. All rights reserved.

needed by the jobs. Here it allows the user to specify word-patterns to skip while counting (line 104).

- Demonstrates the utility of the `Tool` interface and the `GenericOptionsParser` to handle generic Hadoop command-line options (lines 87-116, 119).
- Demonstrates how applications can use `Counters` (line 68) and how they can set application-specific status information via the `Reporter` instance passed to the `map` (and `reduce`) method (line 72).

Java and JNI are trademarks or registered trademarks of Sun Microsystems, Inc. in the United States and other countries.

Copyright © 2007 The Apache Software Foundation. All rights reserved.
