# Supplementary material for: Provenance based data integrity checking and verification in cloud environments
Source: PLoS One. 2017 May 17;12(5):e0177576. doi: 10.1371/journal.pone.0177576 (PMC5435237; doi:10.1371/journal.pone.0177576)
Supplement: S1 Dataset — Files in the Dataset are utilized for performance measurement. (ZIP) [file pone.0177576.s001.zip › dataset/pdf/native_libraries - Copy (2) - Copy.pdf]

Native Hadoop Libraries

Table of contents

1 Purpose....................2
2 Components....................2
3 Usage....................2
4 Supported Platforms....................3
5 Building Native Hadoop Libraries....................3
5.1 Notes....................4
6 Loading native libraries through DistributedCache....................4

Copyright © 2007 The Apache Software Foundation. All rights reserved.

1. Purpose

Hadoop has native implementations of certain components for reasons of both performance and non-availability of Java implementations. These components are available in a single, dynamically-linked, native library. On the *nix platform it is *libhadoop.so*. This document describes the usage and details on how to build the native libraries.

2. Components

Hadoop currently has the following compression codecs as the native components:

- zlib
- gzip
- lzo

Of the above, the availability of native hadoop libraries is imperative for the lzo and gzip compression codecs to work.

3. Usage

It is fairly simple to use the native hadoop libraries:

- Take a look at the supported platforms.
- Either download the pre-built 32-bit i386-Linux native hadoop libraries (available as part of hadoop distribution in `lib/native` directory) or build them yourself.
- Make sure you have either or both of **>zlib-1.2** and **>lzo2.0** packages for your platform installed; depending on your needs.

The `bin/hadoop` script ensures that the native hadoop library is on the library path via the system property *-Djava.library.path=<path>*.

To check everything went alright check the hadoop log files for:

```
DEBUG util.NativeCodeLoader - Trying to load the custom-built
native-hadoop library...
INFO util.NativeCodeLoader - Loaded the native-hadoop library
```

If something goes wrong, then:

```
INFO util.NativeCodeLoader - Unable to load native-hadoop
library for your platform... using builtin-java classes where
applicable
```


Copyright © 2007 The Apache Software Foundation. All rights reserved.

4. Supported Platforms

Hadoop native library is supported only on *nix platforms only. Unfortunately it is known not to work on Cygwin and Mac OS X and has mainly been used on the GNU/Linux platform.

It has been tested on the following GNU/Linux distributions:

- RHEL4/Fedora
- Ubuntu
- Gentoo

On all the above platforms a 32/64 bit Hadoop native library will work with a respective 32/64 bit jvm.

5. Building Native Hadoop Libraries

Hadoop native library is written in ANSI C and built using the GNU autotools-chain (autoconf, autoheader, automake, autoscan, libtool). This means it should be straight-forward to build them on any platform with a standards compliant C compiler and the GNU autotools-chain. See supported platforms.

In particular the various packages you would need on the target platform are:

- C compiler (e.g. GNU C Compiler)
- GNU Autools Chain: autoconf, automake, libtool
- zlib-development package (stable version >= 1.2.0)
- lzo-development package (stable version >= 2.0)

Once you have the pre-requisites use the standard `build.xml` and pass along the `compile.native` flag (set to `true`) to build the native hadoop library:

```
$ ant -Dcompile.native=true <target>
```

The native hadoop library is not built by default since not everyone is interested in building them.

You should see the newly-built native hadoop library in:

```
$ build/native/<platform>/lib
```

where <platform> is combination of the system-properties: `${os.name}-${os.arch}-${sun.arch.data.model}`; for e.g. Linux-i386-32.

Copyright © 2007 The Apache Software Foundation. All rights reserved.

5.1. Notes

- It is **mandatory** to have both the zlib and lzo development packages on the target platform for building the native hadoop library; however for deployment it is sufficient to install zlib or lzo if you wish to use only one of them.
- It is necessary to have the correct 32/64 libraries of both zlib/lzo depending on the 32/64 bit jvm for the target platform for building/deployment of the native hadoop library.

6. Loading native libraries through DistributedCache

User can load native shared libraries through DistributedCache for *distributing* and *symlinking* the library files

Here is an example, describing how to distribute the library and load it from map/reduce task.

1. First copy the library to the HDFS.
   ```
   bin/hadoop fs -copyFromLocal mylib.so.1
   /libraries/mylib.so.1
   ```
2. The job launching program should contain the following:
   ```
   DistributedCache.createSymlink(conf);
   DistributedCache.addCacheFile("hdfs://host:port/libraries/mylib.so.1#m
   conf);
   ```
3. The map/reduce task can contain:
   ```
   System.loadLibrary("mylib.so");
   ```


Copyright © 2007 The Apache Software Foundation. All rights reserved.
