# Supplementary material for: Provenance based data integrity checking and verification in cloud environments
Source: PLoS One. 2017 May 17;12(5):e0177576. doi: 10.1371/journal.pone.0177576 (PMC5435237; doi:10.1371/journal.pone.0177576)
Supplement: S1 Dataset — Files in the Dataset are utilized for performance measurement. (ZIP) [file pone.0177576.s001.zip › dataset/pdf/quickstart - Copy (2) - Copy.pdf]

Hadoop Quickstart

Table of contents

1 Purpose..2
2 Pre-requisites..2
2.1 Supported Platforms...2
2.2 Required Software...2
2.3 Installing Software...2
3 Download..2
4 Prepare to Start the Hadoop Cluster...3
5 Standalone Operation..3
6 Pseudo-Distributed Operation...3
6.1 Configuration...3
6.2 Setup passphraseless ssh...4
6.3 Execution..4
7 Fully-Distributed Operation...5

Copyright © 2007 The Apache Software Foundation. All rights reserved.

1. Purpose

The purpose of this document is to help you get a single-node Hadoop installation up and running very quickly so that you can get a flavour of the Hadoop Distributed File System (HDFS) and the Map/Reduce framework; that is, perform simple operations on HDFS and run example jobs.

2. Pre-requisites

2.1. Supported Platforms

- GNU/Linux is supported as a development and production platform. Hadoop has been demonstrated on GNU/Linux clusters with 2000 nodes.
- Win32 is supported as a *development platform*. Distributed operation has not been well tested on Win32, so it is not supported as a *production platform*.

2.2. Required Software

Required software for Linux and Windows include:

1. JavaTM 1.5.x, preferably from Sun, must be installed.
2. **ssh** must be installed and **sshd** must be running to use the Hadoop scripts that manage remote Hadoop daemons.

Additional requirements for Windows include:

1. Cygwin - Required for shell support in addition to the required software above.

2.3. Installing Software

If your cluster doesn't have the requisite software you will need to install it.

For example on Ubuntu Linux:

```
$ sudo apt-get install ssh
$ sudo apt-get install rsync
```

On Windows, if you did not install the required software when you installed cygwin, start the cygwin installer and select the packages:

- openssh - the *Net* category

3. Download

Copyright © 2007 The Apache Software Foundation. All rights reserved.

To get a Hadoop distribution, download a recent stable release from one of the Apache Download Mirrors.

4. Prepare to Start the Hadoop Cluster

Unpack the downloaded Hadoop distribution. In the distribution, edit the file `conf/hadoop-env.sh` to define at least `JAVA_HOME` to be the root of your Java installation.

Try the following command:
```
$ bin/hadoop
```
This will display the usage documentation for the **hadoop** script.

Now you are ready to start your Hadoop cluster in one of the three supported modes:

- Local (Standalone) Mode
- Pseudo-Distributed Mode
- Fully-Distributed Mode

5. Standalone Operation

By default, Hadoop is configured to run in a non-distributed mode, as a single Java process. This is useful for debugging.

The following example copies the unpacked `conf` directory to use as input and then finds and displays every match of the given regular expression. Output is written to the given `output` directory.
```
$ mkdir input
$ cp conf/*.xml input
$ bin/hadoop jar hadoop-*-examples.jar grep input output
'dfs[a-z.]+'
$ cat output/*
```

6. Pseudo-Distributed Operation

Hadoop can also be run on a single-node in a pseudo-distributed mode where each Hadoop daemon runs in a separate Java process.

6.1. Configuration

Use the following `conf/hadoop-site.xml`:

```
<configuration>
```


Copyright © 2007 The Apache Software Foundation. All rights reserved.

```
  <property>
    <name>fs.default.name</name>
    <value>localhost:9000</value>
  </property>
  <property>
    <name>mapred.job.tracker</name>
    <value>localhost:9001</value>
  </property>
  <property>
    <name>dfs.replication</name>
    <value>1</value>
  </property>
</configuration>
```

6.2. Setup passphraseless ssh

Now check that you can ssh to the localhost without a passphrase:

```
$ ssh localhost
```

If you cannot ssh to localhost without a passphrase, execute the following commands:

```
$ ssh-keygen -t dsa -P '' -f ~/.ssh/id_dsa
$ cat ~/.ssh/id_dsa.pub >> ~/.ssh/authorized_keys
```

6.3. Execution

Format a new distributed-filesystem:

```
$ bin/hadoop namenode -format
```

Start the hadoop daemons:

```
$ bin/start-all.sh
```

The hadoop daemon log output is written to the `${HADOOP_LOG_DIR}` directory (defaults to `${HADOOP_HOME}/logs`).

Browse the web interface for the NameNode and the JobTracker; by default they are

Copyright © 2007 The Apache Software Foundation. All rights reserved.

available at:

- `NameNode` - http://localhost:50070/
- `JobTracker` - http://localhost:50030/

Copy the input files into the distributed filesystem:
```
$ bin/hadoop fs -put conf input
```

Run some of the examples provided:
```
$ bin/hadoop jar hadoop-*-examples.jar grep input output
'dfs[a-z.]+'
```

Examine the output files:

Copy the output files from the distributed filesystem to the local filesytem and examine them:
```
$ bin/hadoop fs -get output output
$ cat output/*
```

or

View the output files on the distributed filesystem:
```
$ bin/hadoop fs -cat output/*
```

When you're done, stop the daemons with:
```
$ bin/stop-all.sh
```

7. Fully-Distributed Operation

Information on setting up fully-distributed, non-trivial clusters can be found here.

Java and JNI are trademarks or registered trademarks of Sun Microsystems, Inc. in the United States and other countries.

Copyright © 2007 The Apache Software Foundation. All rights reserved.
